# Supplementary material for: Factors associated with accessing and utilisation of healthcare and provision of health services for residents of slums in low and middle-income countries: a scoping review of recent literature
Source: BMJ Open. 2022 May 20;12(5):e055415. doi: 10.1136/bmjopen-2021-055415 (PMC9125718; doi:10.1136/bmjopen-2021-055415)
Supplement: Supplementary data [file bmjopen-2021-055415supp001.pdf]

Appendix 1. Search strategy and the result of each database.

Database	Search strategy	Number of studies
Ovid Medline	1. (informal* and settlement*).ti,ab,kw.<br>2. (shanty and town*).ti,ab,kw.<br>3. (favela* or ghetto* or shantytown* or shanty-town* or slum or slums).ti,ab,kw.<br>4. or/1-3<br>5. limit 4 to english language	4,688
Embase	1 (informal* and settlement*).ti,ab,kw.<br>2 (shanty and town*).ti,ab,kw.<br>3 (favela* or ghetto* or shantytown* or shanty-town* or slum or slums).ti,ab,kw.<br>4 or/1-3<br>5 limit 4 to english language	5,090
Web of Science	1 (TS=(favela* OR ghetto* OR shantytown* OR shanty-town* OR slum OR slums)) AND language: (English)<br>2 ((TS=(informal* NEAR settlement*))) AND language: (English)<br>3 (TS=(shanty NEAR town*)) AND language: (English)<br>4 (#1 OR #2 OR #3) AND language: (English)	3,553
Cochrane	1 (informal* and settlement*).ti,ab,kw.<br>2 (shanty and town*).ti,ab,kw.<br>3 (favela* or ghetto* or shantytown* or shanty-town* or slum or slums).ti,ab,kw.<br>4 #1 or #2 or #3	381
CINAHL	1 TI (informal* and settlement*) OR AB (informal* and settlement*)<br>2 TI (shanty and town*) OR AB (shanty and town*)<br>3 TI (favela* or ghetto* or shantytown* or shanty-town* or slum or slums) OR AB (favela* or ghetto* or shantytown* or shanty-town* or slum or slums)<br>S1 OR S2 OR S3	1,757
