# Supplementary material for: Factors associated with accessing and utilisation of healthcare and provision of health services for residents of slums in low and middle-income countries: a scoping review of recent literature
Source: BMJ Open. 2022 May 20;12(5):e055415. doi: 10.1136/bmjopen-2021-055415 (PMC9125718; doi:10.1136/bmjopen-2021-055415)
Supplement: Supplementary data [file bmjopen-2021-055415supp002.pdf]

Supplement 1. Healthcare-seeking behaviours of slum residents reported by included studies and associated factors.

Subcategory	Author (year)	Participants	Country	Study design	Methodology	Outcome	Factors of interest
General healthcare seeking behaviour	Pakhare (2021)[55]	Slum residents diagnosed hypertension or diabetes	India	Prospective study	Quantitative	Linking to healthcare facilities	Age; wealth; distance to facilities ; early engagement by healthcare workers
	Gaiha (2020)[121]	Hetero-couples in slums	India	Cross-sectional study	Mixed method	Ability to attend any health promotion activity	Lack of time related to work as a reason for low male participation
	van der Heijden (2019)[120]	Female workers and key informants in slums	Bangladeshi	Cross-sectional study	Qualitative	Healthcare-seeking behaviour	Competing interest (ability to work and income)
	Aleemi (2018)[118]	Slum residents	Pakistan	Cross-sectional study	Quantitative	Healthcare-seeking behaviour	Household income; government facility; NGO facility
	Wekesah (2019)[139]	Slum residents	Kenya	Cross-sectional study	Qualitative	Care-seeking and adherence to treatment for CVD	Cost of healthcare; lack of healthcare facilities
	Kar (2017)[58]	Slum residents	India	Cross-sectional study	Quantitative	Undiagnosed hypertension	Sex; poverty; unskilled laborer; literacy
	Mistry (2016)[71]	TB patients in slums	India	Retrospective study	Quantitative	Delays in care seeking	Perception of symptoms; home remedies; not want to miss work; provider shopping; delay in advising TB-relevant tests; referral.
	Kulkarni (2016)[70]	Women in slums	India	Cross-sectional study	Quantitative	Participation in breast cancer screening	Age; education; religion; Mother tongue; occupation; marital status; tobacco habits; family history of cancer; history of cancer screening
	Misra (2017)[101]	Slum households	India	Cross-sectional study	Quantitative	Health-seeking practice for cataract	Lack of time, fear of surgery, financial difficulties

	Ramagiri (2020)[77]	Slum residents with diabetes	India	Case control study	Mixed-method	Uptake of diabetic retinopathy screening	Realization of consequences of disease; travel assistance and proximity of the screening facility; absence of an accompanying person; cost
Healthcare for children	Mohanty (2021)[54]	Caregivers of under-five children in urban slums,	India	Cross-sectional study	Quantitative	Healthcare seeking for children	Sex of child; size of the household; social group of caregiver, mother with mass media knowledge; age of mother; education and occupation of mother; suffering from chronic disease; decision making person for seeking health care; time lapse in approaching the health care facility; income loss due to children illnesses
	Lungu (2020)[53]	Caregivers of children under 5 years of age in slums	Malawi	Cross-sectional study	Quantitative	Healthcare-seeking behaviour	Age; illness was perceived to be severe; fever; home management of childhood illness
						Timely healthcare seeking behaviour	Home management of childhood; knowledge of caregivers about child danger signs
	McNairy (2019)[19]	Slum households with children ≤ 5 years old	Haiti	Cross-sectional study	Quantitative	Healthcare access	Inability to afford care
	Hutain (2019)[100]	Caregiver at the time of the child's death in slums	Sierra Leone	Cross-sectional study	Mixed-method	Health care-seeking	Use of traditional medicine; difficultly reaching the health facility; doubts about need for medical care; mistreatment by staff
	Kerai (2019)[56]	Caregiver of children aged 2 months to 5 years in slums	Pakistan	Cross-sectional study	Quantitative	Healthcare-seeking behaviour	Age of child; gender of child; income; education of caretaker; vaccine awareness; breastfeeding awareness;

							presence of symptoms such as fever, tachypnea, chest indrawing, persistent vomiting, recurrent illness.
	Lungu (2018)[110]	Caregivers of children under 5 years of age in slums	Malawi	Prospective study	Quantitative	Healthcare-seeking behaviour	Cost; waiting time; availability of medicines and supplies; attitude of health workers; thorough examination of the child
						Willingness to pay for the health facility	Waiting time; availability of medicine and equipment; superficial or thorough examination; attitude of health workers
	Kamati (2019)[73]	Slum residents	Namibia	Cross-sectional study	Mixed-method	Self-medication	Perceived diagnosis as "minor or mild"; waiting times and queues to receive care
	Mishra (2017)[65]	Mothers living in slums with a child and migrated recently	India	Cross-sectional study	Quantitative	Healthcare seeking behaviour	Symptoms and severity
	Lungu (2016)[85]	Caregivers and health providers in slums	Malawi	Longitudinal study	Qualitative	Healthcare-seeking behaviour	Home management; lack of medicines and supplies; waiting times; facility opening times; attitude of health workers; suboptimal examination of the sick child; distance to health facility; cost of healthcare
Healthcare for women	Muralidharan (2019)[123]	Girls and mothers in slums	India	Cross-sectional study	Qualitative	Healthcare-seeking behaviour	Proximity of healthcare facilities
	Nasrin (2019)[111]	Married women with a child in slums	Bangladesh	Cross-sectional study	Mixed-method	Healthcare-seeking behaviours	Inability to spend the treatment cost

	Jayaweera (2018)[79]	Girls and women in slums	Kenya	Cross-sectional study	Qualitative	Access to contraception and abortion in health facilities	Stigma; lack of education about safe methods of abortion; perceived illegality of abortion; limited access to services because of financial barrier; fear of mistreatment and mistrust of health providers/facilities; geographical proximity
	Williams (2018)[130]	Mothers and medical personnel in slums	Bangladesh	Cross-sectional study	Qualitative	Mental healthcare seeking	Culture and stigma
	Ilankoo (2018)[78]	Women in slums	Sri Lanka	Cross-sectional study	Qualitative	Health-seeking behaviours related to vaginal discharge	Confusion in differentiating normal from abnormal vaginal discharge; effects on day-to-day life; confusion toward the causative factors; difficulties in disclosing; neglecting behaviours; and socio-cultural influences toward health-seeking behaviours.
	Athie (2017)[131]	Anxious and depressed women in slums	Brazil	Cross-sectional study	Qualitative	Healthcare seeking behaviour	High medical turnover and overload of healthcare providers
	Sudhinaraset (2016)[90]	Mothers and their families in slums	India	Cross-sectional study	Qualitative	Maternal health services and delivery experiences	Financial barriers; disrespectful care
	Pune Municipal corporation[38]	Recently delivered slum residents	India	Cross-sectional study	Mixed-method	Seeking front-line worker during labor	No time to call; family did not allow; being out of town; lack of trust; delivery at night
						Going to the Referred Place for Pregnancy Complications	Not necessary; family did not allow; lack of trust/poor quality services; don't like going to a difference facility; too far; cost; no transportation; private hospital
Preference for	Das (2018)[102]	Slum	India	Cross-	Qualitative	Healthcare-seeking	Female prefer informal healers

healthcare providers		residents		sectional study		practice (preference for formal/informal healers)	(cultural competency of care, easy communication, gender-induced affordability, avoidance of social stigma and labelling, living with the burden of cultural expectations and geographical and cognitive distance of formal health care)
							Male prefer formal care (ease of access, quality of treatment, expected outcome of therapies)
	Angeli (2018)[104]	Slum residents	India	Cross-sectional study	Mixed-method	Choice between public or private hospital	Bottom-of-the pyramid patients visit a public hospital more than top-of-the-pyramid patients
Health insurance	Kalyango* (2021)[137]	Households in slum and non-slums	Uganda	Cross-sectional study	Qualitative	Willingness to pay for health insurance	Public and private providers; extended family enrolment
HIV testing	Thomson (2018)[72]	Stakeholder including residents and healthcare service provider	Kenya	Cross-sectional study	Qualitative	HIV testing	Denial; complacency; fear of death; anticipation of unbearable stress; felt ill; had a partner die; learned that their partner was HIV-positive.
Expenditure	Mishra (2017)[59]	Slum households with a child aged 0–14 years and who had migrated within the last 12 years	India	Cross-sectional study	Quantitative	Treatment-seeking behaviour	Child's gender

*Factors reported in the study were associated with participants covering both slum and non-slum residents. CVD: cardiovascular disease; HIV: human immunodeficiency virus; NGO: non-governmental organization; TB: tuberculosis.
