# Supplementary material for: Factors associated with accessing and utilisation of healthcare and provision of health services for residents of slums in low and middle-income countries: a scoping review of recent literature
Source: BMJ Open. 2022 May 20;12(5):e055415. doi: 10.1136/bmjopen-2021-055415 (PMC9125718; doi:10.1136/bmjopen-2021-055415)
Supplement: Supplementary data [file bmjopen-2021-055415supp004.pdf]

Supplement 3. Provision of healthcare services in slums examined by included studies and associated factors

Subcategory	Author (year)	Participants	Country	Study design	Methodology	Outcome	Factors of interest
General provision	Banerjee (2021)[145]	Community-level service providers in the selected city of Nagpur, Maharashtra.	India	Cross-sectional study	Mixed-methods	Implementing urban health and nutrition day	Unserved areas and left-out urban slum pockets; the distribution paradox of Urban Health and Nutrition Day location with an ill-defined geographic boundary; restriction of range of services to antenatal registration and immunisation with gross neglect of other components; suboptimal training of staff; insufficient availability of space, logistics, and health manpower; non-involvement of community members and Urban Local Bodies; and poor monitoring and supervision.
	Muhammad (2021)[129]	Caregivers of children, community influencers, immunisation staff in peri-urban slums	Pakistan	Cross-sectional study	Mixed-method	Childhood vaccination	Underperformance of staff; unreliable immunisation and household data; inefficient utilization of funds; interference of polio campaigns with immunisation
	Kaba (2020)[74]	Stakeholders (community members, community opinion leaders, Urban Health Extension Professionals, and	Ethiopia	Cross-sectional study	Qualitative	Provision of health services	Institutional-level: medical supplies; a lack of passion; attitudes on the part of health service providers<br><br>Community level: shared understanding of the

	city health office representatives.)					problems; services and the community's established values in relation to the problems and services.
Das Gupta (2020)[143]	N/A	India	Case study	Mixed-method	Improving public health services	Devolution of service delivery transferring funds and responsibilities to elected local bodies; management by professional managerial and technical cadres; Tight organisation of public health services; Professional support from the state directorate of public health
Ongarora (2019)[146]	Private healthcare facilities	Kenya	Cross-sectional study	Quantitative	Provision of medicine	Medicine price, affordability and availability of medicine
Agonigi (2018)[142]	Health professionals	Brazil	Cross-sectional study	Qualitative	Production of care in the daily work of health professionals	Issues related to assignment of tasks; inadequate space and equipment; requirement to follow standardised protocol; demands from the management; workload; environment (sanitation, territory); violence; registration
Odhiambo (2016)[140]	Community health workers	Kenya	Longitudinal study	Quantitative	Drug administration activities for schistosomiasis	Community health worker familiarity with households led to warm reception; good knowledge of intervention area by community health workers; high demand for drugs in the final year of treatment; effective community mobilization; opportunity to integrate mass drug administration with other health interventions; presence of community health workers and their supervisory structure, and

							points of referral for serious side effects; fear of side effects, size of tablet and misconceptions regarding treatment; unrelated death and the associated negative publicity by the media; religious beliefs and mistrust of interventions; insufficient time; absence of community members during the drug administration exercise; difficulty in directly observing treatment; unsanitary environmental conditions; inaccessibility (filthy and bush environment); demand for incentives by community members to take drugs.
	Patil (2016)[141]	Healthcare service centres	India	Cross-sectional study	Quantitative	Services provided under Integrated Child Development Services	Lack of basic infrastructural facilities; absence of essential drugs, equipment and logistics; poor pay scale, untimely drug supply, poor community support, more of documentation work, increased work burden, lack of supportive staff and no incentives for the increased work
	Mataboge (2016)[133]	Health services' clients and healthcare providers in an informal settlement	South Africa	Cross-sectional study	Qualitative	Provision of reproductive healthcare services	Healthcare policies; work overload; community-based care
	Prado Junior (2016)[144]	New TB cases living in slum and non-slum	Brazil	Cross-sectional study	Quantitative	Coverage under Family Health system for TB patients	Policy prioritizing low social development areas

TB: tuberculosis
