# Supplementary material for: Factors associated with accessing and utilisation of healthcare and provision of health services for residents of slums in low and middle-income countries: a scoping review of recent literature
Source: BMJ Open. 2022 May 20;12(5):e055415. doi: 10.1136/bmjopen-2021-055415 (PMC9125718; doi:10.1136/bmjopen-2021-055415)
Supplement: Supplementary data [file bmjopen-2021-055415supp003.pdf]

Supplement 2. Healthcare utilisation of slum residents reported by included studies and associated factors

Subcategory	Author (year)	Participants	Country	Study design	Methodology	Outcome	Factors of interest
General utilisation	Wambiya (2021)[64]	Slum household members	Kenya	Cross-sectional study	Quantitative	Private and public healthcare utilisation	Public- satisfaction with cost; satisfaction with healthcare quality; having acute infection or other diseases<br><br>Private- insurance coverage; having acute infection
	Chauhan (2020)[96]	Elderly slum residents	India	Cross-sectional study	Quantitative	Utilization of healthcare services	Unawareness of healthcare facilities; behaviour of service providers; distance from home; transport facility; amenities at healthcare facilities; convenience for attendants
	Otieno (2020)[134]	Slum household members	Kenya	Cross-sectional study	Quantitative	Access to primary healthcare services	Sex of household head; average out-of-pocket healthcare expenditure; source of primary care
	Vora (2020)[46]	Slum household members	India	Cross-sectional study	Quantitative	Unmet need for surgical services	Financial reasons; lack of trust; age; sex; type of problem
	Agrawal (2019)[115]	Older adults in slums	India	Cross-sectional study	Quantitative	Utilisation of welfare schemes	Religion; Caste; education;
	Ahmed (2019)[128]	N/A	Bangladesh	Cross-sectional study	Quantitative	Access to, and availability of healthcare services	Variability in traffic congestion
	Madan (2019)[87]	Female slum residents	India	Cross-sectional study	Qualitative	Access to primary care	Long waiting times and opening times of the primary health care; quality of services; satisfaction with treatments; home remedies; cost; rude attitude of healthcare providers
	Owiti (2018)[86]	Pregnant women in slums	Kenya	Cross-sectional study	Quantitative	Utilisation of maternal health services in public	Perception about public health facility delivery; living within close proximity; waiting time at

					health facilities	the facility; learning about the program; quality of service; ANC attendance at a private and a non-profit health facility
Castiglione (2018)[112]	Slum residents	Brazil	Cross-sectional study	Qualitative	Barrier to healthcare services	*Public healthcare services:* structural aspects of the healthcare system in their community as a whole, such as scarcity of personnel and equipment, or long waiting periods; experiences of conflict when dealing with doctors and other professionals of the public healthcare system<br><br>*Private healthcare services*: Insufficient funds to seek assistance; services or products in the private sector;
Tabrizi* (2018)[114]	Households in slum and non-slums	Iran	Cross-sectional study	Quantitative	Utilisation of health services	High cost of services
					Home care services	High cost of services
					Not taking drugs prescribed	Slums:<br>financial problems<br><br>Non-slums:<br>getting better/feeling well
Wairiuko (2017)[88]	Elderly in slums	Kenya	Cross-sectional study	Mixed-method	Health service utilisation	Family support; satisfaction with healthcare services; gender healthcare worker preference; services by community health worker
Owusu-Ansah (2016)[83]	Slum residents	Ghana	Cross-sectional study	Qualitative	Utilization of healthcare	Education; occupation; NHIS membership; knowledge of symptom; overall knowledge score; transportation

	Adane (2017)[81]	Mothers/caregivers of under-five children in slums	Ethiopia	Cross-sectional study	Quantitative	Utilization of healthcare facilities in children with diarrhoea	Mothers/caregivers education; occupation; time of walking to the nearest health facility; household monthly income; recognized danger signs
	MacPherson (2019)[124]	Slum residents	Malawi	Prospective study	Quantitative	Access to TB diagnosis	Distance to the nearest TB registration clinic
	Wingfield (2017)[122]	Slum households with patients treated for TB	Peru	Randomized controlled study	Quantitative	Initiation of TB preventive therapy	Socioeconomic support and social support
	Iberico (2016)[99]	Healthcare workers and community members in slums	Peru	Cross-sectional study	Qualitative	Utilization of TB preventive therapy	Misunderstanding and fear of treatment
	Snyder* (2016) [52]	TB patients living in slum and non-slum	Brazil	Retrospective study	Quantitative	Abandonment of TB treatment	Residency in a slum; sex; age; extrapulmonary clinical disease; HIV/AIDS; interaction (directly observed treatment × residency in a slum)
	Oluoch (2017)[97]	Slum residents	Nairobi	Cross-sectional study	Quantitative	Attendance to HIV testing and counselling services	Previous test experience
	Martinez Perez (2016)[89]	Healthcare workers and community members in slums	South Africa	Cross-sectional study	Mixed method	HIV Counselling and Testing	Fear; lack of trust
	Amiresmaili (2019)[18]	Slum residents	India	Cross-sectional study	Quantitative	Utilisation of outpatients services	Gender; marital status
						Utilisation of inpatients services	Age of household head; marital status; insurance
	Horng (2019)[49]	Slum households with children under 5 years old who either recently	Bangladesh	Cross-sectional study	Quantitative	Healthcare utilisation in severe acute respiratory illness	Relocation; age of child; education of mother; household wealth; health service knowledge

		relocated <12 months or who were residentially stable living >24 months				Full vaccination coverage	Relocation; number of children in household; age of child; education of mother; occupation of household head; household wealth; health service knowledge
	Kuria (2018)[132]	Patients received hypertension treatment in slums	Kenya	Retrospective study	Quantitative	Compliance with hypertensive treatment	Health facility group than walkway or weekend clinic attenders
	Cernauskas (2018)[125]	Slum residents	India	Cross-sectional study	Quantitative	Health provider choice	Distance to health facilities; friendly attitude of healthcare workers; appropriate service; familiarity
	Kaba (2020)[74]	Stakeholders (community members, community opinion leaders, health professionals, health office representatives.)	Ethiopia	Cross-sectional study	Qualitative	Utilisation of health services	Individual level: awareness about health problems; competing priorities; capacity to pay for services when referred.
	Mataboge (2016)[133]	Health services' clients and healthcare providers in an informal settlement	South Africa	Cross-sectional study	Qualitative	Healthcare utilisation	Long waiting time
Immunisation	Muhammad (2021)[129]	Caregivers of children, community influencers, immunisation staff in peri-urban slums	Pakistan	Cross-sectional study	Mixed-method	Childhood vaccination	Permission for immunisation by decision-maker; lack of knowledge and awareness of the benefit of immunisation; misconceptions and fears regarding vaccines; social and religious barriers
	de Araujo Veras (2020)[45]	Children in slums	Brazil	Cross-sectional study	Quantitative	Childhood vaccination	Age of child: mother's education

Mutua (2020)[106]	Children in slums	Nairobi	Prospective study	Quantitative	Full and on-time vaccination coverage	Place of residence; wealth
Roja (2020)[44]	Mothers of children in slums	India	Cross-sectional study	Quantitative	Immunisation status of children	Number of children in family; age of child; father's education
Obanewa (2020)[60]	Rural/urban formal/slum residents	Nigeria	Retrospective cross-sectional study	Quantitative	Fully-immunized child coverage	For slums:<br>delivery place; maternal education; birth order; antenatal attendance; religion<br><br>For slum and non-slums:<br>year; birth order; antenatal attendance; maternal education; religion; maternal age at child's birth; media exposure; region of the country; interaction between place of residence and place of delivery
Viramgami (2019)[119]	Married slum residents in reproductive age	India	Cross-sectional study	Quantitative	Vaccination status of child	Mother's employment
Singh (2018)[68]	N/A	India	Literature review	-	Childhood vaccination	Fear of adverse events; lack of information/knowledge; disease not harmful/serious; parents busy; income; mother's education; travel/transfer/migration; unawareness of need for health services; faith in immunisation; mother ill; forgetfulness; lack of initiative; family problems; services not available/lack of facility; shortages/reluctant to open 10 dose vials for 1 or 2 infants; current/history of sickness lead to withhold the vaccine

	Pugliese-Garcia (2018)[76]	Stakeholders including slum residents, healthcare workers, health committee members, vaccinators	Zambia	Cross-sectional study	Qualitative	Vaccine hesitancy	Traditional remedies; alcohol use; religious beliefs; distrust towards western medicine; previous adverse events; fear of injections and low perceived need for immunisation; limited understanding of how vaccines work; overlapping local terms for vaccine; pain; perceived risk of infection
	Manandhar (2018)[93]	Slum household with children age of 12-60 months	Nepal	Cross-sectional study	Quantitative	Incomplete immunisation	Knowledge on immunisation schedule
	Dasgupta (2018)[116]	Slum household with children aged 0-59 months, resides in the study area for the past 12 months	India	Cross-sectional study	Quantitative	Vaccine hesitancy	Family type; education of mother
	Lae (2018)[50]	Caregivers in slums	Myanmar	Cross-sectional study	Qualitative	Utilisation of immunisation services	Age of child; income; migration; antenatal visit; receiving additional vaccines before; having immunisation card.
	Schultz (2017)[126]	Parents with children <5 years old in slums	Kenya	Prospective study	Quantitative	Timeliness of vaccination	Close to the clinic; birth in December
	Crocker-Buque (2017)[21]	People living in a low-income urban area or slum in a low-middle income countries	Multiple nations	Systematic review	-	Immunisation coverage	*Socioeconomic and demographic characteristics:* socioeconomic status; wealth; parents' literacy; mother's education; employment; residential status; place of residence; place of delivery; household visit by health workers; premature birth; malnourishment; inadequate housing; poor

						prenatal care; ethnicity; age; maternal age; birth order; sex of child; number of children<br><br>*Migration status:*<br>migration; recent migration<br><br>*Information, beliefs and behaviour:*<br>unaware of the need for vaccines; unaware of clinic location or timing; maternal knowledge of immunisation; lack of access to information; parents being too busy; return to home village; difficulty in accessing services; fear of side effects; attitude of health workers; concerns over cost; being suspicious of free services<br><br>*Health services:*<br>distance from health centre; timing of services; fear of costs; risk of lost income; lack of local knowledge; patients' satisfaction; provision of accurate information; accessing pre-natal care
Shrestha (2016)[82]	Slum households with children aged 12–23 months.	Nepal	Case-control study	Quantitative	Incompletion of immunisation	Home delivery; type of residence; knowledge about healthcare services of primary care-taker; perception towards healthcare services, conflicting priorities, side effect
Devasenapathy (2016)[57]	Slum household with children aged between 12 and 42 months	India	Cross-sectional study	Quantitative	Childhood complete immunisation	Sex; mother's literacy; place of birth; place of childbirth; religion; socioeconomic position; birth certificate

Maternal	Sendo (2021)[92]	Female slum residents	Ethiopia	Cross-sectional study	Qualitative	Delivery in healthcare facilities	Provision of quality, respectful and dignified midwifery care; lack of awareness about facility delivery.
	Kardalkar (2020)[135]	Female delivered within three months in slums	India	Cross-sectional study	Quantitative	Utilization of antenatal care	Literacy; Gravida; occupation
	Sendo (2020)[91]	Women of reproductive age in slums	Ethiopia	Cross-sectional study	Qualitative	Delivery in health facilities	Perceived benefits of home delivery; knowledge deficit about health facility-based delivery; poor access to healthcare facilities; inadequate resources
	Sharma (2020)[127]	Women delivered a baby within one year in slums	India	Cross-sectional study	Quantitative	Utilization of maternal healthcare services	Education; employment of mother; category and type of family; distance and time to reach health facility;
	Yadav (2020)[42]	Married women in slums	India	Cross-sectional study	Quantitative	Unmet need for family planning services	Age; educational status; duration of marriage; number of pregnancies; knowledge of contraceptive methods; opposition to contraceptive use; contact with a midwife
	Razzaque (2020)[66]	Slum residents	Bangladeshi	Cross-sectional study	Quantitative	Healthcare utilisation	Recent migration; wealth; education; employment
	Getachew (2020)[113]	Slum households	Ethiopia	Cross-sectional study	Quantitative	Delivery in healthcare facilities	Perceived as not customary to deliver at health facility; not necessary; unavailability of female birth attendants; perceived quality of services; cost
	Shrestha (2019)[61]	Mothers with infant residing in slums	Nepal	Cross-sectional study	Quantitative	Utilisation of antenatal and delivery services	Educational status of respondents and their husbands; number of pregnancy
						Institutional delivery	Educational status; occupation of husband; number of pregnancy

					Postnatal visit	Occupation of husband
					Utilisation of family planning services	Occupation of husband
					Tetanus Toxoid immunisation	Educational status of respondents; economic status; knowledge about healthcare services; educational status of husband; number of pregnancies
Atusiimire (2019)[98]	Mothers delivered in the past one year in slums	Uganda	Cross-sectional study	Quantitative	Facility based–deliveries	Exposure to media concerning facility delivery; frequency of ANC; timing of 1st ANC
Upadhyai (2019)[39]	Recently delivered mothers residing in slums	India	Cross-sectional study	Quantitative	Healthcare utilisation	Age; education of mother and father; socioeconomic class; antenatal check-ups; institutional delivery services; family type; caesarean delivery; complication or perceived health problem
Angeles* (2019)[47]	Slum and non-slum residents	Bangladesh	Prospective study	Quantitative	Use of modern contraceptive methods	Parity, mother's age; mother's education, socioeconomic status, interaction (slum × time period)
					Delivery by skilled birth attendant	Residing in slums, parity, mother's age, mother's education, length of stay in current city of residence, socioeconomic status, number of available community health worker, distance from health facility, interaction (slum x time period)
Kusuma (2018)[80]	Recent migrant and settled mothers with a child up to the age of 1 year in slums	India	Cross-sectional study	Quantitative	Birth in health facility	Listening to radio; number of ANC visits; plan for hospital birth; plan for transport; some danger sign; knowledge of danger sign

Sharma (2018)[138]	Women living in urban slums and delivered a baby within 1 year	India	Cross-sectional study	Quantitative	Utilisation of maternal care services	Mode of delivery; hospital stay after delivery
Islam* (2018) [107]	Ever-married women aged 15-49 years living in slum and non-slum	Bangladesh	Cross-sectional study	Quantitative	ANC visits	Education; wealth index of the household
Geddam (2017)[67]	Rural to urban internal migrant mothers with a child of less than 2 years of age	India	Cross-sectional study	Quantitative	Utilisation of maternal health services	Education of the mother; family size; occupation of mother
					Delivery in institution	Educational status of mother; number of ANC visit; adequacy of ANC; migration status
Kaba (2017)[94]	Stakeholders including city administrators, community members, healthcare providers	Ethiopia	Cross-sectional study	Qualitative	Maternal health service utilisation	Lack of awareness and lack of perceived needs about available services; fear of stigma; competing priorities, social connectedness; perceived lack of respectful service providers; socio-cultural factors including socially sanctioned expectations
Verma (2017)[75]	Pregnant women and infants in slums	India	Case-control study	Mixed-method	Antenatal care registration/immunisation	Knowledge of healthcare services; perceived need for healthcare services; family support; fear; negative experience with previous vaccination
Sharma (2016)[51]	Married women in slums	Nepal	Cross-sectional study	Quantitative	Antenatal healthcare utilisation	Age; husband education; spouse occupation; family income; type of family; planned pregnancy; death of children
Jolly (2016)[108]	Married women with a pregnancy outcome in the previous year in slums	Bangladesh	Cross-sectional study	Quantitative	Antenatal care; birth assisted by medically trained provider; postnatal care; treatment seeking for	Education; wealth

						delivery complications	
						Use of modern family planning	Wealth
	Tebekaw (2016)[117]	Women in slums	Ethiopia	Cross-sectional study	Quantitative	Antenatal care services	Education; private/public hospital
	Sadhna (2016)[109]	Married women in slums	India	Cross-sectional study	Quantitative	Utilisation of maternal health services	Education; Caste; wealth; distance to preferred health facility
	Neyaz (2016)[62]	Married women in slums	India	Cross-sectional study	Quantitative	Delivery in hospitals	Received ANC; number of ANC visits; education; birth order; living index
	Rahman (2016)[105]	Married women in rural and slum area	India	Cross-sectional study	Quantitative	Intrauterine contraceptive device utilisation	Income; occupation
	Sheehy (2016)[103]	Informant and women in slums	Myanmar	Cross-sectional study	Qualitative	Giving birth in hospital	Financial constraints; lack of transportation; sociocultural and financial considerations
Contraceptive	Renzaho (2017)[48]	Slum residents aged 13-24	Uganda	Cross-sectional study	Quantitative	Access to contraceptive services and family planning	Age; disability
	Abd El Fatah (2019)[136]	Married women aged 15–49 years in slums	Egypt	Cross-sectional study	Quantitative	Contraceptive use	Number of male children
Health insurance	Iyalomhe (2021)[41]	Slum residents	Nigeria	Cross-sectional study	Quantitative	Healthcare insurance coverage	Age; sex; marriage; income; religion; education
	Mendhe (2021)[40]	Female slum residens	India	Cross-sectional study	Quantitative	Healthcare insurance coverage	Socioeconomic status;
						Out of pocket expenditure	Age; government/ private hospital
	Otieno (2019)[84]	Slum residents	Kenya	Cross-sectional study	Quantitative	Enrolment in a health insurance programme	Employment; source of primary care; satisfaction with cost of care; satisfaction with procedure of care; perceived health status
	Kusuma (2018)[69]	Slum residents	India	Cross-	Quantitative	Health insurance	Residential background (old

				sectional study		possession	slums than new); migration period; possession of ration card; household size; occupation of household head
	Gupta (2017)[95]	Slum households having health insurance cards	India	Cross-sectional study	Mixed-method	Utilisation of healthcare insurance	Awareness of the empanelled hospitals; experiences of friends and relatives at national health insurance empanelled hospitals; hospitals refused to accept health insurance cards
Expenditure	Sahu (2017)[63]	Women delivered within a period of 6 weeks in slums	India	Cross-sectional study	Quantitative	Out-of-pocket expenditure for maternal and neonatal health services	Gravidity; type of delivery; place of delivery; morbidity
	Mishra (2017)[59]	Slum households with a child aged 0–14 years and who had migrated within the last 12 years	India	Cross-sectional study	Quantitative	Out-of-pocket expenditure	Child's gender; mother's education; type of illness

[*]Factors reported in the study were associated with participants covering both slum and non-slum residents. ANC: antenatal care; CVD: cardiovascular disease; HIV: human immunodeficiency virus; N/A: not applicable; NGO: non-governmental organization; TB: tuberculosis.
