# Supplementary material for: Factors associated with accessing and utilisation of healthcare and provision of health services for residents of slums in low and middle-income countries: a scoping review of recent literature
Source: BMJ Open. 2022 May 20;12(5):e055415. doi: 10.1136/bmjopen-2021-055415 (PMC9125718; doi:10.1136/bmjopen-2021-055415)
Supplement: Author's manuscript [file bmjopen-2021-055415.draft_revisions.pdf]

BMJ Open

BMJ Open is committed to open peer review. As part of this commitment we make the peer review history of every article we publish publicly available.

When an article is published we post the peer reviewers' comments and the authors' responses online. We also post the versions of the paper that were used during peer review. These are the versions that the peer review comments apply to.

The versions of the paper that follow are the versions that were submitted during the peer review process. They are not the versions of record or the final published versions. They should not be cited or distributed as the published version of this manuscript.

BMJ Open is an open access journal and the full, final, typeset and author-corrected version of record of the manuscript is available on our site with no access controls, subscription charges or pay-per-view fees (http://bmjopen.bmj.com).

If you have any questions on BMJ Open's open peer review process please email info.bmjopen@bmj.com

BMJ Open

Factors associated with accessing health care and provision of health services for residents of slums in low and middle income countries: a scoping review of recent literature

Journal:	*BMJ Open*
Manuscript ID	bmjopen-2021-055415
Article Type:	Original research
Date Submitted by the Author:	12-Jul-2021
Complete List of Authors:	Park, Ji-Eun; University of Warwick, Warwick Medical School; Korea Institute of Oriental Medicine<br>Kibe, Peter; African Population and Health Research Center, Health and Systems for Health<br>Yeboah, Godwin; University of Warwick, Institute for Global Sustainable Development<br>Oyebode, Oyinlola; University of Warwick Warwick Medical School,<br>Harris, Bronwyn; University of Warwick, NIHR Global Health Research Unit on Improving Health in Slums<br>Ajisola, Motunrayo ; University of Ibadan, Sociology<br>Griffiths, Frances; University of Warwick Warwick Medical School, ; University of the Witwatersrand, Centre for Health Policy<br>Aujla, Navneet; Newcastle University, Population Health Sciences Institute; University of Warwick, Division of Health Sciences<br>Gill, Paramjit ; University of Warwick<br>Lilford, RJ; University of Birmingham, Institute of Applied Health Research<br>Chen, Yen-Fu; University of Warwick, Division of Health Sciences
Keywords:	HEALTH SERVICES ADMINISTRATION & MANAGEMENT, International health services < HEALTH SERVICES ADMINISTRATION & MANAGEMENT, PUBLIC HEALTH

SCHOLARONE™
Manuscripts

I, the Submitting Author has the right to grant and does grant on behalf of all authors of the Work (as defined in the below author licence), an exclusive licence and/or a non-exclusive licence for contributions from authors who are: i) UK Crown employees; ii) where BMJ has agreed a CC-BY licence shall apply, and/or iii) in accordance with the terms applicable for US Federal Government officers or employees acting as part of their official duties; on a worldwide, perpetual, irrevocable, royalty-free basis to BMJ Publishing Group Ltd ("BMJ") its licensees and where the relevant Journal is co-owned by BMJ to the co-owners of the Journal, to publish the Work in this journal and any other BMJ products and to exploit all rights, as set out in our licence.

The Submitting Author accepts and understands that any supply made under these terms is made by BMJ to the Submitting Author unless you are acting as an employee on behalf of your employer or a postgraduate student of an affiliated institution which is paying any applicable article publishing charge ("APC") for Open Access articles. Where the Submitting Author wishes to make the Work available on an Open Access basis (and intends to pay the relevant APC), the terms of reuse of such Open Access shall be governed by a Creative Commons licence – details of these licences and which Creative Commons licence will apply to this Work are set out in our licence referred to above.

Other than as permitted in any relevant BMJ Author's Self Archiving Policies, I confirm this Work has not been accepted for publication elsewhere, is not being considered for publication elsewhere and does not duplicate material already published. I confirm all authors consent to publication of this Work and authorise the granting of this licence.

For peer review only

Factors associated with accessing health care and provision of health services for residents of slums in low and middle income countries: a scoping review of recent literature

Ji-Eun Park,[1,2] Peter Kibe,[3] Godwin Yeboah,[4] Oyinlola Oyebode,[1] Bronwyn Harris,[1] Motunrayo Ajisola,[5] Frances Griffiths,[1,6] Navneet Aujla,[1,7] Paramjit Gill,[1] Richard Lilford,[8] Yen-Fu Chen,[1]* on behalf of the Improving Health in Slums Collaborative

Affiliations:

[1] Division of Health Sciences, Warwick Medical School, University of Warwick, Coventry, United Kingdom

[2] Future Medicine Division, Korea Institute of Oriental Medicine, Daejeon, Republic of Korea

[3] African Population and Health Research Center, Nairobi, Kenya

[4] Institute for Global Sustainable Development, University of Warwick, Coventry, United Kingdom

[5] College of Medicine, University of Ibadan, Ibadan, Nigeria

[6] Centre for Health Policy, School of Public Health, University of the Witwatersrand, Johannesburg, South Africa

[7] Population Health Sciences Institute, Newcastle University, Newcastle, United Kingdom

[8] Institute of Applied Health Research, University of Birmingham, Birmingham, United Kingdom

*Corresponding author: Dr Yen-Fu Chen, Division of Health Sciences, Warwick Medical School, University of Warwick, Coventry, CV4 7AL, UK.

Email: Y-F.Chen@warwick.ac.uk

Word count: 4,943

For peer review only

For peer review only - http://bmjopen.bmj.com/site/about/guidelines.xhtml

ABSTRACT

Objective: To identify factors associated with healthcare-seeking behaviour, healthcare utilisation and provision of health services in slums.

Design: A scoping review incorporating a conceptual framework for configuring factors associated with access and provision of healthcare in slums.

Data sources: MEDLINE, Embase, CINAHL, Web of Science and the Cochrane Library were searched in April 2020 using slum related terms.

Eligibility criteria: Empirical studies of all designs reporting relevant factors in slums in low and middle income countries.

Data extraction and synthesis: Studies were categorised and data were charted according a preliminary conceptual framework refined by emerging findings. Results were tabulated and narratively summarised.

Results: Of the14,041 records retrieved from all years, 3895 records dated between 2016-2020 were screened by two independent reviewers and 92 studies were included. The majority (53 studies, 58%) were conducted in Asia, predominantly in India. Eighty-five studies examined healthcare access from slum residents' perspective while only eight studies explored provision of health services from providers/planners' perspective (one study included both). A multitude of factors are associated with accessing and providing healthcare in slums, including recent migration to slums; knowledge, perception and past experience of illness, healthcare needs and health services; financial constraint and competing priorities between health and making a living; lacking social support; unfavourable physical environment and locality; sociocultural expectations and stigma; lack of official recognition; and problems in existing health system.

Conclusion: The scoping review identified a significant body of recent literature reporting

For peer review only - http://bmjopen.bmj.com/site/about/guidelines.xhtml

factors associated with access and provision of healthcare services in slums. We classified the diverse factors under seven broad categories. The findings can inform a holistic approach to improving health services in slums by tackling barriers at different levels, taking into account local context and geospatial features of individual slums.

Systematic review registration: Open Science Framework (OSF, https://osf.io/694t2)

Keywords: slum, informal settlement, scoping review, healthcare-seeking behaviour, healthcare utilisation, health service delivery

For peer review only

Strengths and limitations of this study

- We conducted literature search in multiple databases using generic terms related to slums to ensure that a wide range of relevant studies was captured.
- A conceptual framework explaining factors associated with healthcare seeking behaviour and healthcare utilisation of slum residents as well as provision of healthcare in slums was developed and used to categorise identified studies and factors.
- We examined barriers and facilitators of healthcare access and service provision from the perspectives of both demand side (slum residents) and supply side (healthcare providers and service planners).
- Only studies published between 2016 and 2020 in English language were included, and methodological quality of each included study was not examined because of time constraint.
- We did not explore the complex relationships and interactions between various factors at different slum locations, but our mapping of these factors to the conceptual framework should facilitate further in-depth analyses.

For peer review only - http://bmjopen.bmj.com/site/about/guidelines.xhtml

INTRODUCTION

Rapid urbanisation has resulted in a growing number of residents in slums[1] who face ongoing problems such as unemployment, poor sanitation, lack of transport, high level of crime, and haphazard development.[2] In 2018, over one billion people were living in slum-like conditions, and Central, South and South-East Asia and Sub-Saharan Africa accounted for 80% of them.[1] Even though various definitions of slums exist, there is no universally agreed definition of what constitutes 'a slum', and the term itself is widely debated and contested.[3 4] For the purpose of this scoping review, we refer to slums as densely populated areas characterised by lack of basic services, substandard housing, overcrowding, unhealthy living condition, insecure tenure and poverty,[4 5] taking into account the crucial concepts of place and space that are important in shaping health outcomes and community access to health services in these urban settings.[4]

Previous studies have reported various risk factors affecting health of slum residents such as physical environment,[6] sanitation,[7] social capital[8 9] and water governance,[10] and have observed in some cases that slum residents have worse health status compared to non-slum urban and/or rural residents. For example, Ezeh et al. found that children living in slums had higher mortality than rural and non-slum urban populations.[3] Poorer height-for-age for children[11] and higher prevalence of childhood illnesses and malnutrition[12] have also been observed in slums compared to non-slum urban and rural settings. In addition, slum residents are susceptible to unhealthy behaviours.[13 14] Living in slums has been found to be associated with low physical activity,[13] poor diet,[14] and poor knowledge about the cause and preventability of diseases.[15]

Despite the unfavourable health status and environment, and consequently the potential high level of healthcare needs, previous studies showed that slum residents were less likely to seek and use healthcare services than their non-slum counterparts in the cities.[16 17] Slum residents have been found to have lower rates of healthcare utilisation in antenatal services[16] and services

For peer review only

for non-communicable diseases[17] compared to residents of urban 'formal' settings. One study in Iran showed that only about half of slum households that required outpatient services could use them.[18] Another study in Haiti also reported that one third of slum households were not able to access medical care for their children when it was needed in the past year.[19]

While the health status and needs of slum residents have been described in previous reviews,[3] [20] factors associated with healthcare seeking behaviour and healthcare utilisation of slum residents and factors related to the provision of health services in slums have not been systematically examined (with the exception of immunisation services).[21] This scoping review aims to fill in these evidence gaps and inform efforts to improve healthcare delivery to people in slums.

METHODS

This scoping review was performed according to current best practice guidance.[22] The broad question of interest was: "*What factors are associated with slum residents' care seeking behaviour and access to health care and/or the provision of health services in slum settings in low and middle income countries (LMICs)*?" The protocol for this review was registered in Open Science Framework (OSF).[23]

Literature search and study selection

A comprehensive search of five databases: MEDLINE, Embase, CINAHL, Web of Science and the Cochrane Library was conducted in April 2020. Searches were limited to English language. Key terms related to slums were used: slum or slums or ghetto or ghettos or informal settlement$ or shantytown$ or shanty town$ or favela$. We did not include terms related to

For peer review only - http://bmjopen.bmj.com/site/about/guidelines.xhtml

other concepts in order to maximise the sensitivity of our searches.

Records retrieved from databases (after duplicates were removed) were initially screened by one reviewer (JEP) and those which did not meet the inclusion criteria were disregarded. After that, a second reviewer (PK, GY, OO) examined the remaining records independently based on titles and abstracts. When the decisions of two reviewers differed, the discrepancy was resolved based on full-texts and/or by discussion with a third reviewer (YFC) or the broader review team. This study screening process started from records of the most recent years (i.e. in the past three years) and then proceeded to prior years. Due to the larger than expected volume of the literature, we eventually screened records between 2016 to 2020 and did not cover earlier records in order to synthesise and present the findings from latest evidence in a timely fashion to inform the wider project hosting this review.[24 25]

Inclusion and exclusion criteria

A study was included when it: (1) described factors related to slum residents' healthcare seeking behaviour or access to health care or the provision of health services in slums; and (2) was conducted in relation to slums in LMICs. Only articles written in English were included. A study was excluded when it was a commentary, opinion, or narrative review; described slum residents' access to health services or the provision of health services without exploring the associated factors; investigated informal care at home; or included mixed slum and non-slum populations without separately reporting data for slum residents or investigating residency in slums as a factor for healthcare access.

We included both primary studies and systematic reviews that examine data collected empirically and that derive their findings based on the data. Both quantitative and qualitative studies (and by extension, mixed methods studies) were considered. Even though slums have

For peer review only - http://bmjopen.bmj.com/site/about/guidelines.xhtml

existed in both high-income countries and LMICs, the context may be quite different between these countries. For example, while all slums are vulnerable to natural disasters such as tropical cyclones, the impact of these could be far more severe in slums of LMICs due to the different socioeconomic contexts.[26] In this review, we focused on settings in LMICs and excluded studies conducted in high-income countries.

Study coding and data extraction/charting

Eligible studies were coded and data-extracted/charted according to a pre-specified, preliminary framework shown in Figure 1 below. The preliminary framework was developed by the review authors based on existing conceptual models related to healthcare access and service delivery[27-30] and was modified during the scoping review process to accommodate new factors/themes identified from the literature.

Based on the conceptual framework, each eligible study was coded as being associated with one or more of the three phenomena of interest, namely slum residents' healthcare-seeking behaviours (which covered both perception of needs/desire for care and actual health care seeking), health care utilisation (which covered healthcare reaching[29] and utilisation) and provision of health services (which covered various arrangements related to service delivery) in slum settings (Figure 1).

In addition, to facilitate the organisation of complex evidence in this review, diverse factors were initially classified into six different categories according to the preliminary framework shown in Figure 1. The framework was refined to reflect emerging themes during the coding and data extraction/charting process. The final framework is shown in Figure 2 and contains seven categories:

(1) Personal and biological factors: these relate to personal characteristics of slum residents,

For peer review only

For peer review only - http://bmjopen.bmj.com/site/about/guidelines.xhtml

including age, sex, ethnicity and the nature and severity of health conditions.

(2) Cognitive and experiential factors: these relate to personal awareness, knowledge, perception, attitude, belief and experience etc. formed through cognitive process based on upbringing and past events.

(3) Socioeconomic factors: these include income and wealth, economic hardship/poverty and economic opportunities, marital status, education, crime, social capital (such as bonding, trust and reciprocity between close relatives, neighbours and community members),[31] use of technologies for social and economic purposes, commercial and charitable organisations and activities.

(4) Physical environment: this covers natural environment such as proximity to a health facility, built environment and infrastructure such as water supply, transport and mobile/internet networks, as well as weather conditions and environmental pollutions.

(5) Cultural and religious factors: these include cultural and religious beliefs and activities, and local and national customs.

(6) Legal, political and policy factors: these include government policies and issues related to legal, justice and political systems.

(7) Health system factors: these relate to historical and current organisation and provision of health care that may impact upon provision and delivery of health services in individual slum communities and the services experienced by slum residents.

Data on study population, study design, country in which the study was conducted, methodology, and associated factors were extracted using a data-charting spreadsheet which was developed and continuously updated as the review progressed by two of the reviewers (JEP and YFC). Whether a study was conducted exclusively within slums and whether a comparison was made between slum and non-slum urban or rural residents were also noted.

For peer review only - http://bmjopen.bmj.com/site/about/guidelines.xhtml

Coding of phenomena and factors and data-charting were conducted by one reviewer (JEP) and checked by a second reviewer (PK, GY, OO, YFC). Disagreements were discussed between reviewers until consensus was reached.

Patient and public involvement

Given the focus of this scoping review on published literature, we did not directly involve residents and service providers/planners from slum settings. Nevertheless, our wider project has a work package that specifically engages with slum residents and service providers and planners,[24] and early plans and findings of this review were shared with the wider project team who provided comments based on their experiences of community engagement.

RESULTS

The reporting of this review follows the PRISMA Extension for Scoping Reviews (PRISMA-ScR).[32] Using the search strategy described earlier, a total of 21,248 records were retrieved, with 14,039 records (Medline 3340, Cochrane 129, Embase 1626, CINAHL 323, Web of Science 8621) remaining after excluding duplicates. Two additional articles[18 33] were identified from references of the included studies. As described earlier, screening was limited to the 3895 records published from 2016 onwards.

A total of 92 articles were included in this scoping review. (Figure 3) Twenty-nine studies reported factors associated with healthcare-seeking behaviour of slum residents, 58 studies reported factors related to healthcare service utilisation, and eight articles reported the factors related to provision of healthcare services in slums (three studies reported factors related to more than one phenomenon of interest). Fifty-nine of the 92 studies were quantitative studies,

For peer review only - http://bmjopen.bmj.com/site/about/guidelines.xhtml

19 studies were qualitative studies, and 12 studies were undertaken using mixed-methods. The remaining two studies were systematic reviews. A total of 33 (36%) studies were conducted in India, followed by Kenya (11 studies, 12%). (Table 1)

For peer review only

For peer review only - http://bmjopen.bmj.com/site/about/guidelines.xhtml

Table 1. Characteristics of included studies.

Category	Subcategory		Number of studies (%)	
Publication year	2016		22	(24)
	2017		17	(18)
	2018		23	(25)
	2019		22	(24)
	2020		8	(9)
Analysis method	Quantitative		59	(64)
	Qualitative		19	(21)
	Mixed-methods		12	(13)
	Narrative synthesis		2	(2)
Study location	Asia	India	33	(36)
		Bangladesh	9	(10)
		Nepal	4	(4)
		Myanmar	2	(2)
		Pakistan	2	(2)
		Iran	2	(2)
		Sri Lanka	1	(1)
	South America	Brazil	5	(5)
		Peru	2	(2)
	Africa	Kenya	11	(12)
		Ethiopia	5	(5)
		Malawi	4	(4)
		South Africa	2	(2)
		Uganda	2	(2)
		Sierra Leone	1	(1)
		Nigeria	1	(1)
		Egypt	1	(1)
		Zambia	1	(1)
		Namibia	1	(1)
		Ghana	1	(1)
	North America	Haiti	1	(1)
	Multiple nations		1	(1)
Healthcare services in slums*	Healthcare-seeking behaviour		29	
	Healthcare service utilisation		58	
	Provision of healthcare services		8	
Total			92	(100)

* One study reported factors related to both healthcare-seeking and healthcare utilisation and two studies reported factors related to both healthcare utilisation and provision of healthcare services

For peer review only - http://bmjopen.bmj.com/site/about/guidelines.xhtml

Participants, country, study design, methodology, observed phenomena and outcomes, and factors of interests for each study are described in supplemental tables 1-3. Supplemental table 1 shows 29 studies reporting factors associated with general healthcare seeking behaviours; healthcare seeking for children or women; slum residents' preference for healthcare providers; and healthcare seeking related to HIV testing. Supplemental table 2 presents various factors reported in 58 studies related to general healthcare utilisation as well as use of specific services such as childhood immunisation, maternal healthcare, and possession of health insurance. In Supplemental table 3, eight studies reporting factors related to the provision of health services in slums are summarised. Key findings are described below.

For peer review only

For peer review only - http://bmjopen.bmj.com/site/about/guidelines.xhtml

Demand side: Factors associated with healthcare-seeking behaviour and healthcare utilisation of slum residents

We found 86 articles which identified many different factors affecting healthcare-seeking behaviour and utilisation. These factors are often inter-related and exert their influence at different levels (e.g. from personal, family to community level) in different circumstances. We classified various factors into seven categories (Figure 2). Factors particularly relevant to slum settings and other commonly identified factors within each category are highlighted below.

Personal and biological factors: major life events such as recent migration[21 34-37] and relocation[38] into slums tend to be associated with lower healthcare seeking and utilisation. Recency of migration to slums was also related to uptake of Rashtriya Swasthya Bima Yojana (RSBY), a national health insurance programme run by the Indian government.[39] Other common factors associated with healthcare seeking and utilisation included intrinsic factors such as age,[21 37 38 40-48] sex,[18 21 42 45 49-51] and ethnicity,[21 47] familial factors such as birth order of the sick child,[21 41 47 52-54] or number of male children in the family;[55] as well as personal health and the specific features of the health condition such as fever, tachypnoea, chest in drawing, persistent vomiting,[44 45 56] type of illness,[49] disability[46] and morbidity.[21 42 57] One study showed that tobacco habits and family history of cancer were associated with attending cancer screening test.[43]

Cognitive and experiential factors: these factors were not included in our initial conceptual framework but rather emerged inductively from our data. Consequently, their identification led us to revise the conceptual framework for this scoping review. A wide range of factors formed through cognitive processes and influenced by individual's upbringing, past experience and surrounding environment were reported to be associated with both healthcare-seeking and healthcare utilisation of slum residents. Perception,[36 40 44 48 58-64] knowledge[44 47 65-69] and

For peer review only - http://bmjopen.bmj.com/site/about/guidelines.xhtml

experience of symptoms and illnesses[48] were commonly found to influence healthcare seeking and utilisation. Denial, complacency, fear of death were reported as reasons that participants did not get HIV test.[59] Ability in managing the condition at home[44 70] such as home remedies[58] and perceived need for accessing healthcare services [21 33 36 45 62 63 71 72] also affected healthcare-seeking and healthcare utilisation among slum residents. In addition, perception,[21 63 70 72-79] knowledge,[21 36 38 53 62 72 73 80 81] and experience of healthcare services[35 37 40 43 52 54 62 67 82-84] including fear and distrust of healthcare services,[21 33 36 59 62 63 66 76 85-87] and preference related to care provider's gender[75 88] were frequently cited factors. Provider shopping associated with distrust of healthcare providers and denial of diagnosis delayed first care seeking and treatment initiation of pulmonary tuberculosis patients in India.[58] Perception or experience of healthcare services also affected uptake or renewal of health insurance.[78 81]

Socioeconomic factors: income and wealth[34 36-38 45 48 50 53 68 89-93] including financial constraint [19 21 33 61 64 66 70 71 74 77 83 87 94-96] featured prominently. The socioeconomic challenges faced by slum residents also manifested as competing priorities [61 72 97] and lack of time[21 87 98] for healthcare-seeking and utilisation, because they did not want to or could not afford to miss work and lose income.[21 58] These were exacerbated by lack of social support,[62 64 72 75 86 99] which was linked to further barriers such as not being able to seek healthcare due to security at night.[86] Other socioeconomic factors reported included social class,[21 40 41 47 51 54 93 94 100 101] marital status,[18 43] family composition,[21 35 38-40 48 55 102] education,[21 34-36 38 40 41 43-45 47-54 68 69 89 92 93 101-103] occupation,[35 38 39 43 48 50 53 68 69 91] and employment.[21 34 78 104]

Physical environment: Slum residents considered proximity of healthcare facilities,[21 33 41 64 68 70 73 79 93 105-107] transport such as travel assistance,[64] lack of transportation[33 69 94], traffic congestion,[108] and environment of residence area when they sought and used healthcare services.

For peer review only - http://bmjopen.bmj.com/site/about/guidelines.xhtml

Cultural and religious factors: these included religion;[43 51 52 63 101] sociocultural influence[72 88] such as exposure to media[67 84]; stigma associated with unplanned/extramarital pregnancy[66 72] postpartum depression[109] and other illnesses such as contagious skin disease, barrenness and female sexually-related problems;[88] and use of traditional/home medicine.[63 83 86] Women in slums could not go to hospital because they had difficulties in disclosing the symptoms, postponed their health issues because of their responsibilities at home, and engaged in self-treatment practices such as home remedies recommended by grandmother and friends because because of socio-cultural influences toward healthcare-seeking behaviour.[65] Women in Ethiopia reported not returning to postnatal care due to religious and cultural expectation for mother and baby to stay home for 80 days after birth.[72] One Indian survey showed that some women could not seek healthcare services during labour since their husband or family did not allow that.[33]

Legal and political factors: type of slums (in terms of official recognition and availability of basic facilities) and possession of a ration card were found to be associated with uptake of the Indian RSBY a national health insurance programme.[39] One study reported that slum residents could not seek healthcare facilities for abortion because of the perceived illegality of abortion.[66]

Health systems: slum residents were also influenced by many factors related to health systems when they sought healthcare. These included accessibility associated with the location[21 88] and timing of services;[21 70 83] quality of healthcare services[33 35 71 73 79 83 88] such as delay in advising patients to go for related tests or referral,[58] likelihood of receiving appropriate examination,[70 95] and adverse events.[63] Slum residents considered service organisation including medical turnover,[110] availability of supplies/healthcare workers,[41 70 71 95] attitude of healthcare providers,[83] type of healthcare facilities,[33 73 103 111] and waiting time.[60 70 73 74 83 95 112] In an Ethiopian study, some participants reported unavailability of female birth attendants as a

For peer review only - http://bmjopen.bmj.com/site/about/guidelines.xhtml

reason for not delivering at healthcare facilities.[71] (Table 2)

For peer review only

For peer review only - http://bmjopen.bmj.com/site/about/guidelines.xhtml

Table 2. Factors associated with healthcare-seeking behaviour and healthcare utilisation in slums.

Factors	Healthcare seeking	Healthcare utilization
Personal and biological factors		
Age	Age[43-45]	Age[21 37 38 40-42 46-48]; age of household head[18]
Gender	Sex[45 49 50]	Sex[18 21 42 49 51]
Ethnicity		Ethnicity[21 47]
Migration		Recent migration[21 34-37]; relocation[38]; return to home village[21]
Biological	Symptoms such as fever, tachypnea, chest in drawing, persistent vomiting[44 45 56]	Type of illness[49]; disability[46]; morbidity[21 42 57]
Other personal	Tobacco habits [43]; family history of cancer [43]; family history of cancer [43]	Birth order of sick child [21 47 52-54]; parity [41 57]
Cognitive and experiential factors		
Knowledge/experience of symptoms and illnesses	Perception of symptoms[58] or illness[44 60 64]; knowledge of symptom/disease[44 65 66]; denial and complacency[59]	Experience of child death[48]; planned pregnancy[48]; perceived health status[48 78] and health problem[36 40 61-63]; knowledge of symptom[69] or disease[47 67 68]
Ability/experience in handling health related conditions and perceived needs for accessing health services	Awareness of the need for healthcare services[33 45]; home remedies[58] or management of childhood illness[44 70]	Perceived needs for healthcare services[21 36 62 63 71 72]
Perception/knowledge/experience/preference of health services	Fear of mistreatment[59 87] and doubts about medical care[33 66 86]; gender-induced affordability[88]; provider shopping[58]; history of cancer screening[43]	Perception of healthcare services[21 63 73 75-78] and providers[21 70 72 74 79]; knowledge of health services[21 36 38 53 62 72 73 80] or facilities[21 81]; experience of healthcare services[37 62 82 83]; experiences of friends and relatives at healthcare facilities[81]; previous use of related healthcare services[35 37 40 52 54 67 84]; misunderstanding or fear[21 36 62 63 76 85]; gender healthcare worker preference[75]
Socioeconomic factors		
Socioeconomic status	Social class[100]	Socioeconomic status[21 40 41 47 51 54 94]; Caste[93 101]; residential background[21 39 41]; possession of ration card[39]
Marital status	Marital status[43]	Marital status[18]
Family composition & Living arrangement		Family type[40 48 102]; family size[35 39]; number of children in household[21 38]; number of male children[55]; type of residence[47]; housing condition[21]
Education	Education[43-45 50]	Education[34-36 38 40 47 49 51 53 54 68 69 89 92 93 101-103]; husband education[48]; mother's education and literacy[21 41 51 52]
Income and wealth	Income[45 90]; wealth[50]; inability to	Income[36 37 48 68 91]; wealth[21 34 38 53 89 92]

For peer review only - http://bmjopen.bmj.com/site/about/guidelines.xhtml

	afford care [19 33 64 66 70 77 87 95 96]	[93]; financial constraint[21 61 71 74 83 94 113]
Occupation	Occupation[43 50]	Employment[21 34 78 104]; occupation[35 38 68 69 91]; occupation of spouse[48 53] or household[39]
Social support	Difficulty in reaching services (security risk at night)[86]; accompanying person[64]	Family support[62 75]; social connectedness[72]; socioeconomic support[99]
Competing priorities/lack of time	Competing priorities (ability to work and income)[97]; not want to miss work[58]; lack of time[87 98]	Competing priorities[61 72]; risk of lost income[21]; parents being too busy[21]
Physical environment		
Distance from health facility	Proximity of healthcare facilities[33 64 70 105]; geographical distance of formal healthcare[88]	Distance from health facility[21 41 68 73 79 93 106 107]
Transport	Travel assistance[64]; no transportation[33]	Lack of transportation[69 72 94]; variability in traffic congestion[108]
Environment of residence area	Difficulty in reaching services (darkness at night)[86]	Residential background[21 39]
Cultural and religious factors		
Religion	Religion[43]	Religion[51 52 63 101]
Sociocultural influence	Stigma[66 88 109]; mother tongue[43]; difficulties in disclosing the symptoms, neglecting behaviours, and socio-cultural influences[65]; cultural competency of care[88]; easy communication[88]; living with the burden of cultural expectations[88]; no permission to seek care from family[33]	Exposure to media[67 84]; cultural expectation for women after birth and fear of stigma for pregnancy out of wedlock[72]
Tradition	Traditional medicine[86]	Traditional remedies[63]; home remedies[83]
Legal, political and policy factors		
Legal issues	Perceived illegality of abortion[66]	Type of slums and possession of a ration card[67]
Health system factors		
Accessibility	Ease of access[88]; late facility opening times[70]	Limited access to the services due to location[72]; timing of services[21 83]; healthcare insurance[18 69]; household visit by health workers[21]
Quality and safety of services	Quality of treatment and expected outcome of therapies[33 88]; delay in advising related tests[58]; referral[58]; optimal examination[70 95]; provider shopping[58]	Quality of service[35 71 73 79 83]; adverse events[63]
Service organisation and delivery arrangement	Medical turnover and overload or healthcare providers[110]; government/NGO facility[90]; private hospital[33]	Attitude of healthcare providers[83]; mode of delivery[40 47 51 52 57 114]; hospitals refused to accept health insurance cards[81]
Facility & resources	Availability of medicines and supplies[70 95]; lack of healthcare facilities[115]	Type of healthcare facility[73 103 111]; number of available healthcare workers[41]; unavailability of female birth attendants[71]
Waiting time	Waiting time[60 70 95]	Waiting time[73 74 83 112]

For peer review only

For peer review only - http://bmjopen.bmj.com/site/about/guidelines.xhtml

Supply side: Provision of healthcare services

Eight articles described factors associated with provision of healthcare services in slums from the service providers' perspective. None of the studies reported personal and biological factors. Factors related to other categories are summarised below.

Cognitive and experiential factors: Odhiambo et al. reported slum residents' fear of side effects, size of tablet and misconceptions regarding treatment as the factors hindering drug administration activities by healthcare workers for a deworming programme in Kenya.[116] On the other hand, this study also reported a high demand for drugs from slum residents in the final year of this program because people realised that free treatment was to be ended.[116]

Socioeconomic factors: effective community mobilisation was a facilitator[116] whereas poor community support[117] and insufficient time allocated for providers to implement healthcare programmes[116] were barriers for provision of healthcare services in slums. In the deworming programme mentioned above, community health workers reported that direct observation of slum residents taking deworming drugs after meals was sometimes not feasible because slum residents skipped or age late at night due to food shortage.[116] Some slum residents demanded money to take the deworming drugs, either to facilitate purchase of food or to have their own share of the money that they perceived the community health workers would be paid by the programme if they complied with taking the drugs.[116]

Physical environment: poor sanitation,[116 118] presence of rodents and no pavement,[118] and bushy and unprotected environment[116] were reported as factors making the provision of healthcare services difficult in slums.

Cultural and religious factors: religious beliefs and mistrust of interventions,[116] lack of a shared understanding of the needs, purposes and consequences of family planning and pregnancy related services among slum residents and healthcare providers[61] were the barriers

For peer review only - http://bmjopen.bmj.com/site/about/guidelines.xhtml

for healthcare services provision. In the previous deworming programme, portrayal of unrelated death being linked to the programme and related negative publicity affected participants' compliance.[116]

Legal and political factors and policy: devolution of service delivery through downward transfer of funds and responsibilities from central/national government level to elected local bodies; management by professional managerial and technical cadres; tight organisation of public health services; and professional support from the state directorate of public health were found to strengthen public health service provision in Chennai slums compared with Delhi.[119] One study reported that policies affected healthcare provision negatively because of staff shortage arising from change and suspension of the appointment of health promotors, which led to overwork and lack of time to provide required care by healthcare staff. [112] In Brazil, home visits for the provision of healthcare services was hampered because slum residents could not present documents required to register for healthcare.[118] On the other hand, giving priority to socially less developed areas for strengthening the Family Health System in Brazil might have been associated with better service coverage for slum residents with tuberculosis compared with their urban non-slum counterparts.[120]

Health system: pay scale of frontline healthcare workers,[117] knowledge of intervention area by community health workers,[116] issues related to rigid task assignment by service managers,[118] requirement to follow standardised protocol,[118] demands from the management,[118] work burden[112 118] and no incentive,[117] insufficient time,[116] attitude[61] and support of healthcare providers[117] were associated with healthcare service provision in slums. Lack of community-based care (such as school-based education for reproductive health and community support networks for women),[112] affordability (price) and availability of medicine,[121] limited medical supplies[61 117] and infrastructural facilities,[117] inadequate space and equipment[118] also affected

For peer review only - http://bmjopen.bmj.com/site/about/guidelines.xhtml

service provision. (Table 3)

For peer review only

For peer review only - http://bmjopen.bmj.com/site/about/guidelines.xhtml

Table 3. Factors associated with provision of healthcare services in slums

**Cognitive and experiential factors**	
Perception/knowledge/experience/preference of health services	Fear of side effects, size of tablet and misconceptions regarding treatment, high demand for drugs in the final year of treatment[116]
**Socioeconomic factors**	
Income and wealth	Difficulty in directly observing deworming treatment at meal time due to food shortage[116]
Social support	Effective community mobilisation[116]; poor community support[117]; absence of community members during the drug administration exercise[116]; demand for incentives by community members to take deworming drugs[116]
**Physical environment**	
Environment of residence area	Environment (sanitation, territory)[118]; unsanitary environmental conditions[116]; inaccessibility (filthy and bush environment)[116]
**Cultural and religious factors**	
Religion	Religious beliefs and mistrust of interventions[116]
Sociocultural influence	Lack of shared understanding of the problems in community[61]; unrelated death and the associated negative publicity (of a deworming programme) by the media[116]
**Legal, political and policy factors**	
Policy issues	Devolution of service delivery transferring funds and responsibilities to elected local bodies [119]; management by professional managerial and technical cadres[119]; tight organisation of public health services[119]; professional support from the state directorate of public health[119]; healthcare policies[112]; policy prioritizing low social development areas [120]
Legal issues	Fear of requirement for formal registration[118]
**Health system factors**	
Cost	Pay scale of frontline healthcare workers[117]; medicine price[121]
Quality and safety of services	Knowledge of intervention area by community health workers[116]
Service organisation and delivery arrangement	Issues related to assignment of tasks[118]; requirement to follow standardised protocol [118]; demands from the management[118]; work overload[112,118]; documentation work/work burden/no incentive for work[117]; insufficient time[116]; attitude of healthcare providers[61]; lack of supportive staff[117]; community health worker familiarity with households led to warm reception[116]; opportunity to integrate mass drug administration with other health interventions[116]; presence of community health workers and their supervisory structure, and points of referral for serious side effects[116];
Facility & resources	Community-based care[112]; affordability and availability of medicine[121]; limited medical supplies[61,117]; infrastructural facilities[117]; inadequate space and equipment[118]

For peer review only

Comparison between slums and other settings

Six studies which met our inclusion criteria also included data from non-slum urban and/or rural areas and potentially allowed exploration of factors associated with healthcare access across different settings. Key findings from these studies are summarised in Table 4.

For peer review only - http://bmjopen.bmj.com/site/about/guidelines.xhtml

These recent studies showed a mixed and dynamic picture of healthcare access across slum and other settings and reported various factors associated with this. For example, the proportion of young children fully immunised was found to be lower in slums compared with non-slum urban setting but was higher than rural settings in Nigeria. Nevertheless the coverage improved over time across all settings.[52] While many common factors associated with full immunisation of young children were identified, giving birth in health facilities (as opposed to home) had a larger positive effect on subsequent immunisation coverage in slums compared with non-slum urban and rural settings.[52] A narrowing of gaps in delivery by skilled birth attendants between slum and non-slum urban settings over time and a reverse of the trend from having lower usage to higher usage of modern contraceptive methods by married women in slums versus urban non-slums were reported in Bangladesh.[41] Slum residents reported financial issues being the main reason for not taking prescribed drugs whereas getting better was the cited main reason for urban non-slum residents in Iran.[113] Better coverage of services and higher rates of treatment completion were reported for patients with tuberculosis in slums compared with non-slum urban setting in two studies in Brazil,[42 120] where a higher priority given to enhancing the Family Health system in socially less developed areas in recent years was suggested to be a likely factor associated with better service provision in slums.[120] (Table 4)

For peer review only - http://bmjopen.bmj.com/site/about/guidelines.xhtml

Table 4. Studies that examined factors associated with health care seeking and utilisation in both urban slum and non-slum urban and rural settings

Study & location	Differences in healthcare access	Associated factors
Obanewa (2020)[52]	***Fully-immunised child coverage (FIC)***<br>Proportion in slum lower than urban non-slum but higher than rural; proportions increased between 2003 and 2013 across all three settings	From multivariable regression*: year, birth order, antenatal attendance, maternal education level, religion, maternal age at child's birth, media exposure, region of the country, interaction between place of residence and place of delivery
Angeles (2019)[41]	***Use of modern contraceptive methods***<br>Proportion changed from being lower in slums in 2006 to being higher in slums in 2013 compared with urban non-slums	From multivariable regression*: parity, mother's age, mother's education attainment, socioeconomic status, interaction (slum × time period)
	Delivery by skilled birth attendant<br>Proportion substantially lower in slums compared with urban non-slums but the gaps narrowed over time)	From multivariable regression*: Residing in slums, parity, mother's age, mother's education attainment, length of stay in current city of residence, socioeconomic status, number of available community health worker, distance from health facility, interaction (slum x time period)
Islam (2018)[89]	***Antenatal care visits***<br>"there was a large inequality" between slum and urban non-slum (detail not reported)	Level of educational attainment, wealth index of the household
	Using contraceptive methods<br>"Prevalence rate higher among slum women" than urban non-slum women	Not reported
Tabrizi (2018)[113]	***Utilisation of health services in the past 30 days***<br>Similar utilisation overall, but with lower proportion received needed health services and used private clinics, higher use of vaccination and maternal health services, and lower use of services for heart failure and hypertension for slum residents compared with urban non-slum	High cost of services
	Home care services<br>Very little use both in slum and urban non-slum areas	High cost of services
	Prescribed drug during last visit to health facilities<br>Lower proportion for slum vs urban non-slum	Not reported
	Not taking drugs prescribed<br>Higher proportion for slum vs urban non-slum	Main reason: financial problems for slum vs getting better/feeling well for non-slum urban

For peer review only - http://bmjopen.bmj.com/site/about/guidelines.xhtml

Snyder (2016)[42]	***Directly observed treatment coverage for tuberculosis (TB)***<br>Higher for slum vs urban non-slum TB patients	Not examined
	Abandonment of TB treatment<br>Lower for slum vs urban non-slum TB patients	From multivariable regression*: residency in a slum, sex, age, extrapulmonary clinical disease, HIV/AIDS, interaction (directly observed treatment x residency in a slum)
Prado Junior (2016)[120]	***Coverage under Family Health system for TB patients***<br>Higher for slum vs urban non-slum	Giving the Family Health strategy priority to coverage of areas with lower social development

*From the model with most comprehensive adjustment including residency in slum as one of the variables; only factors that were statistically significant (at 5% level) are shown. AIDS: Acquired Immune Deficiency Syndrome; HIV: human immunodeficiency virus; TB: tuberculosis.

DISCUSSION

Statement of principle findings

This scoping review of recent literature examined demand side factors associated with slum residents' healthcare seeking behaviour and healthcare utilisation, as well as supply side factors associated with provision of health services in slums. We found over 80 studies related to the former, but only eight studies related to the latter. We identified different factors associated with access and provision of health services in slums, and mapped them to a conceptual framework developed and refined for this review into seven broad categories (Figure 2).

Findings in the context of existing literature

Even though previous reviews have investigated factors associated with healthcare access in various settings,[122 123] to our best knowledge this scoping review is the first that has comprehensively examined relevant factors across different service areas of health care in slums. Our findings are consistent with previous studies which highlighted common factors

For peer review only - http://bmjopen.bmj.com/site/about/guidelines.xhtml

associated with healthcare seeking and utilisation such as age, income and education.[122 124] We identified several factors that are particularly pertinent in slum settings, such as costs of healthcare,[19 21 61 64 66 70 74 77 87 94-96] lack of time due to slum residents' competing priorities[21 87 98] and issues arising from adverse physical environment,[69 94 116 118] security,[86 118] fear of formal registration due to distrust of the authorities[118] and proximity of healthcare facilities.[21 64 68 70 73 79 93 105-107] In addition, included studies showed that the effects of a given factor may differ between slum, urban non-slum and rural settings.[52]

Healthcare cost is a major barrier between the intention to seek care and actual utilisation of services. [95 115] Health insurance is one of the key measures to overcome this barrier[125] but results from previous studies showed that uptake of public insurance among slum residents could be low.[39] To improve the access to healthcare services among slum residents, policies that improve the uptake and utilisation of health insurance as well as reducing healthcare costs for slum residents need to be considered.

Several studies reported lack of time and competing priorities as a factor affecting healthcare-seeking behaviour[87 97 98] and health services utilisation.[21 61 72] This suggests a delicate balance between factors that individual slum residents have to strike when making decisions on healthcare seeking and utilisation. Var der Heijden et al. showed that health was considered as an asset for working ability in slums,[97] but paradoxically the ability to work often seems to impede healthcare seeking for health issues. This highlights the importance of considering slum residents' interest and priorities when providing healthcare services and promoting healthcare utilisation in slums.

Strengths and weaknesses of the review

This scoping review has several strengths. We conducted a comprehensive literature search

For peer review only - http://bmjopen.bmj.com/site/about/guidelines.xhtml

using generic terms related to slums with few other restrictions. The search was therefore likely to be sensitive for identifying relevant literature. Contemporary methodological guidelines for undertaking scoping reviews were followed,[22] and a conceptual framework which was adapted based on emerging findings was used to facilitate the organisation of evidence.

The review has enabled theory building and refinement of a conceptual framework. Our preliminary framework included six categories (Figure 1). During data coding and extraction, it emerged that many studies reported perception, knowledge, and experience of slum residents being associated with their healthcare-seeking and utilisation. We subsequently classified these factors as cognitive and experiential factors, which primarily consists of three subcategories: knowledge/experience of illness, perceived needs for accessing healthcare services, and perception/experience of healthcare services. These factors were influenced by other factors included in our original conceptual framework, but highlighted the crucial links between those factors and the ultimate actions by individual slum residents to access health services. Future interventions to promote health service utilisation for slum residents[126] could make use of our framework to develop programme theories and map out causal pathways.

This review also has some limitations. Given time constraint, we were only able to examine the most recent literature published in English, and have not examined the methodological quality of individual studies (which we noted to be quite varied) in detail. We attempted some preliminary synthesis to configure the identified evidence but have not explored the complex relationship between the factors identified in depth. Nevertheless, findings from this scoping review will provide a good foundation for further syntheses.

Methodological considerations

A challenge in the process of classifying and coding data is worth mentioning. Several

For peer review only - http://bmjopen.bmj.com/site/about/guidelines.xhtml

factors associated with healthcare seeking and utilisation can be viewed from different perspectives and therefore potentially be coded under different categories. For example, barriers for healthcare seeking and utilisation related to costs can be considered as socioeconomic issues from the slum dwellers' perspective but can also be viewed as health system issues for not offering the services in an affordable way. Indeed, previous access frameworks suggested that access is created and negotiated in a dynamic interchange between households/communities and healthcare workers/systems (i.e. demand and supply) on each access dimension.[29 127] In such situations, we tried to code a factor under the category that most directly reflects the original data through discussions within the review team (in the example of healthcare cost, the factor was coded primarily under socioeconomic factors rather than health system factors when the factor was reported by slum residents as a barrier); otherwise more than one category was coded (for example, bad experience from previous utilisation of health services was coded both as a cognitive and experiential factor and a health system factor).

Implication for research and practice

The multitude of factors identified in this review are often inter-related and inter-acting, and span across personal, family, community and society levels. For example, the association between occupation and healthcare utilisation were reported in several studies.[35 38 53 68 69 91] The effect of predominantly casual work undertaken by slum residents on their healthcare access could be mediated through working hours, income level, knowledge of health and available services, etc. There is also possibility that occupation was associated with health status and hence needs for healthcare services, instead of/in addition to behaviour of using healthcare services. Teasing out the complicated relationships between various determinants will require in-depth analysis and a more holistic approach to synthesising the evidence. Given the unique

For peer review only - http://bmjopen.bmj.com/site/about/guidelines.xhtml

features of individual slums, service planners and policy makers will need to examine these relationships with due consideration to the context specific to each locality and geospatial features and neighbourhood effects that characterise slum settings.[4]

We found far fewer studies that have examined health service providers' perspective than studies that have investigated factors associated with accessing healthcare from slum residents' perspective. There may be scope for greater research and policy attention to supply-side factors, including experiences and practices of local frontline healthcare providers, availability of healthcare facilities and infrastructure and policy to support them in order to overcome the many barriers highlighted from both supply and demand sides.

Although only six of the included studies explored factors associated with healthcare access or health service provision across slum and non-slum settings, they showed a generally encouraging picture that access to and provision of healthcare are continuously evolving (and often improving) in slums and other settings, and equality between different settings is not beyond reach.

CONCLUSION

This scoping review summarises a large body of recent literature evaluating factors associated with seeking and utilisation of healthcare by slum residents, but found substantially fewer studies examining factors associated with provision of health services from providers' perspective. Recent migration into slums; knowledge, perception (including misconception and distrust) and past experience of illness, healthcare needs and health services; financial constraint, competing priorities and inadequacy of social support; adverse physical environment and unfavourable locality; sociocultural expectations and stigma; lack of official recognition; and various problems in existing health system all contribute towards the

For peer review only - http://bmjopen.bmj.com/site/about/guidelines.xhtml

challenges faced by slum residents. Future research and policy aiming at improving healthcare services in slums should pay more attention to supply side issues ranging from individual healthcare providers and practices to structural and policy level factors to tackle different barriers faced by slum residents, which in turn need to be evaluated holistically and take into account local context and geospatial features of slums.

List of abbreviations

GRADE: Grading of Recommendations Assessment, Development and Evaluation

LMICs: Low and Middle income Countries

MMAT: Mixed Methods Appraisal Tool

WHO: World Health Organization

Ethics approval

Not applicable. This realist synthesis included literature that is available in the public domain and did not involve the collection of personal data.

Consent for publication

The authors were required to notify the funder of the research, the UK National Institute for Health Research (NIHR) prior to the publication of this manuscript. The funder did not otherwise play any roles in the preparation of the manuscript and decision to submit it.

Availability of data and materials

All data relevant to the study were included in the article or uploaded as supplementary information. No additional data were available.

Competing interests

The authors declare that they have no competing interest.

For peer review only - http://bmjopen.bmj.com/site/about/guidelines.xhtml

Acknowledgement and Funding

This research is funded by the NIHR Global Health Research Unit on Improving Health in Slums using UK aid from the UK Government to support global health research (Award/Grant number is not applicable). The views expressed in this publication are those of the author(s) and not necessarily those of the NIHR or the UK Department of Health and Social Care. MA gratefully acknowledges support provided by the Warwick Institute of Advanced Study Global Challenges Research Fund Fellowship No. IAS/32013/1914. FG receives funding as South Africa Research Chair in Health Policy and Systems from the National Research Foundation, South Africa (Award/Grant number is not applicable). RL is supported by the NIHR Applied Research Collaboration (ARC) West Midlands, UK (Award/Grant number is not applicable). YFC is supported by Warwick Evidence, which is a Technology Assessment Review team funded by the NIHR Evidence Synthesis Programme (Award/Grant number is not applicable). Upon submission, NA had joined the Population Health Sciences Institute, Newcastle University (UK).

Contributor

JEP, BH, MA, FG and YFC conceptualised the scoping review; JEP carried out literature searches; JEP, PK, GY, OO, and YFC participated in study screening and coding; JEP and YFC performed data charting and drafted in initial manuscript. NA, PG and RL provided critical input during the drafting of the manuscript. All authors commented on and contributed to the revision of subsequent versions and approved the final version for submission.

Collaborators

The Improving Health in Slums Collaborative:

African Population and Health Research Centre (APHRC), Nairobi, Kenya:

Pauline Bakibinga, Caroline Kabaria, Ziraba Kasiira, Peter Kibe, Lyagamula Kisia, Catherine

For peer review only - http://bmjopen.bmj.com/site/about/guidelines.xhtml

Kyobutungi, Nelson Mbaya, Blessing Mberu, Shukri Mohammed, Anne Njeri.

Aga Khan University, Karachi, Pakistan:

Iqbal Azam, Romaina Iqbal, Ahsana Nazish, Narjis Rizvi.

Independent University, Bangladesh, Dhaka, Bangladesh:

Syed Shifat Ahmed, Nazratun Choudhury, Omar Rahman, Rita Yusuf.

Nigerian Academy of Sciences, Lagos, Nigeria:

Doyin Odubanjo.

University of Ibadan, Ibadan, Nigeria:

Motunrayo Ayobola, Olufunke Fayehun, Akinyinka Omigbodun, Mary Osuh, Eme Owoaje, Olalekan Taiwo.

University of Birmingham, Birmingham, UK:

Richard Lilford, Jo Sartori, Samuel Watson.

University of Lancaster, Lancaster, UK:

Peter Diggle.

University of Warwick, Coventry, UK:

Navneet Aujla, João Porto de Albuquerque, Yen-Fu Chen, Paramjit Gill, Frances Griffiths, Bronwyn Harris, Jason Madan, Oyinlola Oyebode, Ji-Eun Park, Simon Smith, Grant Tregonning, Olalekan Uthman, Ria Wilson, Godwin Yeboah.

REFERENCES

1. United Nations. The Sustainable Development Goals Report, 2019.

2. Kosamkar A. Problems in urban society. *EPRA Int J Multidiscip Res* 2020;6.

3. Ezeh A, Oyebode O, Satterthwaite D, et al. The history, geography, and sociology of slums and the health problems of people who live in slums. *Lancet* 2017;389:547-58.

For peer review only - http://bmjopen.bmj.com/site/about/guidelines.xhtml

4. Lilford R, Kyobutungi C, Ndugwa R, et al. Because space matters: conceptual framework to help distinguish slum from non-slum urban areas. *BMJ Glob Health* 2019;4:e001267.

5. Zulu EM, Beguy D, Ezeh AC, et al. Overview of migration, poverty and health dynamics in Nairobi City's slum settlements. *J Urban Health* 2011;88 Suppl 2:S185-99.

6. Michiani MV, Asano J. Physical upgrading plan for slum riverside settlement in traditional area: A case study in Kuin Utara, Banjarmasin, Indonesia. *Front Archit Res* 2019;8:378-95.

7. Winter SC, Dreibelbis R, Dzombo MN, et al. A mixed-methods study of women's sanitation utilization in informal settlements in Kenya. *PLoS One* 2019;14:e0214114.

8. Salinas DA, Fouts HN, Neitzel CL, et al. Young Children's Social Networks in an Informal Urban Settlement in Kenya: Examining Network Characteristics Among Kamba, Kikuyu, Luo, and Maasai Children. *J Cross-Cult Psychol* 2019;50:639-58.

9. Prayitno G, Sari N, Putri IK. Social capitl in poversity alleviation through pro-poor tourism concept in slum area (case study: Kelurahan jodipan, Malang city). *Int J GEOMATE* 2019;16:131-37.

10. Khalil D. The Flexible Governance of Water in Cairo's Informal Areas. *Water* 2019;11:1644.

11. Portner CC, Su YH. Differences in Child Health Across Rural, Urban, and Slum Areas: Evidence From India. *Demography* 2018;55:223-47.

12. Mberu BU, Haregu TN, Kyobutungi C, et al. Health and health-related indicators in slum, rural, and urban communities: a comparative analysis. *Glob Health Action* 2016;9:33163.

13. Mlangeni L, Makola L, Naidoo I, et al. Factors associated with physical activity in south africa: Evidence from a national population based survey. *Open Public Health Journal* 2018;11:516-25.

For peer review only

For peer review only - http://bmjopen.bmj.com/site/about/guidelines.xhtml

14. Rebecca L. Nunn, Sarah H. Kehoe, Harsha Chopra, et al. Dietary micronutrient intakes among women of reproductive age in Mumbai slums. *Euro J of Clinical Nutrition* 2019;73:1536–45.

15. Samal J. Perception and knowledge of tuberculosis and its services among slum dwellers in Chhattisgarh. *Indian J Respir Care* 2017;6:828-31.

16. Khan MZ, Shujaa MD, Iftikhar H. Utilization of ante-natal services among reproductive age women of Bahawalpur. *Indo Am J Pharm Sci* 2018;5:11355-65.

17. Duy Kien V, Van Minh H, Bao Giang K, et al. Horizontal inequity in public health care service utilization for non-communicable diseases in urban Vietnam. *Glob Health Action* 2014;7:24919.

18. Amiresmaili M, Yazdi-Feyzabadi V, Heidarijamebozorgi M. Health services utilization among slum dwellers: An experience from Iran. *J Educ Health Promot* 2019;8:210.

19. McNairy ML, Tymejczyk O, Rivera V, et al. High Burden of Non-communicable Diseases among a Young Slum Population in Haiti. *J Urban Health* 2019;96:797-812.

20. Sverdlik A. Ill-health and poverty: a literature review on health in informal settlements. *Environ Urban* 2011;23:123-55.

21. Crocker-Buque T, Mindra G, Duncan R, et al. Immunization, urbanization and slums - a systematic review of factors and interventions. *BMC Public Health* 2017;17:556.

22. Peters MDJ, Godfrey C, McInerney P, et al. Chapter 11: Scoping Reviews. In: Aromataris E, Munn Z (Editors). Joanna Briggs Institute Reviewer's Manual 2017

23. Center for Open Science 2020. Slum health healthcare access and provision of services scoping review protocol 05082020.pdf (Version: 1). Available from: https://osf.io/mj6kp/ [Accessed 9 Jun 2021].

24. Ahmed S, Ajisola M, Azeem K, et al. Impact of the societal response to COVID-19 on

For peer review only - http://bmjopen.bmj.com/site/about/guidelines.xhtml

access to healthcare for non-COVID-19 health issues in slum communities of Bangladesh, Kenya, Nigeria and Pakistan: results of pre-COVID and COVID-19 lockdown stakeholder engagements. *BMJ Glob Health* 2020;5:e003042.

25. Improving Health in Slums C. A protocol for a multi-site, spatially-referenced household survey in slum settings: methods for access, sampling frame construction, sampling, and field data collection. *BMC Med Res Methodol* 2019;19:109.

26. Peduzzi P, Chatenoux B, Dao H, et al. Global trends in tropical cyclone risk. *Nature climate change* 2012;2:289-94.

27. Effective Practice and Organisation of Care (EPOC). EPOC Taxonomy 2015. Available from: https://epoc.cochrane.org/epoc-taxonomy [Accessed 27 Jul 2020].

28. Lavis JN, Wilson MG, Moat KA, et al. Developing and refining the methods for a 'one-stop shop' for research evidence about health systems. *Health Res Policy Syst* 2015;13:10.

29. Levesque JF, Harris MF, Russell G. Patient-centred access to health care: conceptualising access at the interface of health systems and populations. *Int J Equity Health* 2013;12:18.

30. National Cancer Institute. Theory at a Glance: A Guide for Health Promotion Practice, 2005.

31. Mpanje D, Gibbons P, McDermott R. Social capital in vulnerable urban settings: an analytical framework. *J Int Humanit Action* 2018;3:4.

32. Tricco AC, Lillie E, Zarin W, et al. PRISMA Extension for Scoping Reviews (PRISMA-ScR): Checklist and Explanation. *Ann Intern Med* 2018;169:467-73.

33. Pune Municipal Corporation. City Health Plan 2016 - 2020, 2016. Available from: https://www.healthynewbornnetwork.org/hnn-content/uploads/Pune-City-Health-Plan.pdf [Accessed 10 Jun 2021]

34. Razzaque A, Clair K, Chin B, et al. Association of Time since Migration from Rural to

For peer review only - http://bmjopen.bmj.com/site/about/guidelines.xhtml

Urban Slums and Maternal and Child Outcomes: Dhaka (North and South) and Gazipur City Corporations. *J Urban Health* 2020;97:158-70.

35. Geddam JB, Ponna SN, Kommu PR, et al. Utilization of maternal health services by the migrant population living in the non-notified slums of Hyderabad city, India. *Indian J Community Health* 2017;29:29-38.

36. Singh S, Sahu D, Agrawal A, et al. Ensuring childhood vaccination among slums dwellers under the National Immunization Program in India - Challenges and opportunities. *Prev Med* 2018;112:54-60.

37. Lae WL, Jayasvasti I, Mongkolchati A, et al. Utilization of immunization service and predictors among under 3-year-old children in urban slums of Chanmyathazi Township, Mandalay, Myanmar. *J Med Assoc Thai* 2018;101:1085-92.

38. Horng L, Kakoly NS, Abedin J, et al. Effect of household relocation on child vaccination and health service utilisation in Dhaka, Bangladesh: a cross-sectional community survey. *BMJ Open* 2019;9: e026176.

39. Kusuma YS, Pal M, Babu BV. Health Insurance: Awareness, Utilization, and its Determinants among the Urban Poor in Delhi, India. *J Epidemiol Glob Health* 2018;8:69-76.

40. Upadhyai N, Gupta SK. Utilization of Postnatal Care Services and Factors Affecting It among Women of Urban Slums in Dehradun, Uttarakhand. *Indian J Community Health* 2019;31:470-76.

41. Angeles G, Ahsan KZ, Streatfield PK, et al. Reducing Inequity in Urban Health: Have the Intra-urban Differentials in Reproductive Health Service Utilization and Child Nutritional Outcome Narrowed in Bangladesh? *J Urban Health* 2019;96:193-207.

42. Snyder RE, Marlow MA, Phuphanich ME, et al. Risk factors for differential outcome

For peer review only

following directly observed treatment (DOT) of slum and non-slum tuberculosis patients: a retrospective cohort study. *BMC Infectious Diseases* 2016;16:494.

43. Kulkarni S, Mishra G, Dussane R, et al. Determinants of compliance to breast cancer screening and referral among women from urban slums in India. *Eur J Cancer* 2016;54:S51.

44. Lungu EA, Darker C, Biesma R. Determinants of healthcare seeking for childhood illnesses among caregivers of under-five children in urban slums in Malawi: a population-based cross-sectional study. *BMC Pediatr* 2020;20:20.

45. Kerai S, Nisar I, Muhammad I, et al. A Community-Based Survey on Health-Care Utilization for Pneumonia in Children in Peri-Urban Slums of Karachi, Pakistan. *Am J Trop Med Hyg* 2019;101:1034-41.

46. Renzaho AM, Kamara JK, Georgeou N, et al. Sexual, Reproductive Health Needs, and Rights of Young People in Slum Areas of Kampala, Uganda: A Cross Sectional Study. *PLoS One* 2017;12:e0169721.

47. Shrestha S, Shrestha M, Wagle RR, et al. Predictors of incompletion of immunization among children residing in the slums of Kathmandu valley, Nepal: a case-control study. *BMC Public Health* 2016;16:970.

48. Sharma D, Pokharel HP, Budhathoki SS, et al. Antenatal Health Care Service Utilization in Slum Areas of Pokhara Sub-Metropolitan City, Nepal. *J Nepal Health Res Counc* 2016;14:39-46.

49. Mishra S, Kusuma YS, Babu BV. Treatment-seeking and out-of-pocket expenditure on childhood illness in a migrant tribal community in Bhubaneswar, Odisha State, India. *Paediatr Int Child Health* 2017;37:181-87.

50. Kar SS, Kalaiselvi S, Archana R, et al. Is rule of halves still an occurrence in South India: Findings from community-based survey in a selected urban area of Puducherry. *J Postgrad*

For peer review only - http://bmjopen.bmj.com/site/about/guidelines.xhtml

Med 2017;63:232-36.

51. Devasenapathy N, Jerath SG, Sharma S, et al. Determinants of childhood immunisation coverage in urban poor settlements of Delhi, India: a cross-sectional study. *BMJ Open* 2016;6:e013015.
52. Obanewa OA, Newell ML. The role of place of residency in childhood immunisation coverage in Nigeria: analysis of data from three DHS rounds 2003-2013. *BMC public health* 2020;20:123.
53. Shrestha S, Shrestha DK. Utilization of Maternal Health Care Services among Mothers Residing at Slum Area. *J Nepal Health Res Counc* 2019;17:193-99.
54. Neyaz A, Ahmed MS, Sahu PC. Preference and practices regarding place of childbirth in the slums of a city in Northern India. *J Pioneer Med Sci* 2016;6:33-36.
55. Abd El Fatah SAM, El Habashy EM, Ismail HAH. Role of receipt of antenatal care in subsequent contraceptive use at primary health care centres serving slum areas of Cairo, Egypt. *Eur J Contracept Reprod Health Care* 2019;24:356-61.
56. Mishra S, Kusuma YS, Babu BV. Mother's Recognition of and Treatment Triggers for Common Childhood Illnesses among Migrant Santal Tribe Living in Bhubaneswar, Odisha, India. *J Trop Pediatr* 2017;63:301-06.
57. Sahu KS, Bharati B. Out-of-Pocket health expenditure and sources of financing for delivery, postpartum, and neonatal health in urban slums of Bhubaneswar, Odisha, India. *Indian J Public Health* 2017;61:67-73.
58. Mistry N, Rangan S, Dholakia Y, et al. Durations and Delays in Care Seeking, Diagnosis and Treatment Initiation in Uncomplicated Pulmonary Tuberculosis Patients in Mumbai, India. *PLoS One* 2016;11:e0152287.
59. Thomson KA, Telfer B, Opondo AP, et al. Navigating the risks of prevention of mother to

For peer review only - http://bmjopen.bmj.com/site/about/guidelines.xhtml

child transmission (PMTCT) of HIV services in Kibera, Kenya: Barriers to engaging and remaining in care. *PLoS One* 2018;13: e0191463.

60. Kamati M, Godman B, Kibuule D. Prevalence of Self-Medication for Acute Respiratory Infections in Young Children in Namibia: Findings and Implications. *J Res Pharm Pract* 2019;8:220-24.

61. Kaba M, Taye G, Getachew S, et al. Perceived barriers to health care for residents in vulnerable urban centers of Ethiopia. *Ethiop J Health Dev* 2020;34:4-11.

62. Verma H, Sagili KD, Zachariah R, et al. Do incentivised community workers in informal settlements influence maternal and infant health in urban India? *Public Health Action* 2017;7:61-66.

63. Pugliese-Garcia M, Heyerdahl LW, Mwamba C, et al. Factors influencing vaccine acceptance and hesitancy in three informal settlements in Lusaka, Zambia. *Vaccine* 2018;36:5617-24.

64. Ramagiri R, Kannuri NK, Lewis MG, et al. Evaluation of whether health education using video technology increases the uptake of screening for diabetic retinopathy among individuals with diabetes in a slum population in Hyderabad. *Indian J ophthalmol* 2020;68:S37-S41.

65. Sumudrika Ilankoon I, Evangeline Goonewardena C, Fernandopulle R, et al. Women's Understanding and Cultural Practices Related to Vaginal Discharge: A Qualitative Study. *Nurs Midwifery Stud* 2018;7:74-80.

66. Jayaweera RT, Ngui FM, Hall KS, et al. Women's experiences with unplanned pregnancy and abortion in Kenya: A qualitative study. *PLoS One* 2018;13:e0191412.

67. Kusuma YS, Kaushal S, Garg R, et al. Birth preparedness and determinants of birth place among migrants living in slums and slum-like pockets in Delhi, India. *Sex Reprod Healthc*

For peer review only - http://bmjopen.bmj.com/site/about/guidelines.xhtml

2018;16:160-66.

68. Adane M, Mengistie B, Mulat W, et al. Utilization of health facilities and predictors of health-seeking behavior for under-five children with acute diarrhea in slums of Addis Ababa, Ethiopia: a community-based cross-sectional study. *J Health Popul Nutr* 2017;36:9.

69. Owusu-Ansah FE, Tagbor H, Togbe MA. Access to health in city slum dwellers: The case of Sodom and Gomorrah in Accra, Ghana. *Afr J Prim Health Care Fam Med* 2016;8:e1-7.

70. Lungu EA, Biesma R, Chirwa M, et al. Healthcare seeking practices and barriers to accessing under-five child health services in urban slums in Malawi: a qualitative study. *BMC Health Serv Res* 2016;16:410.

71. Getachew S, Kaba M, Gizaw M, et al. Health service access, utilization and prevailing health problems in the urban vulnerable sections of Ethiopia. *Ethiop J Health Dev* 2020;34:12-23.

72. Kaba M, Taye G, Gizaw M, et al. Maternal health service utilization in urban slums of selected towns in Ethiopia: Qualitative study. *Ethiop J Health Dev* 2017;31:96-102.

73. Owiti A, Oyugi J, Essink D. Utilization of Kenya's free maternal health services among women living in Kibera slums: a cross-sectional study. *Pan Afr Med J* 2018;30:86.

74. Castiglione D, Lovasi GS, Carvalho MS. Perceptions and Uses of Public and Private Health Care in a Brazilian Favela. *Qual Health Res* 2018;28:159-72.

75. Wairiuko JM, Cheboi SK, Ochieng GO, et al. Access to Healthcare Services in Informal Settlement: Perspective of the Elderly in Kibera Slum Nairobi-Kenya. *Ann Med Health Sci Res* 2017;7:5-9.

76. Martinez Perez G, Cox V, Ellman T, et al. 'I Know that I Do Have HIV but Nobody Saw Me': Oral HIV Self-Testing in an Informal Settlement in South Africa. *PLoS ONE* 2016;11:e0152653.

For peer review only

77. Sudhinaraset M, Beyeler N, Barge S, et al. Decision-making for delivery location and quality of care among slum-dwellers: a qualitative study in Uttar Pradesh, India. *BMC Pregnancy Childbirth* 2016;16:148.

78. Otieno PO, Wambiya EOA, Mohamed SF, et al. Prevalence and factors associated with health insurance coverage in resource-poor urban settings in Nairobi, Kenya: a cross-sectional study. *BMJ Open* 2019;9:e031543.

79. Cernauskas V, Angeli F, Jaiswal AK, et al. Underlying determinants of health provider choice in urban slums: results from a discrete choice experiment in Ahmedabad, India. *BMC Health Serv Res* 2018;18:473.

80. Manandhar K, Bajcharya K, Prajapati R, et al. Prevalence and Predictors of Incomplete Immunization among Children Residing in the Slums of Kathmandu Valley: A Community Based Door-to-Door Survey. *Kathmandu Univ Med J* 2018;16:8-13.

81. Gupta S. Awareness and utilization of Rashtriya Swasthaya Bima Yojana and its implications for access to health care by the poor in slum areas of Delhi. *Health Systems* 2017;6:242-59.

82. Oluoch P, Orwa J, Lugalia F, et al. Application of psychosocial models to Home-Based Testing and Counseling (HBTC) for increased uptake and household coverage in a large informal urban settlement in Kenya. *Pan Afr Med J* 2017;27:285.

83. Madan NV. Ethnographic Perspectives on Slum-dwelling Women's Access to Primary Care: The Case of Pune, India. *Urbanities* 2019;9:114-30.

84. Atusiimire LB, Waiswa P, Atuyambe L, et al. Determinants of facility based-deliveries among urban slum dwellers of Kampala, Uganda. *PLoS ONE* 2019;14:e0214995.

85. Iberico MM, Montoya R, Valiente B, et al. Uptake and utilization of tuberculosis preventive therapy in a Peruvian Peri-urban Shantytown. *Ann Glob Health* 2016;82 (3):366.

For peer review only - http://bmjopen.bmj.com/site/about/guidelines.xhtml

86. Hutain J, Perry HB, Koffi AK, et al. Engaging communities in collecting and using results from verbal autopsies for child deaths: an example from urban slums in Freetown, Sierra Leone. *J Glob Health* 2019;9:010419.

87. Misra V, Vashist P, Singh SS, et al. Awareness and eye health-seeking practices for cataract among urban slum population of Delhi: The North India eye disease awareness study. *Indian J Ophthalmol* 2017;65:1483-88.

88. Das M, Angeli F, Krumeich A, et al. The gendered experience with respect to health-seeking behaviour in an urban slum of Kolkata, India. *Int J Equity Health* 2018;17:24.

89. Islam M. Use of reproductive health care services among urban slum women in Bangladesh. *Eur J Public Health* 2018;28:67.

90. Aleemi AR, Khaliqui H, Faisal A. Challenges and Patterns of Seeking Primary Health Care in Slums of Karachi: A Disaster Lurking in Urban Shadows. *Asia Pac J Public Health* 2018;30:479-90.

91. Rahman S. A Comparative Study of Intrauterine Contraceptive Device Utilization among Currently Married Women in a Rural Area of Rani Block and Urban Slums of Guwahati City. *Int J Sci Study* 2016;4:55-59.

92. Jolly SP, Rahman M, Afsana K, et al. Evaluation of Maternal Health Service Indicators in Urban Slum of Bangladesh. *PLoS ONE* 2016;11:e0162825.

93. Sadhna S, Kajal J, Debabratta R, et al. Utilisation of maternal health services and its predictors in slum population. *Acta Medica International* 2016;3:56-62.

94. Sheehy G, Aung Y, Foster AM. "She Learned it from her Mother and Grandmother": Women's Experiences with Delivery and Post-partum Practices in Peri-urban Yangon, Myanmar. *Matern Child Health J* 2016;20:854-61.

95. Lungu EA, Guda Obse A, Darker C, et al. What influences where they seek care?

For peer review only

Caregivers' preferences for under-five child healthcare services in urban slums of Malawi: A discrete choice experiment. *PLoS One* 2018;13:e0189940.

96. Nasrin M, Sarker MNI, Huda N. Determinants of health care seeking behavior of pregnant slums dwellers in Bangladesh. *Medical Science* 2019;23:35-41.

97. van der Heijden J, Gray N, Stringer B, et al. 'Working to stay healthy', health-seeking behaviour in Bangladesh's urban slums: a qualitative study. *BMC Public Health* 2019;19:600.

98. Gaiha SM, Gillander Gadin K. 'No time for health:' exploring couples' health promotion in Indian slums. *Health Promot Int* 2020;35:70-81.

99. Wingfield T, Tovar MA, Huff D, et al. Socioeconomic support to improve initiation of tuberculosis preventive therapy and increase tuberculosis treatment success in Peru: a household-randomised, controlled evaluation. *Lancet* 2017;389:S16.

100. Angeli F, Ishwardat ST, Jaiswal AK, et al. Socio-Cultural Sustainability of Private Healthcare Providers in an Indian Slum Setting: A Bottom-of-the-Pyramid Perspective. *Sustainability* 2018;10:4702.

101. Agrawal D, Tyagi N, Dhakar JS, Chaturvedi M. Awareness and utilization of Geriatric Welfare Schemes among urban elderly population of District Gautam Budh Nagar. *Indian J Community Health* 2019;31:315-21.

102. Dasgupta P, Bhattacherjee S, Mukherjee A, et al. Vaccine hesitancy for childhood vaccinations in slum areas of Siliguri, India. *Indian J Public Health* 2018;62:253-58.

103. Tebekaw Y, Mashalla YJ, Thupayagale-Tshweneagae G. The adequacy of antenatal care services among slum residents in Addis Ababa, Ethiopia. *Ann Glob Health* 2016;82:527-28.

104. Viramgami AP, Verma PB, Vala MC, et al. A Cross-Sectional Study to Assess

For peer review only

For peer review only - http://bmjopen.bmj.com/site/about/guidelines.xhtml

Reproductive and Child Health Profile of Working Women Residing in Urban Slums of Rajkot City. *Indian J Community Med* 2019;44:313-16.

105. Muralidharan A. Constrained Choices? Menstrual Health and Hygiene Needs Among Adolescents in Mumbai Slums. *Indian J Gender Stud* 2019;26:12-39.

106. MacPherson P, Khundi M, Nliwasa M, et al. Disparities in access to diagnosis and care in Blantyre, Malawi, identified through enhanced tuberculosis surveillance and spatial analysis. *BMC Med* 2019;17:21.

107. Schultz JS, Muema S, Ouma A, et al. Timeliness of vaccination of vacination in an urban slum in Nairobi, Kenya. *Am J Trop Med Hyg* 2017;95:567.

108. Ahmed S, Adams AM, Islam R, et al. Impact of traffic variability on geographic accessibility to 24/7 emergency healthcare for the urban poor: A GIS study in Dhaka, Bangladesh. *PLoS One* 2019;14:e0222488.

109. Williams A, Sarker M, Ferdous ST. Cultural Attitudes toward Postpartum Depression in Dhaka, Bangladesh. *Med Anthropol* 2018;37:194-205.

110. Athie K, Dowrick C, Menezes AL, et al. Anxious and depressed women's experiences of emotional suffering and help seeking in a Rio de Janeiro favela. *Cien Saude Colet* 2017;22:75-86.

111. Kuria N, Reid A, Owiti P, et al. Compliance with follow-up and adherence to medication in hypertensive patients in an urban informal settlement in Kenya: comparison of three models of care. *Trop Med Int Health* 2018;23:785-94.

112. Mataboge MLS, Beukes S, Nolte AGW. The experiences of clients and healthcare providers regarding the provision of reproductive health services including the prevention of HIV and AIDS in an informal settlement in Tshwane. *Health SA Gesondheid* 2016;21:67-76.

For peer review only - http://bmjopen.bmj.com/site/about/guidelines.xhtml

113. Tabrizi JS, Farahbakhsh M, Bazargani HS, et al. Health Services Utilization and Responsiveness: A comparison of Slum and Non-slum Regions in Tabriz, Iran. *Medical Science* 2018;22:577-82.

114. Sharma S, Verma PB, Viramgami AP, et al. Analysis of Out-of-Pocket Expenditure in Utilization of Maternity Care Services in Urban Slums of Rajkot City, Gujarat. *Indian J Community Med* 2018;43:215-9.

115. Wekesah FM, Kyobutungi C, Grobbee DE, et al. Understanding of and perceptions towards cardiovascular diseases and their risk factors: a qualitative study among residents of urban informal settings in Nairobi. *BMJ Open* 2019;9:e026852.

116. Odhiambo GO, Musuva RM, Odiere MR, et al. Experiences and perspectives of community health workers from implementing treatment for schistosomiasis using the community directed intervention strategy in an informal settlement in Kisumu City, western Kenya. *BMC Public Health* 2016;16:986.

117. Patil SK, Ahmed MM. Assessment of integrated child development services in urban slums of Belagavi city, Karnataka. *Indian J Public Health Res Dev* 2016;7:208-11.

118. Agonigi RC, Carvalho SM, Freire MAM, et al. The production of care in the routine of Family Health Teams. *Rev Bras Enferm* 2018;71:2659-65.

119. Das Gupta M, Dasgupta R, Kugananthan P, et al. Flies without Borders: Lessons from Chennai on Improving India's Municipal Public Health Services. *J Dev Stud* 2020;56:907-28.

120. Prado Junior JC, Virgilio TC, Medronho Rde A. Cure rates for tuberculosis in the municipality of Rio de Janeiro, Brazil, in 2012 compared with coverage by, and time of establishment of, Family Health units, and socio-economic and demographic factors. *Ciencia & Saude Coletiva* 2016;21:1491-8.

For peer review only - http://bmjopen.bmj.com/site/about/guidelines.xhtml

121. Ongarora D, Karumbi J, Minnaard W, et al. Medicine Prices, Availability, and Affordability in Private Health Facilities in Low-Income Settlements in Nairobi County, Kenya. *Pharmacy (Basel)* 2019;7:40.

122. Liu Y, Kong Q, Yuan S, et al. Factors influencing choice of health system access level in China: A systematic review. *PLoS One* 2018;13:e0201887.

123. Banke-Thomas OE, Banke-Thomas AO, Ameh CA. Factors influencing utilisation of maternal health services by adolescent mothers in Low-and middle-income countries: a systematic review. *BMC Pregnancy Childbirth* 2017;17:65.

124. Mendoza-Sassi R, Béria JU. Health services utilization: a systematic review of related factors. *Cad Saude Publica* 2001;17:819-32.

125. Ministerial Leadership Initiative for Global Health. Reducing Financial Barriers to Reproductive Health Care: Experiences with Free Care and Health Insurance 2010. Available from: https://www.aspeninstitute.org/wp-content/uploads/files/content/docs/pubs/mli_issue_brief_reducing_financial_barriers_to_reproductive_health_care.pdf [Accessed 10 Jun 2021]

126. Lilford RJ, Oyebode O, Satterthwaite D, et al. Improving the health and welfare of people who live in slums. *Lancet* 2017;389:559-70.

127. McIntyre D, Thiede M, Birch S. Access as a policy-relevant concept in low- and middle-income countries. *Health Econ Policy Law* 2009;4:179-93.

For peer review only - http://bmjopen.bmj.com/site/about/guidelines.xhtml

Figure legends.

Figure 1. Preliminary framework for factors influencing slum residents' healthcare seeking behaviour and utilization of health services and the provision of services in slum settings

Figure 2. Updated framework of factors influencing healthcare-seeking behaviour/healthcare utilisation/provision of healthcare services in slums.

Figure 3. Flowchart

For peer review only

For peer review only - http://bmjopen.bmj.com/site/about/guidelines.xhtml

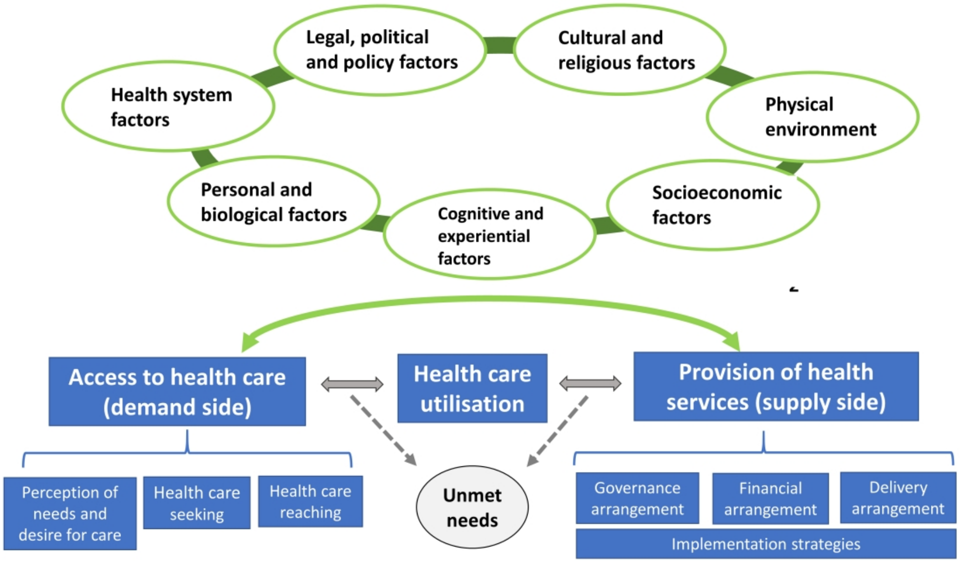

Figure 1. Preliminary framework for factors influencing slum residents' healthcare seeking behaviour and utilization of health services and the provision of services in slum settings

338x190mm (300 x 300 DPI)

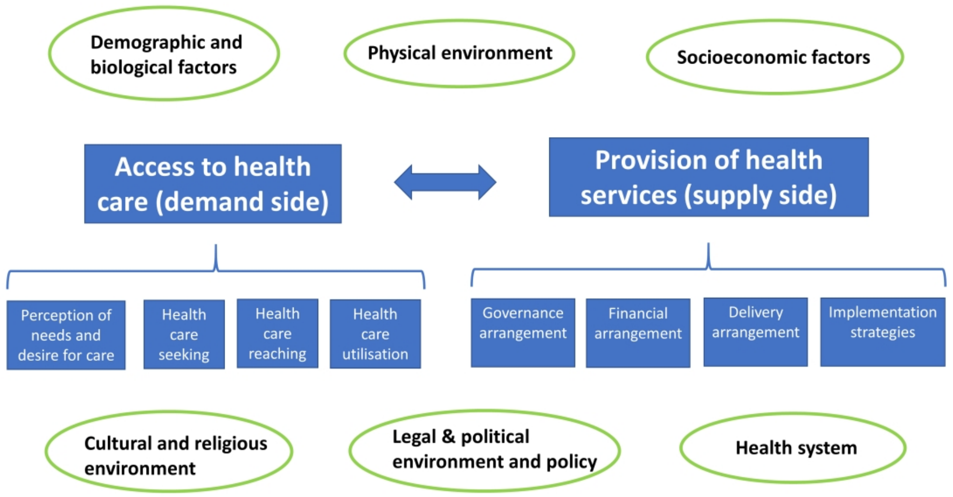

Figure 2. Updated framework of factors influencing healthcare-seeking behaviour/healthcare utilisation/provision of healthcare services in slums.

338x190mm (300 x 300 DPI)

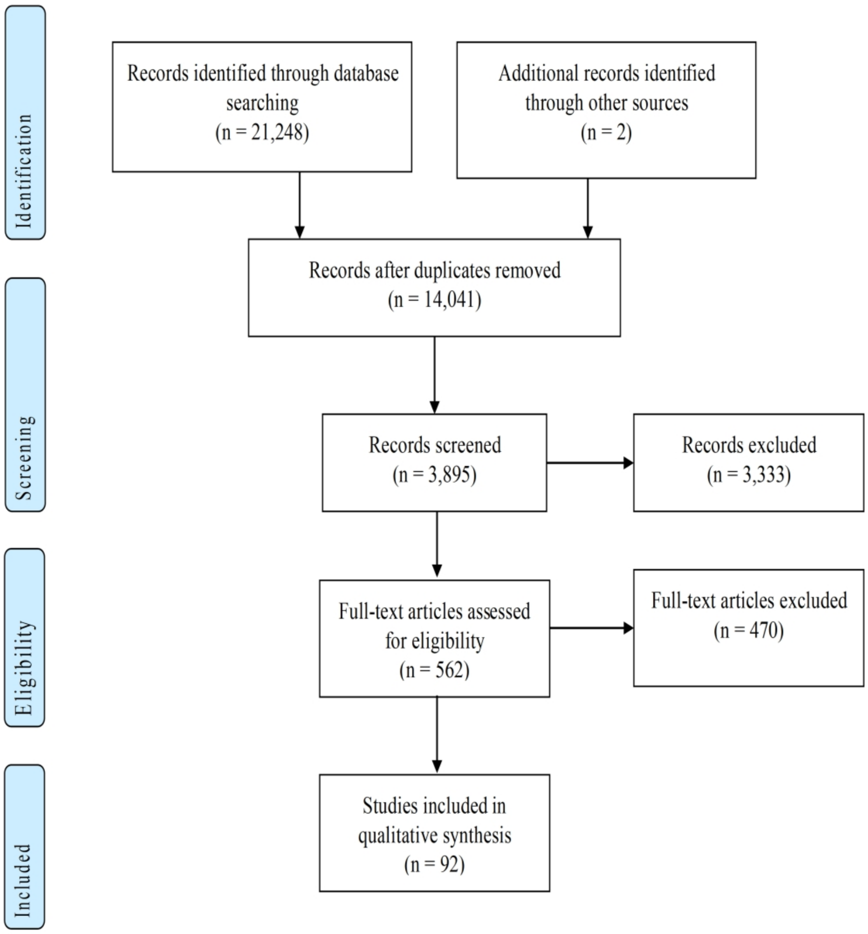

Figure 3. Flowchart

338x451mm (300 x 300 DPI)

Supplemental Table 1. Healthcare-seeking behaviours of slum residents reported by included studies and associated factors.

Subcategory	Author (year)	Participants	Country	Study design	Methodology	Outcome	Factors of interest for this review
General healthcare seeking behaviour	Gaiha (2020)[98]	Hetero-couples in slums	India	Cross-sectional study	Mixed method	Ability to attend any health promotion activity	Lack of time related to work as a reason for low male participation
	van der Heijden (2019)[97]	Female workers and key informants in slums	Bangladeshi	Cross-sectional study	Qualitative	Healthcare-seeking behaviour	Competing interest (ability to work and income)
	Aleemi (2018)[90]	Slum residents	Pakistan	Cross-sectional study	Quantitative	Healthcare-seeking behaviour	Household income; government facility; NGO facility
	Wekesah (2019)[115]	Slum residents	Kenya	Cross-sectional study	Qualitative	Care-seeking and adherence to treatment for CVD	Cost of healthcare; lack of healthcare facilities
	Kar (2017)[50]	Slum residents	India	Cross-sectional study	Quantitative	Undiagnosed hypertension	Sex; poverty; unskilled laborer; literacy
	Mistry (2016)[58]	TB patients in slums	India	Retrospective study	Quantitative	Delays in care seeking	Perception of symptoms; home remedies; not want to miss work; provider shopping; delay in advising TB-relevant tests; referral.
	Kulkarni (2016)[43]	Women in slums	India	Cross-sectional study	Quantitative	Participation in breast cancer screening	Age; education; religion; Mother tongue; occupation; marital status; tobacco habits; family history of cancer; history of cancer screening
	Misra (2017)[87]	Slum households	India	Cross-sectional study	Quantitative	Health-seeking practice for cataract	Lack of time, fear of surgery, financial difficulties
	Ramagiri (2020)[64]	Slum residents with diabetes	India	Case control study	Mixed-method	Uptake of diabetic retinopathy screening	Realization of consequences of disease; travel assistance and proximity of the screening facility; absence of an accompanying person; cost
Healthcare for	Lungu	Caregivers of	Malawi	Cross-	Quantitative	Healthcare-seeking	Age; education; illness was

For peer review only

children	(2020)[44]	children under 5 years of age in slums		sectional study		behaviour	perceived to be severe; fever; home management of childhood illness
						Timely healthcare seeking behaviour	Home management of childhood; knowledge of caregivers about child danger signs
	McNairy (2019)[19]	Slum households with children ≤ 5 years old	Haiti	Cross-sectional study	Quantitative	Healthcare access	Inability to afford care
	Hutain (2019)[86]	Caregiver at the time of the child's death in slums	Sierra Leone	Cross-sectional study	Mixed-method	Health care-seeking	Use of traditional medicine; difficultly reaching the health facility; doubts about need for medical care; mistreatment by staff
	Kerai (2019)[45]	Caregiver of children aged 2 months to 5 years in slums	Pakistan	Cross-sectional study	Quantitative	Healthcare-seeking behaviour	Age of child; gender of child; income; education of caretaker; vaccine awareness; breastfeeding awareness; presence of symptoms such as fever, tachypnea, chest indrawing, persistent vomiting, recurrent illness.
	Lungu (2018)[95]	Caregivers of children under 5 years of age in slums	Malawi	Prospective study	Quantitative	Healthcare-seeking behaviour	Cost; waiting time; availability of medicines and supplies; attitude of health workers; thorough examination of the child
						Willingness to pay for the health facility	Waiting time; availability of medicine and equipment; superficial or thorough examination; attitude of health workers
	Kamati (2019)[60]	Slum residents	Namibia	Cross-sectional study	Mixed-method	Self-medication	Perceived diagnosis as "minor or mild"; waiting times and queues to receive care
	Mishra (2017)[56]	Mothers living in slums with a child and	India	Cross-sectional study	Quantitative	Healthcare seeking behaviour	Symptoms and severity

For peer review only

		migrated recently					
	Lungu (2016)[70]	Caregivers and health providers in slums	Malawi	Longitudinal study	Qualitative	Healthcare-seeking behaviour	Home management; lack of medicines and supplies; waiting times; facility opening times; attitude of health workers; suboptimal examination of the sick child; distance to health facility; cost of healthcare
Healthcare for women	Muralidharan (2019)[105]	Girls and mothers in slums	India	Cross-sectional study	Qualitative	Healthcare-seeking behaviour	Proximity of healthcare facilities
	Nasrin (2019)[96]	Married women with a child in slums	Bangladesh	Cross-sectional study	Mixed-method	Healthcare-seeking behaviours	Inability to spend the treatment cost
	Jayaweera (2018)[66]	Girls and women in slums	Kenya	Cross-sectional study	Qualitative	Access to contraception and abortion in health facilities	Stigma; lack of education about safe methods of abortion; perceived illegality of abortion; limited access to services because of financial barrier; fear of mistreatment and mistrust of health providers/facilities; geographical proximity
	Williams (2018)[109]	Mothers and medical personnel in slums	Bangladesh	Cross-sectional study	Qualitative	Mental healthcare seeking	Culture and stigma
	Ilankoo (2018)[65]	Women in slums	Sri Lanka	Cross-sectional study	Qualitative	Health-seeking behaviours related to vaginal discharge	Confusion in differentiating normal from abnormal vaginal discharge; effects on day-to-day life; confusion toward the causative factors; difficulties in disclosing; neglecting behaviours; and socio-cultural influences toward health-seeking behaviours.
	Athie (2017)[110]	Anxious and depressed women in	Brazil	Cross-sectional study	Qualitative	Healthcare seeking behaviour	High medical turnover and overload of healthcare providers

For peer review only

		slums					
	Sudhinaraset (2016)[77]	Mothers and their families in slums	India	Cross-sectional study	Qualitative	Maternal health services and delivery experiences	Financial barriers; disrespectful care
	Pune Municipal corporation[33]	Recently delivered slum residents	India	Cross-sectional study	Mixed-method	Seeking front-line worker during labor	No time to call; family did not allow; being out of town; lack of trust; delivery at night
						Going to the Referred Place for Pregnancy Complications	Not necessary; family did not allow; lack of trust/poor quality services; don't like going to a difference facility; too far; cost; no transportation; private hospital
Preference for healthcare providers	Das (2018)[88]	Slum residents	India	Cross-sectional study	Qualitative	Healthcare-seeking practice (preference for formal/informal healers)	Female prefer informal healers (cultural competency of care, easy communication, gender-induced affordability, avoidance of social stigma and labelling, living with the burden of cultural expectations and geographical and cognitive distance of formal health care)
							Male prefer formal care (ease of access, quality of treatment, expected outcome of therapies)
	Angeli (2018)[100]	Slum residents	India	Cross-sectional study	Mixed-method	Choice between public or private hospital	Bottom-of-the pyramid patients visit a public hospital more than top-of-the-pyramid patients
HIV testing	Thomson (2018)[59]	Stakeholder including residents and healthcare service provider	Kenya	Cross-sectional study	Qualitative	HIV testing	Denial; complacency; fear of death; anticipation of unbearable stress; felt ill; had a partner die; learned that their partner was HIV-positive.
Expenditure	Mishra (2017)[49]	Slum households with a child aged 0–14	India	Cross-sectional study	Quantitative	Treatment-seeking behaviour	Child's gender

For peer review only

	years and who had migrated within the last 12 years	

CVD: cardiovascular disease; HIV: human immunodeficiency virus; NGO: non-governmental organization; TB: tuberculosis.

For peer review only

Supplemental Table 2. Healthcare utilisation of slum residents reported by included studies and associated factors

Subcategory	Author (year)	Participants	Country	Study design	Methodology	Outcome	Factors of interest for this review
General utilisation	Agrawal (2019)[101]	Older adults in slums	India	Cross-sectional study	Quantitative	Utilisation of welfare schemes	Religion; Caste; education;
	Ahmed (2019)[108]	N/A	Bangladesh	Cross-sectional study	Quantitative	Access to, and availability of healthcare services	Variability in traffic congestion
	Madan (2019)[83]	Female slum residents	India	Cross-sectional study	Qualitative	Access to primary care	Long waiting times and opening times of the primary health care; quality of services; satisfaction with treatments; home remedies; cost; rude attitude of healthcare providers
	Owiti (2018)[73]	Pregnant women in slums	Kenya	Cross-sectional study	Quantitative	Utilisation of maternal health services in public health facilities	Perception about public health facility delivery; living within close proximity; waiting time at the facility; learning about the program; quality of service; ANC attendance at a private and a non-profit health facility
	Castiglione (2018)[74]	Slum residents	Brazil	Cross-sectional study	Qualitative	Barrier to healthcare services	*Public healthcare services:* structural aspects of the healthcare system in their community as a whole, such as scarcity of personnel and equipment, or long waiting periods; experiences of conflict when dealing with doctors and other professionals of the public healthcare system

For peer review only

						Private healthcare services: Insufficient funds to seek assistance; services or products in the private sector;
Tabrizi* (2018)[113]	Households in slum and non-slums	Iran	Cross-sectional study	Quantitative	Utilisation of health services	High cost of services
					Home care services	High cost of services
					Not taking drugs prescribed	Slums: financial problems<br><br>Non-slums: getting better/feeling well
Wairiuko (2017)[75]	Elderly in slums	Kenya	Cross-sectional study	Mixed-method	Health service utilisation	Family support; satisfaction with healthcare services; gender healthcare worker preference; services by community health worker
Owusu-Ansah (2016)[69]	Slum residents	Ghana	Cross-sectional study	Qualitative	Utilization of healthcare	Education; occupation; NHIS membership; knowledge of symptom; overall knowledge score; transportation
Adane (2017)[68]	Mothers/caregivers of under-five children in slums	Ethiopia	Cross-sectional study	Quantitative	Utilization of healthcare facilities in children with diarrhoea	Mothers/caregivers education; occupation; time of walking to the nearest health facility; household monthly income; recognized danger signs
MacPherson (2019)[106]	Slum residents	Malawi	Prospective study	Quantitative	Access to TB diagnosis	Distance to the nearest TB registration clinic
Wingfield	Slum households	Peru	Randomized	Quantitative	Initiation of TB	Socioeconomic support and

For peer review only

(2017)[99]	with patients treated for TB		controlled study		preventive therapy	social support
Iberico (2016)[85]	Healthcare workers and community members in slums	Peru	Cross-sectional study	Qualitative	Utilization of TB preventive therapy	Misunderstanding and fear of treatment
Snyder* (2016) [42]	TB patients living in slum and non-slum	Brazil	Retrospective study	Quantitative	Abandonment of TB treatment	Residency in a slum; sex; age; extrapulmonary clinical disease; HIV/AIDS; interaction (directly observed treatment × residency in a slum)
Oluoch (2017)[82]	Slum residents	Nairobi	Cross-sectional study	Quantitative	Attendance to HIV testing and counselling services	Previous test experience
Martinez Perez (2016)[76]	Healthcare workers and community members in slums	South Africa	Cross-sectional study	Mixed method	HIV Counselling and Testing	Fear; lack of trust
Amiresmaili (2019)[18]	Slum residents	India	Cross-sectional study	Quantitative	Utilisation of outpatients services	Gender; marital status
					Utilisation of inpatients services	Age of household head; marital status; insurance
Horng (2019)[38]	Slum households with children under 5 years old who either recently relocated <12 months or who were residentially stable living >24 months	Bangladesh	Cross-sectional study	Quantitative	Healthcare utilisation in severe acute respiratory illness	Relocation; age of child; education of mother; household wealth; health service knowledge
					Full vaccination coverage	Relocation; number of children in household; age of child; education of mother; occupation of household head; household wealth; health service knowledge
Kuria (2018)[111]	Patients received hypertension treatment in slums	Kenya	Retrospective study	Quantitative	Compliance with hypertensive treatment	Health facility group than walkway or weekend clinic attenders

For peer review only

	Cernauskas (2018)[79]	Slum residents	India	Cross-sectional study	Quantitative	Health provider choice	Distance to health facilities; friendly attitude of healthcare workers; appropriate service; familiarity
	Kaba (2020)[61]	Stakeholders (community members, community opinion leaders, health professionals, health office representatives.)	Ethiopia	Cross-sectional study	Qualitative	Utilisation of health services	Individual level: awareness about health problems; competing priorities; capacity to pay for services when referred.
	Mataboge (2016)[112]	Health services' clients and healthcare providers in an informal settlement	South Africa	Cross-sectional study	Qualitative	Healthcare utiliastion	Long waiting time
Immunization	Obanewa (2020)[52]	Rural/urban formal/slum residents	Nigeria	Retrospective cross-sectional study	Quantitative	Fully-immunized child coverage	For slums:<br>delivery place; maternal education; birth order; antenatal attendance; religion<br><br>For slum and non-slums:<br>year; birth order; antenatal attendance; maternal education; religion; maternal age at child's birth; media exposure; region of the country; interaction between place of residence and place of delivery
	Viramgami (2019)[104]	Married slum residents in	India	Cross-sectional	Quantitative	Vaccination status of child	Mother's employment

For peer review only

	reproductive age		study			
Singh (2018)[36]	N/A	India	Literature review	-	Childhood vaccination	Fear of adverse events; lack of information/knowledge; disease not harmful/serious; parents busy; income; mother's education; travel/transfer/migration; unawareness of need for health services; faith in immunization; mother ill; forgetfulness; lack of initiative; family problems; services not available/lack of facility; shortages/reluctant to open 10 dose vials for 1 or 2 infants; current/history of sickness lead to withhold the vaccine
Pugliese-Garcia (2018)[63]	Stakeholders including slum residents, healthcare workers, health committee members, vaccinators	Zambia	Cross-sectional study	Qualitative	Vaccine hesitancy	Traditional remedies; alcohol use; religious beliefs; distrust towards western medicine; previous adverse events; fear of injections and low perceived need for immunisation; limited understanding of how vaccines work; overlapping local terms for vaccine; pain; perceived risk of infection
Manandhar (2018)[80]	Slum household with children age of 12-60 months	Nepal	Cross-sectional study	Quantitative	Incomplete immunisation	Knowledge on immunisation schedule
Dasgupta (2018)[102]	Slum household with children aged	India	Cross-sectional	Quantitative	Vaccine hesitancy	Family type; education of mother

For peer review only

	0-59 months, resides in the study area for the past 12 months		study			
Lae (2018)[37]	Caregivers in slums	Myanmar	Cross-sectional study	Qualitative	Utilisation of immunisation services	Age of child; income; migration; antenatal visit; receiving additional vaccines before; Having immunisation card.
Schultz (2017)[107]	Parents with children <5 years old in slums	Kenya	Prospective study	Quantitative	Timeliness of vaccination	Close to the clinic; birth in December
Crocker-Buque (2017)[21]	People living in a low-income urban area or slum in a low-middle income countries	Multiple nations	Systematic review	-	Immunisation coverage	*Socioeconomic and demographic characteristics:* socioeconomic status; wealth; parents' literacy; mother's education; employment; residential status; place of residence; place of delivery; household visit by health workers; premature birth; malnourishment; inadequate housing; poor prenatal care; ethnicity; age; maternal age; birth order; sex of child; number of children<br><br>*Migration status:* migration; recent migration<br><br>*Information, beliefs and behaviour:* unaware of the need for vaccines; unaware of clinic location or timing; maternal knowledge of

For peer review only

							immunization; lack of access to information; parents being too busy; return to home village; difficulty in accessing services; fear of side effects; attitude of health workers; concerns over cost; being suspicious of free services<br><br>*Health services:*<br>distance from health centre; timing of services; fear of costs; risk of lost income; lack of local knowledge; patients' satisfaction; provision of accurate information; accessing pre-natal care
	Shrestha (2016)[47]	Slum households with children aged 12–23 months.	Nepal	Case-control study	Quantitative	Incompletion of immunisation	Age; birth order; home delivery; education; ethnicity; type of residence; socioeconomic status; knowledge of primary care-taker
	Devasenapathy (2016)[51]	Slum household with children aged between 12 and 42 months	India	Cross-sectional study	Quantitative	Childhood complete immunisation	Sex; mother's literacy; place of birth; place of childbirth; religion; socioeconomic position; birth certificate
Maternal	Razzaque (2020)[34]	Slum residents	Bangladeshi	Cross-sectional study	Quantitative	Healthcare utilisation	Recent migration; wealth; education; employment
	Getachew (2020)[71]	Slum households	Ethiopia	Cross-sectional study	Quantitative	Delivery in healthcare facilities	Perceived as not customary to deliver at health facility; not necessary; unavailability of female birth attendants; perceived

For peer review only

						quality of services; cost
Shrestha (2019)[53]	Mothers with infant residing in slums	Nepal	Cross-sectional study	Quantitative	Utilisation of antenatal and delivery services	Educational status of respondents and their husbands; number of pregnancy
					Institutional delivery	Educational status; occupation of husband; number of pregnancy
					Postnatal visit	Occupation of husband
					Utilisation of family planning services	Occupation of husband
					Tetanus Toxoid immunisation	Educational status of respondents; economic status; knowledge about healthcare services; educational status of husband; number of pregnancies
Atusiimire (2019)[84]	Mothers delivered in the past one year in slums	Uganda	Cross-sectional study	Quantitative	Facility based–deliveries	Exposure to media concerning facility delivery; frequency of ANC; timing of 1st ANC
Upadhyai (2019)[40]	Recently delivered mothers residing in slums	India	Cross-sectional study	Quantitative	Healthcare utilisation	Age; education of mother and father; socioeconomic class; antenatal check-ups; institutional delivery services; family type; caesarean delivery; complication or perceived health problem
Angeles[*] (2019)[41]	Slum and non-slum residents	Bangladesh	Prospective study	Quantitative	Use of modern contraceptive methods	Parity, mother's age; mother's education, socioeconomic status, interaction (slum × time period)
					Delivery by skilled birth attendant	Residing in slums, parity, mother's age, mother's education, length of stay in

For peer review only

						current city of residence, socioeconomic status, number of available community health worker, distance from health facility, interaction (slum x time period)
Kusuma (2018)[67]	Recent migrant and settled mothers with a child up to the age of 1 year in slums	India	Cross-sectional study	Quantitative	Birth in health facility	Listening to radio; number of ANC visits; plan for hospital birth; plan for transport; some danger sign; knowledge of danger sign
Sharma (2018)[114]	Women living in urban slums and delivered a baby within 1 year	India	Cross-sectional study	Quantitative	Utilisation of maternal care services	Mode of delivery; hospital stay after delivery
Islam* (2018) [89]	Ever-married women aged 15-49 years living in slum and non-slum	Bangladesh	Cross-sectional study	Quantitative	ANC visits	Education; wealth index of the household
Geddam (2017)[35]	Rural to urban internal migrant mothers with a child of less than 2 years of age	India	Cross-sectional study	Quantitative	Utilisation of maternal health services	Education of the mother; family size; occupation of mother
					Delivery in institution	Educational status of mother; number of ANC visit; adequacy of ANC; migration status
Kaba (2017)[72]	Stakeholders including city administrators, community members, healthcare providers	Ethiopia	Cross-sectional study	Qualitative	Maternal health service utilisation	Lack of awareness and lack of perceived needs about available services; fear of stigma; competing priorities, social connectedness; perceived lack of respectful service providers; socio-cultural factors including socially sanctioned expectations
Verma	Pregnant	India	Case-control	Mixed-	Antenatal care	Knowledge of healthcare

	(2017)[62]	women and infants in slums		study	method	registration/immunisation	services; perceived need for healthcare services; family support; fear; negative experience with previous vaccination
	Sharma (2016)[48]	Married women in slums	Nepal	Cross-sectional study	Quantitative	Antenatal healthcare utilisation	Age; husband education; spouse occupation; family income; type of family; planned pregnancy; death of children
	Jolly (2016)[92]	Married women with a pregnancy outcome in the previous year in slums	Bangladesh	Cross-sectional study	Quantitative	Antenatal care; birth assisted by medically trained provider; postnatal care; treatment seeking for delivery complications	Education; wealth
						Use of modern family planning	Wealth
	Tebekaw (2016)[103]	Women in slums	Ethiopia	Cross-sectional study	Quantitative	Antenatal care services	Education; private/public hospital
	Sadhna (2016)[93]	Married women in slums	India	Cross-sectional study	Quantitative	Utilisation of maternal health services	Education; Caste; wealth; distance to preferred health facility
	Neyaz (2016)[54]	Married women in slums	India	Cross-sectional study	Quantitative	Delivery in hospitals	Received ANC; number of ANC visits; education; birth order; living index
	Rahman (2016)[91]	Married women in rural and slum area	India	Cross-sectional study	Quantitative	Intrauterine contraceptive device utilisation	Income; occupation
	Sheehy (2016)[94]	Informant and women in slums	Myanmar	Cross-sectional study	Qualitative	Giving birth in hospital	Financial constraints; lack of transportation; sociocultural and financial considerations
Contraceptive	Renzaho (2017)[46]	Slum residents aged 13-24	Uganda	Cross-sectional study	Quantitative	Access to contraceptive services and family planning	Age; disability
	Abd El Fatah	Married women	Egypt	Cross-	Quantitative	Contraceptive use	Number of male children

For peer review only

	(2019)[55]	aged 15–49 years in slums		sectional study			
Health insurance	Otieno (2019)[78]	Slum residents	Kenya	Cross-sectional study	Quantitative	Enrolment in a health insurance programme	Employment; source of primary care; satisfaction with cost of care; satisfaction with procedure of care; perceived health status
	Kusuma (2018)[39]	Slum residents	India	Cross-sectional study	Quantitative	Health insurance possession	Residential background (old slums than new); migration period; possession of ration card; household size; occupation of household head
	Gupta (2017)[81]	Slum households having health insurance cards	India	Cross-sectional study	Mixed-method	Utilisation of healthcare insurance	Awareness of the empanelled hospitals; experiences of friends and relatives at national health insurance empanelled hospitals; hospitals refused to accept health insurance cards
Expenditure	Sahu (2017)[57]	Women delivered within a period of 6 weeks in slums	India	Cross-sectional study	Quantitative	Out-of-pocket expenditure for maternal and neonatal health services	Gravidity; type of delivery; place of delivery; morbidity
	Mishra (2017)[49]	Slum households with a child aged 0–14 years and who had migrated within the last 12 years	India	Cross-sectional study	Quantitative	Out-of-pocket expenditure	Child's gender; mother's education; type of illness

*Factors reported in the study were associated with participants covering both slum and non-slum residents. ANC: antenatal care; CVD: cardiovascular disease; HIV: human immunodeficiency virus; N/A: not applicable; NGO: non-governmental organization; TB: tuberculosis.

For peer review only

Supplemental Table 3. Provision of healthcare services in slums examined by included studies and associated factors

Subcategory	Author (year)	Participants	Country	Study design	Methodology	Outcome	Factors of interest
General provision	Kaba (2020)[61]	Stakeholders (community members, community opinion leaders, Urban Health Extension Professionals, and city health office representatives.)	Ethiopia	Cross-sectional study	Qualitative	Provision of health services	Institutional-level: medical supplies; a lack of passion; attitudes on the part of health service providers<br><br>Community level: shared understanding of the problems; services and the community's established values in relation to the problems and services.
	Das Gupta (2020)[119]	N/A	India	Case study	Mixed-method	Improving public health services	Devolution of service delivery transferring funds and responsibilities to elected local bodies; management by professional managerial and technical cadres; Tight organisation of public health services; Professional support from the state directorate of public health
	Ongarora (2019)[121]	Private healthcare facilities	Kenya	Cross-sectional study	Quantitative	Provision of medicine	Medicine price, affordability and availability of medicine
	Agonigi (2018)[118]	Health professionals	Brazil	Cross-sectional study	Qualitative	Production of care in the daily work of health professionals	Issues related to assignment of tasks; inadequate space and equipment; requirement to follow standardised protocol; demands from the management; workload; environment (sanitation, territory); violence; registration

For peer review only

Odhiambo (2016)[116]	Community health workers	Kenya	Longitudinal study	Quantitative	Drug administration activities for schistosomiasis	Community health worker familiarity with households led to warm reception; good knowledge of intervention area by community health workers; high demand for drugs in the final year of treatment; effective community mobilization; opportunity to integrate mass drug administration with other health interventions; presence of community health workers and their supervisory structure, and points of referral for serious side effects; fear of side effects, size of tablet and misconceptions regarding treatment; unrelated death and the associated negative publicity by the media; religious beliefs and mistrust of interventions; insufficient time; absence of community members during the drug administration exercise; difficulty in directly observing treatment; unsanitary environmental conditions; inaccessibility (filthy and bush environment); demand for incentives by community members to take drugs.
Patil (2016)[117]	Healthcare service centres	India	Cross-sectional study	Quantitative	Services provided under Integrated Child	Lack of basic infrastructural facilities; absence of essential drugs, equipment and

For peer review only

						Development Services	logistics; poor pay scale, untimely drug supply, poor community support, more of documentation work, increased work burden, lack of supportive staff and no incentives for the increased work
	Mataboge (2016)[112]	Health services' clients and healthcare providers in an informal settlement	South Africa	Cross-sectional study	Qualitative	Provision of reproductive healthcare services	Healthcare policies; work overload; community-based care
	Prado Junior (2016)[120]	New TB cases living in slum and non-slum	Brazil	Cross-sectional study	Quantitative	Coverage under Family Health system for TB patients	Policy prioritizing low social development areas

TB: tuberculosis

For peer review only

Preferred Reporting Items for Systematic reviews and Meta-Analyses extension for Scoping Reviews (PRISMA-ScR) Checklist

SECTION	ITEM	PRISMA-ScR CHECKLIST ITEM	REPORTED ON PAGE #
**TITLE**			
Title	1	Identify the report as a scoping review.	1
**ABSTRACT**			
Structured summary	2	Provide a structured summary that includes (as applicable): background, objectives, eligibility criteria, sources of evidence, charting methods, results, and conclusions that relate to the review questions and objectives.	2
**INTRODUCTION**			
Rationale	3	Describe the rationale for the review in the context of what is already known. Explain why the review questions/objectives lend themselves to a scoping review approach.	4
Objectives	4	Provide an explicit statement of the questions and objectives being addressed with reference to their key elements (e.g., population or participants, concepts, and context) or other relevant key elements used to conceptualize the review questions and/or objectives.	5
**METHODS**			
Protocol and registration	5	Indicate whether a review protocol exists; state if and where it can be accessed (e.g., a Web address); and if available, provide registration information, including the registration number.	5
Eligibility criteria	6	Specify characteristics of the sources of evidence used as eligibility criteria (e.g., years considered, language, and publication status), and provide a rationale.	6-7
Information sources*	7	Describe all information sources in the search (e.g., databases with dates of coverage and contact with authors to identify additional sources), as well as the date the most recent search was executed.	5-6
Search	8	Present the full electronic search strategy for at least 1 database, including any limits used, such that it could be repeated.	5, Supplement 3
Selection of sources of evidence†	9	State the process for selecting sources of evidence (i.e., screening and eligibility) included in the scoping review.	5-6
Data charting process‡	10	Describe the methods of charting data from the included sources of evidence (e.g., calibrated forms or forms that have been tested by the team before their use, and whether data charting was done independently or in duplicate) and any processes for obtaining and confirming data from investigators.	6
Data items	11	List and define all variables for which data were sought and any assumptions and simplifications made.	7-9
Critical appraisal of individual sources of evidence§	12	If done, provide a rationale for conducting a critical appraisal of included sources of evidence; describe the methods used and how this information was used in any data synthesis (if appropriate).	N/A

St. Michael's
Inspired Care. Inspiring Science.

For peer review only - http://bmjopen.bmj.com/site/about/guidelines.xhtml

SECTION	ITEM	PRISMA-ScR CHECKLIST ITEM	REPORTED ON PAGE #
Synthesis of results	13	Describe the methods of handling and summarizing the data that were charted.	7-9
**RESULTS**			
Selection of sources of evidence	14	Give numbers of sources of evidence screened, assessed for eligibility, and included in the review, with reasons for exclusions at each stage, ideally using a flow diagram.	9, Figure 1
Characteristics of sources of evidence	15	For each source of evidence, present characteristics for which data were charted and provide the citations.	Supplement 1-2
Critical appraisal within sources of evidence	16	If done, present data on critical appraisal of included sources of evidence (see item 12).	N/A
Results of individual sources of evidence	17	For each included source of evidence, present the relevant data that were charted that relate to the review questions and objectives.	Table 2-3
Synthesis of results	18	Summarize and/or present the charting results as they relate to the review questions and objectives.	Figure 2
**DISCUSSION**			
Summary of evidence	19	Summarize the main results (including an overview of concepts, themes, and types of evidence available), link to the review questions and objectives, and consider the relevance to key groups.	25-26
Limitations	20	Discuss the limitations of the scoping review process.	27
Conclusions	21	Provide a general interpretation of the results with respect to the review questions and objectives, as well as potential implications and/or next steps.	29
**FUNDING**			
Funding	22	Describe sources of funding for the included sources of evidence, as well as sources of funding for the scoping review. Describe the role of the funders of the scoping review.	31

JBI = Joanna Briggs Institute; PRISMA-ScR = Preferred Reporting Items for Systematic reviews and Meta-Analyses extension for Scoping Reviews.

* Where *sources of evidence* (see second footnote) are compiled from, such as bibliographic databases, social media platforms, and Web sites.

† A more inclusive/heterogeneous term used to account for the different types of evidence or data sources (e.g., quantitative and/or qualitative research, expert opinion, and policy documents) that may be eligible in a scoping review as opposed to only studies. This is not to be confused with *information sources* (see first footnote).

‡ The frameworks by Arksey and O'Malley (6) and Levac and colleagues (7) and the JBI guidance (4, 5) refer to the process of data extraction in a scoping review as data charting.

§ The process of systematically examining research evidence to assess its validity, results, and relevance before using it to inform a decision. This term is used for items 12 and 19 instead of "risk of bias" (which is more applicable to systematic reviews of interventions) to include and acknowledge the various sources of evidence that may be used in a scoping review (e.g., quantitative and/or qualitative research, expert opinion, and policy document).

From: Tricco AC, Lillie E, Zarin W, O'Brien KK, Colquhoun H, Levac D, et al. PRISMA Extension for Scoping Reviews (PRISMAScR): Checklist and Explanation. Ann Intern Med. 2018;169:467–473. doi: 10.7326/M18-0850.

For peer review only - http://bmjopen.bmj.com/site/about/guidelines.xhtml

Factors associated with accessing and utilisation of health care and provision of health services for residents of slums in low and middle income countries: a scoping review of recent literature

Journal:	*BMJ Open*
Manuscript ID	bmjopen-2021-055415.R1
Article Type:	Original research
Date Submitted by the Author:	14-Jan-2022
Complete List of Authors:	Park, Ji-Eun; University of Warwick, Warwick Medical School; Korea Institute of Oriental Medicine<br>Kibe, Peter; African Population and Health Research Center, Health and Systems for Health<br>Yeboah, Godwin; University of Warwick, Institute for Global Sustainable Development<br>Oyebode, Oyinlola; University of Warwick Warwick Medical School,<br>Harris, Bronwyn; University of Warwick, NIHR Global Health Research Unit on Improving Health in Slums<br>Ajisola, Motunrayo ; University of Ibadan, Sociology<br>Griffiths, Frances; University of Warwick Warwick Medical School, ; University of the Witwatersrand, Centre for Health Policy<br>Aujla, Navneet; Newcastle University, Population Health Sciences Institute; University of Warwick, Division of Health Sciences<br>Gill, Paramjit ; University of Warwick<br>Lilford, RJ; University of Birmingham, Institute of Applied Health Research<br>Chen, Yen-Fu; University of Warwick, Division of Health Sciences
**Primary Subject Heading**:	Global health
Secondary Subject Heading:	Epidemiology, General practice / Family practice, Health policy, Health services research, Public health
Keywords:	HEALTH SERVICES ADMINISTRATION & MANAGEMENT, International health services < HEALTH SERVICES ADMINISTRATION & MANAGEMENT, PUBLIC HEALTH

For peer review only - http://bmjopen.bmj.com/site/about/guidelines.xhtml

I, the Submitting Author has the right to grant and does grant on behalf of all authors of the Work (as defined in the below author licence), an exclusive licence and/or a non-exclusive licence for contributions from authors who are: i) UK Crown employees; ii) where BMJ has agreed a CC-BY licence shall apply, and/or iii) in accordance with the terms applicable for US Federal Government officers or employees acting as part of their official duties; on a worldwide, perpetual, irrevocable, royalty-free basis to BMJ Publishing Group Ltd ("BMJ") its licensees and where the relevant Journal is co-owned by BMJ to the co-owners of the Journal, to publish the Work in this journal and any other BMJ products and to exploit all rights, as set out in our licence.

The Submitting Author accepts and understands that any supply made under these terms is made by BMJ to the Submitting Author unless you are acting as an employee on behalf of your employer or a postgraduate student of an affiliated institution which is paying any applicable article publishing charge ("APC") for Open Access articles. Where the Submitting Author wishes to make the Work available on an Open Access basis (and intends to pay the relevant APC), the terms of reuse of such Open Access shall be governed by a Creative Commons licence – details of these licences and which Creative Commons licence will apply to this Work are set out in our licence referred to above.

Other than as permitted in any relevant BMJ Author's Self Archiving Policies, I confirm this Work has not been accepted for publication elsewhere, is not being considered for publication elsewhere and does not duplicate material already published. I confirm all authors consent to publication of this Work and authorise the granting of this licence.

For peer review only

Factors associated with accessing and utilisation of health care and provision of health services for residents of slums in low and middle income countries: a scoping review of recent literature

Ji-Eun Park,[1,2] Peter Kibe,[3] Godwin Yeboah,[4] Oyinlola Oyebode,[1] Bronwyn Harris,[1] Motunrayo Ajisola,[5] Frances Griffiths,[1,6] Navneet Aujla,[1,7] Paramjit Gill,[1] Richard Lilford,[8] Yen-Fu Chen,[1]* on behalf of the Improving Health in Slums Collaborative

Affiliations:

[1] Division of Health Sciences, Warwick Medical School, University of Warwick, Coventry, United Kingdom

[2] Future Medicine Division, Korea Institute of Oriental Medicine, Daejeon, Republic of Korea

[3] African Population and Health Research Center, Nairobi, Kenya

[4] Institute for Global Sustainable Development, University of Warwick, Coventry, United Kingdom

[5] College of Medicine, University of Ibadan, Ibadan, Nigeria

[6] Centre for Health Policy, School of Public Health, University of the Witwatersrand, Johannesburg, South Africa

[7] Population Health Sciences Institute, Newcastle University, Newcastle, United Kingdom

[8] Institute of Applied Health Research, University of Birmingham, Birmingham, United Kingdom

*Corresponding author: Dr Yen-Fu Chen, Division of Health Sciences, Warwick Medical School, University of Warwick, Coventry, CV4 7AL, UK.

Email: Y-F.Chen@warwick.ac.uk

Word count: **6,083**

For peer review only - http://bmjopen.bmj.com/site/about/guidelines.xhtml

ABSTRACT

Objective: To identify factors associated with accessing and utilisation of healthcare and provision of health services in slums.

Design: A scoping review incorporating a conceptual framework **for configuring reported factors.**

Data sources: MEDLINE, Embase, CINAHL, Web of Science and the Cochrane Library were searched **from their inception to December 2021** using slum related terms.

Eligibility criteria: Empirical studies of all designs reporting relevant factors in slums in low and middle income countries.

Data extraction and synthesis: Studies were categorised and data were charted according **to** a preliminary conceptual framework refined by emerging findings. Results were tabulated and narratively summarised.

Results: Of the **15,091** records retrieved from all years, **4,368** records dated between **2016-2021** were screened by two independent reviewers and **111** studies were included. The majority (**63 studies, 57%)** were conducted in Asia, predominantly in India. In total **104** studies examined healthcare access and utilisation from slum residents' perspective while only **ten** studies explored provision of health services from providers/planners' perspective (**three** study included both). A multitude of factors are associated with accessing, utilising and providing healthcare in slums, including recent migration to slums; knowledge, perception and past experience of illness, healthcare needs and health services; financial constraint and competing priorities between health and making a living; lacking social support; unfavourable physical environment and locality; sociocultural expectations and stigma; lack of official recognition; and problems in existing health system.

Conclusion: The scoping review identified a significant body of recent literature reporting

For peer review only - http://bmjopen.bmj.com/site/about/guidelines.xhtml

factors associated with accessing, utilisation and provision of healthcare services in slums. We classified the diverse factors under seven broad categories. The findings can inform a holistic approach to improving health services in slums by tackling barriers at different levels, taking into account local context and geospatial features of individual slums.

Systematic review registration: Open Science Framework (OSF, https://osf.io/694t2)

Keywords: slum, informal settlement, scoping review, healthcare-seeking behaviour, healthcare utilisation, health service delivery

Strengths and limitations of this study

- We conducted literature search in multiple databases using generic terms related to slums to ensure that a wide range of relevant studies was captured.
- A conceptual framework explaining factors associated with accessing and utilisation of healthcare by slum residents as well as provision of healthcare in slums was developed and used to categorise identified studies and factors.
- We examined barriers and facilitators of accessing healthcare and service provision from the perspectives of both demand side (slum residents) and supply side (healthcare providers and service planners).
- Only studies published **in academic journals** between **2016 and 2021** in English language were included, and methodological quality of each included study was not examined because of time constraint.
- We did not explore the complex relationships and interactions between various factors **in different contexts** at different slum locations, but our mapping of these factors to the conceptual framework should facilitate further in-depth analyses.

For peer review only - http://bmjopen.bmj.com/site/about/guidelines.xhtml

INTRODUCTION

Rapid urbanisation has resulted in a growing number of residents in slums[1] who face ongoing problems such as unemployment, poor sanitation, lack of transport, high level of crime, and haphazard development.[2] In 2018, over one billion people were living in slum-like conditions, and Central, South and South-East Asia and Sub-Saharan Africa accounted for 80% of them.[1] Even though various definitions of slums exist, there is no universally agreed definition of what constitutes 'a slum', and the term itself is widely debated and contested.[3 4] For the purpose of this scoping review, we refer to slums as densely populated areas characterised by lack of basic services, substandard housing, overcrowding, unhealthy living condition, insecure tenure and poverty,[4 5] taking into account the crucial concepts of place and space that are important in shaping health outcomes and community access to health services in these urban settings.[4]

Previous studies have reported various risk factors affecting health of slum residents such as physical environment,[6] sanitation,[7] social capital[8 9] and water governance,[10] and have observed in some cases that slum residents have worse health status compared to non-slum urban and/or rural residents. For example, Ezeh et al. found that children living in slums had higher mortality than rural and non-slum urban populations.[3] Poorer height-for-age for children[11] and higher prevalence of childhood illnesses and malnutrition[12] have also been observed in slums compared to non-slum urban and rural settings. In addition, slum residents are susceptible to unhealthy behaviours.[13 14] Living in slums has been found to be associated with low physical activity,[13] poor diet,[14] and poor knowledge about the cause and preventability of diseases.[15]

Despite the unfavourable health status and environment, and consequently the potential high level of healthcare needs, previous studies showed that slum residents were less likely to seek and use healthcare services than their non-slum counterparts in the cities.[16 17] Slum residents have been found to have lower rates of healthcare utilisation in antenatal services[16] and services

For peer review only - http://bmjopen.bmj.com/site/about/guidelines.xhtml

for non-communicable diseases[17] compared to residents of urban 'formal' settings. One study in Iran showed that only about half of slum households that required outpatient services could use them.[18] Another study in Haiti also reported that one third of slum households were not able to access medical care for their children when it was needed in the past year.[19]

While the health status and needs of slum residents have been described in previous reviews,[3, 20] factors associated with healthcare seeking behaviour and healthcare utilisation of slum residents and factors related to the provision of health services in slums have not been systematically examined (with the exception of immunisation services).[21] This scoping review aims to fill in these evidence gaps and inform efforts to improve healthcare delivery to people in slums.

METHODS

This scoping review was performed according to current best practice guidance.[22] The broad question of interest was: "*What factors are associated with slum residents'* ***accessing and utilisation*** *of health care and/or the provision of health services in slum settings in low and middle income countries (LMICs)*?" The protocol for this review was registered in Open Science Framework (OSF).[23]

Literature search and study selection

A broad search of five databases (MEDLINE, Embase, CINAHL, Web of Science and the Cochrane Library) was conducted in April 2020 **and updated in December 2021**. Searches were limited to English language. Key terms related to slums were used: slum or slums or

For peer review only - http://bmjopen.bmj.com/site/about/guidelines.xhtml

ghetto or ghettos or informal settlement$ or shantytown$ or shanty town$ or favela$. **(Appendix 1)** We did not include terms related to other concepts in order to maximise the sensitivity of our searches. **In addition, we searched organizational websites of Slum Dwellers International, UN HABITAT, UN and WHO but did not identify relevant studies.**[24-27]

Records retrieved from databases (after duplicates were removed) were initially screened by one reviewer (JEP) and those which did not meet the inclusion criteria were disregarded. After that, a second reviewer (PK, GY, OO) examined the remaining records independently based on titles and abstracts. When the decisions of two reviewers differed, the discrepancy was resolved based on full-texts and/or by discussion with a third reviewer (YFC) or the broader review team. This study screening process started from records of the most recent years (i.e. in the past three years) and then proceeded to prior years. Due to the larger than expected volume of the literature, we eventually screened records **between 2016 to 2021** and did not cover earlier records in order to synthesise and present the findings from latest evidence in a timely fashion to inform the wider project hosting this review.[28 29]

Inclusion and exclusion criteria

A study was included when it: (1) described factors related to slum residents' accessing or utilisation of health care or the provision of health services in slums; and (2) was conducted in relation to slums in LMICs. Only articles written in English were included. A study was excluded when it was a commentary, opinion, or narrative review; described slum residents' utilisation of health services or the provision of health services without exploring the associated factors; investigated informal care at home; or included mixed slum and non-slum populations without separately reporting data for slum residents or investigating residency in slums as a

For peer review only - http://bmjopen.bmj.com/site/about/guidelines.xhtml

factor for healthcare access.

During our updated search in December 2021, we found several studies reporting healthcare utilisation[28] and provision related to COVID-19 in slums.[30] These studies were not included in this scoping review, since the factors associated with healthcare utilisation and health service provision under the pandemic situation are dramatically different and warrant a separate synthesis.

We included both primary studies and systematic reviews that examine data collected empirically and that derive their findings based on the data. Both quantitative and qualitative studies (and by extension, mixed methods studies) were considered. Even though slums have existed in both high-income countries and LMICs, the context may be quite different between these countries. For example, while all slums are vulnerable to natural disasters such as tropical cyclones, the impact of these could be far more severe in slums of LMICs due to the different socioeconomic contexts.[31] In this review, we focused on settings in LMICs and excluded studies conducted in high-income countries.

Study coding and data extraction/charting

Eligible studies were coded and data-extracted/charted according to a pre-specified, preliminary framework shown in Figure 1 below. The preliminary framework was developed by the review authors based on existing conceptual models related to healthcare access and service delivery[32-35] and was modified during the scoping review process to accommodate new factors/themes identified from the literature. **The refined conceptual framework is shown in Figure 2.**

Based on the refined conceptual framework, each eligible study was coded as being associated with one or more of the three phenomena of interest, namely slum residents'

For peer review only

healthcare accessing (which covered perception of needs/desire for care, healthcare seeking and healthcare reaching as defined by Levesque et al[34]), health care utilisation and provision of health services (which covered various arrangements related to service delivery) in slum settings. (Figure 2)

In addition, to facilitate the organisation of complex evidence in this review, diverse factors reported in the included studies were initially classified into six different categories according to the preliminary framework shown in Figure 1. However, during the data charting process, we realised that many factors such as perception of symptoms and experience from past use of healthcare services did not fit into one of these six original categories. A new category of 'cognitive and experiential factors' was therefore added to the refined conceptual framework (Figure 2) to reflect the emerging themes, which include seven categories:

(1) Personal and biological factors: these relate to personal characteristics of slum residents, including age, sex, ethnicity and the nature and severity of health conditions.

(2) Cognitive and experiential factors: these relate to personal awareness, knowledge, perception, attitude, belief and experience etc. formed through cognitive process based on upbringing and past events.

(3) Socioeconomic factors: these include income and wealth, economic hardship/poverty and economic opportunities, marital status, education, crime, social capital (such as bonding, trust and reciprocity between close relatives, neighbours and community members),[36] use of technologies for social and economic purposes, commercial and charitable organisations and activities.

(4) Physical environment: this covers natural environment such as proximity to a health facility, built environment and infrastructure such as water supply, transport and

For peer review only - http://bmjopen.bmj.com/site/about/guidelines.xhtml

mobile/internet networks, as well as weather conditions and environmental pollutions.

(5) Cultural and religious factors: these include cultural and religious beliefs and activities, and local and national customs.

(6) Legal, political and policy factors: these include government policies and issues related to legal, justice and political systems.

(7) Health system factors: these relate to historical and current organisation and provision of health care that may impact upon provision and delivery of health services in individual slum communities and the services experienced by slum residents.

In addition to the addition of the 'cognitive and experiential factors' category, another major difference between the preliminary (Figure 1) and refined (Figure 2) conceptual framework relates to the definition of healthcare access. Our preliminary framework adopted the definition by Levesque and colleagues, who defined healthcare access as "the possibility to identify healthcare needs, to seek healthcare services, to reach the healthcare resources, to obtain or use health care services, and to actually be offered services appropriate to the needs for care."[34] However, during our study screening and data charting process, we found that it would be helpful to make a distinction between the process of 'accessing' healthcare (which covers gaining awareness of needs, forming an intention to seek healthcare and taking an action to reach healthcare) and the actual receipt and utilisation of health care ('accessed care') when examining empirical evidence, as healthcare needs could only be met when the latter occurs and this not only depends on factors related to service users (demand side) but also relies on factors related to service providers/planners (supply side). Therefore we separated out utilisation of health care from 'accessing health care' to highlight that it requires a match between demand and supply side factors.

For peer review only - http://bmjopen.bmj.com/site/about/guidelines.xhtml

Data on study population, study design, country in which the study was conducted, methodology, and associated factors were extracted using a data-charting spreadsheet which was developed and continuously updated as the review progressed by two of the reviewers (JEP and YFC). Whether a study was conducted exclusively within slums and whether a comparison was made between slum and non-slum urban or rural residents were also noted. Coding of phenomena and factors and data-charting were conducted by one reviewer (JEP) and checked by a second reviewer (PK, GY, OO, YFC). Disagreements were discussed between reviewers until consensus was reached.

Patient and public involvement

Given the focus of this scoping review on published literature, we did not directly involve residents and service providers/planners from slum settings. Nevertheless, our wider project has a work package that specifically engages with slum residents and service providers and planners,[28] and early plans and findings of this review were shared with the wider project team who provided comments based on their experiences of community engagement.

RESULTS

The reporting of this review follows the PRISMA Extension for Scoping Reviews (PRISMA-ScR).[37] Using the search strategy described earlier, a total of **15,091 records were retrieved from the initial and updated searches (Medline 4668, Embase 5090, Web of Science 3553, Cochrane 381, CINAHL 1575),** with **9,916** records remaining after excluding duplicates. Two additional articles[18 38] were identified from references of the included studies. As described earlier, screening was limited to the **4,368** records published from 2016 onwards.

For peer review only - http://bmjopen.bmj.com/site/about/guidelines.xhtml

A total of **111** articles were included in this scoping review. (Figure 3) **Thirty-two** studies reported factors associated with healthcare accessing of slum residents, **73** studies reported factors related to healthcare service utilisation, and **10** articles reported the factors related to provision of healthcare services in slums (**four** studies reported factors related to more than one phenomenon of interest). **Seventy-four of the 111 studies were quantitative studies, 21 studies were qualitative studies, and 14 studies were undertaken using mixed-methods**. The remaining two studies were systematic reviews. A total of **42 (38%) studies were conducted in India, followed by Kenya (14 studies, 13%)**. (Table 1)

For peer review only

For peer review only - http://bmjopen.bmj.com/site/about/guidelines.xhtml

Table 1. Characteristics of included studies.

Category	Subcategory		Number of studies (%)	
Publication year	2016		22	(20)
	2017		17	(15)
	2018		23	(21)
	2019		22	(20)
	**2020**		**18**	**(16)**
	**2021**		**9**	**(8)**
Analysis method	**Quantitative**		**74**	**(67)**
	**Qualitative**		**21**	**(19)**
	**Mixed-methods**		**14**	**(13)**
	Narrative synthesis		2	(2)
Study location	Asia	**India**	**42**	**(38)**
		**Bangladesh**	**9**	**(8)**
		**Nepal**	**4**	**(4)**
		**Pakistan**	**3**	**(3)**
		**Myanmar**	**2**	**(2)**
		**Iran**	**2**	**(2)**
		**Sri Lanka**	**1**	**(1)**
	South America	**Brazil**	**7**	**(6)**
		**Peru**	**2**	**(2)**
	Africa	**Kenya**	**14**	**(13)**
		**Ethiopia**	**7**	**(6)**
		**Malawi**	**4**	**(4)**
		**Uganda**	**3**	**(3)**
		**South Africa**	**2**	**(2)**
		**Sierra Leone**	**1**	**(1)**
		**Nigeria**	**1**	**(1)**
		**Egypt**	**1**	**(1)**
		**Zambia**	**1**	**(1)**
		**Namibia**	**1**	**(1)**
		**Ghana**	**1**	**(1)**
	North America	Haiti	1	(1)
	Multiple nations		1	(1)
Healthcare services in slums*	Healthcare accessing		**32**	
	Healthcare service utilisation		**73**	
	Provision of healthcare services		**10**	
Total			**111**	(100)

* One study reported factors related to both healthcare accessing and healthcare utilisation and **three** studies reported factors related to both healthcare utilisation and provision of healthcare services

For peer review only

For peer review only - http://bmjopen.bmj.com/site/about/guidelines.xhtml

Participants, country, study design, methodology, observed phenomena and outcomes, and factors of interests for each study are described in supplemental tables 1-3. Supplemental table 1 shows **32** studies reporting factors associated with general healthcare seeking behaviours; healthcare seeking for children or women; slum residents' preference for healthcare providers; and healthcare seeking related to HIV testing. Supplemental table 2 presents various factors reported in **73** studies related to general healthcare utilisation as well as use of specific services such as childhood immunisation, maternal healthcare, and possession of health insurance. In Supplemental table 3, **ten** studies reporting factors related to the provision of health services in slums are summarised. Key findings are described below.

Demand side: Factors associated with healthcare accessing and healthcare utilisation of slum residents

We found **104** articles which identified many different factors affecting healthcare accessing and utilisation. These factors are often inter-related and exert their influence at different levels (e.g. from personal, family to community level) in different circumstances. We classified various factors into seven categories (Figure 2). Factors particularly relevant to slum settings and other commonly identified factors within each category are highlighted below.

Personal and biological factors: The common factors associated with healthcare **accessing** and utilisation included intrinsic factors such as **age**[21 39-56], **sex**[18 21 41 46 52 54 56-59]**,** and ethnicity,[21] familial factors such as birth order of the sick child,[21 47 60-62] as well as personal health and **type of illness**[46 59]**, disability**[48] **and morbidity**[21 52 63 64] and the specific features of the health condition.[53 56 65] **Slum residents are more like to seek healthcare services when sick children are younger,**[49 50 53 56] **but evidence on the association between mother's age and**

For peer review only - http://bmjopen.bmj.com/site/about/guidelines.xhtml

child's vaccination was inconsistent.[21 39] Healthcare seeking and utilisation were different by sex, but the association was context dependent. Several studies reported higher healthcare utilisation among female slum dwellers,[18 52 56 58] while other studies showed male children had higher vaccination coverage[57] and incurred more medical expenditure.[59] Major life events such as recent migration[21 50 66-68] and relocation[49] into slums tend to be associated with lower healthcare seeking and utilisation. Recency of migration to slums was also related to lower uptake of Rashtriya Swasthya Bima Yojana (RSBY), a national health insurance programme run by the Indian government for poor families.[69] People with specific symptoms (such as fever, tachypnea, persistent vomiting),[53 56 65] disability[48] and illnesses including chronic disease[21 52 54 63 64] tend to use healthcare services more. Although people with tobacco habit were less likely to participate in breast cancer screening, they were more likely to take part when they had family history of cancer or history of cancer screening.[70] Lower birth order of the child was associated with increased utilisation of hospitals for childbirth,[21 47 60-62] while the use of family planning service[47] and out-of-pocket expenditure was higher in multigravida than primigravida.[63]

Cognitive and experiential factors: these factors were not included in our initial conceptual framework but rather emerged inductively from our data. Consequently, their identification led us to revise the conceptual framework for this scoping review. A wide range of factors formed through cognitive processes and influenced by individual's upbringing, past experience and surrounding environment were reported to be associated with both healthcare-seeking and healthcare utilisation of slum residents. Perception,[39 51 53 68 71-77] knowledge[53 78-83] and experience of symptoms and illnesses[51] were commonly found to influence healthcare seeking

For peer review only - http://bmjopen.bmj.com/site/about/guidelines.xhtml

and utilisation. **Mothers who experienced child death and subsequently planned pregnancy showed higher use of antenatal healthcare services.[51] When people perceived the symptom or disease to be serious they tend to seek healthcare services.[53 71 73 77] Although lack of knowledge could be a barrier to accessing healthcare services,[78 79] one study showed caregivers with good knowledge of child danger signs were less likely to seek healthcare services timely.[53] People perceiving their health status as good showed lower odds of having insurance,[84] but awareness and knowledge of health problems lead people to use healthcare services.[39 74 76 80 81 83] Home remedy and home management delayed healthcare seeking behavior.** [53 71 82 85] In addition, perception,[21 42 64 76 84 86-90] knowledge,[21 42 49 61 68 75 82 86 91-96] and experience of healthcare services[39 50 60 62 67 70 75 80 87 97 98] including fear and distrust of healthcare services,[21 38 68 72 75 76 79 89 99-101] and preference related to care provider's gender[88 102] were frequently cited factors. Provider shopping associated with distrust of healthcare providers and denial of diagnosis delayed first care seeking and treatment initiation of pulmonary tuberculosis patients in India.[71] Perception or experience of healthcare services also affected uptake or renewal of health insurance.[84 95]

Socioeconomic factors: **Socioeconomic status was associated with utilisation of healthcare services,[21 39 40 47 57 62 82 103] and even though one study showed that slum residents of lower socioeconomic class were more likely to enrol in health insurance than slum residents of higher socioeconomic class, [40] the latter were more likely to use healthcare services.[39 47 57 62] One study reported higher public hospital visits (compared with private hospital visits) among lower socioeconomic status.[104] Income and wealth[21 41 49-51 61 66 68 81 105-109] including financial constraint[19 21 38 46 74 77 79 85 87 90 101 103 110-114] featured prominently. Higher education level [39 49 56-59 61 62 66-68 70 81-83 107-109 115-117] and higher income**

For peer review only - http://bmjopen.bmj.com/site/about/guidelines.xhtml

[21 49-51 56 58 61 66 68 81 105 107-109 118] were associated with more seeking and utilisation of healthcare services. With some exceptions,[67 105] previous studies reported that employed slum residents tend to seek and use healthcare services more frequently than unemployed slum residents and housewives.[49 51 66 67 70 81 83 84 119] Even though married people tend to seek and use more healthcare services,[18 70] the reported influence of family type was inconsisent.[39 51 116] Female slum residents in nuclear family used more antenatal services than those in joint family type,[51] but female in joint family type used more postnatal service[39] and immunisation service for their children.[116] Smaller family size used more maternal healthcare services,[67] and bigger households had higher odds of having health insurance.[69] The socioeconomic challenges faced by slum residents also manifested as competing priorities [74 94 120] and lack of time[21 101 121] for healthcare-seeking and utilisation, because they did not want to or could not afford to miss work and lose income,[21 71] **which can be** exacerbated by lack of social support.[75 77 88 94 100 122]

Physical environment: Slum residents considered proximity of healthcare facilities,[21 38 47 77 81 85 86 96 109 123-127] transport such as travel assistance,[77] lack of transportation,[38 83 103] traffic congestion,[128] and environment of residence area when they sought and used healthcare services. **Long distance from health facility,[38 55 77 85 102 123] no transportation or travel assistance,[38 83 103] unsafe environment of residential area such as darkness at night were reported as barriers to reaching healthcare facilities.[100]**

Cultural and religious factors: these included religion;**[41 57 60 70 76 115 129]** sociocultural influence[94 102] such as exposure to media[80 98]; stigma associated with unplanned/extramarital pregnancy[79 94] postpartum depression[130] and other illnesses such as contagious skin disease,

For peer review only - http://bmjopen.bmj.com/site/about/guidelines.xhtml

barrenness and female sexually-related problems;[102] and use of traditional/home medicine.[76 87 100] Women in slums could not go to hospital because they had difficulties in disclosing the symptoms, postponed their health issues because of their responsibilities at home, and engaged in self-treatment practices such as home remedies recommended by grandmother and friends because of socio-cultural influences toward healthcare-seeking behaviour.[78] Women in Ethiopia reported not returning to postnatal care due to religious and cultural expectation for mother and baby to stay home for 80 days after birth.[94] One Indian survey showed that some women could not seek healthcare services during labour since their husband or family did not allow that.[38]

Legal, **political and policy factors**: type of slums (in terms of official recognition and availability of basic facilities) and possession of a ration card were found to be associated with uptake of the Indian RSBY national health insurance programme.[69] One study reported that slum residents could not seek healthcare facilities for abortion because of the perceived illegality of abortion.[79]

Health system factors: slum residents were also influenced by many factors related to health systems when they sought healthcare. These included accessibility associated with the location[21 102] and timing of services;[21 85 87] quality of healthcare services[38 67 86 87 102 113 125] such as delay in advising patients to go for related tests or referral,[71] likelihood of receiving appropriate examination,[85 110] and adverse events.[76] Slum residents considered service organisation including medical turnover,[131] availability of supplies/healthcare workers,[47 85 110 113] attitude of healthcare providers,[87] type of healthcare facilities,[38 86 117 132] and waiting time.[73 85-87 110 112 133] **Slum residents tend to seek government and non-governmental organisation**

For peer review only - http://bmjopen.bmj.com/site/about/guidelines.xhtml

(NGO) facility[118] and avoid private hospitals[38] for healthcare services. Healthcare utilisation was higher among slum residents with healthcare insurance than those without it,[18 83] and households with higher quarterly out-of-pocket healthcare expenditure had lower scores for an index of access to primary health care.[135] In an Ethiopian study, some participants reported unavailability of female birth attendants as a reason for not delivering at healthcare facilities.[113] (Table 2)

For peer review only

Table 2. Factors associated with healthcare accessing and healthcare utilisation in slums **from service user's (demand side) perspective.**

Factors	Healthcare accessing	Healthcare utilisation
Personal and biological factors		
Age	**(-) Age[53-56]**	**(±) Age[21 39-52]**; **(+)** age of household head[18]
Gender	**(±) Sex[54 56 58 59]**	**(±) Sex[18 21 41 46 52 57 59]; (male) sex of household head[134]**
Ethnicity		Ethnicity[21]
Migration		**(-)** Recent migration[21 50 66-69]; **(-)** relocation[49]; **(-)** return to home village[21]
Biological	**(+)** Symptoms such as fever, tachypnea, chest in drawing, persistent vomiting[53 56 65]**; having disease[54]**	**Type of illness[46 59]; (+)** having a disability[48]; **(+) morbidity[21 52 63 64]**
Other personal	**(-)** Tobacco habits [70]; **(+)** family history of cancer and history of cancer screening [70]	**(-)** Birth order of sick child [21 47 60-62]; **(-) parity [42 47 63 135]**
Cognitive and experiential factors		
Knowledge/experience of symptoms and illnesses	**(+)** Perception of symptoms[71] or illness[53 73 77]; **(±)** knowledge of symptom/disease[53 78 79]**; (-)** denial and complacency[72]	**(+)** Experience of child death[51]; **(+)** planned pregnancy[51]; **(+)** perceived health status[84] and health problem[39 74 76]; **(+)** knowledge of symptom[83]; disease[80 81]
Ability/experience in handling health related conditions and perceived needs for accessing health services	**(+)** Awareness of the need for healthcare services[38 54 56]; **(-)** home remedies[71] or management of childhood illness[53 85]	**(+) Perceived needs for healthcare services[21 68 75 76 91 94 113 129]; (-) home delivery[82]**
Perception/knowledge/experience/preference of health services	**(-)** Fear of mistreatment[72 101] and **(-)** doubts about medical care[38 79 100]; gender-induced affordability[102]; **(-)** provider shopping[71]	**(positive)** Perception of healthcare services[21 42 64 76 84 86-90] and providers[21 85 94 112 125]; **(+)** knowledge of health services[21 42 49 61 68 75 82 86 91-94] or facilities[21 95 96]; **(+) previous use of related healthcare services[39 42 50 60 62 67 80 97 98]; (-)** bad experiences of friends and relatives at healthcare facilities[95]; **(-) misunderstanding or fear[21 68 75 76 89 99 129]**; gender healthcare worker preference[88]; **(-) side effect[82]; lack of trust[46]**
Socioeconomic factors		
Socioeconomic status	**(-)** Social class[104]; **social group (caste) of caregiver[54]**	**(+)** Socioeconomic status[21 39 40 47 57 62 82 103]; Caste[109 115]; (rent-> negative) residential background[21 47 69 82]; **(+)** possession of ration card[69]
Marital status	**(married)** Marital status[70]	**(married) Marital status[18 41]; duration of marriage[42]**
Family composition & Living arrangement	**(-) Family size[54]**	**(±)** Family type[39 51 116 127]; **(±)** family size[67 69]; **(-) number of children in household[21 44 49]**; **(+)** number of male children[136]; **(+)** housing condition[21]
Education	**(+)** Education[54 56 58 70]	**(+)** Education[39 41 42 45 49 57 59 61 62 66-68 81-83 107-109 115-117 127 135]; **(±) husband education[44 51]**; **(+) mother's education and literacy[21 43 47 57 60]**
Income and wealth	**(+)** Income[56 118]; **(+)** wealth[55 58]; **(-)**	**(+)** Income[41 50 51 68 81 105]; **(+)** wealth[21 49 61]

For peer review only - http://bmjopen.bmj.com/site/about/guidelines.xhtml

		inability to afford care [19 38 77 79 85 90 101 110 111]	[66 106-109]; (-) financial constraint[21 46 74 87 103 112-114]
	Occupation	(+) Occupation[54 58 70]	(+) Employment[21 66 84 119 127]; (±) occupation[49 67 81 83 105 135]; (±) occupation of spouse[51 61] or household[69]
	Social support	(-) Difficulty in reaching services (security risk at night)[100]; (+) accompanying person[77]; **decision making person for seeking health care[54]**	(+) Family support[75 88]; (+) social connectedness[94]; (+) socioeconomic support[122]; **permission for immunisation by decision-maker[129]**
	Competing priorities/lack of time	(-) Competing priorities (ability to work and income)[120]; (-) not want to miss work[71]; (-) lack of time[101 121]	(-) Competing priorities[74 82 94]; (-) risk of lost income[21]; (-) parents being too busy[21]
Physical environment			
	Distance from health facility	Proximity of healthcare facilities[38 55 77 85 123]; geographical distance of formal healthcare[102]	(-) Distance from health facility[21 47 81 86 96 109 124-127]
	Transport	(+) Travel assistance[77]; (-) no transportation[38]	(-) Lack of transportation[83 96 103]; (-) variability in traffic congestion[128]
	Environment of residence area	(-) Difficulty in reaching services (darkness at night)[100]	Residential background[21 69 106]
Cultural and religious factors			
	Religion	Religion[70]	Religion[41 57 60 76 115 129]
	Sociocultural influence	(-) Stigma[79 102 130]; mother tongue[70]; (-) difficulties in disclosing the symptoms, (-) neglecting behaviours, and socio-cultural influences[78]; (+) cultural competency of care[102]; (+) easy communication[102]; living with the burden of cultural expectations[102]; (-) no permission to seek care from family[38]	(-) Exposure to media[80 98]; **stigma[129]; (-)** cultural expectation for women after birth and fear of stigma for pregnancy out of wedlock[94]
	Tradition	(-) Traditional medicine[100]	(-) Traditional remedies[76]; (-) home remedies[87]
Legal, political and policy factors			
	Legal issues	(-) Perceived illegality of abortion[79]	Type of slums and possession of a ration card[80]
Health system factors			
	Accessibility	(+) Ease of access[102]; (-) late facility opening times[85]	(-) Limited access to the services due to location[91 94]; (-) timing of services[21 87]; household visit by health workers[21]
	Quality and safety of services	Quality of treatment and expected outcome of therapies[38 102]; (-) delay in advising related tests[71]; referral[71]; optimal examination[85 110]; (-) provider shopping[71]	Quality of service[67 86 87 92 113 125]; (-) adverse events[76]
	**Charges for health services**	**(+) Insurance coverage of both public and private providers and of extended family members[137]**	**(-) Average out-of-pocket healthcare expenditure[135]; healthcare insurance[18 64 83]**
	Service organisation and delivery arrangement	(-) Medical turnover and overload or healthcare providers[131]; (+) government/NGO facility[118]; (-) private hospital[38]; **early engagement by healthcare workers[55]**	Attitude of healthcare providers[87 96]; mode of delivery[39 57 60 63 82 138]; (-) hospitals refused to accept health insurance cards[95]

For peer review only - http://bmjopen.bmj.com/site/about/guidelines.xhtml

Facility & resources	Availability of medicines and supplies[85 110]; **(-)** lack of healthcare facilities[139]	Type of healthcare facility[40 86 96 117 132 134]; **inadequate resources[91]; (+)** number of available healthcare workers[47]; **(-)** unavailability of female birth attendants[113]
Waiting time	**(-)** Waiting time[73 85 110]	**(-)** Waiting time[86 87 112 133]

(-) negative association; **(±)** inconsistent/conflicting evidence or context-dependent; **(+)** positive association; NGO: non-governmental organisation

For peer review only

For peer review only - http://bmjopen.bmj.com/site/about/guidelines.xhtml

Supply side: Provision of healthcare services

Ten articles described factors associated with provision of healthcare services in slums from the service providers' perspective. None of the studies reported personal and biological factors. Factors related to other categories are summarised below.

Cognitive and experiential factors: Odhiambo et al. reported slum residents' fear of side effects, size of tablet and misconceptions regarding treatment as the factors hindering drug administration activities by healthcare workers for a deworming programme in Kenya.[140] On the other hand, this study also reported a high demand for drugs from slum residents in the final year of this program because people realised that free treatment was to be ended.[140]

Socioeconomic factors: effective community mobilisation was a facilitator[140] whereas poor community support[141] and insufficient time allocated for providers to implement healthcare programmes[140] were barriers for provision of healthcare services in slums. In the deworming programme mentioned above, community health workers reported that direct observation of slum residents taking deworming drugs after meals was sometimes not feasible because slum residents skipped or age late at night due to food shortage.[140] Some slum residents demanded money to take the deworming drugs, either to facilitate purchase of food or to have their own share of the money that they perceived the community health workers would be paid by the programme if they complied with taking the drugs.[140]

Physical environment: poor sanitation,[140 142] presence of rodents and no pavement,[142] bushy and unprotected environment[140] were reported as factors making the provision of healthcare services difficult in slums.

Cultural and religious factors: religious beliefs and mistrust of interventions,[140] lack of a shared understanding of the needs, purposes and consequences of family planning and pregnancy related services among slum residents and healthcare providers[74] were the barriers

For peer review only - http://bmjopen.bmj.com/site/about/guidelines.xhtml

for healthcare services provision. In the previous deworming programme, portrayal of unrelated death being linked to the programme and related negative publicity affected participants' compliance.[140]

Legal, political and policy factors: devolution of service delivery through downward transfer of funds and responsibilities from central/national government level to elected local bodies; management by professional managerial and technical cadres; tight organisation of public health services; and professional support from the state directorate of public health were found to strengthen public health service provision in Chennai slums compared with Delhi.[143] One study reported that policies affected healthcare provision negatively because of staff shortage arising from change and suspension of the appointment of health promotors, which led to overwork and lack of time to provide required care by healthcare staff. [133] In Brazil, home visits for the provision of healthcare services was hampered because slum residents could not present documents required to register for healthcare.[142] On the other hand, giving priority to socially less developed areas for strengthening the Family Health System in Brazil might have been associated with better service coverage for slum residents with tuberculosis compared with their urban non-slum counterparts.[144]

Health system: pay scale of frontline healthcare workers,[141] knowledge of intervention area by community health workers,[140] issues related to rigid task assignment by service managers,[142] requirement to follow standardised protocol,[142] demands from the management,[142] work burden[133] [142] and no incentive,[141] insufficient time,[140] attitude[74] and support of healthcare providers,[141] **ill-defined geographic boundary of service with unserved areas and left-out urban slum pockets[145]** were associated with healthcare service provision in slums. Lack of community-based care (such as school-based education for reproductive health and community support networks for women),[133] **unreliable immunisation and household data[129];**

For peer review only - http://bmjopen.bmj.com/site/about/guidelines.xhtml

inefficient utilisation of funds,[129] affordability (price) and availability of medicine,[146] limited medical supplies[74 141] and infrastructural facilities,[141] inadequate space and equipment,[142 145] **suboptimal training of staff,**[145] **insufficient availability of logistics, and health manpower**[145] also affected service provision. (Table 3)

For peer review only

For peer review only - http://bmjopen.bmj.com/site/about/guidelines.xhtml

Table 3. Factors associated with provision of healthcare services in slums **from service provider's (supply side) perspective.**

Cognitive and experiential factors	
Perception/knowledge/experience/preference of health services	Fear of side effects, size of tablet and misconceptions regarding treatment, high demand for drugs in the final year of treatment[140]
Socioeconomic factors	
Income and wealth	Difficulty in directly observing deworming treatment at meal time due to food shortage[140]
Social support	Effective community mobilisation[140]; poor community support[141]; **non-involvement of community members and Urban Local Bodies[145];** absence of community members during the drug administration exercise[140]; demand for incentives by community members to take deworming drugs[140]
Physical environment	
Environment of residence area	Environment (sanitation, territory)[142]; unsanitary environmental conditions[140]; inaccessibility (filthy and bush environment)[140]
Cultural and religious factors	
Religion	Religious beliefs and mistrust of interventions[140]
Sociocultural influence	Lack of shared understanding of the problems in community[74]; unrelated death and the associated negative publicity (of a deworming programme) by the media[140]
Legal, political and policy factors	
Policy issues	Devolution of service delivery transferring funds and responsibilities to elected local bodies [143]; management by professional managerial and technical cadres[143]; tight organisation of public health services[143]; professional support from the state directorate of public health[143]; healthcare policies[133]; policy prioritizing low social development areas [144]
Legal issues	Fear of requirement for formal registration[142]
Health system factors	
Cost	Pay scale of frontline healthcare workers[141]; medicine price[146]
Quality and safety of services	Knowledge of intervention area by community health workers[140]
Service organisation and delivery arrangement	Issues related to assignment of tasks[142]; requirement to follow standardised protocol [142]; demands from the management[142]; work overload[133 142]; **underperformance of staff[129];** documentation work/work burden/no incentive for work[141]; insufficient time[140]; attitude of healthcare providers[74]; lack of supportive staff[141]; community health worker familiarity with households led to warm reception[140]; opportunity to integrate mass drug administration with other health interventions[140]; presence of community health workers and their supervisory structure, and points of referral for serious side effects[140]; **restriction of range of services[145]; unserved areas and left-out urban slum pockets[145]; poor monitoring and supervision[145]; unreliable immunisation and household data[129]**
Facility & resources	Community-based care[133]**; inefficient utilisation of funds[129];** affordability and availability of medicine[146]; limited medical supplies[74 141]; infrastructural facilities[141]; inadequate space and equipment[142]; **suboptimal training of staff[145]; insufficient availability of space, logistics, and health manpower[145]**

For peer review only - http://bmjopen.bmj.com/site/about/guidelines.xhtml

Comparison between slums and other settings

Seven studies which met our inclusion criteria also included data from non-slum urban and/or rural areas and potentially allowed exploration of factors associated with healthcare access across different settings. Key findings from these studies are summarised in Table 4.

These recent studies showed a mixed and dynamic picture of healthcare access across slum and other settings and reported various factors associated with this. For example, the proportion of young children fully immunised was found to be lower in slums compared with non-slum urban setting but was higher than rural settings in Nigeria. Nevertheless the coverage improved over time across all settings.[60] While many common factors associated with full immunisation of young children were identified, giving birth in health facilities (as opposed to home) had a larger positive effect on subsequent immunisation coverage in slums compared with non-slum urban and rural settings.[60] A narrowing of gaps in delivery by skilled birth attendants between slum and non-slum urban settings over time and a reverse of the trend from having lower usage to higher usage of modern contraceptive methods by married women in slums versus urban non-slums were reported in Bangladesh.[47] Slum residents reported financial issues being the main reason for not taking prescribed drugs whereas getting better was the cited main reason for urban non-slum residents in Iran.[114] Better coverage of services and higher rates of treatment completion were reported for patients with tuberculosis in slums compared with non-slum urban setting in two studies in Brazil,[52 144] where a higher priority given to enhancing the Family Health system in socially less developed areas in recent years was suggested to be a likely factor associated with better service provision in slums.[144] (Table 4)

For peer review only - http://bmjopen.bmj.com/site/about/guidelines.xhtml

Table 4. Studies that examined factors associated with health care seeking and utilisation in both urban slum and non-slum urban and rural settings

Study & location	Differences in healthcare access	Associated factors
**Kanyango (2021)[137]**	***Preferences and willingness to pay for health insurance*** Households in non-slum communities had a high preference for health insurance plans covering chronic illnesses and major surgeries to other plans.	Coverage of extended family (vs restricted enrollment of children); coverage of both private and public providers (vs private only)
Obanewa (2020)[60]	***Fully-immunised child coverage (FIC)*** Proportion in slum lower than urban non-slum but higher than rural; proportions increased between 2003 and 2013 across all three settings	From multivariable regression*: year, birth order, antenatal attendance, maternal education level, religion, maternal age at child's birth, media exposure, region of the country, interaction between place of residence and place of delivery
Angeles (2019)[47]	***Use of modern contraceptive methods*** Proportion changed from being lower in slums in 2006 to being higher in slums in 2013 compared with urban non-slums	From multivariable regression*: parity, mother's age, mother's education attainment, socioeconomic status, interaction (slum × time period)
	Delivery by skilled birth attendant Proportion substantially lower in slums compared with urban non-slums but the gaps narrowed over time)	From multivariable regression*: Residing in slums, parity, mother's age, mother's education attainment, length of stay in current city of residence, socioeconomic status, number of available community health worker, distance from health facility, interaction (slum x time period)
Islam (2018)[107]	***Antenatal care visits*** "there was a large inequality" between slum and urban non-slum (detail not reported)	Level of educational attainment, wealth index of the household
	Using contraceptive methods "Prevalence rate higher among slum women" than urban non-slum women	Not reported

For peer review only - http://bmjopen.bmj.com/site/about/guidelines.xhtml

Tabrizi (2018)[114]	***Utilisation of health services in the past 30 days***<br>Similar utilisation overall, but with lower proportion received needed health services and used private clinics, higher use of vaccination and maternal health services, and lower use of services for heart failure and hypertension for slum residents compared with urban non-slum	High cost of services
	Home care services<br>Very little use both in slum and urban non-slum areas	High cost of services
	Prescribed drug during last visit to health facilities<br>Lower proportion for slum vs urban non-slum	Not reported
	Not taking drugs prescribed<br>Higher proportion for slum vs urban non-slum	Main reason: financial problems for slum vs getting better/feeling well for non-slum urban
Snyder (2016)[52]	***Directly observed treatment coverage for tuberculosis (TB)***<br>Higher for slum vs urban non-slum TB patients	Not examined
	Abandonment of TB treatment<br>Lower for slum vs urban non-slum TB patients	From multivariable regression*: residency in a slum, sex, age, extrapulmonary clinical disease, HIV/AIDS, interaction (directly observed treatment x residency in a slum)
Prado Junior (2016)[144]	***Coverage under Family Health system for TB patients***<br>Higher for slum vs urban non-slum	Giving the Family Health strategy priority to coverage of areas with lower social development

*From the model with most comprehensive adjustment including residency in slum as one of the variables; only factors that were statistically significant (at 5% level) are shown. AIDS: Acquired Immune Deficiency Syndrome; HIV: human immunodeficiency virus; TB: tuberculosis.

For peer review only

For peer review only - http://bmjopen.bmj.com/site/about/guidelines.xhtml

DISCUSSION

Statement of principle findings

This scoping review of recent literature examined demand side factors associated with slum residents' healthcare **accessing and** utilisation, as well as supply side factors associated with provision of health services in slums. **We found over 104 studies related to the former, but only 10 studies related to the latter.** We identified different factors associated with **accessing, utilisation** and provision of health services in slums, and mapped them to a conceptual framework developed and refined for this review into seven broad categories (Figure 2).

Findings in the context of existing literature

Even though previous reviews have investigated factors associated with healthcare access in various settings,[147 148] to our best knowledge this scoping review is the first that has examined **wide-ranging** factors across different service areas of health care in slums. Our findings are consistent with previous studies which highlighted common factors associated with healthcare seeking and utilisation such as age, income and education.[147 149] We identified several factors that are particularly pertinent in slum settings, such as costs of healthcare,[19 21 74 77 79 85 90 101 103 110-112] lack of time due to slum residents' competing priorities[21 101 121] and issues arising from adverse physical environment,[83 103 140 142] security,[100 142] fear of formal registration due to distrust of the authorities[142] and proximity of healthcare facilities.[21 77 81 85 86 109 123-126] In addition, included studies showed that the effects of a given factor may differ between slum, urban non-slum and rural settings.[60]

Healthcare cost is a major barrier between the intention to seek care and actual utilisation of services. [110 139] **Health insurance could be one of the potential** measures to overcome this barrier.[150 151] **Although possession of/coverage by health insurance was associated with**

For peer review only - http://bmjopen.bmj.com/site/about/guidelines.xhtml

higher levels of utilisation of health services among slum residents,[18 83] studies showed that uptake of **government-run** public insurance among slum residents was low.[69 84] **This may be attributed to lack of awareness, difficulties in navigating through the health system and in obtaining official proof of identity required for enrolment,**[69] **and poor quality of care and range of services offered.** [69 84] **Even among slum residents covered by health insurance, access to care was often refused and additional charges were frequently requested.**[95] **Policies that aim to improve access to healthcare services among slum residents through public health insurance will need to address these challenges.**

Several studies reported lack of time and competing priorities as a factor affecting healthcare-seeking behaviour[101 120 121] and health services utilisation.[21 74 94] This suggests a delicate balance between factors that individual slum residents have to strike when making decisions on healthcare seeking and utilisation. Var der Heijden et al. showed that health was considered as an asset for working ability in slums,[120] but paradoxically the ability to work often seems to impede healthcare seeking for health issues. This highlights the importance of considering slum residents' interest and priorities when providing healthcare services and promoting healthcare utilisation in slums.

Strengths and weaknesses of the review

This scoping review has several strengths. We conducted a comprehensive literature search using generic terms related to slums with few other restrictions. The search was therefore likely to be sensitive for identifying relevant literature. Contemporary methodological guidelines for undertaking scoping reviews were followed,[22] and a conceptual framework which was adapted based on emerging findings was used to facilitate the organisation of evidence.

The review has enabled theory building and refinement of a conceptual framework. Our

For peer review only - http://bmjopen.bmj.com/site/about/guidelines.xhtml

preliminary framework included six categories (Figure 1). During data coding and extraction, it emerged that many studies reported perception, knowledge, and experience of slum residents being associated with their healthcare-seeking and utilisation. We subsequently classified these factors as cognitive and experiential factors, which primarily consists of three subcategories: knowledge/experience of illness, perceived needs for accessing healthcare services, and perception/experience of healthcare services. These factors were influenced by other factors included in our original conceptual framework, but highlighted the crucial links between those factors and the ultimate actions by individual slum residents to access health services. Future interventions to promote health service utilisation for slum residents[152] could make use of our framework to develop programme theories and map out causal pathways.

This review also has some limitations. Given time constraint, we were only able to examine the most recent literature published in English **in academic journals**,, and have not examined the methodological quality of individual studies (which we noted to be quite varied) in detail. We attempted some preliminary synthesis to configure the identified evidence but have not explored the complex relationship between the factors identified **and their interplay with the context of individual slums** in depth. Nevertheless, findings from this scoping review will provide a good foundation for further syntheses.

For peer review only

Methodological considerations

A number of challenges in the process of classifying and coding data are worth mentioning. **Firstly, access to healthcare has been conceptualised and defined in various ways in previous studies. The World Health Organization suggested six building blocks of a health system including service delivery, health workforce, health information systems, access to essential medicines, financing, leadership/governance to strengthen health**

For peer review only - http://bmjopen.bmj.com/site/about/guidelines.xhtml

systems,[153] and in the report, defined access to healthcare as public responsibility for ensuring all citizens' entitlements to the protection of their health beyond simply proportion of a target population that benefits from an intervention or universal coverage.[153] They also pointed out system constraints such as financial access difficulty, physical access difficulty, low knowledge and skills, poorly motivated staff, weak leadership and management, ineffective intersectoral action and partnership as barriers to access.[153] The WHO's definition and conceptual framework focus on health system level factors and would be particularly useful when examining supply side factors, which seem to be under-studied based on our findings. As described in the Methods section, we primarily adopted the conceptual model of healthcare access developed by Levesque and colleagues given our shared focus on service users. However, in our conceptual model we separated the dynamic stages of 'accessing' healthcare from the actual 'accessed' healthcare utilisation to highlight the crucial match required between the demand side and supply side factors to facilitate access to healthcare.

Several factors associated with healthcare accessing and utilisation can be viewed from different perspectives and therefore potentially be coded under different categories. For example, barriers for healthcare seeking and utilisation related to costs can be considered as socioeconomic issues from the slum dwellers' perspective but can also be viewed as health system issues for not offering the services in an affordable way. Indeed, previous access frameworks suggested that access is created and negotiated in a dynamic interchange between households/communities and healthcare workers/systems (i.e. demand and supply) on each access dimension.[34 154] In such situations, we tried to code a factor under the category that most directly reflects the original data through discussions within the review team (in the example of healthcare cost, the factor was coded primarily under socioeconomic factors rather than

For peer review only - http://bmjopen.bmj.com/site/about/guidelines.xhtml

health system factors when the factor was reported by slum residents as a barrier); otherwise more than one category was coded (for example, bad experience from previous utilisation of health services was coded both as a cognitive and experiential factor and a health system factor).

Implication for research and practice

The multitude of factors identified in this review are often inter-related and inter-acting, and span across personal, family, community and society levels. For example, the association between occupation and healthcare utilisation were reported in several studies.[49 61 67 81 83 105] The effect of predominantly casual work undertaken by slum residents on their healthcare access could be mediated through working hours, income level, knowledge of health and available services, etc. There is also possibility that occupation was associated with health status and hence needs for healthcare services, instead of/in addition to behaviour of using healthcare services. Teasing out the complicated relationships between various determinants **and their interaction with the diverse contexts of slums** will require in-depth analysis and a more holistic approach to synthesising the evidence. Given the unique features of individual slums, service planners and policy makers will need to examine these relationships with due consideration to the context specific to each locality and geospatial features and neighbourhood effects that characterise slum settings.[4]

We found far fewer studies that have examined health service providers' perspective than studies that have investigated factors associated with accessing healthcare from slum residents' perspective. There may be scope for greater research and policy attention to supply-side factors, including experiences and practices of local frontline healthcare providers, availability of healthcare facilities and infrastructure and policy to support them in order to overcome the many barriers highlighted from both supply and demand sides.

For peer review only - http://bmjopen.bmj.com/site/about/guidelines.xhtml

Although only six of the included studies explored factors associated with healthcare access or health service provision across slum and non-slum settings, they showed a generally encouraging picture that access to and provision of healthcare are continuously evolving (and often improving) in slums and other settings, and equality between different settings is not beyond reach.

CONCLUSION

This scoping review summarises a large body of recent literature evaluating factors associated with seeking and utilisation of healthcare by slum residents, but found substantially fewer studies examining factors associated with provision of health services from providers' perspective. Recent migration into slums; knowledge, perception (including misconception and distrust) and past experience of illness, healthcare needs and health services; financial constraint, competing priorities and inadequacy of social support; adverse physical environment and unfavourable locality; sociocultural expectations and stigma; lack of official recognition; and various problems in existing health system all contribute towards the challenges faced by slum residents. Future research and policy aiming at improving healthcare services in slums should pay more attention to supply side issues ranging from individual healthcare providers and practices to structural and policy level factors to tackle different barriers faced by slum residents, which in turn need to be evaluated holistically and take into account local context and geospatial features of slums.

For peer review only - http://bmjopen.bmj.com/site/about/guidelines.xhtml

For peer review only

List of abbreviations

GRADE: Grading of Recommendations Assessment, Development and Evaluation

LMICs: Low and Middle income Countries

MMAT: Mixed Methods Appraisal Tool

WHO: World Health Organization

Ethics approval

Not applicable. This realist synthesis included literature that is available in the public domain and did not involve the collection of personal data.

Consent for publication

The authors were required to notify the funder of the research, the UK National Institute for Health Research (NIHR) prior to the publication of this manuscript. The funder did not otherwise play any roles in the preparation of the manuscript and decision to submit it.

Availability of data and materials

All data relevant to the study were included in the article or uploaded as supplementary information. No additional data were available.

Competing interests

The authors declare that they have no competing interest.

Acknowledgement and Funding

This research is funded by the NIHR Global Health Research Unit on Improving Health in Slums using UK aid from the UK Government to support global health research (Award/Grant number is not applicable). The views expressed in this publication are those of the author(s) and not necessarily those of the NIHR or the UK Department of Health and Social Care. MA gratefully acknowledges support provided by the Warwick Institute of Advanced Study Global Challenges Research Fund Fellowship No. IAS/32013/1914. FG receives funding as South

For peer review only

Africa Research Chair in Health Policy and Systems from the National Research Foundation, South Africa (Award/Grant number is not applicable). RL is supported by the NIHR Applied Research Collaboration (ARC) West Midlands, UK (Award/Grant number is not applicable). YFC is supported by Warwick Evidence, which is a Technology Assessment Review team funded by the NIHR Evidence Synthesis Programme (Award/Grant number is not applicable). Upon submission, NA had joined the Population Health Sciences Institute, Newcastle University (UK).

Contributor

JEP, BH, MA, FG and YFC conceptualised the scoping review; JEP carried out literature searches; JEP, PK, GY, OO, and YFC participated in study screening and coding; JEP and YFC performed data charting and drafted in initial manuscript. NA, PG and RL provided critical input during the drafting of the manuscript. All authors commented on and contributed to the revision of subsequent versions and approved the final version for submission.

Collaborators

The Improving Health in Slums Collaborative:

African Population and Health Research Centre (APHRC), Nairobi, Kenya:

Pauline Bakibinga, Caroline Kabaria, Ziraba Kasiira, Peter Kibe, Lyagamula Kisia, Catherine Kyobutungi, Nelson Mbaya, Blessing Mberu, Shukri Mohammed, Anne Njeri.

Aga Khan University, Karachi, Pakistan:

Iqbal Azam, Romaina Iqbal, Ahsana Nazish, Narjis Rizvi.

Independent University, Bangladesh, Dhaka, Bangladesh:

Syed Shifat Ahmed, Nazratun Choudhury, Omar Rahman, Rita Yusuf.

Nigerian Academy of Sciences, Lagos, Nigeria:

Doyin Odubanjo.

For peer review only

For peer review only - http://bmjopen.bmj.com/site/about/guidelines.xhtml

University of Ibadan, Ibadan, Nigeria:

Motunrayo Ayobola, Olufunke Fayehun, Akinyinka Omigbodun, Mary Osuh, Eme Owoaje, Olalekan Taiwo.

University of Birmingham, Birmingham, UK:

Richard Lilford, Jo Sartori, Samuel Watson.

University of Lancaster, Lancaster, UK:

Peter Diggle.

University of Warwick, Coventry, UK:

Navneet Aujla, João Porto de Albuquerque, Yen-Fu Chen, Paramjit Gill, Frances Griffiths, Bronwyn Harris, Jason Madan, Oyinlola Oyebode, Ji-Eun Park, Simon Smith, Grant Tregonning, Olalekan Uthman, Ria Wilson, Godwin Yeboah.

REFERENCES

1. United Nations. The Sustainable Development Goals Report, 2019.

2. Kosamkar A. Problems in urban society. *EPRA Int J Multidiscip Res* 2020;6.

3. Ezeh A, Oyebode O, Satterthwaite D, et al. The history, geography, and sociology of slums and the health problems of people who live in slums. *Lancet* 2017;389:547-58.

4. Lilford R, Kyobutungi C, Ndugwa R, et al. Because space matters: conceptual framework to help distinguish slum from non-slum urban areas. *BMJ Glob Health* 2019;4:e001267.

5. Zulu EM, Beguy D, Ezeh AC, et al. Overview of migration, poverty and health dynamics in Nairobi City's slum settlements. *J Urban Health* 2011;88 Suppl 2:S185-99.

6. Michiani MV, Asano J. Physical upgrading plan for slum riverside settlement in traditional area: A case study in Kuin Utara, Banjarmasin, Indonesia. *Front Archit Res* 2019;8:378-95.

For peer review only - http://bmjopen.bmj.com/site/about/guidelines.xhtml

7. Winter SC, Dreibelbis R, Dzombo MN, et al. A mixed-methods study of women's sanitation utilization in informal settlements in Kenya. *PLoS One* 2019;14:e0214114.

8. Salinas DA, Fouts HN, Neitzel CL, et al. Young Children's Social Networks in an Informal Urban Settlement in Kenya: Examining Network Characteristics Among Kamba, Kikuyu, Luo, and Maasai Children. *J Cross-Cult Psychol* 2019;50:639-58.

9. Prayitno G, Sari N, Putri IK. Social capitl in poversity alleviation through pro-poor tourism concept in slum area (case study: Kelurahan jodipan, Malang city). *Int J GEOMATE* 2019;16:131-37.

10. Khalil D. The Flexible Governance of Water in Cairo's Informal Areas. *Water* 2019;11:1644.

11. Portner CC, Su YH. Differences in Child Health Across Rural, Urban, and Slum Areas: Evidence From India. *Demography* 2018;55:223-47.

12. Mberu BU, Haregu TN, Kyobutungi C, et al. Health and health-related indicators in slum, rural, and urban communities: a comparative analysis. *Glob Health Action* 2016;9:33163.

13. Mlangeni L, Makola L, Naidoo I, et al. Factors associated with physical activity in south africa: Evidence from a national population based survey. *Open Public Health Journal* 2018;11:516-25.

14. Rebecca L. Nunn, Sarah H. Kehoe, Harsha Chopra, et al. Dietary micronutrient intakes among women of reproductive age in Mumbai slums. *Euro J of Clinical Nutrition* 2019;73:1536–45.

15. Samal J. Perception and knowledge of tuberculosis and its services among slum dwellers in Chhattisgarh. *Indian J Respir Care* 2017;6:828-31.

16. Khan MZ, Shujaa MD, Iftikhar H. Utilization of ante-natal services among reproductive age women of Bahawalpur. *Indo Am J Pharm Sci* 2018;5:11355-65.

For peer review only - http://bmjopen.bmj.com/site/about/guidelines.xhtml

17. Duy Kien V, Van Minh H, Bao Giang K, et al. Horizontal inequity in public health care service utilization for non-communicable diseases in urban Vietnam. *Glob Health Action* 2014;7:24919.

18. Amiresmaili M, Yazdi-Feyzabadi V, Heidarijamebozorgi M. Health services utilization among slum dwellers: An experience from Iran. *J Educ Health Promot* 2019;8:210.

19. McNairy ML, Tymejczyk O, Rivera V, et al. High Burden of Non-communicable Diseases among a Young Slum Population in Haiti. *J Urban Health* 2019;96:797-812.

20. Sverdlik A. Ill-health and poverty: a literature review on health in informal settlements. *Environ Urban* 2011;23:123-55.

21. Crocker-Buque T, Mindra G, Duncan R, et al. Immunization, urbanization and slums - a systematic review of factors and interventions. *BMC Public Health* 2017;17:556.

22. Peters MDJ, Godfrey C, McInerney P, et al. Chapter 11: Scoping Reviews. In: Aromataris E, Munn Z (Editors). Joanna Briggs Institute Reviewer's Manual 2017

23. Center for Open Science 2020. Slum health healthcare access and provision of services scoping review protocol 05082020.pdf (Version: 1). Available from: https://osf.io/mj6kp/ [Accessed 9 Jun 2021].

24. United Nations. United Nations 2021. Available from: https://www.un.org/en [Accessed 25 Dec 2021].

25. World Health Organization. World Health Organization 2021. Available from: https://www.who.int [Accessed 25 Dec 2021].

26. SDI. SDI 2021 Available from: https://sdinet.org [Accessed 25 Dec 2021].

27. UN HABITAT. UN-Habitat for a better urban future 2021. Available from: https://unhabitat.org/ [Accessed 25 Dec 2021].

28. Ahmed S, Ajisola M, Azeem K, et al. Impact of the societal response to COVID-19 on

For peer review only - http://bmjopen.bmj.com/site/about/guidelines.xhtml

access to healthcare for non-COVID-19 health issues in slum communities of Bangladesh, Kenya, Nigeria and Pakistan: results of pre-COVID and COVID-19 lockdown stakeholder engagements. *BMJ Glob Health* 2020;5:e003042.

29. Improving Health in Slums C. A protocol for a multi-site, spatially-referenced household survey in slum settings: methods for access, sampling frame construction, sampling, and field data collection. *BMC Med Res Methodol* 2019;19:109.

30. **George CE, Inbaraj LR, Rajukutty S, et al. Challenges, experience and coping of health professionals in delivering healthcare in an urban slum in India during the first 40 days of COVID-19 crisis: a mixed method study. *BMJ Open* 2020;10:e042171.**

31. Peduzzi P, Chatenoux B, Dao H, et al. Global trends in tropical cyclone risk. *Nature climate change* 2012;2:289-94.

32. Effective Practice and Organisation of Care (EPOC). EPOC Taxonomy 2015. Available from: https://epoc.cochrane.org/epoc-taxonomy [Accessed 27 Jul 2020].

33. Lavis JN, Wilson MG, Moat KA, et al. Developing and refining the methods for a 'one-stop shop' for research evidence about health systems. *Health Res Policy Syst* 2015;13:10.

34. Levesque JF, Harris MF, Russell G. Patient-centred access to health care: conceptualising access at the interface of health systems and populations. *Int J Equity Health* 2013;12:18.

35. National Cancer Institute. Theory at a Glance: A Guide for Health Promotion Practice, 2005.

36. Mpanje D, Gibbons P, McDermott R. Social capital in vulnerable urban settings: an analytical framework. *J Int Humanit Action* 2018;3:4.

37. Tricco AC, Lillie E, Zarin W, et al. PRISMA Extension for Scoping Reviews (PRISMA-ScR): Checklist and Explanation. *Ann Intern Med* 2018;169:467-73.

38. Pune Municipal Corporation. City Health Plan 2016 - 2020, 2016. Available from:

For peer review only

For peer review only - http://bmjopen.bmj.com/site/about/guidelines.xhtml

https://www.healthynewbornnetwork.org/hnn-content/uploads/Pune-City-Health-Plan.pdf [Accessed 10 Jun 2021]

39. Upadhyai N, Gupta SK. Utilization of Postnatal Care Services and Factors Affecting It among Women of Urban Slums in Dehradun, Uttarakhand. *Indian J Community Health* 2019;31:470-76.

40. Mendhe HG, David R, Singh D, et al. Universal Health Insurance coverage and utilization among women in urban slum of Rajnandgaon, Chhattisgarh. *J Family Med Prim Care* 2021;10:1313-19.

41. Iyalomhe FO, Adekola PO, Cirella GT. Community-based health financing: empirical evaluation of the socio-demographic factors determining its uptake in Awka, Anambra state, Nigeria. *Int J Equity Health* 2021;20:235.

42. Yadav K, Shukla M, Agarwal M, et al. Unmet need for family planning services among young married women (15-24 years) living in urban slums of India. *BMC Womens Health* 2020;20:187.

43. Veras A, Lima EJD, Caminha MDC, et al. Vaccine uptake and associated factors in an irregular urban settlement in northeastern Brazil: a cross-sectional study. *BMC Public Health* 2020;20:1152

44. Roja VR, Narayanan P, Sekaran VC, et al. Living environment and health of under-five children in urban slums of a coastal region in South India. *Ghana Medical Journal* 2020;54:238-44.

45. de Araujo Veras AAC, da Fonseca Lima EJ, Caminha MFC, et al. Vaccine uptake and associated factors in an irregular urban settlement in northeastern Brazil: a cross-sectional study. *BMC Public Health* 2020;20:1152.

46. Vora K, Saiyed S, Shah AR, et al. Surgical Unmet Need in a Low-Income Area of a

For peer review only - http://bmjopen.bmj.com/site/about/guidelines.xhtml

Metropolitan City in India: A Cross-Sectional Study. *World J Surg* 2020;44:2511-7.

47. Angeles G, Ahsan KZ, Streatfield PK, et al. Reducing Inequity in Urban Health: Have the Intra-urban Differentials in Reproductive Health Service Utilization and Child Nutritional Outcome Narrowed in Bangladesh? *J Urban Health* 2019;96:193-207.

48. Renzaho AM, Kamara JK, Georgeou N, et al. Sexual, Reproductive Health Needs, and Rights of Young People in Slum Areas of Kampala, Uganda: A Cross Sectional Study. *PLoS One* 2017;12:e0169721.

49. Horng L, Kakoly NS, Abedin J, et al. Effect of household relocation on child vaccination and health service utilisation in Dhaka, Bangladesh: a cross-sectional community survey. *BMJ Open* 2019;9: e026176.

50. Lae WL, Jayasvasti I, Mongkolchati A, et al. Utilization of immunization service and predictors among under 3-year-old children in urban slums of Chanmyathazi Township, Mandalay, Myanmar. *J Med Assoc Thai* 2018;101:1085-92.

51. Sharma D, Pokharel HP, Budhathoki SS, et al. Antenatal Health Care Service Utilization in Slum Areas of Pokhara Sub-Metropolitan City, Nepal. *J Nepal Health Res Counc* 2016;14:39-46.

52. Snyder RE, Marlow MA, Phuphanich ME, et al. Risk factors for differential outcome following directly observed treatment (DOT) of slum and non-slum tuberculosis patients: a retrospective cohort study. *BMC Infectious Diseases* 2016;16:494.

53. Lungu EA, Darker C, Biesma R. Determinants of healthcare seeking for childhood illnesses among caregivers of under-five children in urban slums in Malawi: a population-based cross-sectional study. *BMC Pediatr* 2020;20:20.

54. Mohanty P, Patnaik L, Satpathy SK, et al. Do the caregivers of under-fives have proper health care seeking behaviour for their children? A study from urban slums

For peer review only - http://bmjopen.bmj.com/site/about/guidelines.xhtml

of a city in eastern India. *J Nepal Paediatr Soc* 2021;41:169-76.

55. Pakhare A, Joshi A, Kumar S, et al. Linkage to primary-care public health facilities for cardiovascular disease prevention: A community-based cohort study from urban slums in India. *BMJ Open* 2021;11:e045997.

56. Kerai S, Nisar I, Muhammad I, et al. A Community-Based Survey on Health-Care Utilization for Pneumonia in Children in Peri-Urban Slums of Karachi, Pakistan. *Am J Trop Med Hyg* 2019;101:1034-41.

57. Devasenapathy N, Jerath SG, Sharma S, et al. Determinants of childhood immunisation coverage in urban poor settlements of Delhi, India: a cross-sectional study. *BMJ Open* 2016;6:e013015.

58. Kar SS, Kalaiselvi S, Archana R, et al. Is rule of halves still an occurrence in South India: Findings from community-based survey in a selected urban area of Puducherry. *J Postgrad Med* 2017;63:232-36.

59. Mishra S, Kusuma YS, Babu BV. Treatment-seeking and out-of-pocket expenditure on childhood illness in a migrant tribal community in Bhubaneswar, Odisha State, India. *Paediatr Int Child Health* 2017;37:181-87.

60. Obanewa OA, Newell ML. The role of place of residency in childhood immunisation coverage in Nigeria: analysis of data from three DHS rounds 2003-2013. *BMC public health* 2020;20:123.

61. Shrestha S, Shrestha DK. Utilization of Maternal Health Care Services among Mothers Residing at Slum Area. *J Nepal Health Res Counc* 2019;17:193-99.

62. Neyaz A, Ahmed MS, Sahu PC. Preference and practices regarding place of childbirth in the slums of a city in Northern India. *J Pioneer Med Sci* 2016;6:33-36.

63. Mishra S, Kusuma YS, Babu BV. Mother's Recognition of and Treatment Triggers for

For peer review only - http://bmjopen.bmj.com/site/about/guidelines.xhtml

Common Childhood Illnesses among Migrant Santal Tribe Living in Bhubaneswar, Odisha, India. *J Trop Pediatr* 2017;63:301-06.

64. Sahu KS, Bharati B. Out-of-Pocket health expenditure and sources of financing for delivery, postpartum, and neonatal health in urban slums of Bhubaneswar, Odisha, India. *Indian J Public Health* 2017;61:67-73.

65. Wambiya EOA, Otieno PO, Mutua MK, et al. Patterns and predictors of private and public health care utilization among residents of an informal settlement in Nairobi, Kenya: a cross-sectional study. *BMC Public Health* 2021;21:850.

66. Razzaque A, Clair K, Chin B, et al. Association of Time since Migration from Rural to Urban Slums and Maternal and Child Outcomes: Dhaka (North and South) and Gazipur City Corporations. *J Urban Health* 2020;97:158-70.

67. Geddam JB, Ponna SN, Kommu PR, et al. Utilization of maternal health services by the migrant population living in the non-notified slums of Hyderabad city, India. *Indian J Community Health* 2017;29:29-38.

68. Singh S, Sahu D, Agrawal A, et al. Ensuring childhood vaccination among slums dwellers under the National Immunization Program in India - Challenges and opportunities. *Prev Med* 2018;112:54-60.

69. Kusuma YS, Pal M, Babu BV. Health Insurance: Awareness, Utilization, and its Determinants among the Urban Poor in Delhi, India. *J Epidemiol Glob Health* 2018;8:69-76.

70. Kulkarni S, Mishra G, Dussane R, et al. Determinants of compliance to breast cancer screening and referral among women from urban slums in India. *Eur J Cancer* 2016;54:S51.

71. Mistry N, Rangan S, Dholakia Y, et al. Durations and Delays in Care Seeking, Diagnosis and Treatment Initiation in Uncomplicated Pulmonary Tuberculosis Patients in Mumbai,

For peer review only

India. *PLoS One* 2016;11:e0152287.

72. Thomson KA, Telfer B, Opondo AP, et al. Navigating the risks of prevention of mother to child transmission (PMTCT) of HIV services in Kibera, Kenya: Barriers to engaging and remaining in care. *PLoS One* 2018;13: e0191463.

73. Kamati M, Godman B, Kibuule D. Prevalence of Self-Medication for Acute Respiratory Infections in Young Children in Namibia: Findings and Implications. *J Res Pharm Pract* 2019;8:220-24.

74. Kaba M, Taye G, Getachew S, et al. Perceived barriers to health care for residents in vulnerable urban centers of Ethiopia. *Ethiop J Health Dev* 2020;34:4-11.

75. Verma H, Sagili KD, Zachariah R, et al. Do incentivised community workers in informal settlements influence maternal and infant health in urban India? *Public Health Action* 2017;7:61-66.

76. Pugliese-Garcia M, Heyerdahl LW, Mwamba C, et al. Factors influencing vaccine acceptance and hesitancy in three informal settlements in Lusaka, Zambia. *Vaccine* 2018;36:5617-24.

77. Ramagiri R, Kannuri NK, Lewis MG, et al. Evaluation of whether health education using video technology increases the uptake of screening for diabetic retinopathy among individuals with diabetes in a slum population in Hyderabad. *Indian J ophthalmol* 2020;68:S37-S41.

78. Sumudrika Ilankoon I, Evangeline Goonewardena C, Fernandopulle R, et al. Women's Understanding and Cultural Practices Related to Vaginal Discharge: A Qualitative Study. *Nurs Midwifery Stud* 2018;7:74-80.

79. Jayaweera RT, Ngui FM, Hall KS, et al. Women's experiences with unplanned pregnancy and abortion in Kenya: A qualitative study. *PLoS One* 2018;13:e0191412.

For peer review only

For peer review only - http://bmjopen.bmj.com/site/about/guidelines.xhtml

80. Kusuma YS, Kaushal S, Garg R, et al. Birth preparedness and determinants of birth place among migrants living in slums and slum-like pockets in Delhi, India. *Sex Reprod Healthc* 2018;16:160-66.

81. Adane M, Mengistie B, Mulat W, et al. Utilization of health facilities and predictors of health-seeking behavior for under-five children with acute diarrhea in slums of Addis Ababa, Ethiopia: a community-based cross-sectional study. *J Health Popul Nutr* 2017;36:9.

82. Shrestha S, Shrestha M, Wagle RR, et al. Predictors of incompletion of immunization among children residing in the slums of Kathmandu valley, Nepal: a case-control study. *BMC Public Health* 2016;16:970.

83. Owusu-Ansah FE, Tagbor H, Togbe MA. Access to health in city slum dwellers: The case of Sodom and Gomorrah in Accra, Ghana. *Afr J Prim Health Care Fam Med* 2016;8:e1-7.

84. Otieno PO, Wambiya EOA, Mohamed SF, et al. Prevalence and factors associated with health insurance coverage in resource-poor urban settings in Nairobi, Kenya: a cross-sectional study. *BMJ Open* 2019;9:e031543.

85. Lungu EA, Biesma R, Chirwa M, et al. Healthcare seeking practices and barriers to accessing under-five child health services in urban slums in Malawi: a qualitative study. *BMC Health Serv Res* 2016;16:410.

86. Owiti A, Oyugi J, Essink D. Utilization of Kenya's free maternal health services among women living in Kibera slums: a cross-sectional study. *Pan Afr Med J* 2018;30:86.

87. Madan NV. Ethnographic Perspectives on Slum-dwelling Women's Access to Primary Care: The Case of Pune, India. *Urbanities* 2019;9:114-30.

88. Wairiuko JM, Cheboi SK, Ochieng GO, et al. Access to Healthcare Services in Informal Settlement: Perspective of the Elderly in Kibera Slum Nairobi-Kenya. *Ann Med Health Sci Res* 2017;7:5-9.

For peer review only - http://bmjopen.bmj.com/site/about/guidelines.xhtml

89. Martinez Perez G, Cox V, Ellman T, et al. 'I Know that I Do Have HIV but Nobody Saw Me': Oral HIV Self-Testing in an Informal Settlement in South Africa. *PLoS ONE* 2016;11:e0152653.

90. Sudhinaraset M, Beyeler N, Barge S, et al. Decision-making for delivery location and quality of care among slum-dwellers: a qualitative study in Uttar Pradesh, India. *BMC Pregnancy Childbirth* 2016;16:148.

91. Sendo EG, Chauke ME, Ganga-Limando M. Why some women who attend focused antenatal care fail to deliver in health facilities: a qualitative study of women's perspectives from slums of Addis Ababa, Ethiopia. *BMJ Open* 2020;10:e039189.

92. Sendo EG, Chauke ME, Ganga-Limando M. Women's perspectives on the measures that need to be taken to increase the use of health-care facility delivery service among slums women, Addis Ababa, Ethiopia: a qualitative study. *Reprod Health* 2021;18(1):174.

93. Manandhar K, Bajcharya K, Prajapati R, et al. Prevalence and Predictors of Incomplete Immunization among Children Residing in the Slums of Kathmandu Valley: A Community Based Door-to-Door Survey. *Kathmandu Univ Med J* 2018;16:8-13.

94. Kaba M, Taye G, Gizaw M, et al. Maternal health service utilization in urban slums of selected towns in Ethiopia: Qualitative study. *Ethiop J Health Dev* 2017;31:96-102.

95. Gupta S. Awareness and utilization of Rashtriya Swasthaya Bima Yojana and its implications for access to health care by the poor in slum areas of Delhi. *Health Systems* 2017;6:242-59.

96. Chauhan M, Saxena S. Barriers in utilization of public health services by elderly slum dwellers in Jaipur city. *Indian Journal of Public Health Research and Development* 2020;11:730-36.

97. Oluoch P, Orwa J, Lugalia F, et al. Application of psychosocial models to Home-Based

For peer review only - http://bmjopen.bmj.com/site/about/guidelines.xhtml

Testing and Counseling (HBTC) for increased uptake and household coverage in a large informal urban settlement in Kenya. *Pan Afr Med J* 2017;27:285.

98. Atusiimire LB, Waiswa P, Atuyambe L, et al. Determinants of facility based-deliveries among urban slum dwellers of Kampala, Uganda. *PLoS ONE* 2019;14:e0214995.

99. Iberico MM, Montoya R, Valiente B, et al. Uptake and utilization of tuberculosis preventive therapy in a Peruvian Peri-urban Shantytown. *Ann Glob Health* 2016;82 (3):366.

100. Hutain J, Perry HB, Koffi AK, et al. Engaging communities in collecting and using results from verbal autopsies for child deaths: an example from urban slums in Freetown, Sierra Leone. *J Glob Health* 2019;9:010419.

101. Misra V, Vashist P, Singh SS, et al. Awareness and eye health-seeking practices for cataract among urban slum population of Delhi: The North India eye disease awareness study. *Indian J Ophthalmol* 2017;65:1483-88.

102. Das M, Angeli F, Krumeich A, et al. The gendered experience with respect to health-seeking behaviour in an urban slum of Kolkata, India. *Int J Equity Health* 2018;17:24.

103. Sheehy G, Aung Y, Foster AM. "She Learned it from her Mother and Grandmother": Women's Experiences with Delivery and Post-partum Practices in Peri-urban Yangon, Myanmar. *Matern Child Health J* 2016;20:854-61.

104. Angeli F, Ishwardat ST, Jaiswal AK, et al. Socio-Cultural Sustainability of Private Healthcare Providers in an Indian Slum Setting: A Bottom-of-the-Pyramid Perspective. *Sustainability* 2018;10:4702.

105. Rahman S. A Comparative Study of Intrauterine Contraceptive Device Utilization among Currently Married Women in a Rural Area of Rani Block and Urban Slums of Guwahati City. *Int J Sci Study* 2016;4:55-59.

106. Mutua MK, Mohamed SF, Iddi S, et al. Do inequalities exist in the disadvantaged

For peer review only - http://bmjopen.bmj.com/site/about/guidelines.xhtml

populations? Levels and trends of full and on-time vaccination coverage in two Nairobi urban informal settlements. *Glob Epidemiol* 2020;2:100044.

107. Islam M. Use of reproductive health care services among urban slum women in Bangladesh. *Eur J Public Health* 2018;28:67.

108. Jolly SP, Rahman M, Afsana K, et al. Evaluation of Maternal Health Service Indicators in Urban Slum of Bangladesh. *PLoS ONE* 2016;11:e0162825.

109. Sadhna S, Kajal J, Debabratta R, et al. Utilisation of maternal health services and its predictors in slum population. *Acta Medica International* 2016;3:56-62.

110. Lungu EA, Guda Obse A, Darker C, et al. What influences where they seek care? Caregivers' preferences for under-five child healthcare services in urban slums of Malawi: A discrete choice experiment. *PLoS One* 2018;13:e0189940.

111. Nasrin M, Sarker MNI, Huda N. Determinants of health care seeking behavior of pregnant slums dwellers in Bangladesh. *Medical Science* 2019;23:35-41.

112. Castiglione D, Lovasi GS, Carvalho MS. Perceptions and Uses of Public and Private Health Care in a Brazilian Favela. *Qual Health Res* 2018;28:159-72.

113. Getachew S, Kaba M, Gizaw M, et al. Health service access, utilization and prevailing health problems in the urban vulnerable sections of Ethiopia. *Ethiop J Health Dev* 2020;34:12-23.

114. Tabrizi JS, Farahbakhsh M, Bazargani HS, et al. Health Services Utilization and Responsiveness: A comparison of Slum and Non-slum Regions in Tabriz, Iran. *Medical Science* 2018;22:577-82.

115. Agrawal D, Tyagi N, Dhakar JS, Chaturvedi M. Awareness and utilization of Geriatric Welfare Schemes among urban elderly population of District Gautam Budh Nagar. *Indian J Community Health* 2019;31:315-21.

For peer review only - http://bmjopen.bmj.com/site/about/guidelines.xhtml

116. Dasgupta P, Bhattacherjee S, Mukherjee A, et al. Vaccine hesitancy for childhood vaccinations in slum areas of Siliguri, India. *Indian J Public Health* 2018;62:253-58.

117. Tebekaw Y, Mashalla YJ, Thupayagale-Tshweneagae G. The adequacy of antenatal care services among slum residents in Addis Ababa, Ethiopia. *Ann Glob Health* 2016;82:527-28.

118. Aleemi AR, Khaliqui H, Faisal A. Challenges and Patterns of Seeking Primary Health Care in Slums of Karachi: A Disaster Lurking in Urban Shadows. *Asia Pac J Public Health* 2018;30:479-90.

119. Viramgami AP, Verma PB, Vala MC, et al. A Cross-Sectional Study to Assess Reproductive and Child Health Profile of Working Women Residing in Urban Slums of Rajkot City. *Indian J Community Med* 2019;44:313-16.

120. van der Heijden J, Gray N, Stringer B, et al. 'Working to stay healthy', health-seeking behaviour in Bangladesh's urban slums: a qualitative study. *BMC Public Health* 2019;19:600.

121. Gaiha SM, Gillander Gadin K. 'No time for health:' exploring couples' health promotion in Indian slums. *Health Promot Int* 2020;35:70-81.

122. Wingfield T, Tovar MA, Huff D, et al. Socioeconomic support to improve initiation of tuberculosis preventive therapy and increase tuberculosis treatment success in Peru: a household-randomised, controlled evaluation. *Lancet* 2017;389:S16.

123. Muralidharan A. Constrained Choices? Menstrual Health and Hygiene Needs Among Adolescents in Mumbai Slums. *Indian J Gender Stud* 2019;26:12-39.

124. MacPherson P, Khundi M, Nliwasa M, et al. Disparities in access to diagnosis and care in Blantyre, Malawi, identified through enhanced tuberculosis surveillance and spatial analysis. *BMC Med* 2019;17:21.

For peer review only

For peer review only - http://bmjopen.bmj.com/site/about/guidelines.xhtml

125. Cernauskas V, Angeli F, Jaiswal AK, et al. Underlying determinants of health provider choice in urban slums: results from a discrete choice experiment in Ahmedabad, India. *BMC Health Serv Res* 2018;18:473.

126. Schultz JS, Muema S, Ouma A, et al. Timeliness of vaccination of vacination in an urban slum in Nairobi, Kenya. *Am J Trop Med Hyg* 2017;95:567.

127. Sharma S, Sarathi Mohanty P, Omar R, et al. Determinants and Utilization of Maternal Health Care Services in Urban Slums of an Industrialized City, in Western India. *J Family Reprod Health* 2020;14:95-101.

128. Ahmed S, Adams AM, Islam R, et al. Impact of traffic variability on geographic accessibility to 24/7 emergency healthcare for the urban poor: A GIS study in Dhaka, Bangladesh. *PLoS One* 2019;14:e0222488.

129. Muhammad A, Khan U, Yazdani AT, et al. Unveiling and addressing implementation barriers to routine immunization in the peri-urban slums of Karachi, Pakistan: a mixed-methods study. *Health Res Policy Syst* 2021;19:55.

130. Williams A, Sarker M, Ferdous ST. Cultural Attitudes toward Postpartum Depression in Dhaka, Bangladesh. *Med Anthropol* 2018;37:194-205.

131. Athie K, Dowrick C, Menezes AL, et al. Anxious and depressed women's experiences of emotional suffering and help seeking in a Rio de Janeiro favela. *Cien Saude Colet* 2017;22:75-86.

132. Kuria N, Reid A, Owiti P, et al. Compliance with follow-up and adherence to medication in hypertensive patients in an urban informal settlement in Kenya: comparison of three models of care. *Trop Med Int Health* 2018;23:785-94.

133. Mataboge MLS, Beukes S, Nolte AGW. The experiences of clients and healthcare providers regarding the provision of reproductive health services including the prevention

For peer review only - http://bmjopen.bmj.com/site/about/guidelines.xhtml

of HIV and AIDS in an informal settlement in Tshwane. *Health SA Gesondheid* 2016;21:67-76.

134. Otieno PO, Wambiya EOA, Mohamed SM, et al. Access to primary healthcare services and associated factors in urban slums in Nairobi-Kenya. *BMC Public Health* 2020;20:981.

135. Kardalkar S, Sherkhane MS. Utilization of antenatal care package among women of urban slums. *Indian J Public Health Research and Development* 2020;11:517-23.

136. Abd El Fatah SAM, El Habashy EM, Ismail HAH. Role of receipt of antenatal care in subsequent contraceptive use at primary health care centres serving slum areas of Cairo, Egypt. *Eur J Contracept Reprod Health Care* 2019;24:356-61.

137. Sharma S, Verma PB, Viramgami AP, et al. Analysis of Out-of-Pocket Expenditure in Utilization of Maternity Care Services in Urban Slums of Rajkot City, Gujarat. *Indian J Community Med* 2018;43:215-9.

138. Wekesah FM, Kyobutungi C, Grobbee DE, et al. Understanding of and perceptions towards cardiovascular diseases and their risk factors: a qualitative study among residents of urban informal settings in Nairobi. *BMJ Open* 2019;9:e026852.

139. Kalyango E, Kananura RM, Kiracho EE. Household preferences and willingness to pay for health insurance in Kampala City: a discrete choice experiment. *Cost Eff Resour Alloc* 2021;19:21.

140. Odhiambo GO, Musuva RM, Odiere MR, et al. Experiences and perspectives of community health workers from implementing treatment for schistosomiasis using the community directed intervention strategy in an informal settlement in Kisumu City, western Kenya. *BMC Public Health* 2016;16:986.

141. Patil SK, Ahmed MM. Assessment of integrated child development services in urban

For peer review only - http://bmjopen.bmj.com/site/about/guidelines.xhtml

slums of Belagavi city, Karnataka. *Indian J Public Health Res Dev* 2016;7:208-11.

142. Agonigi RC, Carvalho SM, Freire MAM, et al. The production of care in the routine of Family Health Teams. *Rev Bras Enferm* 2018;71:2659-65.

143. Banerjee S, Selvaraj K, Bandyopadhyay K, et al. Urban health and nutrition day or only immunisation day? barriers and bottlenecks in implementing Urban health and nutrition day in an Urban primary health centre of Nagpur, Central India. *J Mother Child* 2021;25:51-60.

144. Das Gupta M, Dasgupta R, Kugananthan P, et al. Flies without Borders: Lessons from Chennai on Improving India's Municipal Public Health Services. *J Dev Stud* 2020;56:907-28.

145. Prado Junior JC, Virgilio TC, Medronho Rde A. Cure rates for tuberculosis in the municipality of Rio de Janeiro, Brazil, in 2012 compared with coverage by, and time of establishment of, Family Health units, and socio-economic and demographic factors. *Ciencia & Saude Coletiva* 2016;21:1491-8.

146. Ongarora D, Karumbi J, Minnaard W, et al. Medicine Prices, Availability, and Affordability in Private Health Facilities in Low-Income Settlements in Nairobi County, Kenya. *Pharmacy (Basel)* 2019;7:40.

147. Liu Y, Kong Q, Yuan S, et al. Factors influencing choice of health system access level in China: A systematic review. *PLoS One* 2018;13:e0201887.

148. Banke-Thomas OE, Banke-Thomas AO, Ameh CA. Factors influencing utilisation of maternal health services by adolescent mothers in Low-and middle-income countries: a systematic review. *BMC Pregnancy Childbirth* 2017;17:65.

149. Mendoza-Sassi R, Béria J. Health services utilization: a systematic review of related factors. *Cad Saude Publica* 2001;17:819-32.

For peer review only

150. Ministerial Leadership Initiative for Global Health. Reducing Financial Barriers to Reproductive Health Care: Experiences with Free Care and Health Insurance 2010. Available from: https://www.aspeninstitute.org/wp-content/uploads/files/content/docs/pubs/mli_issue_brief_reducing_financial_barriers_to_reproductive_health_care.pdf [Accessed 10 Jun 2021]

151. Haw NJL, Uy J, Ho BL. Association of SHI coverage and level of healthcare utilization and costs in the Philippines: a 10-year pooled analysis. *J Public Health (Oxf)* 2020;42:e496-e505.

152. Lilford RJ, Oyebode O, Satterthwaite D, et al. Improving the health and welfare of people who live in slums. *Lancet* 2017;389:559-70.

153. World Health Organization. Strengthening Health Systems to Improve Health Outcomes, 2007. Available from: https://apps.who.int/iris/handle/10665/43918 [Accessed 25 Dec 2021]

154. McIntyre D, Thiede M, Birch S. Access as a policy-relevant concept in low- and middle-income countries. *Health Econ Policy Law* 2009;4:179-93.

For peer review only - http://bmjopen.bmj.com/site/about/guidelines.xhtml

Figure legends.

Figure 1. Preliminary framework for factors influencing slum residents' healthcare seeking behaviour and utilization of health services and the provision of services in slum settings

Figure 2. Updated framework of factors influencing healthcare-seeking behaviour/healthcare utilisation/provision of healthcare services in slums.

Figure 3. Flowchart

For peer review only

For peer review only - http://bmjopen.bmj.com/site/about/guidelines.xhtml

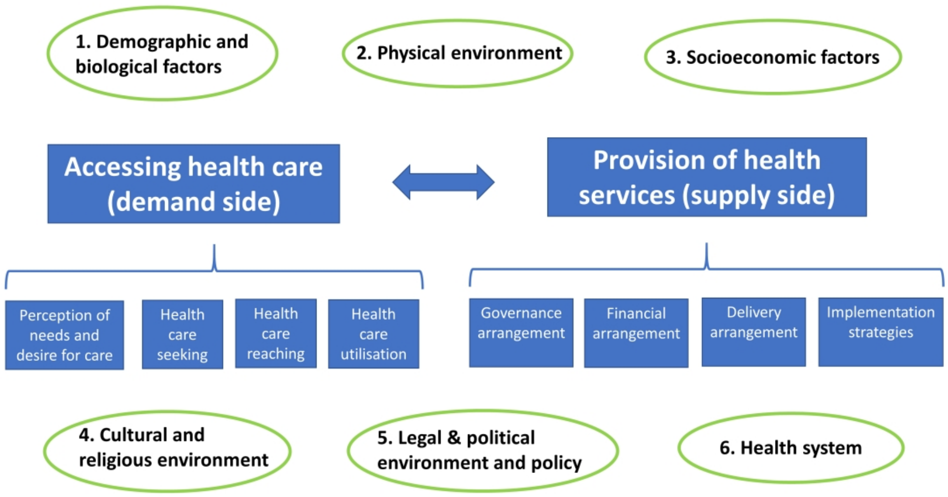

Preliminary framework for factors influencing slum residents' healthcare seeking behaviour and utilization of health services and the provision of services in slum settings

338x190mm (300 x 300 DPI)

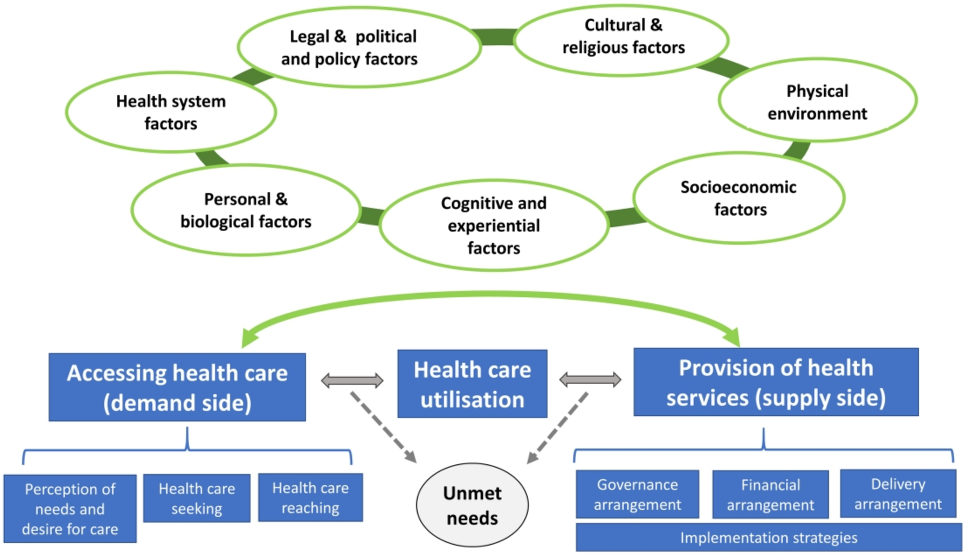

Updated framework of factors influencing healthcare-seeking behaviour/healthcare utilisation/provision of healthcare services in slums.

338x190mm (300 x 300 DPI)

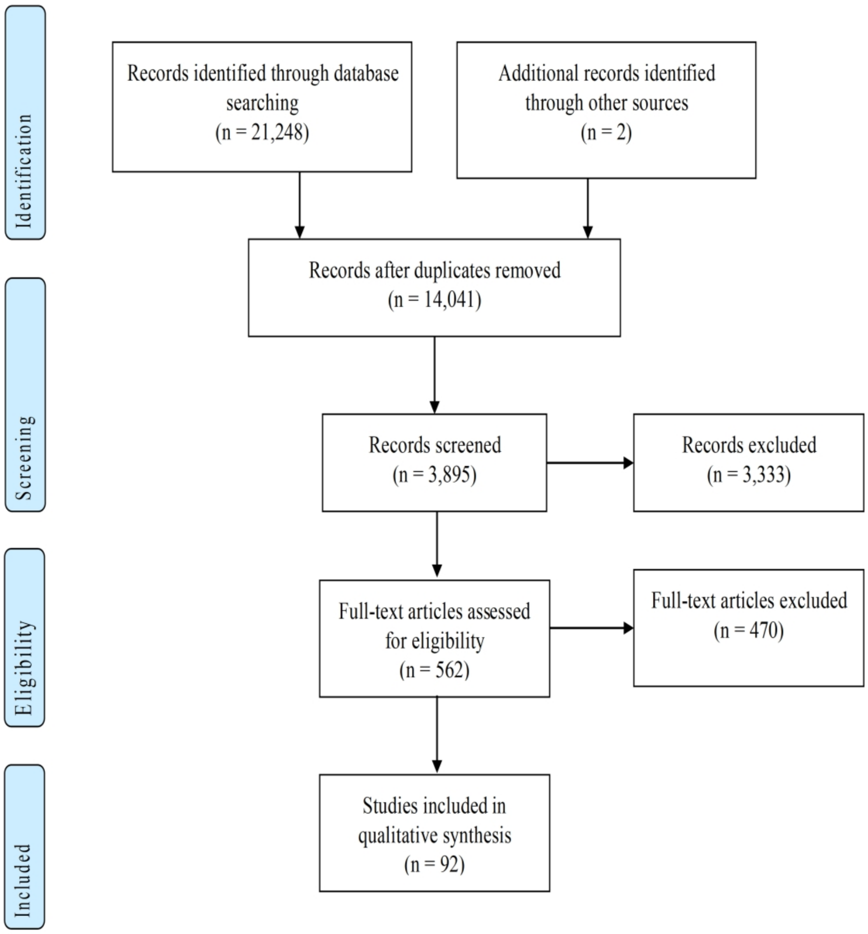

Flowchart

338x451mm (300 x 300 DPI)

Supplement 1. Healthcare-seeking behaviours of slum residents reported by included studies and associated factors.

Subcategory	Author (year)	Participants	Country	Study design	Methodology	Outcome	Factors of interest
General healthcare seeking behaviour	**Pakhare (2021)[55]**	**Slum residents diagnosed hypertension or diabetes**	**India**	**Prospective study**	**Quantitative**	**Linking to healthcare facilities**	**Age; wealth; distance to facilities ; early engagement by healthcare workers**
	Gaiha (2020)[121]	Hetero-couples in slums	India	Cross-sectional study	Mixed method	Ability to attend any health promotion activity	Lack of time related to work as a reason for low male participation
	van der Heijden (2019)[120]	Female workers and key informants in slums	Bangladeshi	Cross-sectional study	Qualitative	Healthcare-seeking behaviour	Competing interest (ability to work and income)
	Aleemi (2018)[118]	Slum residents	Pakistan	Cross-sectional study	Quantitative	Healthcare-seeking behaviour	Household income; government facility; NGO facility
	Wekesah (2019)[139]	Slum residents	Kenya	Cross-sectional study	Qualitative	Care-seeking and adherence to treatment for CVD	Cost of healthcare; lack of healthcare facilities
	Kar (2017)[58]	Slum residents	India	Cross-sectional study	Quantitative	Undiagnosed hypertension	Sex; poverty; unskilled laborer; literacy
	Mistry (2016)[71]	TB patients in slums	India	Retrospective study	Quantitative	Delays in care seeking	Perception of symptoms; home remedies; not want to miss work; provider shopping; delay in advising TB-relevant tests; referral.
	Kulkarni (2016)[70]	Women in slums	India	Cross-sectional study	Quantitative	Participation in breast cancer screening	Age; education; religion; Mother tongue; occupation; marital status; tobacco habits; family history of cancer; history of cancer screening
	Misra (2017)[101]	Slum households	India	Cross-sectional study	Quantitative	Health-seeking practice for cataract	Lack of time, fear of surgery, financial difficulties

For peer review only

	Ramagiri (2020)[77]	Slum residents with diabetes	India	Case control study	Mixed-method	Uptake of diabetic retinopathy screening	Realization of consequences of disease; travel assistance and proximity of the screening facility; absence of an accompanying person; cost
Healthcare for children	**Mohanty (2021)[54]**	**Caregivers of under-five children in urban slums,**	**India**	**Cross-sectional study**	**Quantitative**	**Healthcare seeking for children**	**Sex of child; size of the household; social group of caregiver, mother with mass media knowledge; age of mother; education and occupation of mother; suffering from chronic disease; decision making person for seeking health care; time lapse in approaching the health care facility; income loss due to children illnesses**
	Lungu (2020)[53]	Caregivers of children under 5 years of age in slums	Malawi	Cross-sectional study	Quantitative	Healthcare-seeking behaviour	Age; illness was perceived to be severe; fever; home management of childhood illness
						Timely healthcare seeking behaviour	Home management of childhood; knowledge of caregivers about child danger signs
	McNairy (2019)[19]	Slum households with children ≤ 5 years old	Haiti	Cross-sectional study	Quantitative	Healthcare access	Inability to afford care
	Hutain (2019)[100]	Caregiver at the time of the child's death in slums	Sierra Leone	Cross-sectional study	Mixed-method	Health care-seeking	Use of traditional medicine; difficultly reaching the health facility; doubts about need for medical care; mistreatment by staff
	Kerai (2019)[56]	Caregiver of children aged 2 months to 5 years in slums	Pakistan	Cross-sectional study	Quantitative	Healthcare-seeking behaviour	Age of child; gender of child; income; education of caretaker; vaccine awareness; breastfeeding awareness;

For peer review only

							presence of symptoms such as fever, tachypnea, chest indrawing, persistent vomiting, recurrent illness.
	Lungu (2018)[110]	Caregivers of children under 5 years of age in slums	Malawi	Prospective study	Quantitative	Healthcare-seeking behaviour	Cost; waiting time; availability of medicines and supplies; attitude of health workers; thorough examination of the child
						Willingness to pay for the health facility	Waiting time; availability of medicine and equipment; superficial or thorough examination; attitude of health workers
	Kamati (2019)[73]	Slum residents	Namibia	Cross-sectional study	Mixed-method	Self-medication	Perceived diagnosis as "minor or mild"; waiting times and queues to receive care
	Mishra (2017)[65]	Mothers living in slums with a child and migrated recently	India	Cross-sectional study	Quantitative	Healthcare seeking behaviour	Symptoms and severity
	Lungu (2016)[85]	Caregivers and health providers in slums	Malawi	Longitudinal study	Qualitative	Healthcare-seeking behaviour	Home management; lack of medicines and supplies; waiting times; facility opening times; attitude of health workers; suboptimal examination of the sick child; distance to health facility; cost of healthcare
Healthcare for women	Muralidharan (2019)[123]	Girls and mothers in slums	India	Cross-sectional study	Qualitative	Healthcare-seeking behaviour	Proximity of healthcare facilities
	Nasrin (2019)[111]	Married women with a child in slums	Bangladesh	Cross-sectional study	Mixed-method	Healthcare-seeking behaviours	Inability to spend the treatment cost

For peer review only

	Jayaweera (2018)[79]	Girls and women in slums	Kenya	Cross-sectional study	Qualitative	Access to contraception and abortion in health facilities	Stigma; lack of education about safe methods of abortion; perceived illegality of abortion; limited access to services because of financial barrier; fear of mistreatment and mistrust of health providers/facilities; geographical proximity
	Williams (2018)[130]	Mothers and medical personnel in slums	Bangladesh	Cross-sectional study	Qualitative	Mental healthcare seeking	Culture and stigma
	Ilankoo (2018)[78]	Women in slums	Sri Lanka	Cross-sectional study	Qualitative	Health-seeking behaviours related to vaginal discharge	Confusion in differentiating normal from abnormal vaginal discharge; effects on day-to-day life; confusion toward the causative factors; difficulties in disclosing; neglecting behaviours; and socio-cultural influences toward health-seeking behaviours.
	Athie (2017)[131]	Anxious and depressed women in slums	Brazil	Cross-sectional study	Qualitative	Healthcare seeking behaviour	High medical turnover and overload of healthcare providers
	Sudhinaraset (2016)[90]	Mothers and their families in slums	India	Cross-sectional study	Qualitative	Maternal health services and delivery experiences	Financial barriers; disrespectful care
	Pune Municipal corporation[38]	Recently delivered slum residents	India	Cross-sectional study	Mixed-method	Seeking front-line worker during labor	No time to call; family did not allow; being out of town; lack of trust; delivery at night
						Going to the Referred Place for Pregnancy Complications	Not necessary; family did not allow; lack of trust/poor quality services; don't like going to a difference facility; too far; cost; no transportation; private hospital
Preference for	Das (2018)[102]	Slum	India	Cross-	Qualitative	Healthcare-seeking	Female prefer informal healers

For peer review only

healthcare providers		residents		sectional study		practice (preference for formal/informal healers)	(cultural competency of care, easy communication, gender-induced affordability, avoidance of social stigma and labelling, living with the burden of cultural expectations and geographical and cognitive distance of formal health care)
							Male prefer formal care (ease of access, quality of treatment, expected outcome of therapies)
	Angeli (2018)[104]	Slum residents	India	Cross-sectional study	Mixed-method	Choice between public or private hospital	Bottom-of-the pyramid patients visit a public hospital more than top-of-the-pyramid patients
**Health insurance**	**Kalyango[*] (2021)[137]**	**Households in slum and non-slums**	**Uganda**	**Cross-sectional study**	**Qualitative**	**Willingness to pay for health insurance**	**Public and private providers; extended family enrolment**
HIV testing	Thomson (2018)[72]	Stakeholder including residents and healthcare service provider	Kenya	Cross-sectional study	Qualitative	HIV testing	Denial; complacency; fear of death; anticipation of unbearable stress; felt ill; had a partner die; learned that their partner was HIV-positive.
Expenditure	Mishra (2017)[59]	Slum households with a child aged 0–14 years and who had migrated within the last 12 years	India	Cross-sectional study	Quantitative	Treatment-seeking behaviour	Child's gender

[*]**Factors reported in the study were associated with participants covering both slum and non-slum residents.** CVD: cardiovascular disease; HIV: human immunodeficiency virus; NGO: non-governmental organization; TB: tuberculosis.

For peer review only

Supplement 2. Healthcare utilisation of slum residents reported by included studies and associated factors

Subcategory	Author (year)	Participants	Country	Study design	Methodology	Outcome	Factors of interest
General utilisation	**Wambiya (2021)[64]**	**Slum household members**	**Kenya**	**Cross-sectional study**	**Quantitative**	**Private and public healthcare utilisation**	**Public- satisfaction with cost; satisfaction with healthcare quality; having acute infection or other diseases**<br><br>**Private- insurance coverage; having acute infection**
	**Chauhan (2020)[96]**	**Elderly slum residents**	**India**	**Cross-sectional study**	**Quantitative**	**Utilization of healthcare services**	**Unawareness of healthcare facilities; behaviour of service providers; distance from home; transport facility; amenities at healthcare facilities; convenience for attendants**
	**Otieno (2020)[134]**	**Slum household members**	**Kenya**	**Cross-sectional study**	**Quantitative**	**Access to primary healthcare services**	**Sex of household head; average out-of-pocket healthcare expenditure; source of primary care**
	**Vora (2020)[46]**	**Slum household members**	**India**	**Cross-sectional study**	**Quantitative**	**Unmet need for surgical services**	**Financial reasons; lack of trust; age; sex; type of problem**
	Agrawal (2019)[115]	Older adults in slums	India	Cross-sectional study	Quantitative	Utilisation of welfare schemes	Religion; Caste; education;
	Ahmed (2019)[128]	N/A	Bangladesh	Cross-sectional study	Quantitative	Access to, and availability of healthcare services	Variability in traffic congestion
	Madan (2019)[87]	Female slum residents	India	Cross-sectional study	Qualitative	Access to primary care	Long waiting times and opening times of the primary health care; quality of services; satisfaction with treatments; home remedies; cost; rude attitude of healthcare providers
	Owiti (2018)[86]	Pregnant women in slums	Kenya	Cross-sectional	Quantitative	Utilisation of maternal health	Perception about public health facility delivery; living within

For peer review only

			study		services in public health facilities	close proximity; waiting time at the facility; learning about the program; quality of service; ANC attendance at a private and a non-profit health facility
Castiglione (2018)[112]	Slum residents	Brazil	Cross-sectional study	Qualitative	Barrier to healthcare services	*Public healthcare services:* structural aspects of the healthcare system in their community as a whole, such as scarcity of personnel and equipment, or long waiting periods; experiences of conflict when dealing with doctors and other professionals of the public healthcare system<br><br>*Private healthcare services*: Insufficient funds to seek assistance; services or products in the private sector;
Tabrizi* (2018)[114]	Households in slum and non-slums	Iran	Cross-sectional study	Quantitative	Utilisation of health services	High cost of services
					Home care services	High cost of services
					Not taking drugs prescribed	Slums:<br>financial problems<br><br>Non-slums:<br>getting better/feeling well
Wairiuko (2017)[88]	Elderly in slums	Kenya	Cross-sectional study	Mixed-method	Health service utilisation	Family support; satisfaction with healthcare services; gender healthcare worker preference; services by community health worker
Owusu-Ansah (2016)[83]	Slum residents	Ghana	Cross-sectional study	Qualitative	Utilization of healthcare	Education; occupation; NHIS membership; knowledge of symptom; overall knowledge

For peer review only

						score; transportation
Adane (2017)[81]	Mothers/caregivers of under-five children in slums	Ethiopia	Cross-sectional study	Quantitative	Utilization of healthcare facilities in children with diarrhoea	Mothers/caregivers education; occupation; time of walking to the nearest health facility; household monthly income; recognized danger signs
MacPherson (2019)[124]	Slum residents	Malawi	Prospective study	Quantitative	Access to TB diagnosis	Distance to the nearest TB registration clinic
Wingfield (2017)[122]	Slum households with patients treated for TB	Peru	Randomized controlled study	Quantitative	Initiation of TB preventive therapy	Socioeconomic support and social support
Iberico (2016)[99]	Healthcare workers and community members in slums	Peru	Cross-sectional study	Qualitative	Utilization of TB preventive therapy	Misunderstanding and fear of treatment
Snyder* (2016) [52]	TB patients living in slum and non-slum	Brazil	Retrospective study	Quantitative	Abandonment of TB treatment	Residency in a slum; sex; age; extrapulmonary clinical disease; HIV/AIDS; interaction (directly observed treatment × residency in a slum)
Oluoch (2017)[97]	Slum residents	Nairobi	Cross-sectional study	Quantitative	Attendance to HIV testing and counselling services	Previous test experience
Martinez Perez (2016)[89]	Healthcare workers and community members in slums	South Africa	Cross-sectional study	Mixed method	HIV Counselling and Testing	Fear; lack of trust
Amiresmaili (2019)[18]	Slum residents	India	Cross-sectional study	Quantitative	Utilisation of outpatients services	Gender; marital status
					Utilisation of inpatients services	Age of household head; marital status; insurance
Horng (2019)[49]	Slum households with children under 5 years old who either recently	Bangladesh	Cross-sectional study	Quantitative	Healthcare utilisation in severe acute respiratory illness	Relocation; age of child; education of mother; household wealth; health service knowledge

For peer review only

		relocated <12 months or who were residentially stable living >24 months				Full vaccination coverage	Relocation; number of children in household; age of child; education of mother; occupation of household head; household wealth; health service knowledge
	Kuria (2018)[132]	Patients received hypertension treatment in slums	Kenya	Retrospective study	Quantitative	Compliance with hypertensive treatment	Health facility group than walkway or weekend clinic attenders
	Cernauskas (2018)[125]	Slum residents	India	Cross-sectional study	Quantitative	Health provider choice	Distance to health facilities; friendly attitude of healthcare workers; appropriate service; familiarity
	Kaba (2020)[74]	Stakeholders (community members, community opinion leaders, health professionals, health office representatives.)	Ethiopia	Cross-sectional study	Qualitative	Utilisation of health services	Individual level: awareness about health problems; competing priorities; capacity to pay for services when referred.
	Mataboge (2016)[133]	Health services' clients and healthcare providers in an informal settlement	South Africa	Cross-sectional study	Qualitative	Healthcare utilisation	Long waiting time
Immunisation	**Muhammad (2021)[129]**	**Caregivers of children, community influencers, immunisation staff in peri-urban slums**	**Pakistan**	**Cross-sectional study**	**Mixed-method**	**Childhood vaccination**	**Permission for immunisation by decision-maker; lack of knowledge and awareness of the benefit of immunisation; misconceptions and fears regarding vaccines; social and religious barriers**
	**de Araujo Veras (2020)[45]**	**Children in slums**	**Brazil**	**Cross-sectional study**	**Quantitative**	**Childhood vaccination**	**Age of child: mother's education**

For peer review only

**Mutua (2020)[106]**	**Children in slums**	**Nairobi**	**Prospective study**	**Quantitative**	**Full and on-time vaccination coverage**	**Place of residence; wealth**
**Roja (2020)[44]**	**Mothers of children in slums**	**India**	**Cross-sectional study**	**Quantitative**	**Immunisation status of children**	**Number of children in family; age of child; father's education**
Obanewa (2020)[60]	Rural/urban formal/slum residents	Nigeria	Retrospective cross-sectional study	Quantitative	Fully-immunized child coverage	For slums:<br>delivery place; maternal education; birth order; antenatal attendance; religion<br><br>For slum and non-slums:<br>year; birth order; antenatal attendance; maternal education; religion; maternal age at child's birth; media exposure; region of the country; interaction between place of residence and place of delivery
Viramgami (2019)[119]	Married slum residents in reproductive age	India	Cross-sectional study	Quantitative	Vaccination status of child	Mother's employment
Singh (2018)[68]	N/A	India	Literature review	-	Childhood vaccination	Fear of adverse events; lack of information/knowledge; disease not harmful/serious; parents busy; income; mother's education; travel/transfer/migration; unawareness of need for health services; faith in immunisation; mother ill; forgetfulness; lack of initiative; family problems; services not available/lack of facility; shortages/reluctant to open 10 dose vials for 1 or 2 infants; current/history of sickness lead to withhold the vaccine

For peer review only

Pugliese-Garcia (2018)[76]	Stakeholders including slum residents, healthcare workers, health committee members, vaccinators	Zambia	Cross-sectional study	Qualitative	Vaccine hesitancy	Traditional remedies; alcohol use; religious beliefs; distrust towards western medicine; previous adverse events; fear of injections and low perceived need for immunisation; limited understanding of how vaccines work; overlapping local terms for vaccine; pain; perceived risk of infection
Manandhar (2018)[93]	Slum household with children age of 12-60 months	Nepal	Cross-sectional study	Quantitative	Incomplete immunisation	Knowledge on immunisation schedule
Dasgupta (2018)[116]	Slum household with children aged 0-59 months, resides in the study area for the past 12 months	India	Cross-sectional study	Quantitative	Vaccine hesitancy	Family type; education of mother
Lae (2018)[50]	Caregivers in slums	Myanmar	Cross-sectional study	Qualitative	Utilisation of immunisation services	Age of child; income; migration; antenatal visit; receiving additional vaccines before; having immunisation card.
Schultz (2017)[126]	Parents with children <5 years old in slums	Kenya	Prospective study	Quantitative	Timeliness of vaccination	Close to the clinic; birth in December
Crocker-Buque (2017)[21]	People living in a low-income urban area or slum in a low-middle income countries	Multiple nations	Systematic review	-	Immunisation coverage	*Socioeconomic and demographic characteristics:* socioeconomic status; wealth; parents' literacy; mother's education; employment; residential status; place of residence; place of delivery; household visit by health workers; premature birth; malnourishment; inadequate housing; poor

						prenatal care; ethnicity; age; maternal age; birth order; sex of child; number of children<br><br>*Migration status:*<br>migration; recent migration<br><br>*Information, beliefs and behaviour:*<br>unaware of the need for vaccines; unaware of clinic location or timing; maternal knowledge of immunisation; lack of access to information; parents being too busy; return to home village; difficulty in accessing services; fear of side effects; attitude of health workers; concerns over cost; being suspicious of free services<br><br>*Health services:*<br>distance from health centre; timing of services; fear of costs; risk of lost income; lack of local knowledge; patients' satisfaction; provision of accurate information; accessing pre-natal care
Shrestha (2016)[82]	Slum households with children aged 12–23 months.	Nepal	Case-control study	Quantitative	Incompletion of immunisation	Home delivery; type of residence; knowledge about healthcare services of primary care-taker; perception towards healthcare services, conflicting priorities, side effect
Devasenapathy (2016)[57]	Slum household with children aged between 12 and 42 months	India	Cross-sectional study	Quantitative	Childhood complete immunisation	Sex; mother's literacy; place of birth; place of childbirth; religion; socioeconomic position; birth certificate

For peer review only

Maternal	**Sendo (2021)[92]**	**Female slum residents**	**Ethiopia**	**Cross-sectional study**	**Qualitative**	**Delivery in healthcare facilities**	**Provision of quality, respectful and dignified midwifery care; lack of awareness about facility delivery.**
	**Kardalkar (2020)[135]**	**Female delivered within three months in slums**	**India**	**Cross-sectional study**	**Quantitative**	**Utilization of antenatal care**	**Literacy; Gravida; occupation**
	**Sendo (2020)[91]**	**Women of reproductive age in slums**	**Ethiopia**	**Cross-sectional study**	**Qualitative**	**Delivery in health facilities**	**Perceived benefits of home delivery; knowledge deficit about health facility-based delivery; poor access to healthcare facilities; inadequate resources**
	**Sharma (2020)[127]**	**Women delivered a baby within one year in slums**	**India**	**Cross-sectional study**	**Quantitative**	**Utilization of maternal healthcare services**	**Education; employment of mother; category and type of family; distance and time to reach health facility;**
	**Yadav (2020)[42]**	**Married women in slums**	**India**	**Cross-sectional study**	**Quantitative**	**Unmet need for family planning services**	**Age; educational status; duration of marriage; number of pregnancies; knowledge of contraceptive methods; opposition to contraceptive use; contact with a midwife**
	Razzaque (2020)[66]	Slum residents	Bangladeshi	Cross-sectional study	Quantitative	Healthcare utilisation	Recent migration; wealth; education; employment
	Getachew (2020)[113]	Slum households	Ethiopia	Cross-sectional study	Quantitative	Delivery in healthcare facilities	Perceived as not customary to deliver at health facility; not necessary; unavailability of female birth attendants; perceived quality of services; cost
	Shrestha (2019)[61]	Mothers with infant residing in slums	Nepal	Cross-sectional study	Quantitative	Utilisation of antenatal and delivery services	Educational status of respondents and their husbands; number of pregnancy

For peer review only

					Institutional delivery	Educational status; occupation of husband; number of pregnancy
					Postnatal visit	Occupation of husband
					Utilisation of family planning services	Occupation of husband
					Tetanus Toxoid immunisation	Educational status of respondents; economic status; knowledge about healthcare services; educational status of husband; number of pregnancies
Atusiimire (2019)[98]	Mothers delivered in the past one year in slums	Uganda	Cross-sectional study	Quantitative	Facility based–deliveries	Exposure to media concerning facility delivery; frequency of ANC; timing of 1st ANC
Upadhyai (2019)[39]	Recently delivered mothers residing in slums	India	Cross-sectional study	Quantitative	Healthcare utilisation	Age; education of mother and father; socioeconomic class; antenatal check-ups; institutional delivery services; family type; caesarean delivery; complication or perceived health problem
Angeles* (2019)[47]	Slum and non-slum residents	Bangladesh	Prospective study	Quantitative	Use of modern contraceptive methods	Parity, mother's age; mother's education, socioeconomic status, interaction (slum × time period)
					Delivery by skilled birth attendant	Residing in slums, parity, mother's age, mother's education, length of stay in current city of residence, socioeconomic status, number of available community health worker, distance from health facility, interaction (slum x time period)
Kusuma (2018)[80]	Recent migrant and settled mothers with a	India	Cross-sectional study	Quantitative	Birth in health facility	Listening to radio; number of ANC visits; plan for hospital birth; plan for transport; some

For peer review only

For peer review only - http://bmjopen.bmj.com/site/about/guidelines.xhtml

	child up to the age of 1 year in slums					danger sign; knowledge of danger sign
Sharma (2018)[138]	Women living in urban slums and delivered a baby within 1 year	India	Cross-sectional study	Quantitative	Utilisation of maternal care services	Mode of delivery; hospital stay after delivery
Islam* (2018) [107]	Ever-married women aged 15-49 years living in slum and non-slum	Bangladesh	Cross-sectional study	Quantitative	ANC visits	Education; wealth index of the household
Geddam (2017)[67]	Rural to urban internal migrant mothers with a child of less than 2 years of age	India	Cross-sectional study	Quantitative	Utilisation of maternal health services	Education of the mother; family size; occupation of mother
					Delivery in institution	Educational status of mother; number of ANC visit; adequacy of ANC; migration status
Kaba (2017)[94]	Stakeholders including city administrators, community members, healthcare providers	Ethiopia	Cross-sectional study	Qualitative	Maternal health service utilisation	Lack of awareness and lack of perceived needs about available services; fear of stigma; competing priorities, social connectedness; perceived lack of respectful service providers; socio-cultural factors including socially sanctioned expectations
Verma (2017)[75]	Pregnant women and infants in slums	India	Case-control study	Mixed-method	Antenatal care registration/immunisation	Knowledge of healthcare services; perceived need for healthcare services; family support; fear; negative experience with previous vaccination
Sharma (2016)[51]	Married women in slums	Nepal	Cross-sectional study	Quantitative	Antenatal healthcare utilisation	Age; husband education; spouse occupation; family income; type of family; planned pregnancy; death of children
Jolly (2016)[108]	Married women with a pregnancy outcome in the previous year in	Bangladesh	Cross-sectional study	Quantitative	Antenatal care; birth assisted by medically trained provider; postnatal	Education; wealth

For peer review only

		slums				care; treatment seeking for delivery complications	
						Use of modern family planning	Wealth
	Tebekaw (2016)[117]	Women in slums	Ethiopia	Cross-sectional study	Quantitative	Antenatal care services	Education; private/public hospital
	Sadhna (2016)[109]	Married women in slums	India	Cross-sectional study	Quantitative	Utilisation of maternal health services	Education; Caste; wealth; distance to preferred health facility
	Neyaz (2016)[62]	Married women in slums	India	Cross-sectional study	Quantitative	Delivery in hospitals	Received ANC; number of ANC visits; education; birth order; living index
	Rahman (2016)[105]	Married women in rural and slum area	India	Cross-sectional study	Quantitative	Intrauterine contraceptive device utilisation	Income; occupation
	Sheehy (2016)[103]	Informant and women in slums	Myanmar	Cross-sectional study	Qualitative	Giving birth in hospital	Financial constraints; lack of transportation; sociocultural and financial considerations
Contraceptive	Renzaho (2017)[48]	Slum residents aged 13-24	Uganda	Cross-sectional study	Quantitative	Access to contraceptive services and family planning	Age; disability
	Abd El Fatah (2019)[136]	Married women aged 15–49 years in slums	Egypt	Cross-sectional study	Quantitative	Contraceptive use	Number of male children
Health insurance	**Iyalomhe (2021)[41]**	**Slum residents**	**Nigeria**	**Cross-sectional study**	**Quantitative**	**Healthcare insurance coverage**	**Age; sex; marriage; income; religion; education**
	**Mendhe (2021)[40]**	**Female slum residens**	**India**	**Cross-sectional study**	**Quantitative**	**Healthcare insurance coverage**	**Socioeconomic status;**
						**Out of pocket expenditure**	**Age; government/ private hospital**
	Otieno (2019)[84]	Slum residents	Kenya	Cross-sectional	Quantitative	Enrolment in a health insurance	Employment; source of primary care; satisfaction with cost of

For peer review only

				study		programme	care; satisfaction with procedure of care; perceived health status
	Kusuma (2018)[69]	Slum residents	India	Cross-sectional study	Quantitative	Health insurance possession	Residential background (old slums than new); migration period; possession of ration card; household size; occupation of household head
	Gupta (2017)[95]	Slum households having health insurance cards	India	Cross-sectional study	Mixed-method	Utilisation of healthcare insurance	Awareness of the empanelled hospitals; experiences of friends and relatives at national health insurance empanelled hospitals; hospitals refused to accept health insurance cards
Expenditure	Sahu (2017)[63]	Women delivered within a period of 6 weeks in slums	India	Cross-sectional study	Quantitative	Out-of-pocket expenditure for maternal and neonatal health services	Gravidity; type of delivery; place of delivery; morbidity
	Mishra (2017)[59]	Slum households with a child aged 0–14 years and who had migrated within the last 12 years	India	Cross-sectional study	Quantitative	Out-of-pocket expenditure	Child's gender; mother's education; type of illness

*Factors reported in the study were associated with participants covering both slum and non-slum residents. ANC: antenatal care; CVD: cardiovascular disease; HIV: human immunodeficiency virus; N/A: not applicable; NGO: non-governmental organization; TB: tuberculosis.

For peer review only

Supplement 3. Provision of healthcare services in slums examined by included studies and associated factors

Subcategory	Author (year)	Participants	Country	Study design	Methodology	Outcome	Factors of interest
General provision	**Banerjee (2021)[145]**	**Community-level service providers in the selected city of Nagpur, Maharashtra.**	**India**	**Cross-sectional study**	**Mixed-methods**	**Implementing urban health and nutrition day**	**Unserved areas and left-out urban slum pockets; the distribution paradox of Urban Health and Nutrition Day location with an ill-defined geographic boundary; restriction of range of services to antenatal registration and immunisation with gross neglect of other components; suboptimal training of staff; insufficient availability of space, logistics, and health manpower; non-involvement of community members and Urban Local Bodies; and poor monitoring and supervision.**
	**Muhammad (2021)[129]**	**Caregivers of children, community influencers, immunisation staff in peri-urban slums**	**Pakistan**	**Cross-sectional study**	**Mixed-method**	**Childhood vaccination**	**Underperformance of staff; unreliable immunisation and household data; inefficient utilization of funds; interference of polio campaigns with immunisation**
	Kaba (2020)[74]	Stakeholders (community members, community opinion leaders, Urban Health Extension	Ethiopia	Cross-sectional study	Qualitative	Provision of health services	Institutional-level: medical supplies; a lack of passion; attitudes on the part of health service providers<br><br>Community level:

For peer review only

	Professionals, and city health office representatives.)					shared understanding of the problems; services and the community's established values in relation to the problems and services.
Das Gupta (2020)[143]	N/A	India	Case study	Mixed-method	Improving public health services	Devolution of service delivery transferring funds and responsibilities to elected local bodies; management by professional managerial and technical cadres; Tight organisation of public health services; Professional support from the state directorate of public health
Ongarora (2019)[146]	Private healthcare facilities	Kenya	Cross-sectional study	Quantitative	Provision of medicine	Medicine price, affordability and availability of medicine
Agonigi (2018)[142]	Health professionals	Brazil	Cross-sectional study	Qualitative	Production of care in the daily work of health professionals	Issues related to assignment of tasks; inadequate space and equipment; requirement to follow standardised protocol; demands from the management; workload; environment (sanitation, territory); violence; registration
Odhiambo (2016)[140]	Community health workers	Kenya	Longitudinal study	Quantitative	Drug administration activities for schistosomiasis	Community health worker familiarity with households led to warm reception; good knowledge of intervention area by community health workers; high demand for drugs in the final year of treatment; effective community mobilization; opportunity to integrate mass drug administration with other health interventions; presence of community health workers and their supervisory structure, and points of referral for serious side

For peer review only

						effects; fear of side effects, size of tablet and misconceptions regarding treatment; unrelated death and the associated negative publicity by the media; religious beliefs and mistrust of interventions; insufficient time; absence of community members during the drug administration exercise; difficulty in directly observing treatment; unsanitary environmental conditions; inaccessibility (filthy and bush environment); demand for incentives by community members to take drugs.
Patil (2016)[141]	Healthcare service centres	India	Cross-sectional study	Quantitative	Services provided under Integrated Child Development Services	Lack of basic infrastructural facilities; absence of essential drugs, equipment and logistics; poor pay scale, untimely drug supply, poor community support, more of documentation work, increased work burden, lack of supportive staff and no incentives for the increased work
Mataboge (2016)[133]	Health services' clients and healthcare providers in an informal settlement	South Africa	Cross-sectional study	Qualitative	Provision of reproductive healthcare services	Healthcare policies; work overload; community-based care
Prado Junior (2016)[144]	New TB cases living in slum and non-slum	Brazil	Cross-sectional study	Quantitative	Coverage under Family Health system for TB patients	Policy prioritizing low social development areas

TB: tuberculosis

For peer review only

Appendix 1. Search strategy and the result of each database.

Database	Search strategy	Number of studies
Ovid Medline	1. (informal* and settlement*).ti,ab,kw.<br>2. (shanty and town*).ti,ab,kw.<br>3. (favela* or ghetto* or shantytown* or shanty-town* or slum or slums).ti,ab,kw.<br>4. or/1-3<br>5. limit 4 to english language	**4,688**
Embase	1 (informal* and settlement*).ti,ab,kw.<br>2 (shanty and town*).ti,ab,kw.<br>3 (favela* or ghetto* or shantytown* or shanty-town* or slum or slums).ti,ab,kw.<br>4 or/1-3<br>5 limit 4 to english language	**5,090**
Web of Science	1 (TS=(favela* OR ghetto* OR shantytown* OR shanty-town* OR slum OR slums)) AND language: (English)<br>2 ((TS=(informal* NEAR settlement*))) AND language: (English)<br>3 (TS=(shanty NEAR town*)) AND language: (English)<br>4 (#1 OR #2 OR #3) AND language: (English)	**3,553**
Cochrane	1 (informal* and settlement*).ti,ab,kw.<br>2 (shanty and town*).ti,ab,kw.<br>3 (favela* or ghetto* or shantytown* or shanty-town* or slum or slums).ti,ab,kw.<br>4 #1 or #2 or #3	**381**
CINAHL	1 TI (informal* and settlement*) OR AB (informal* and settlement*)<br>2 TI (shanty and town*) OR AB (shanty and town*)<br>3 TI (favela* or ghetto* or shantytown* or shanty-town* or slum or slums) OR AB (favela* or ghetto* or shantytown* or shanty-town* or slum or slums)<br>S1 OR S2 OR S3	**1,757**

For peer review only

Preferred Reporting Items for Systematic reviews and Meta-Analyses extension for Scoping Reviews (PRISMA-ScR) Checklist

SECTION	ITEM	PRISMA-ScR CHECKLIST ITEM	REPORTED ON PAGE #
**TITLE**			
Title	1	Identify the report as a scoping review.	1
**ABSTRACT**			
Structured summary	2	Provide a structured summary that includes (as applicable): background, objectives, eligibility criteria, sources of evidence, charting methods, results, and conclusions that relate to the review questions and objectives.	2
**INTRODUCTION**			
Rationale	3	Describe the rationale for the review in the context of what is already known. Explain why the review questions/objectives lend themselves to a scoping review approach.	4
Objectives	4	Provide an explicit statement of the questions and objectives being addressed with reference to their key elements (e.g., population or participants, concepts, and context) or other relevant key elements used to conceptualize the review questions and/or objectives.	5
**METHODS**			
Protocol and registration	5	Indicate whether a review protocol exists; state if and where it can be accessed (e.g., a Web address); and if available, provide registration information, including the registration number.	5
Eligibility criteria	6	Specify characteristics of the sources of evidence used as eligibility criteria (e.g., years considered, language, and publication status), and provide a rationale.	6-7
Information sources*	7	Describe all information sources in the search (e.g., databases with dates of coverage and contact with authors to identify additional sources), as well as the date the most recent search was executed.	5-6
Search	8	Present the full electronic search strategy for at least 1 database, including any limits used, such that it could be repeated.	5-6, appendix1
Selection of sources of evidence†	9	State the process for selecting sources of evidence (i.e., screening and eligibility) included in the scoping review.	5-7
Data charting process‡	10	Describe the methods of charting data from the included sources of evidence (e.g., calibrated forms or forms that have been tested by the team before their use, and whether data charting was done independently or in duplicate) and any processes for obtaining and confirming data from investigators.	7-9
Data items	11	List and define all variables for which data were sought and any assumptions and simplifications made.	7-9
Critical appraisal of individual sources of evidence§	12	If done, provide a rationale for conducting a critical appraisal of included sources of evidence; describe the methods used and how this information was used in any data synthesis (if appropriate).	N/A

For peer review only - http://bmjopen.bmj.com/site/about/guidelines.xhtml

SECTION	ITEM	PRISMA-ScR CHECKLIST ITEM	REPORTED ON PAGE #
Synthesis of results	13	Describe the methods of handling and summarizing the data that were charted.	7-9
**RESULTS**			
Selection of sources of evidence	14	Give numbers of sources of evidence screened, assessed for eligibility, and included in the review, with reasons for exclusions at each stage, ideally using a flow diagram.	10-11, Figure 1
Characteristics of sources of evidence	15	For each source of evidence, present characteristics for which data were charted and provide the citations.	Supplement 1-3
Critical appraisal within sources of evidence	16	If done, present data on critical appraisal of included sources of evidence (see item 12).	N/A
Results of individual sources of evidence	17	For each included source of evidence, present the relevant data that were charted that relate to the review questions and objectives.	Table 2-3
Synthesis of results	18	Summarize and/or present the charting results as they relate to the review questions and objectives.	13-25, Figure 2
**DISCUSSION**			
Summary of evidence	19	Summarize the main results (including an overview of concepts, themes, and types of evidence available), link to the review questions and objectives, and consider the relevance to key groups.	29-30
Limitations	20	Discuss the limitations of the scoping review process.	31
Conclusions	21	Provide a general interpretation of the results with respect to the review questions and objectives, as well as potential implications and/or next steps.	34
**FUNDING**			
Funding	22	Describe sources of funding for the included sources of evidence, as well as sources of funding for the scoping review. Describe the role of the funders of the scoping review.	35-36

JBI = Joanna Briggs Institute; PRISMA-ScR = Preferred Reporting Items for Systematic reviews and Meta-Analyses extension for Scoping Reviews.

* Where *sources of evidence* (see second footnote) are compiled from, such as bibliographic databases, social media platforms, and Web sites.

† A more inclusive/heterogeneous term used to account for the different types of evidence or data sources (e.g., quantitative and/or qualitative research, expert opinion, and policy documents) that may be eligible in a scoping review as opposed to only studies. This is not to be confused with *information sources* (see first footnote).

‡ The frameworks by Arksey and O'Malley (6) and Levac and colleagues (7) and the JBI guidance (4, 5) refer to the process of data extraction in a scoping review as data charting.

§ The process of systematically examining research evidence to assess its validity, results, and relevance before using it to inform a decision. This term is used for items 12 and 19 instead of "risk of bias" (which is more applicable to systematic reviews of interventions) to include and acknowledge the various sources of evidence that may be used in a scoping review (e.g., quantitative and/or qualitative research, expert opinion, and policy document).

From: Tricco AC, Lillie E, Zarin W, O'Brien KK, Colquhoun H, Levac D, et al. PRISMA Extension for Scoping Reviews (PRISMAScR): Checklist and Explanation. Ann Intern Med. 2018;169:467–473. doi: 10.7326/M18-0850.

For peer review only - http://bmjopen.bmj.com/site/about/guidelines.xhtml

BMJ Open

Factors associated with accessing and utilisation of health care and provision of health services for residents of slums in low and middle income countries: a scoping review of recent literature

Journal:	*BMJ Open*
Manuscript ID	bmjopen-2021-055415.R2
Article Type:	Original research
Date Submitted by the Author:	17-Mar-2022
Complete List of Authors:	Park, Ji-Eun; University of Warwick, Warwick Medical School; Korea Institute of Oriental Medicine<br>Kibe, Peter; African Population and Health Research Center, Health and Systems for Health<br>Yeboah, Godwin; University of Warwick, Institute for Global Sustainable Development<br>Oyebode, Oyinlola; University of Warwick Warwick Medical School,<br>Harris, Bronwyn; University of Warwick, NIHR Global Health Research Unit on Improving Health in Slums<br>Ajisola, Motunrayo ; University of Ibadan, Sociology<br>Griffiths, Frances; University of Warwick Warwick Medical School, ; University of the Witwatersrand, Centre for Health Policy<br>Aujla, Navneet; Newcastle University, Population Health Sciences Institute; University of Warwick, Division of Health Sciences<br>Gill, Paramjit ; University of Warwick<br>Lilford, RJ; University of Birmingham, Institute of Applied Health Research<br>Chen, Yen-Fu; University of Warwick, Division of Health Sciences
**Primary Subject Heading**:	Global health
Secondary Subject Heading:	Epidemiology, General practice / Family practice, Health policy, Health services research, Public health
Keywords:	HEALTH SERVICES ADMINISTRATION & MANAGEMENT, International health services < HEALTH SERVICES ADMINISTRATION & MANAGEMENT, PUBLIC HEALTH

SCHOLARONE™
Manuscripts

I, the Submitting Author has the right to grant and does grant on behalf of all authors of the Work (as defined in the below author licence), an exclusive licence and/or a non-exclusive licence for contributions from authors who are: i) UK Crown employees; ii) where BMJ has agreed a CC-BY licence shall apply, and/or iii) in accordance with the terms applicable for US Federal Government officers or employees acting as part of their official duties; on a worldwide, perpetual, irrevocable, royalty-free basis to BMJ Publishing Group Ltd ("BMJ") its licensees and where the relevant Journal is co-owned by BMJ to the co-owners of the Journal, to publish the Work in this journal and any other BMJ products and to exploit all rights, as set out in our licence.

The Submitting Author accepts and understands that any supply made under these terms is made by BMJ to the Submitting Author unless you are acting as an employee on behalf of your employer or a postgraduate student of an affiliated institution which is paying any applicable article publishing charge ("APC") for Open Access articles. Where the Submitting Author wishes to make the Work available on an Open Access basis (and intends to pay the relevant APC), the terms of reuse of such Open Access shall be governed by a Creative Commons licence – details of these licences and which Creative Commons licence will apply to this Work are set out in our licence referred to above.

Other than as permitted in any relevant BMJ Author's Self Archiving Policies, I confirm this Work has not been accepted for publication elsewhere, is not being considered for publication elsewhere and does not duplicate material already published. I confirm all authors consent to publication of this Work and authorise the granting of this licence.

For peer review only

Factors associated with accessing and utilisation of health care and provision of health services for residents of slums in low and middle income countries: a scoping review of recent literature

Ji-Eun Park,[1,2] Peter Kibe,[3] Godwin Yeboah,[4] Oyinlola Oyebode,[1] Bronwyn Harris,[1] Motunrayo Ajisola,[5] Frances Griffiths,[1,6] Navneet Aujla,[1,7] Paramjit Gill,[1] Richard Lilford,[8] Yen-Fu Chen,[1]* on behalf of the Improving Health in Slums Collaborative

Affiliations:

[1] Division of Health Sciences, Warwick Medical School, University of Warwick, Coventry, United Kingdom

[2] Future Medicine Division, Korea Institute of Oriental Medicine, Daejeon, Republic of Korea

[3] African Population and Health Research Center, Nairobi, Kenya

[4] Institute for Global Sustainable Development, University of Warwick, Coventry, United Kingdom

[5] College of Medicine, University of Ibadan, Ibadan, Nigeria

[6] Centre for Health Policy, School of Public Health, University of the Witwatersrand, Johannesburg, South Africa

[7] Population Health Sciences Institute, Newcastle University, Newcastle, United Kingdom

[8] Institute of Applied Health Research, University of Birmingham, Birmingham, United Kingdom

*Corresponding author: Dr Yen-Fu Chen, Division of Health Sciences, Warwick Medical School, University of Warwick, Coventry, CV4 7AL, UK.

Email: Y-F.Chen@warwick.ac.uk

Word count: 6,146

For peer review only
For peer review only - http://bmjopen.bmj.com/site/about/guidelines.xhtml

ABSTRACT

Objective: To identify factors associated with accessing and utilisation of healthcare and provision of health services in slums.

Design: A scoping review incorporating a conceptual framework for configuring reported factors.

Data sources: MEDLINE, Embase, CINAHL, Web of Science and the Cochrane Library were searched from their inception to December 2021 using slum related terms.

Eligibility criteria: Empirical studies of all designs reporting relevant factors in slums in low and middle income countries.

Data extraction and synthesis: Studies were categorised and data were charted according **to** a preliminary conceptual framework refined by emerging findings. Results were tabulated and narratively summarised.

Results: Of the 15,091 records retrieved from all years, 4,368 records dated between 2016-2021 were screened by two independent reviewers and 111 studies were included. The majority (63 studies, 57%) were conducted in Asia, predominantly in India. In total 104 studies examined healthcare access and utilisation from slum residents' perspective while only ten studies explored provision of health services from providers/planners' perspective (three studies included both). A multitude of factors are associated with accessing, utilising and providing healthcare in slums, including recent migration to slums; knowledge, perception and past experience of illness, healthcare needs and health services; financial constraint and competing priorities between health and making a living; lacking social support; unfavourable physical environment and locality; sociocultural expectations and stigma; lack of official recognition; and existing problems in the health system.

Conclusion: The scoping review identified a significant body of recent literature reporting

For peer review only

factors associated with accessing, utilisation and provision of healthcare services in slums. We classified the diverse factors under seven broad categories. The findings can inform a holistic approach to improving health services in slums by tackling barriers at different levels, taking into account local context and geospatial features of individual slums.

Systematic review registration: Open Science Framework (OSF, https://osf.io/694t2)

Keywords: slum, informal settlement, scoping review, healthcare-seeking behaviour, healthcare utilisation, health service delivery

Strengths and limitations of this study

- We conducted literature search in multiple databases using generic terms related to slums to ensure that a wide range of relevant studies was captured.
- A conceptual framework explaining factors associated with accessing and utilisation of healthcare by slum residents as well as provision of healthcare in slums was developed and used to categorise identified studies and factors.
- We examined barriers and facilitators of accessing healthcare and service provision from the perspectives of both demand side (slum residents) and supply side (healthcare providers and service planners).
- Only studies published in academic journals between 2016 and 2021 in English language were included, and methodological quality of each included study was not examined because of time constraint.
- We did not explore the complex relationships and interactions between various factors in different contexts at different slum locations, but our mapping of these factors to the conceptual framework should facilitate further in-depth analyses.

For peer review only - http://bmjopen.bmj.com/site/about/guidelines.xhtml

INTRODUCTION

Rapid urbanisation has resulted in a growing number of residents in slums [1] who face ongoing problems such as unemployment, poor sanitation, lack of transport, high level of crime, and haphazard development [2]. In 2018, over one billion people were living in slum-like conditions, and Central, South and South-East Asia and Sub-Saharan Africa accounted for 80% of them [1]. Even though various definitions of slums exist, there is no universally agreed definition of what constitutes 'a slum', and the term itself is widely debated and contested [3, 4]. For the purpose of this scoping review, we refer to slums as densely populated areas characterised by lack of basic services, substandard housing, overcrowding, unhealthy living condition, insecure tenure and poverty [4, 5], taking into account the crucial concepts of place and space that are important in shaping health outcomes and community access to health services in these urban settings [4].

Previous studies have reported various risk factors affecting health of slum residents such as physical environment [6], sanitation [7], social capital [8, 9] and water governance [10], and have observed in some cases that slum residents have worse health status compared to non-slum urban and/or rural residents. For example, Ezeh et al. found that children living in slums had higher mortality than rural and non-slum urban populations [3]. Poorer height-for-age for children [11] and higher prevalence of childhood illnesses and malnutrition [12] have also been observed in slums compared to non-slum urban and rural settings. In addition, slum residents are susceptible to unhealthy behaviours [13, 14]. Living in slums has been found to be associated with low physical activity [13], poor diet [14], and poor knowledge about the cause and preventability of diseases [15].

Despite the unfavourable health status and environment, and consequently the potential high level of healthcare needs, previous studies showed that slum residents were less likely to seek

For peer review only - http://bmjopen.bmj.com/site/about/guidelines.xhtml

and use healthcare services than their non-slum counterparts in the cities [16, 17]. Slum residents have been found to have lower rates of healthcare utilisation in antenatal services [16] and services for non-communicable diseases [17] compared to residents of urban 'formal' settings. One study in Iran showed that only about half of slum households that required outpatient services could use them [18]. Another study in Haiti also reported that one third of slum households were not able to access medical care for their children when it was needed in the past year [19].

While the health status and needs of slum residents have been described in previous reviews [3, 20], factors associated with healthcare seeking behaviour and healthcare utilisation of slum residents and factors related to the provision of health services in slums have not been systematically examined (with the exception of immunisation services) [21]. This scoping review aims to fill in these evidence gaps and inform efforts to improve healthcare delivery to people in slums.

METHODS

This scoping review was performed according to current best practice guidance [22]. The broad question of interest was: "*What factors are associated with slum residents'* ***accessing and utilisation*** *of health care and/or the provision of health services in slum settings in low and middle income countries (LMICs)*?" The protocol for this review was registered in Open Science Framework (OSF) [23].

Literature search and study selection

For peer review only - http://bmjopen.bmj.com/site/about/guidelines.xhtml

A broad search of five databases (MEDLINE, Embase, CINAHL, Web of Science and the Cochrane Library) was conducted in April 2020 and updated in December 2021. Searches were limited to English language. Key terms related to slums were used: slum or slums or ghetto or ghettos or informal settlement$ or shantytown$ or shanty town$ or favela$. (Appendix 1) We did not include terms related to other concepts in order to maximise the sensitivity of our searches. In addition, we searched organizational websites of Slum Dwellers International, UN HABITAT, UN and WHO but did not identify relevant studies [24-27].

Records retrieved from databases (after duplicates were removed) were initially screened by one reviewer (JEP) and those which did not meet the inclusion criteria were disregarded. After that, a second reviewer (PK, GY, OO) examined the remaining records independently based on titles and abstracts. When the decisions of two reviewers differed, the discrepancy was resolved based on full-texts and/or by discussion with a third reviewer (YFC) or the broader review team. This study screening process started from records of the most recent years (i.e. in the past three years) and then proceeded to prior years. Due to the larger than expected volume of the literature, we eventually screened records between 2016 to 2021 and did not cover earlier records in order to synthesise and present the findings from latest evidence in a timely fashion to inform the wider project hosting this review [28, 29].

Inclusion and exclusion criteria

A study was included when it: (1) described factors related to slum residents' accessing or utilisation of health care or the provision of health services in slums; and (2) was conducted in relation to slums in LMICs. Only articles written in English were included. A study was excluded when it was a commentary, opinion, or narrative review; described slum residents' utilisation of health services or the provision of health services without exploring the associated

For peer review only - http://bmjopen.bmj.com/site/about/guidelines.xhtml

factors; investigated informal care at home; or included mixed slum and non-slum populations without separately reporting data for slum residents or investigating residency in slums as a factor for healthcare access.

During our updated search in December 2021, we found several studies reporting healthcare utilisation [28] and provision related to COVID-19 in slums [30]. These studies were not included in this scoping review, since the factors associated with healthcare utilisation and health service provision under the pandemic situation are dramatically different and warrant a separate synthesis.

We included both primary studies and systematic reviews that examine data collected empirically and that derive their findings based on the data. Both quantitative and qualitative studies (and by extension, mixed methods studies) were considered. Even though slums have existed in both high-income countries and LMICs, the context may be quite different between these countries. For example, while all slums are vulnerable to natural disasters such as tropical cyclones, the impact of these could be far more severe in slums of LMICs due to the different socioeconomic contexts [31]. In this review, we focused on settings in LMICs and excluded studies conducted in high-income countries.

Study coding and data extraction/charting

Eligible studies were coded and data-extracted/charted according to a pre-specified, preliminary framework shown in Figure 1 below. The preliminary framework was developed by the review authors based on existing conceptual models related to healthcare access and service delivery [32-35] and was modified during the scoping review process to accommodate new factors/themes identified from the literature. The refined conceptual framework is shown in Figure 2.

For peer review only - http://bmjopen.bmj.com/site/about/guidelines.xhtml

Based on the refined conceptual framework, each eligible study was coded as being associated with one or more of the three phenomena of interest, namely slum residents' healthcare accessing (which covered perception of needs/desire for care, healthcare seeking and healthcare reaching as defined by Levesque et al [34]), health care utilisation and provision of health services (which covered various arrangements related to service delivery) in slum settings. (Figure 2)

In addition, to facilitate the organisation of complex evidence in this review, diverse factors reported in the included studies were initially classified into six different categories according to the preliminary framework shown in Figure 1. However, during the data charting process, we realised that many factors such as perception of symptoms and experience from past use of healthcare services did not fit into one of these six original categories. A new category of 'cognitive and experiential factors' was therefore added to the refined conceptual framework (Figure 2) to reflect the emerging themes, which include seven categories:

(1) Personal and biological factors: these relate to personal characteristics of slum residents, including age, sex, ethnicity and the nature and severity of health conditions.

(2) Cognitive and experiential factors: these relate to personal awareness, knowledge, perception, attitude, belief and experience etc. formed through cognitive process based on upbringing and past events.

(3) Socioeconomic factors: these include income and wealth, economic hardship/poverty and economic opportunities, marital status, education, crime, social capital (such as bonding, trust and reciprocity between close relatives, neighbours and community members) [36], use of technologies for social and economic purposes, commercial and charitable organisations and activities.

(4) Physical environment: this covers natural environment such as proximity to a health

For peer review only

For peer review only - http://bmjopen.bmj.com/site/about/guidelines.xhtml

facility, built environment and infrastructure such as water supply, transport and mobile/internet networks, as well as weather conditions and environmental pollutions.

(5) Cultural and religious factors: these include cultural and religious beliefs and activities, and local and national customs.

(6) Legal, political and policy factors: these include government policies and issues related to legal, justice and political systems.

(7) Health system factors: these relate to historical and current organisation and provision of health care that may impact upon provision and delivery of health services in individual slum communities and the services experienced by slum residents.

In addition to the addition of the 'cognitive and experiential factors' category, another major difference between the preliminary (Figure 1) and refined (Figure 2) conceptual framework relates to the definition of healthcare access. Our preliminary framework adopted the definition by Levesque and colleagues, who defined healthcare access as "the possibility to identify healthcare needs, to seek healthcare services, to reach the healthcare resources, to obtain or use health care services, and to actually be offered services appropriate to the needs for care." [34] However, during our study screening and data charting process, we found that it would be helpful to make a distinction between the process of 'accessing' healthcare (which covers gaining awareness of needs, forming an intention to seek healthcare and taking an action to reach healthcare) and the actual receipt and utilisation of health care ('accessed care') when examining empirical evidence, as healthcare needs could only be met when the latter occurs and this not only depends on factors related to service users (demand side) but also relies on factors related to service providers/planners (supply side). Therefore we separated out utilisation of health care from 'accessing health care' to highlight that it requires a match between demand and supply side factors.

For peer review only

Data on study population, study design, country in which the study was conducted, methodology, and associated factors were extracted using a data-charting spreadsheet which was developed and continuously updated as the review progressed by two of the reviewers (JEP and YFC). Whether a study was conducted exclusively within slums and whether a comparison was made between slum and non-slum urban or rural residents were also noted. Coding of phenomena and factors and data-charting were conducted by one reviewer (JEP) and checked by a second reviewer (PK, GY, OO, YFC). Disagreements were discussed between reviewers until consensus was reached.

Patient and public involvement

Given the focus of this scoping review on published literature, we did not directly involve residents and service providers/planners from slum settings. Nevertheless, our wider project has a work package that specifically engages with slum residents and service providers and planners [28], and early plans and findings of this review were shared with the wider project team who provided comments based on their experiences of community engagement.

RESULTS

The reporting of this review follows the PRISMA Extension for Scoping Reviews (PRISMA-ScR) [37]. Using the search strategy described earlier, a total of 15,091 records were retrieved from the initial and updated searches (Medline 4668, Embase 5090, Web of Science 3553, Cochrane 381, CINAHL 1575), with 9,916 records remaining after excluding duplicates. Two additional articles [18, 38] were identified from references of the included studies. As described earlier, screening was limited to the 4,368 records published from 2016 onwards.

For peer review only - http://bmjopen.bmj.com/site/about/guidelines.xhtml

A total of 111 articles were included in this scoping review. (Figure 3) Thirty-two studies reported factors associated with healthcare accessing of slum residents, 73 studies reported factors related to healthcare service utilisation, and 10 articles reported the factors related to provision of healthcare services in slums (four studies reported factors related to more than one phenomenon of interest). Seventy-four of the 111 studies were quantitative studies, 21 studies were qualitative studies, and 14 studies were undertaken using mixed-methods. The remaining two studies were systematic reviews. A total of 42 (38%) studies were conducted in India, followed by Kenya (14 studies, 13%). (Table 1)

For peer review only - http://bmjopen.bmj.com/site/about/guidelines.xhtml

Table 1. Characteristics of included studies.

Category	Subcategory		Number of studies (%)
Publication year	2016		22 (20)
	2017		17 (15)
	2018		23 (21)
	2019		22 (20)
	2020		18 (16)
	2021		9 (8)
Analysis method	Quantitative		74 (67)
	Qualitative		21 (19)
	Mixed-methods		14 (13)
	Narrative synthesis		2 (2)
Study location	Asia	India	42 (38)
		Bangladesh	9 (8)
		Nepal	4 (4)
		Pakistan	3 (3)
		Myanmar	2 (2)
		Iran	2 (2)
		Sri Lanka	1 (1)
	South America	Brazil	7 (6)
		Peru	2 (2)
	Africa	Kenya	14 (13)
		Ethiopia	7 (6)
		Malawi	4 (4)
		Uganda	3 (3)
		South Africa	2 (2)
		Sierra Leone	1 (1)
		Nigeria	1 (1)
		Egypt	1 (1)
		Zambia	1 (1)
		Namibia	1 (1)
		Ghana	1 (1)
	North America	Haiti	1 (1)
	Multiple nations		1 (1)
Healthcare services in slums*	Healthcare accessing		32
	Healthcare service utilisation		73
	Provision of healthcare services		10
Total			111 (100)

* One study reported factors related to both healthcare accessing and healthcare utilisation and three studies reported factors related to both healthcare utilisation and provision of healthcare services

For peer review only - http://bmjopen.bmj.com/site/about/guidelines.xhtml

Participants, country, study design, methodology, observed phenomena and outcomes, and factors of interests for each study are described in supplemental tables 1-3. Supplemental table 1 shows 32 studies reporting factors associated with general healthcare seeking behaviours; healthcare seeking for children or women; slum residents' preference for healthcare providers; and healthcare seeking related to HIV testing. Supplemental table 2 presents various factors reported in 73 studies related to general healthcare utilisation as well as use of specific services such as childhood immunisation, maternal healthcare, and possession of health insurance. In Supplemental table 3, ten studies reporting factors related to the provision of health services in slums are summarised. Key findings are described below.

Demand side: Factors associated with healthcare accessing and healthcare utilisation of slum residents

We found 104 articles which identified many different factors affecting healthcare accessing and utilisation. These factors are often inter-related and exert their influence at different levels (e.g. from personal, family to community level) in different circumstances. We classified various factors into seven categories (Figure 2). Factors particularly relevant to slum settings and other commonly identified factors within each category are highlighted below.

Personal and biological factors: The common factors associated with healthcare accessing and utilisation included intrinsic factors such as age [21, 39-56], sex [18, 21, 41, 46, 52, 54, 56-59], and ethnicity [21], familial factors such as birth order of the sick child [21, 47, 60-62], as well as personal health and type of illness [46, 59], disability [48] and morbidity [21, 52, 63, 64] and the specific features of the health condition [53, 56, 65]. Slum residents are more like to seek healthcare services when sick children are younger [49, 50, 53, 56], but evidence on

For peer review only - http://bmjopen.bmj.com/site/about/guidelines.xhtml

For peer review only

the association between mother's age and child's vaccination was inconsistent [21, 39]. Healthcare seeking and utilisation were different by sex, but the association was context dependent. Several studies reported higher healthcare utilisation among female slum dwellers [18, 52, 56, 58], while other studies showed male children had higher vaccination coverage [57] and incurred more medical expenditure [59]. Major life events such as recent migration [21, 50, 66-68] and relocation [49] into slums tend to be associated with lower healthcare seeking and utilisation. Recency of migration to slums was also related to lower uptake of Rashtriya Swasthya Bima Yojana (RSBY), a national health insurance programme run by the Indian government for poor families [69]. People with specific symptoms (such as fever, tachypnea, persistent vomiting) [53, 56, 65], disability [48] and illnesses including chronic disease [21, 52, 54, 63, 64] tend to use healthcare services more. Although people with tobacco habit were less likely to participate in breast cancer screening, they were more likely to take part when they had family history of cancer or history of cancer screening [70]. Lower birth order of the child was associated with increased utilisation of hospitals for childbirth [21, 47, 60-62], while the use of family planning service [47] and out-of-pocket expenditure was higher in multigravida than primigravida [63].

Cognitive and experiential factors: these factors were not included in our initial conceptual framework but rather emerged inductively from our data. Consequently, their identification led us to revise the conceptual framework for this scoping review. A wide range of factors formed through cognitive processes and influenced by individual's upbringing, past experience and surrounding environment were reported to be associated with both healthcare-seeking and healthcare utilisation of slum residents. Perception [39, 51, 53, 68, 71-77], knowledge [53, 78-83] and experience of symptoms and illnesses [51] were commonly found to influence

For peer review only - http://bmjopen.bmj.com/site/about/guidelines.xhtml

healthcare seeking and utilisation. Mothers who experienced child death and subsequently planned pregnancy showed higher use of antenatal healthcare services [51]. When people perceived the symptom or disease to be serious they tend to seek healthcare services [53, 71, 73, 77]. Although lack of knowledge could be a barrier to accessing healthcare services [78, 79], one study showed caregivers with good knowledge of child danger signs were less likely to seek healthcare services timely [53]. People perceiving their health status as good showed lower odds of having insurance [84], but awareness and knowledge of health problems lead people to use healthcare services [39, 74, 76, 80, 81, 83]. Home remedy and home management delayed healthcare seeking behavior [53, 71, 82, 85]. In addition, perception [21, 42, 64, 76, 84, 86-90], knowledge [21, 42, 49, 61, 68, 75, 82, 86, 91-96], and experience of healthcare services [39, 50, 60, 62, 67, 70, 75, 80, 87, 97, 98] including fear and distrust of healthcare services [21, 38, 68, 72, 75, 76, 79, 89, 99-101], and preference related to care provider's gender [88, 102] were frequently cited factors. Provider shopping associated with distrust of healthcare providers and denial of diagnosis delayed first care seeking and treatment initiation of pulmonary tuberculosis patients in India [71]. Perception or experience of healthcare services also affected uptake or renewal of health insurance [84, 95].

Socioeconomic factors: Socioeconomic status was associated with utilisation of healthcare services [21, 39, 40, 47, 57, 62, 82, 103], and even though one study showed that slum residents of lower socioeconomic class were more likely to enrol in health insurance than slum residents of higher socioeconomic class [40], the latter were more likely to use healthcare services [39, 47, 57, 62]. One study reported higher public hospital visits (compared with private hospital visits) among lower socioeconomic status [104]. Income and wealth [21, 41, 49-51, 61, 66, 68, 81, 105-109] including financial constraint [19, 21, 38, 46, 74, 77, 79, 85, 87, 90, 101, 103,

For peer review only - http://bmjopen.bmj.com/site/about/guidelines.xhtml

110-114] featured prominently. Higher education level [39, 49, 56-59, 61, 62, 66-68, 70, 81-83, 107-109, 115-117] and higher income [21, 49-51, 56, 58, 61, 66, 68, 81, 105, 107-109, 118] were associated with more seeking and utilisation of healthcare services. With some exceptions [67, 105], previous studies reported that employed slum residents tend to seek and use healthcare services more frequently than unemployed slum residents and housewives [49, 51, 66, 67, 70, 81, 83, 84, 119]. Even though married people tend to seek and use more healthcare services [18, 70], the reported influence of family type was inconsistent [39, 51, 116]. Female slum residents in nuclear family used more antenatal services than those in joint family type [51], but female in joint family type used more postnatal service [39] and immunisation service for their children [116]. Smaller family size used more maternal healthcare services [67], and bigger households had higher odds of having health insurance [69]. The socioeconomic challenges faced by slum residents also manifested as competing priorities [74, 94, 120] and lack of time [21, 101, 121] for healthcare-seeking and utilisation, because they did not want to or could not afford to miss work and lose income [21, 71], which can be exacerbated by lack of social support [75, 77, 88, 94, 100, 122].

Physical environment: Slum residents considered proximity of healthcare facilities [21, 38, 47, 77, 81, 85, 86, 96, 109, 123-127], transport such as travel assistance [77], lack of transportation [38, 83, 103], traffic congestion [128], and environment of residence area when they sought and used healthcare services. Long distance from health facility [38, 55, 77, 85, 102, 123], no transportation or travel assistance [38, 83, 103], unsafe environment of residential area such as darkness at night were reported as barriers to reaching healthcare facilities [100].

Cultural and religious factors: these included religion [41, 57, 60, 70, 76, 115, 129];

For peer review only - http://bmjopen.bmj.com/site/about/guidelines.xhtml

sociocultural influence [94, 102] such as exposure to media [80, 98]; stigma associated with unplanned/extramarital pregnancy [79, 94], postpartum depression [130] and other illnesses such as contagious skin disease, barrenness and female sexually-related problems [102]; and use of traditional/home medicine [76, 87, 100]. Women in slums could not go to hospital because they had difficulties in disclosing the symptoms, postponed their health issues because of their responsibilities at home, and engaged in self-treatment practices such as home remedies recommended by grandmother and friends because of socio-cultural influences toward healthcare-seeking behaviour [78]. Women in Ethiopia reported not returning to postnatal care due to religious and cultural expectation for mother and baby to stay home for 80 days after birth [94]. One Indian survey showed that some women could not seek healthcare services during labour since their husband or family did not allow that [38].

Legal, political and policy factors: type of slums (in terms of official recognition and availability of basic facilities) and possession of a ration card were found to be associated with uptake of the Indian RSBY national health insurance programme [69]. One study reported that slum residents could not seek healthcare facilities for abortion because of the perceived illegality of abortion [79].

Health system factors: slum residents were also influenced by many factors related to health systems when they sought healthcare. These included accessibility associated with the location [21, 102] and timing of services [21, 85, 87]; quality of healthcare services [38, 67, 86, 87, 102, 113, 125] such as delay in advising patients to go for related tests or referral [71], likelihood of receiving appropriate examination [85, 110], and adverse events [76]. Slum residents considered service organisation including medical turnover [131], availability of supplies/healthcare workers [47, 85, 110, 113], attitude of healthcare providers [87], type of healthcare facilities [38, 86, 117, 132], and waiting time [73, 85-87, 110, 112, 133]. Slum

For peer review only - http://bmjopen.bmj.com/site/about/guidelines.xhtml

residents tend to seek government and non-governmental organisation (NGO) facility [118] and avoid private hospitals [38] for healthcare services. Healthcare utilisation was higher among slum residents with healthcare insurance than those without it [18, 83], and households with higher quarterly out-of-pocket healthcare expenditure had lower scores for an index of access to primary health care [134].

In an Ethiopian study, some participants reported unavailability of female birth attendants as a reason for not delivering at healthcare facilities [113]. (Table 2)

For peer review only

For peer review only - http://bmjopen.bmj.com/site/about/guidelines.xhtml

Table 2. Factors associated with healthcare accessing and healthcare utilisation in slums from service user's (demand side) perspective.

Factors	Healthcare accessing	Healthcare utilisation
Personal and biological factors		
Age	(-) Age [53-56]	(±) Age [21, 39-52]; (+) age of household head [18]
Gender	(±) Sex [54, 56, 58, 59]	(±) Sex [18, 21, 41, 46, 52, 57, 59]; (male) sex of household head [134]
Ethnicity		Ethnicity [21]
Migration		(-) Recent migration [21, 50, 66-69]; (-) relocation [49]; (-) return to home village [21]
Biological	(+) Symptoms such as fever, tachypnea, chest in drawing, persistent vomiting [53, 56, 65]; having disease [54]	Type of illness [46, 59]; (+) having a disability [48]; (+) morbidity [21, 52, 63, 64]
Other personal	(-) Tobacco habits [70]; (+) family history of cancer and history of cancer screening [70]	(-) Birth order of sick child [21, 47, 60-62]; (-) parity [42, 47, 63, 135]
Cognitive and experiential factors		
Knowledge/experience of symptoms and illnesses	(+) Perception of symptoms [71] or illness [53, 73, 77]; (±) knowledge of symptom/disease [53, 78, 79]; (-) denial and complacency [72]	(+) Experience of child death [51]; (+) planned pregnancy [51]; (+) perceived health status [84] and health problem [39, 74, 76]; (+) knowledge of symptom [83]; disease [80, 81]
Ability/experience in handling health related conditions and perceived needs for accessing health services	(+) Awareness of the need for healthcare services [38, 54, 56]; (-) home remedies [71] or management of childhood illness [53, 85]	(+) Perceived needs for healthcare services [21, 68, 75, 76, 91, 94, 113, 129]; (-) home delivery [82]
Perception/knowledge/experience/preference of health services	(-) Fear of mistreatment [72, 101] and (-) doubts about medical care [38, 79, 100]; gender-induced affordability [102]; (-) provider shopping [71]	(positive) Perception of healthcare services [21, 42, 64, 76, 84, 86-90] and providers [21, 85, 94, 112, 125]; (+) knowledge of health services [21, 42, 49, 61, 68, 75, 82, 86, 91-94] or facilities [21, 95, 96]; (+) previous use of related healthcare services [39, 42, 50, 60, 62, 67, 80, 97, 98]; (-) bad experiences of friends and relatives at healthcare facilities [95]; (-) misunderstanding or fear [21, 68, 75, 76, 89, 99, 129]; gender healthcare worker preference [88], (-) side effect [82]; lack of trust [46]
Socioeconomic factors		
Socioeconomic status	(-) Social class [104]; social group (caste) of caregiver [54]	(+) Socioeconomic status [21, 39, 40, 47, 57, 62, 82, 103]; Caste [109, 115]; (rent-> negative) residential background [21, 47, 69, 82]; (+) possession of ration card [69]
Marital status	(married) Marital status [70]	(married) Marital status [18, 41]; duration of marriage [42]
Family composition & Living arrangement	(-) Family size [54]	(±) Family type [39, 51, 116, 127]; (±) family size [67, 69]; (-) number of children in household [21, 44, 49]; (+) number of male children [136]; (+)

For peer review only - http://bmjopen.bmj.com/site/about/guidelines.xhtml

		housing condition [21]
Education	(+) Education [54, 56, 58, 70]	(+) Education [39, 41, 42, 45, 49, 57, 59, 61, 62, 66-68, 81-83, 107-109, 115-117, 127, 135]; (±) husband education [44, 51]; (+) mother's education and literacy [21, 43, 47, 57, 60]
Income and wealth	(+) Income [56, 118]; (+) wealth [55, 58]; (-) inability to afford care [19, 38, 77, 79, 85, 90, 101, 110, 111]	(+) Income [41, 50, 51, 68, 81, 105]; (+) wealth [21, 49, 61, 66, 106-109]; (-) financial constraint [21, 46, 74, 87, 103, 112-114]
Occupation	(+) Occupation [54, 58, 70]	(+) Employment [21, 66, 84, 119, 127]; (±) occupation [49, 67, 81, 83, 105, 135]; (±) occupation of spouse [51, 61] or household [69]
Social support	(-) Difficulty in reaching services (security risk at night) [100]; (+) accompanying person [77]; decision making person for seeking health care [54]	(+) Family support [75, 88]; (+) social connectedness [94]; (+) socioeconomic support [122]; permission for immunisation by decision-maker [129]
Competing priorities/lack of time	(-) Competing priorities (ability to work and income) [120]; (-) not want to miss work [71]; (-) lack of time [101, 121]	(-) Competing priorities [74, 82, 94]; (-) risk of lost income [21]; (-) parents being too busy [21]
Physical environment		
Distance from health facility	Proximity of healthcare facilities [38, 55, 77, 85, 123]; geographical distance of formal healthcare [102]	(-) Distance from health facility [21, 47, 81, 86, 96, 109, 124-127]
Transport	(+) Travel assistance [77]; (-) no transportation [38]	(-) Lack of transportation [83, 96, 103]; (-) variability in traffic congestion [128]
Environment of residence area	(-) Difficulty in reaching services (darkness at night) [100]	Residential background [21, 69, 106]
Cultural and religious factors		
Religion	Religion [70]	Religion [41, 57, 60, 76, 115, 129]
Sociocultural influence	(-) Stigma [79, 102, 130]; mother tongue [70]; (-) difficulties in disclosing the symptoms, (-) neglecting behaviours, and socio-cultural influences [78]; (+) cultural competency of care [102]; (+) easy communication [102]; living with the burden of cultural expectations [102]; (-) no permission to seek care from family [38]	(-) Exposure to media [80, 98]; stigma [129]; (-) cultural expectation for women after birth and fear of stigma for pregnancy out of wedlock [94]
Tradition	**(-)** Traditional medicine [100]	**(-)** Traditional remedies [76]; **(-)** home remedies [87]
Legal, political and policy factors		
Legal issues	**(-)** Perceived illegality of abortion [79]	Type of slums and possession of a ration card [80]
Health system factors		
Accessibility	(+) Ease of access [102]; (-) late facility opening times [85]	(-) Limited access to the services due to location [91, 94]; (-) timing of services [21, 87]; household visit by health workers [21]
Quality and safety of services	Quality of treatment and expected outcome of therapies [38, 102]; (-) delay in advising related tests [71];	Quality of service [67, 86, 87, 92, 113, 125]; (-) adverse events [76]

For peer review only - http://bmjopen.bmj.com/site/about/guidelines.xhtml

	referral [71]; optimal examination [85, 110]; (-) provider shopping [71]	
Charges for health services	(+) Insurance coverage of both public and private providers and of extended family members [137]	(-) Average out-of-pocket healthcare expenditure [134]; healthcare insurance [18, 64, 83]
Service organisation and delivery arrangement	(-) Medical turnover and overload or healthcare providers [131]; (+) government/NGO facility [118]; (-) private hospital [38]; early engagement by healthcare workers [55]	Attitude of healthcare providers [87, 96]; mode of delivery [39, 57, 60, 63, 82, 138]; (-) hospitals refused to accept health insurance cards [95]
Facility & resources	Availability of medicines and supplies [85, 110]; (-) lack of healthcare facilities [139]	Type of healthcare facility [40, 86, 96, 117, 132, 134]; inadequate resources [91]; (+)number of available healthcare workers [47]; (-) unavailability of female birth attendants [113]
Waiting time	**(-)** Waiting time [73, 85, 110]	**(-)** Waiting time [86, 87, 112, 133]

(-) negative association; **(±)** inconsistent/conflicting evidence or context-dependent; **(+)** positive association; NGO: non-governmental organisation

For peer review only

For peer review only - http://bmjopen.bmj.com/site/about/guidelines.xhtml

Supply side: Provision of healthcare services

Ten articles described factors associated with provision of healthcare services in slums from the service providers' perspective. None of the studies reported personal and biological factors. Factors related to other categories are summarised below.

Cognitive and experiential factors: Odhiambo et al. reported slum residents' fear of side effects, size of tablet and misconceptions regarding treatment as the factors hindering drug administration activities by healthcare workers for a deworming programme in Kenya [140]. On the other hand, this study also reported a high demand for drugs from slum residents in the final year of this program because people realised that free treatment was to be ended [140].

Socioeconomic factors: effective community mobilisation was a facilitator [140] whereas poor community support [141] and insufficient time allocated for providers to implement healthcare programmes [140] were barriers for provision of healthcare services in slums. In the deworming programme mentioned above, community health workers reported that direct observation of slum residents taking deworming drugs after meals was sometimes not feasible because slum residents skipped or age late at night due to food shortage [140]. Some slum residents demanded money to take the deworming drugs, either to facilitate purchase of food or to have their own share of the money that they perceived the community health workers would be paid by the programme if they complied with taking the drugs [140].

Physical environment: poor sanitation [140, 142], presence of rodents and no pavement [142], bushy and unprotected environment [140] were reported as factors making the provision of healthcare services difficult in slums.

Cultural and religious factors: religious beliefs and mistrust of interventions [140], lack of a shared understanding of the needs, purposes and consequences of family planning and pregnancy related services among slum residents and healthcare providers [74] were the

For peer review only - http://bmjopen.bmj.com/site/about/guidelines.xhtml

barriers for healthcare services provision. In the previous deworming programme, portrayal of unrelated death being linked to the programme and related negative publicity affected participants' compliance [140].

Legal and political factors and policy: devolution of service delivery through downward transfer of funds and responsibilities from central/national government level to elected local bodies; management by professional managerial and technical cadres; tight organisation of public health services; and professional support from the state directorate of public health were found to strengthen public health service provision in Chennai slums compared with Delhi [143]. One study reported that policies affected healthcare provision negatively because of staff shortage arising from change and suspension of the appointment of health promotors, which led to overwork and lack of time to provide required care by healthcare staff [133]. In Brazil, home visits for the provision of healthcare services was hampered because slum residents could not present documents required to register for healthcare [142]. On the other hand, giving priority to socially less developed areas for strengthening the Family Health System in Brazil might have been associated with better service coverage for slum residents with tuberculosis compared with their urban non-slum counterparts [144].

Health system: pay scale of frontline healthcare workers [141], knowledge of intervention area by community health workers [140], issues related to rigid task assignment by service managers [142], requirement to follow standardised protocol [142], demands from the management [142], work burden [133, 142] and no incentive [141], insufficient time [140], attitude [74] and support of healthcare providers [141], ill-defined geographic boundary of service with unserved areas and left-out urban slum pockets [145] were associated with healthcare service provision in slums.

For peer review only - http://bmjopen.bmj.com/site/about/guidelines.xhtml

Lack of community-based care (such as school-based education for reproductive health and community support networks for women) [133], unreliable immunisation and household data [129]; inefficient utilisation of funds [129], affordability (price) and availability of medicine [146], limited medical supplies [74, 141] and infrastructural facilities [141], inadequate space and equipment [142, 145], suboptimal training of staff [145], insufficient availability of logistics, and health manpower [145] also affected service provision. (Table 3)

For peer review only

For peer review only - http://bmjopen.bmj.com/site/about/guidelines.xhtml

Table 3. Factors associated with provision of healthcare services in slums from service provider's (supply side) perspective.

Cognitive and experiential factors	
Perception/knowledge/experience/preference of health services	Fear of side effects, size of tablet and misconceptions regarding treatment, high demand for drugs in the final year of treatment [140]
Socioeconomic factors	
Income and wealth	Difficulty in directly observing deworming treatment at meal time due to food shortage [140]
Social support	Effective community mobilisation [140]; poor community support [141]; ; non-involvement of community members and Urban Local Bodies [145]; absence of community members during the drug administration exercise [140]; demand for incentives by community members to take deworming drugs [140]
Physical environment	
Environment of residence area	Environment (sanitation, territory) [142]; unsanitary environmental conditions [140]; inaccessibility (filthy and bush environment) [140]
Cultural and religious factors	
Religion	Religious beliefs and mistrust of interventions [140]
Sociocultural influence	Lack of shared understanding of the problems in community [74]; unrelated death and the associated negative publicity (of a deworming programme) by the media [140]
Legal, political and policy factors	
Policy issues	Devolution of service delivery transferring funds and responsibilities to elected local bodies [143]; management by professional managerial and technical cadres [143]; tight organisation of public health services [143]; professional support from the state directorate of public health [143]; healthcare policies [133]; policy prioritizing low social development areas [144]
Legal issues	Fear of requirement for formal registration [142]
Health system factors	
Cost	Pay scale of frontline healthcare workers [141]; medicine price [146]
Quality and safety of services	Knowledge of intervention area by community health workers [140]
Service organisation and delivery arrangement	Issues related to assignment of tasks [142]; requirement to follow standardised protocol [142]; demands from the management [142]; work overload [133, 142]; underperformance of staff [129]; documentation work/work burden/no incentive for work [141]; insufficient time [140]; attitude of healthcare providers [74]; lack of supportive staff [141]; community health worker familiarity with households led to warm reception [140]; opportunity to integrate mass drug administration with other health interventions [140]; presence of community health workers and their supervisory structure, and points of referral for serious side effects [140]; restriction of range of services [145]; unserved areas and left-out urban slum pockets [145]; poor monitoring and supervision [145]; unreliable immunisation and household data [129]
Facility & resources	Community-based care [133]; inefficient utilisation of funds [129]; affordability and availability of medicine [146]; limited medical supplies [74, 141]; infrastructural facilities [141]; inadequate space and equipment [142]; suboptimal training of staff [145]; insufficient availability of space, logistics, and health manpower [145]

For peer review only - http://bmjopen.bmj.com/site/about/guidelines.xhtml

Comparison between slums and other settings

Seven studies which met our inclusion criteria also included data from non-slum urban and/or rural areas and potentially allowed exploration of factors associated with healthcare access across different settings. Key findings from these studies are summarised in Table 4.

These recent studies showed a mixed and dynamic picture of healthcare access across slum and other settings and reported various factors associated with this. For example, the proportion of young children fully immunised was found to be lower in slums compared with non-slum urban setting but was higher than rural settings in Nigeria. Nevertheless the coverage improved over time across all settings [60]. While many common factors associated with full immunisation of young children were identified, giving birth in health facilities (as opposed to home) had a larger positive effect on subsequent immunisation coverage in slums compared with non-slum urban and rural settings [60]. A narrowing of gaps in delivery by skilled birth attendants between slum and non-slum urban settings over time and a reverse of the trend from having lower usage to higher usage of modern contraceptive methods by married women in slums versus urban non-slums were reported in Bangladesh [47]. Slum residents reported financial issues being the main reason for not taking prescribed drugs whereas getting better was the cited main reason for urban non-slum residents in Iran [114]. Better coverage of services and higher rates of treatment completion were reported for patients with tuberculosis in slums compared with non-slum urban setting in two studies in Brazil [52, 144], where a higher priority given to enhancing the Family Health system in socially less developed areas in recent years was suggested to be a likely factor associated with better service provision in slums [144].

(Table 4)

For peer review only - http://bmjopen.bmj.com/site/about/guidelines.xhtml

Table 4. Studies that examined factors associated with health care seeking and utilisation in both urban slum and non-slum urban and rural settings

Study & location	Differences in healthcare access	Associated factors
Kanyango (2021) [137]	*Preferences and willingness to pay for health insurance*<br>Households in non-slum communities had a high preference for health insurance plans covering chronic illnesses and major surgeries to other plans.	Coverage of extended family (vs restricted enrollment of children); coverage of both private and public providers (vs private only)
Obanewa (2020) [60]	*Fully-immunised child coverage (FIC)*<br>Proportion in slum lower than urban non-slum but higher than rural; proportions increased between 2003 and 2013 across all three settings	From multivariable regression*: year, birth order, antenatal attendance, maternal education level, religion, maternal age at child's birth, media exposure, region of the country, interaction between place of residence and place of delivery
Angeles (2019) [47]	*Use of modern contraceptive methods*<br>Proportion changed from being lower in slums in 2006 to being higher in slums in 2013 compared with urban non-slums	From multivariable regression*: parity, mother's age, mother's education attainment, socioeconomic status, interaction (slum × time period)
	Delivery by skilled birth attendant<br>Proportion substantially lower in slums compared with urban non-slums but the gaps narrowed over time)	From multivariable regression*: Residing in slums, parity, mother's age, mother's education attainment, length of stay in current city of residence, socioeconomic status, number of available community health worker, distance from health facility, interaction (slum x time period)
Islam (2018) [107]	***Antenatal care visits***<br>"there was a large inequality" between slum and urban non-slum (detail not reported)	Level of educational attainment, wealth index of the household
	Using contraceptive methods<br>"Prevalence rate higher among slum women" than urban non-slum women	Not reported

For peer review only - http://bmjopen.bmj.com/site/about/guidelines.xhtml

Tabrizi (2018) [114]	***Utilisation of health services in the past 30 days*** Similar utilisation overall, but with lower proportion received needed health services and used private clinics, higher use of vaccination and maternal health services, and lower use of services for heart failure and hypertension for slum residents compared with urban non-slum	High cost of services
	Home care services Very little use both in slum and urban non-slum areas	High cost of services
	Prescribed drug during last visit to health facilities Lower proportion for slum vs urban non-slum	Not reported
	Not taking drugs prescribed Higher proportion for slum vs urban non-slum	Main reason: financial problems for slum vs getting better/feeling well for non-slum urban
Snyder (2016) [52]	***Directly observed treatment coverage for tuberculosis (TB)*** Higher for slum vs urban non-slum TB patients	Not examined
	Abandonment of TB treatment Lower for slum vs urban non-slum TB patients	From multivariable regression*: residency in a slum, sex, age, extrapulmonary clinical disease, HIV/AIDS, interaction (directly observed treatment x residency in a slum)
Prado Junior (2016) [144]	***Coverage under Family Health system for TB patients*** Higher for slum vs urban non-slum	Giving the Family Health strategy priority to coverage of areas with lower social development

*From the model with most comprehensive adjustment including residency in slum as one of the variables; only factors that were statistically significant (at 5% level) are shown. AIDS: Acquired Immune Deficiency Syndrome; HIV: human immunodeficiency virus; TB: tuberculosis.

For peer review only

For peer review only - http://bmjopen.bmj.com/site/about/guidelines.xhtml

DISCUSSION

Statement of principle findings

This scoping review of recent literature examined demand side factors associated with slum residents' healthcare accessing and utilisation, as well as supply side factors associated with provision of health services in slums. We found over 104 studies related to the former, but only 10 studies related to the latter. We identified different factors associated with accessing, utilisation and provision of health services in slums, and mapped them to a conceptual framework developed and refined for this review into seven broad categories (Figure 2).

Findings in the context of existing literature

Even though previous reviews have investigated factors associated with healthcare access in various settings [147, 148], to our best knowledge this scoping review is the first that has examined wide-ranging factors across different service areas of health care in slums. Our findings are consistent with previous studies which highlighted common factors associated with healthcare seeking and utilisation such as age, income and education [147, 149]. We identified several factors that are particularly pertinent in slum settings, such as costs of healthcare [19, 21, 74, 77, 79, 85, 90, 101, 103, 110-112], lack of time due to slum residents' competing priorities [21, 101, 121] and issues arising from adverse physical environment [83, 103, 140, 142], security [100, 142], fear of formal registration due to distrust of the authorities [142] and proximity of healthcare facilities [21, 77, 81, 85, 86, 109, 123-126]. In addition, included studies showed that the effects of a given factor may differ between slum, urban non-slum and rural settings [60].

Healthcare cost is a major barrier between the intention to seek care and actual utilisation of services [110, 139]. Healthcare provision supported by tax-based financing and/or various

For peer review only - http://bmjopen.bmj.com/site/about/guidelines.xhtml

forms of social and private insurance that reduce out-of-pocket expenditure at point of care could be potential measures to overcome this barrier and help achieve universal coverage goals. Limited evidence showed that initiatives prioritising primary healthcare coverage in slums could improve access [144], but there is insufficient evidence from studies included in this review to determine the best model of healthcare financing for improving healthcare access and coverage in slum settings.

Although possession of/coverage by health insurance was associated with higher levels of utilisation of health services among slum residents [18, 83], studies showed that uptake of government-run public insurance among slum residents was low [69, 84]. This may be attributed to lack of awareness, difficulties in navigating through the health system and in obtaining official proof of identity required for enrolment [69], and poor quality of care and range of services offered [69, 84]. Even among slum residents covered by health insurance, access to care was often refused and additional charges were frequently requested [95]. Policies that aim to improve access to healthcare services among slum residents through public health insurance will need to address these challenges.

Several studies reported lack of time and competing priorities as a factor affecting healthcare-seeking behaviour [101, 120, 121] and health services utilisation [21, 74, 94]. This suggests a delicate balance between factors that individual slum residents have to strike when making decisions on healthcare seeking and utilisation. Var der Heijden et al. showed that health was considered as an asset for working ability in slums [120], but paradoxically the ability to work often seems to impede healthcare seeking for health issues. This highlights the importance of considering slum residents' interest and priorities when providing healthcare services and promoting healthcare utilisation in slums.

For peer review only - http://bmjopen.bmj.com/site/about/guidelines.xhtml

Strengths and weaknesses of the review

This scoping review has several strengths. We conducted a comprehensive literature search using generic terms related to slums with few other restrictions. The search was therefore likely to be sensitive for identifying relevant literature. Contemporary methodological guidelines for undertaking scoping reviews were followed [22], and a conceptual framework which was adapted based on emerging findings was used to facilitate the organisation of evidence.

The review has enabled theory building and refinement of a conceptual framework. Our preliminary framework included six categories (Figure 1). During data coding and extraction, it emerged that many studies reported perception, knowledge, and experience of slum residents being associated with their healthcare-seeking and utilisation. We subsequently classified these factors as cognitive and experiential factors, which primarily consists of three subcategories: knowledge/experience of illness, perceived needs for accessing healthcare services, and perception/experience of healthcare services. These factors were influenced by other factors included in our original conceptual framework, but highlighted the crucial links between those factors and the ultimate actions by individual slum residents to access health services. Future interventions to promote health service utilisation for slum residents [150] could make use of our framework to develop programme theories and map out causal pathways.

This review also has some limitations. Given time constraint, we were only able to examine the most recent literature published in English in academic journals, and have not examined the methodological quality of individual studies (which we noted to be quite varied) in detail. We attempted some preliminary synthesis to configure the identified evidence but have not explored the complex relationship between the factors identified and their interplay with the context of individual slums in depth. Nevertheless, findings from this scoping review will provide a good foundation for further syntheses.

For peer review only

For peer review only - http://bmjopen.bmj.com/site/about/guidelines.xhtml

Methodological considerations

A number of challenges in the process of classifying and coding data are worth mentioning. Firstly, access to healthcare has been conceptualised and defined in various ways in previous studies. The World Health Organization suggested six building blocks of a health system including service delivery, health workforce, health information systems, access to essential medicines, financing, leadership/governance to strengthen health systems [151], and in the report, defined access to healthcare as public responsibility for ensuring all citizens' entitlements to the protection of their health beyond simply proportion of a target population that benefits from an intervention or universal coverage [151]. They also pointed out system constraints such as financial access difficulty, physical access difficulty, low knowledge and skills, poorly motivated staff, weak leadership and management, ineffective intersectoral action and partnership as barriers to access [151]. The WHO's definition and conceptual framework focus on health system level factors and would be particularly useful when examining supply side factors, which seem to be under-studied based on our findings. As described in the Methods section, we primarily adopted the conceptual model of healthcare access developed by Levesque and colleagues given our shared focus on service users. However, in our conceptual model we separated the dynamic stages of 'accessing' healthcare from the actual 'accessed' healthcare utilisation to highlight the crucial match required between the demand side and supply side factors to facilitate access to healthcare.

Several factors associated with healthcare accessing and utilisation can be viewed from different perspectives and therefore potentially be coded under different categories. For example, barriers for healthcare seeking and utilisation related to costs can be considered as socioeconomic issues from the slum dwellers' perspective but can also be viewed as health

For peer review only - http://bmjopen.bmj.com/site/about/guidelines.xhtml

system issues for not offering the services in an affordable way. Indeed, previous access frameworks suggested that access is created and negotiated in a dynamic interchange between households/communities and healthcare workers/systems (i.e. demand and supply) on each access dimension [34, 152]. In such situations, we tried to code a factor under the category that most directly reflects the original data through discussions within the review team (in the example of healthcare cost, the factor was coded primarily under socioeconomic factors rather than health system factors when the factor was reported by slum residents as a barrier); otherwise more than one category was coded (for example, bad experience from previous utilisation of health services was coded both as a cognitive and experiential factor and a health system factor).

Implication for research and practice

The multitude of factors identified in this review are often inter-related and inter-acting, and span across personal, family, community and society levels. For example, the association between occupation and healthcare utilisation were reported in several studies [49, 61, 67, 81, 83, 105]. The effect of predominantly casual work undertaken by slum residents on their healthcare access could be mediated through working hours, income level, knowledge of health and available services, etc. There is also possibility that occupation was associated with health status and hence needs for healthcare services, instead of/in addition to behaviour of using healthcare services. Teasing out the complicated relationships between various determinants and their interaction with the diverse contexts of slums will require in-depth analysis and a more holistic approach to synthesising the evidence. Given the unique features of individual slums, service planners and policy makers will need to examine these relationships with due consideration to the context specific to each locality and geospatial features and neighbourhood

For peer review only - http://bmjopen.bmj.com/site/about/guidelines.xhtml

For peer review only

effects that characterise slum settings [4].

We found far fewer studies that have examined health service providers' perspective than studies that have investigated factors associated with accessing healthcare from slum residents' perspective. There may be scope for greater research and policy attention to supply-side factors, including experiences and practices of local frontline healthcare providers, availability of healthcare facilities and infrastructure and policy to support them in order to overcome the many barriers highlighted from both supply and demand sides.

Although only six of the included studies explored factors associated with healthcare access or health service provision across slum and non-slum settings, they showed a generally encouraging picture that access to and provision of healthcare are continuously evolving (and often improving) in slums and other settings, and equality between different settings is not beyond reach.

CONCLUSION

This scoping review summarises a large body of recent literature evaluating factors associated with seeking and utilisation of healthcare by slum residents, but found substantially fewer studies examining factors associated with provision of health services from providers' perspective. Recent migration into slums; knowledge, perception (including misconception and distrust) and past experience of illness, healthcare needs and health services; financial constraint, competing priorities and inadequacy of social support; adverse physical environment and unfavourable locality; sociocultural expectations and stigma; lack of official recognition; and various problems in existing health system all contribute towards the challenges faced by slum residents. Future research and policy aiming at improving healthcare

For peer review only - http://bmjopen.bmj.com/site/about/guidelines.xhtml

services in slums should pay more attention to supply side issues ranging from individual healthcare providers and practices to structural and policy level factors to tackle different barriers faced by slum residents, which in turn need to be evaluated holistically and take into account local context and geospatial features of slums.

For peer review only

For peer review only - http://bmjopen.bmj.com/site/about/guidelines.xhtml

List of abbreviations

GRADE: Grading of Recommendations Assessment, Development and Evaluation

LMICs: Low and Middle income Countries

MMAT: Mixed Methods Appraisal Tool

WHO: World Health Organization

Ethics approval

Not applicable. This realist synthesis included literature that is available in the public domain and did not involve the collection of personal data.

Consent for publication

The authors were required to notify the funder of the research, the UK National Institute for Health Research (NIHR) prior to the publication of this manuscript. The funder did not otherwise play any roles in the preparation of the manuscript and decision to submit it.

Availability of data and materials

All data relevant to the study were included in the article or uploaded as supplementary information. No additional data were available.

Competing interests

The authors declare that they have no competing interest.

Acknowledgement and Funding

This research is funded by the NIHR Global Health Research Unit on Improving Health in Slums using UK aid from the UK Government to support global health research (Award/Grant number is not applicable). The views expressed in this publication are those of the author(s) and not necessarily those of the NIHR or the UK Department of Health and Social Care. MA gratefully acknowledges support provided by the Warwick Institute of Advanced Study Global Challenges Research Fund Fellowship No. IAS/32013/1914. FG receives funding as South

For peer review only

For peer review only - http://bmjopen.bmj.com/site/about/guidelines.xhtml

Africa Research Chair in Health Policy and Systems from the National Research Foundation, South Africa (Award/Grant number is not applicable). RL is supported by the NIHR Applied Research Collaboration (ARC) West Midlands, UK (Award/Grant number is not applicable). YFC is supported by Warwick Evidence, which is a Technology Assessment Review team funded by the NIHR Evidence Synthesis Programme (Award/Grant number is not applicable). Upon submission, NA had joined the Population Health Sciences Institute, Newcastle University (UK).

Contributor

JEP, BH, MA, FG and YFC conceptualised the scoping review; JEP carried out literature searches; JEP, PK, GY, OO, and YFC participated in study screening and coding; JEP and YFC performed data charting and drafted in initial manuscript. NA, PG and RL provided critical input during the drafting of the manuscript. All authors commented on and contributed to the revision of subsequent versions and approved the final version for submission.

Collaborators

The Improving Health in Slums Collaborative:

African Population and Health Research Centre (APHRC), Nairobi, Kenya:

Pauline Bakibinga, Caroline Kabaria, Ziraba Kasiira, Peter Kibe, Lyagamula Kisia, Catherine Kyobutungi, Nelson Mbaya, Blessing Mberu, Shukri Mohammed, Anne Njeri.

Aga Khan University, Karachi, Pakistan:

Iqbal Azam, Romaina Iqbal, Ahsana Nazish, Narjis Rizvi.

Independent University, Bangladesh, Dhaka, Bangladesh:

Syed Shifat Ahmed, Nazratun Choudhury, Omar Rahman, Rita Yusuf.

Nigerian Academy of Sciences, Lagos, Nigeria:

Doyin Odubanjo.

For peer review only

For peer review only - http://bmjopen.bmj.com/site/about/guidelines.xhtml

University of Ibadan, Ibadan, Nigeria:

Motunrayo Ayobola, Olufunke Fayehun, Akinyinka Omigbodun, Mary Osuh, Eme Owoaje, Olalekan Taiwo.

University of Birmingham, Birmingham, UK:

Richard Lilford, Jo Sartori, Samuel Watson.

University of Lancaster, Lancaster, UK:

Peter Diggle.

University of Warwick, Coventry, UK:

Navneet Aujla, João Porto de Albuquerque, Yen-Fu Chen, Paramjit Gill, Frances Griffiths, Bronwyn Harris, Jason Madan, Oyinlola Oyebode, Ji-Eun Park, Simon Smith, Grant Tregonning, Olalekan Uthman, Ria Wilson, Godwin Yeboah.

REFERENCES

[1] United Nations. The Sustainable Development Goals Report, 2019.

[2] Kosamkar A. Problems in urban society. *EPRA Int J Multidiscip Res* 2020;6.

[3] Ezeh A, Oyebode O, Satterthwaite D, et al. The history, geography, and sociology of slums and the health problems of people who live in slums. *Lancet* 2017;389:547-58.

[4] Lilford R, Kyobutungi C, Ndugwa R, et al. Because space matters: conceptual framework to help distinguish slum from non-slum urban areas. *BMJ Glob Health* 2019;4:e001267.

[5] Zulu EM, Beguy D, Ezeh AC, et al. Overview of migration, poverty and health dynamics in Nairobi City's slum settlements. *J Urban Health* 2011;88 Suppl 2:S185-99.

[6] Michiani MV, Asano J. Physical upgrading plan for slum riverside settlement in traditional area: A case study in Kuin Utara, Banjarmasin, Indonesia. *Front Archit Res* 2019;8:378-95.

For peer review only - http://bmjopen.bmj.com/site/about/guidelines.xhtml

[7] Winter SC, Dreibelbis R, Dzombo MN, et al. A mixed-methods study of women's sanitation utilization in informal settlements in Kenya. *PLoS One* 2019;14:e0214114.

[8] Salinas DA, Fouts HN, Neitzel CL, et al. Young Children's Social Networks in an Informal Urban Settlement in Kenya: Examining Network Characteristics Among Kamba, Kikuyu, Luo, and Maasai Children. *J Cross-Cult Psychol* 2019;50:639-58.

[9] Prayitno G, Sari N, Putri IK. Social capitl in poversity alleviation through pro-poor tourism concept in slum area (case study: Kelurahan jodipan, Malang city). *Int J GEOMATE* 2019;16:131-37.

[10] Khalil D. The Flexible Governance of Water in Cairo's Informal Areas. *Water* 2019;11:1644.

[11] Portner CC, Su YH. Differences in Child Health Across Rural, Urban, and Slum Areas: Evidence From India. *Demography* 2018;55:223-47.

[12] Mberu BU, Haregu TN, Kyobutungi C, et al. Health and health-related indicators in slum, rural, and urban communities: a comparative analysis. *Glob Health Action* 2016;9:33163.

[13] Mlangeni L, Makola L, Naidoo I, et al. Factors associated with physical activity in south africa: Evidence from a national population based survey. *Open Public Health Journal* 2018;11:516-25.

[14] Rebecca L. Nunn, Sarah H. Kehoe, Harsha Chopra, et al. Dietary micronutrient intakes among women of reproductive age in Mumbai slums. *Euro J of Clinical Nutrition* 2019;73:1536–45.

[15] Samal J. Perception and knowledge of tuberculosis and its services among slum dwellers in Chhattisgarh. *Indian J Respir Care* 2017;6:828-31.

[16] Khan MZ, Shujaa MD, Iftikhar H. Utilization of ante-natal services among reproductive age women of Bahawalpur. *Indo Am J Pharm Sci* 2018;5:11355-65.

For peer review only - http://bmjopen.bmj.com/site/about/guidelines.xhtml

[17] Duy Kien V, Van Minh H, Bao Giang K, et al. Horizontal inequity in public health care service utilization for non-communicable diseases in urban Vietnam. *Glob Health Action* 2014;7:24919.

[18] Amiresmaili M, Yazdi-Feyzabadi V, Heidarijamebozorgi M. Health services utilization among slum dwellers: An experience from Iran. *J Educ Health Promot* 2019;8:210.

[19] McNairy ML, Tymejczyk O, Rivera V, et al. High Burden of Non-communicable Diseases among a Young Slum Population in Haiti. *J Urban Health* 2019;96:797-812.

[20] Sverdlik A. Ill-health and poverty: a literature review on health in informal settlements. *Environ Urban* 2011;23:123-55.

[21] Crocker-Buque T, Mindra G, Duncan R, et al. Immunization, urbanization and slums - a systematic review of factors and interventions. *BMC Public Health* 2017;17:556.

[22] Peters MDJ, Godfrey C, McInerney P, et al. Chapter 11: Scoping Reviews. In: Aromataris E, Munn Z (Editors). Joanna Briggs Institute Reviewer's Manual 2017

[23] Center for Open Science 2020. Slum health healthcare access and provision of services scoping review protocol 05082020.pdf (Version: 1). Available from: https://osf.io/mj6kp/ [Accessed 9 Jun 2021].

[24] United Nations. United Nations 2021. Available from: https://www.un.org/en [Accessed 25 Dec 2021].

[25] World Health Organization. World Health Organization 2021. Available from: https://www.who.int [Accessed 25 Dec 2021].

[26] SDI. SDI 2021 Available from: https://sdinet.org [Accessed 25 Dec 2021].

[27] UN HABITAT. UN-Habitat for a better urban future 2021. Available from: https://unhabitat.org/ [Accessed 25 Dec 2021].

[28] Ahmed S, Ajisola M, Azeem K, et al. Impact of the societal response to COVID-19 on

For peer review only - http://bmjopen.bmj.com/site/about/guidelines.xhtml

access to healthcare for non-COVID-19 health issues in slum communities of Bangladesh, Kenya, Nigeria and Pakistan: results of pre-COVID and COVID-19 lockdown stakeholder engagements. *BMJ Glob Health* 2020;5:e003042.

[29] Improving Health in Slums C. A protocol for a multi-site, spatially-referenced household survey in slum settings: methods for access, sampling frame construction, sampling, and field data collection. *BMC Med Res Methodol* 2019;19:109.

[30] George CE, Inbaraj LR, Rajukutty S, et al. Challenges, experience and coping of health professionals in delivering healthcare in an urban slum in India during the first 40 days of COVID-19 crisis: a mixed method study. *BMJ Open* 2020;10:e042171.

[31] Peduzzi P, Chatenoux B, Dao H, et al. Global trends in tropical cyclone risk. *Nature climate change* 2012;2:289-94.

[32] Effective Practice and Organisation of Care (EPOC). EPOC Taxonomy 2015. Available from: https://epoc.cochrane.org/epoc-taxonomy [Accessed 27 Jul 2020].

[33] Lavis JN, Wilson MG, Moat KA, et al. Developing and refining the methods for a 'one-stop shop' for research evidence about health systems. *Health Res Policy Syst* 2015;13:10.

[34] Levesque JF, Harris MF, Russell G. Patient-centred access to health care: conceptualising access at the interface of health systems and populations. *Int J Equity Health* 2013;12:18.

[35] National Cancer Institute. Theory at a Glance: A Guide for Health Promotion Practice, 2005.

[36] Mpanje D, Gibbons P, McDermott R. Social capital in vulnerable urban settings: an analytical framework. *J Int Humanit Action* 2018;3:4.

[37] Tricco AC, Lillie E, Zarin W, et al. PRISMA Extension for Scoping Reviews (PRISMA-ScR): Checklist and Explanation. *Ann Intern Med* 2018;169:467-73.

[38] Pune Municipal Corporation. City Health Plan 2016 - 2020, 2016. Available from:

For peer review only - http://bmjopen.bmj.com/site/about/guidelines.xhtml

https://www.healthynewbornnetwork.org/hnn-content/uploads/Pune-City-Health-Plan.pdf [Accessed 10 Jun 2021]

[39] Upadhyai N, Gupta SK. Utilization of Postnatal Care Services and Factors Affecting It among Women of Urban Slums in Dehradun, Uttarakhand. *Indian J Community Health* 2019;31:470-76.

[40] Mendhe HG, David R, Singh D, et al. Universal Health Insurance coverage and utilization among women in urban slum of Rajnandgaon, Chhattisgarh. *J Family Med Prim Care* 2021;10:1313-19.

[41] Iyalomhe FO, Adekola PO, Cirella GT. Community-based health financing: empirical evaluation of the socio-demographic factors determining its uptake in Awka, Anambra state, Nigeria. *Int J Equity Health* 2021;20:235.

[42] Yadav K, Shukla M, Agarwal M, et al. Unmet need for family planning services among young married women (15-24 years) living in urban slums of India. *BMC Womens Health* 2020;20:187.

[43] Veras A, Lima EJD, Caminha MDC, et al. Vaccine uptake and associated factors in an irregular urban settlement in northeastern Brazil: a cross-sectional study. *BMC Public Health* 2020;20:1152

[44] Roja VR, Narayanan P, Sekaran VC, et al. Living environment and health of under-five children in urban slums of a coastal region in South India. *Ghana Medical Journal* 2020;54:238-44.

[45] de Araujo Veras AAC, da Fonseca Lima EJ, Caminha MFC, et al. Vaccine uptake and associated factors in an irregular urban settlement in northeastern Brazil: a cross-sectional study. *BMC Public Health* 2020;20:1152]

[46] Vora K, Saiyed S, Shah AR, et al. Surgical Unmet Need in a Low-Income Area of a

For peer review only - http://bmjopen.bmj.com/site/about/guidelines.xhtml

Metropolitan City in India: A Cross-Sectional Study. *World J Surg* 2020;44:2511-7.

[47] Angeles G, Ahsan KZ, Streatfield PK, et al. Reducing Inequity in Urban Health: Have the Intra-urban Differentials in Reproductive Health Service Utilization and Child Nutritional Outcome Narrowed in Bangladesh? *J Urban Health* 2019;96:193-207.

[48] Renzaho AM, Kamara JK, Georgeou N, et al. Sexual, Reproductive Health Needs, and Rights of Young People in Slum Areas of Kampala, Uganda: A Cross Sectional Study. *PLoS One* 2017;12:e0169721.

[49] Horng L, Kakoly NS, Abedin J, et al. Effect of household relocation on child vaccination and health service utilisation in Dhaka, Bangladesh: a cross-sectional community survey. *BMJ Open* 2019;9: e026176.

[50] Lae WL, Jayasvasti I, Mongkolchati A, et al. Utilization of immunization service and predictors among under 3-year-old children in urban slums of Chanmyathazi Township, Mandalay, Myanmar. *J Med Assoc Thai* 2018;101:1085-92.

[51] Sharma D, Pokharel HP, Budhathoki SS, et al. Antenatal Health Care Service Utilization in Slum Areas of Pokhara Sub-Metropolitan City, Nepal. *J Nepal Health Res Counc* 2016;14:39-46.

[52] Snyder RE, Marlow MA, Phuphanich ME, et al. Risk factors for differential outcome following directly observed treatment (DOT) of slum and non-slum tuberculosis patients: a retrospective cohort study. *BMC Infectious Diseases* 2016;16:494.

[53] Lungu EA, Darker C, Biesma R. Determinants of healthcare seeking for childhood illnesses among caregivers of under-five children in urban slums in Malawi: a population-based cross-sectional study. *BMC Pediatr* 2020;20:20.

[54] Mohanty P, Patnaik L, Satpathy SK, et al. Do the caregivers of under-fives have proper health care seeking behaviour for their children? A study from urban slums of a city in

For peer review only

For peer review only - http://bmjopen.bmj.com/site/about/guidelines.xhtml

eastern India. *J Nepal Paediatr Soc* 2021;41:169-76.

[55] Pakhare A, Joshi A, Kumar S, et al. Linkage to primary-care public health facilities for cardiovascular disease prevention: A community-based cohort study from urban slums in India. *BMJ Open* 2021;11:e045997.

[56] Kerai S, Nisar I, Muhammad I, et al. A Community-Based Survey on Health-Care Utilization for Pneumonia in Children in Peri-Urban Slums of Karachi, Pakistan. *Am J Trop Med Hyg* 2019;101:1034-41.

[57] Devasenapathy N, Jerath SG, Sharma S, et al. Determinants of childhood immunisation coverage in urban poor settlements of Delhi, India: a cross-sectional study. *BMJ Open* 2016;6:e013015.

[58] Kar SS, Kalaiselvi S, Archana R, et al. Is rule of halves still an occurrence in South India: Findings from community-based survey in a selected urban area of Puducherry. *J Postgrad Med* 2017;63:232-36.

[59] Mishra S, Kusuma YS, Babu BV. Treatment-seeking and out-of-pocket expenditure on childhood illness in a migrant tribal community in Bhubaneswar, Odisha State, India. *Paediatr Int Child Health* 2017;37:181-87.

[60] Obanewa OA, Newell ML. The role of place of residency in childhood immunisation coverage in Nigeria: analysis of data from three DHS rounds 2003-2013. *BMC public health* 2020;20:123.

[61] Shrestha S, Shrestha DK. Utilization of Maternal Health Care Services among Mothers Residing at Slum Area. *J Nepal Health Res Counc* 2019;17:193-99.

[62] Neyaz A, Ahmed MS, Sahu PC. Preference and practices regarding place of childbirth in the slums of a city in Northern India. *J Pioneer Med Sci* 2016;6:33-36.

[63] Sahu KS, Bharati B. Out-of-Pocket health expenditure and sources of financing for

For peer review only - http://bmjopen.bmj.com/site/about/guidelines.xhtml

delivery, postpartum, and neonatal health in urban slums of Bhubaneswar, Odisha, India. *Indian J Public Health* 2017;61:67-73.

[64] Wambiya EOA, Otieno PO, Mutua MK, et al. Patterns and predictors of private and public health care utilization among residents of an informal settlement in Nairobi, Kenya: a cross-sectional study. *BMC Public Health* 2021;21:850.

[65] Mishra S, Kusuma YS, Babu BV. Mother's Recognition of and Treatment Triggers for Common Childhood Illnesses among Migrant Santal Tribe Living in Bhubaneswar, Odisha, India. *J Trop Pediatr* 2017;63:301-06.

[66] Razzaque A, Clair K, Chin B, et al. Association of Time since Migration from Rural to Urban Slums and Maternal and Child Outcomes: Dhaka (North and South) and Gazipur City Corporations. *J Urban Health* 2020;97:158-70.

[67] Geddam JB, Ponna SN, Kommu PR, et al. Utilization of maternal health services by the migrant population living in the non-notified slums of Hyderabad city, India. *Indian J Community Health* 2017;29:29-38.

[68] Singh S, Sahu D, Agrawal A, et al. Ensuring childhood vaccination among slums dwellers under the National Immunization Program in India - Challenges and opportunities. *Prev Med* 2018;112:54-60.

[69] Kusuma YS, Pal M, Babu BV. Health Insurance: Awareness, Utilization, and its Determinants among the Urban Poor in Delhi, India. *J Epidemiol Glob Health* 2018;8:69-76.

[70] Kulkarni S, Mishra G, Dussane R, et al. Determinants of compliance to breast cancer screening and referral among women from urban slums in India. *Eur J Cancer* 2016;54:S51.

[71] Mistry N, Rangan S, Dholakia Y, et al. Durations and Delays in Care Seeking, Diagnosis and Treatment Initiation in Uncomplicated Pulmonary Tuberculosis Patients in Mumbai,

For peer review only - http://bmjopen.bmj.com/site/about/guidelines.xhtml

India. *PLoS One* 2016;11:e0152287.

[72] Thomson KA, Telfer B, Opondo AP, et al. Navigating the risks of prevention of mother to child transmission (PMTCT) of HIV services in Kibera, Kenya: Barriers to engaging and remaining in care. *PLoS One* 2018;13: e0191463.

[73] Kamati M, Godman B, Kibuule D. Prevalence of Self-Medication for Acute Respiratory Infections in Young Children in Namibia: Findings and Implications. *J Res Pharm Pract* 2019;8:220-24.

[74] Kaba M, Taye G, Getachew S, et al. Perceived barriers to health care for residents in vulnerable urban centers of Ethiopia. *Ethiop J Health Dev* 2020;34:4-11.

[75] Verma H, Sagili KD, Zachariah R, et al. Do incentivised community workers in informal settlements influence maternal and infant health in urban India? *Public Health Action* 2017;7:61-66.

[76] Pugliese-Garcia M, Heyerdahl LW, Mwamba C, et al. Factors influencing vaccine acceptance and hesitancy in three informal settlements in Lusaka, Zambia. *Vaccine* 2018;36:5617-24.

[77] Ramagiri R, Kannuri NK, Lewis MG, et al. Evaluation of whether health education using video technology increases the uptake of screening for diabetic retinopathy among individuals with diabetes in a slum population in Hyderabad. *Indian J ophthalmol* 2020;68:S37-S41.

[78] Sumudrika Ilankoon I, Evangeline Goonewardena C, Fernandopulle R, et al. Women's Understanding and Cultural Practices Related to Vaginal Discharge: A Qualitative Study. *Nurs Midwifery Stud* 2018;7:74-80.

[79] Jayaweera RT, Ngui FM, Hall KS, et al. Women's experiences with unplanned pregnancy and abortion in Kenya: A qualitative study. *PLoS One* 2018;13:e0191412.

For peer review only - http://bmjopen.bmj.com/site/about/guidelines.xhtml

[80] Kusuma YS, Kaushal S, Garg R, et al. Birth preparedness and determinants of birth place among migrants living in slums and slum-like pockets in Delhi, India. *Sex Reprod Healthc* 2018;16:160-66.

[81] Adane M, Mengistie B, Mulat W, et al. Utilization of health facilities and predictors of health-seeking behavior for under-five children with acute diarrhea in slums of Addis Ababa, Ethiopia: a community-based cross-sectional study. *J Health Popul Nutr* 2017;36:9.

[82] Shrestha S, Shrestha M, Wagle RR, et al. Predictors of incompletion of immunization among children residing in the slums of Kathmandu valley, Nepal: a case-control study. *BMC Public Health* 2016;16:970.

[83] Owusu-Ansah FE, Tagbor H, Togbe MA. Access to health in city slum dwellers: The case of Sodom and Gomorrah in Accra, Ghana. *Afr J Prim Health Care Fam Med* 2016;8:e1-7.

[84] Otieno PO, Wambiya EOA, Mohamed SF, et al. Prevalence and factors associated with health insurance coverage in resource-poor urban settings in Nairobi, Kenya: a cross-sectional study. *BMJ Open* 2019;9:e031543.

[85] Lungu EA, Biesma R, Chirwa M, et al. Healthcare seeking practices and barriers to accessing under-five child health services in urban slums in Malawi: a qualitative study. *BMC Health Serv Res* 2016;16:410.

[86] Owiti A, Oyugi J, Essink D. Utilization of Kenya's free maternal health services among women living in Kibera slums: a cross-sectional study. *Pan Afr Med J* 2018;30:86.

[87] Madan NV. Ethnographic Perspectives on Slum-dwelling Women's Access to Primary Care: The Case of Pune, India. *Urbanities* 2019;9:114-30.

[88] Wairiuko JM, Cheboi SK, Ochieng GO, et al. Access to Healthcare Services in Informal Settlement: Perspective of the Elderly in Kibera Slum Nairobi-Kenya. *Ann Med Health Sci Res* 2017;7:5-9.

For peer review only

For peer review only - http://bmjopen.bmj.com/site/about/guidelines.xhtml

[89] Martinez Perez G, Cox V, Ellman T, et al. 'I Know that I Do Have HIV but Nobody Saw Me': Oral HIV Self-Testing in an Informal Settlement in South Africa. *PLoS ONE* 2016;11:e0152653.

[90] Sudhinaraset M, Beyeler N, Barge S, et al. Decision-making for delivery location and quality of care among slum-dwellers: a qualitative study in Uttar Pradesh, India. *BMC Pregnancy Childbirth* 2016;16:148.

[91] Sendo EG, Chauke ME, Ganga-Limando M. Why some women who attend focused antenatal care fail to deliver in health facilities: a qualitative study of women's perspectives from slums of Addis Ababa, Ethiopia. *BMJ Open* 2020;10:e039189.

[92] Sendo EG, Chauke ME, Ganga-Limando M. Women's perspectives on the measures that need to be taken to increase the use of health-care facility delivery service among slums women, Addis Ababa, Ethiopia: a qualitative study. *Reprod Health* 2021;18(1):174.

[93] Manandhar K, Bajcharya K, Prajapati R, et al. Prevalence and Predictors of Incomplete Immunization among Children Residing in the Slums of Kathmandu Valley: A Community Based Door-to-Door Survey. *Kathmandu Univ Med J* 2018;16:8-13.

[94] Kaba M, Taye G, Gizaw M, et al. Maternal health service utilization in urban slums of selected towns in Ethiopia: Qualitative study. *Ethiop J Health Dev* 2017;31:96-102.

[95] Gupta S. Awareness and utilization of Rashtriya Swasthaya Bima Yojana and its implications for access to health care by the poor in slum areas of Delhi. *Health Systems* 2017;6:242-59.

[96] Chauhan M, Saxena S. Barriers in utilization of public health services by elderly slum dwellers in Jaipur city. *Indian Journal of Public Health Research and Development* 2020;11:730-36.

[97] Oluoch P, Orwa J, Lugalia F, et al. Application of psychosocial models to Home-Based

For peer review only

Testing and Counseling (HBTC) for increased uptake and household coverage in a large informal urban settlement in Kenya. *Pan Afr Med J* 2017;27:285.

[98] Atusiimire LB, Waiswa P, Atuyambe L, et al. Determinants of facility based-deliveries among urban slum dwellers of Kampala, Uganda. *PLoS ONE* 2019;14:e0214995.

[99] Iberico MM, Montoya R, Valiente B, et al. Uptake and utilization of tuberculosis preventive therapy in a Peruvian Peri-urban Shantytown. *Ann Glob Health* 2016;82 (3):366.

[100] Hutain J, Perry HB, Koffi AK, et al. Engaging communities in collecting and using results from verbal autopsies for child deaths: an example from urban slums in Freetown, Sierra Leone. *J Glob Health* 2019;9:010419.

[101] Misra V, Vashist P, Singh SS, et al. Awareness and eye health-seeking practices for cataract among urban slum population of Delhi: The North India eye disease awareness study. *Indian J Ophthalmol* 2017;65:1483-88.

[102] Das M, Angeli F, Krumeich A, et al. The gendered experience with respect to health-seeking behaviour in an urban slum of Kolkata, India. *Int J Equity Health* 2018;17:24.

[103] Sheehy G, Aung Y, Foster AM. "She Learned it from her Mother and Grandmother": Women's Experiences with Delivery and Post-partum Practices in Peri-urban Yangon, Myanmar. *Matern Child Health J* 2016;20:854-61.

[104] Angeli F, Ishwardat ST, Jaiswal AK, et al. Socio-Cultural Sustainability of Private Healthcare Providers in an Indian Slum Setting: A Bottom-of-the-Pyramid Perspective. *Sustainability* 2018;10:4702.

[105] Rahman S. A Comparative Study of Intrauterine Contraceptive Device Utilization among Currently Married Women in a Rural Area of Rani Block and Urban Slums of Guwahati City. *Int J Sci Study* 2016;4:55-59.

[106] Mutua MK, Mohamed SF, Iddi S, et al. Do inequalities exist in the disadvantaged

For peer review only - http://bmjopen.bmj.com/site/about/guidelines.xhtml

populations? Levels and trends of full and on-time vaccination coverage in two Nairobi urban informal settlements. *Glob Epidemiol* 2020;2:100044.

[107] Islam M. Use of reproductive health care services among urban slum women in Bangladesh. *Eur J Public Health* 2018;28:67.

[108] Jolly SP, Rahman M, Afsana K, et al. Evaluation of Maternal Health Service Indicators in Urban Slum of Bangladesh. *PLoS ONE* 2016;11:e0162825.

[109] Sadhna S, Kajal J, Debabratta R, et al. Utilisation of maternal health services and its predictors in slum population. *Acta Medica International* 2016;3:56-62.

[110] Lungu EA, Guda Obse A, Darker C, et al. What influences where they seek care? Caregivers' preferences for under-five child healthcare services in urban slums of Malawi: A discrete choice experiment. *PLoS One* 2018;13:e0189940.

[111] Nasrin M, Sarker MNI, Huda N. Determinants of health care seeking behavior of pregnant slums dwellers in Bangladesh. *Medical Science* 2019;23:35-41.

[112] Castiglione D, Lovasi GS, Carvalho MS. Perceptions and Uses of Public and Private Health Care in a Brazilian Favela. *Qual Health Res* 2018;28:159-72.

[113] Getachew S, Kaba M, Gizaw M, et al. Health service access, utilization and prevailing health problems in the urban vulnerable sections of Ethiopia. *Ethiop J Health Dev* 2020;34:12-23.

[114] Tabrizi JS, Farahbakhsh M, Bazargani HS, et al. Health Services Utilization and Responsiveness: A comparison of Slum and Non-slum Regions in Tabriz, Iran. *Medical Science* 2018;22:577-82.

[115] Agrawal D, Tyagi N, Dhakar JS, Chaturvedi M. Awareness and utilization of Geriatric Welfare Schemes among urban elderly population of District Gautam Budh Nagar. *Indian J Community Health* 2019;31:315-21.

For peer review only - http://bmjopen.bmj.com/site/about/guidelines.xhtml

[116] Dasgupta P, Bhattacherjee S, Mukherjee A, et al. Vaccine hesitancy for childhood vaccinations in slum areas of Siliguri, India. *Indian J Public Health* 2018;62:253-58.

[117] Tebekaw Y, Mashalla YJ, Thupayagale-Tshweneagae G. The adequacy of antenatal care services among slum residents in Addis Ababa, Ethiopia. *Ann Glob Health* 2016;82:527-28.

[118] Aleemi AR, Khaliqui H, Faisal A. Challenges and Patterns of Seeking Primary Health Care in Slums of Karachi: A Disaster Lurking in Urban Shadows. *Asia Pac J Public Health* 2018;30:479-90.

[119] Viramgami AP, Verma PB, Vala MC, et al. A Cross-Sectional Study to Assess Reproductive and Child Health Profile of Working Women Residing in Urban Slums of Rajkot City. *Indian J Community Med* 2019;44:313-16.

[120] van der Heijden J, Gray N, Stringer B, et al. 'Working to stay healthy', health-seeking behaviour in Bangladesh's urban slums: a qualitative study. *BMC Public Health* 2019;19:600.

[121] Gaiha SM, Gillander Gadin K. 'No time for health:' exploring couples' health promotion in Indian slums. *Health Promot Int* 2020;35:70-81.

[122] Wingfield T, Tovar MA, Huff D, et al. Socioeconomic support to improve initiation of tuberculosis preventive therapy and increase tuberculosis treatment success in Peru: a household-randomised, controlled evaluation. *Lancet* 2017;389:S16.

[123] Muralidharan A. Constrained Choices? Menstrual Health and Hygiene Needs Among Adolescents in Mumbai Slums. *Indian J Gender Stud* 2019;26:12-39.

[124] MacPherson P, Khundi M, Nliwasa M, et al. Disparities in access to diagnosis and care in Blantyre, Malawi, identified through enhanced tuberculosis surveillance and spatial analysis. *BMC Med* 2019;17:21.

For peer review only

For peer review only - http://bmjopen.bmj.com/site/about/guidelines.xhtml

[125] Cernauskas V, Angeli F, Jaiswal AK, et al. Underlying determinants of health provider choice in urban slums: results from a discrete choice experiment in Ahmedabad, India. *BMC Health Serv Res* 2018;18:473.

[126] Schultz JS, Muema S, Ouma A, et al. Timeliness of vaccination of vacination in an urban slum in Nairobi, Kenya. *Am J Trop Med Hyg* 2017;95:567.

[127] Sharma S, Sarathi Mohanty P, Omar R, et al. Determinants and Utilization of Maternal Health Care Services in Urban Slums of an Industrialized City, in Western India. *J Family Reprod Health* 2020;14:95-101.

[128] Ahmed S, Adams AM, Islam R, et al. Impact of traffic variability on geographic accessibility to 24/7 emergency healthcare for the urban poor: A GIS study in Dhaka, Bangladesh. *PLoS One* 2019;14:e0222488.

[129] Muhammad A, Khan U, Yazdani AT, et al. Unveiling and addressing implementation barriers to routine immunization in the peri-urban slums of Karachi, Pakistan: a mixed-methods study. *Health Res Policy Syst* 2021;19:55.

[130] Williams A, Sarker M, Ferdous ST. Cultural Attitudes toward Postpartum Depression in Dhaka, Bangladesh. *Med Anthropol* 2018;37:194-205.

[131] Athie K, Dowrick C, Menezes AL, et al. Anxious and depressed women's experiences of emotional suffering and help seeking in a Rio de Janeiro favela. *Cien Saude Colet* 2017;22:75-86.

[132] Kuria N, Reid A, Owiti P, et al. Compliance with follow-up and adherence to medication in hypertensive patients in an urban informal settlement in Kenya: comparison of three models of care. *Trop Med Int Health* 2018;23:785-94.

[133] Mataboge MLS, Beukes S, Nolte AGW. The experiences of clients and healthcare providers regarding the provision of reproductive health services including the prevention

For peer review only - http://bmjopen.bmj.com/site/about/guidelines.xhtml

of HIV and AIDS in an informal settlement in Tshwane. *Health SA Gesondheid* 2016;21:67-76.

[134] Otieno PO, Wambiya EOA, Mohamed SM, et al. Access to primary healthcare services and associated factors in urban slums in Nairobi-Kenya. *BMC Public Health* 2020;20:981.

[135] Kardalkar S, Sherkhane MS. Utilization of antenatal care package among women of urban slums. *Indian J Public Health Research and Development* 2020;11:517-23.

[136] Abd El Fatah SAM, El Habashy EM, Ismail HAH. Role of receipt of antenatal care in subsequent contraceptive use at primary health care centres serving slum areas of Cairo, Egypt. *Eur J Contracept Reprod Health Care* 2019;24:356-61.

[137] Kalyango E, Kananura RM, Kiracho EE. Household preferences and willingness to pay for health insurance in Kampala City: a discrete choice experiment. *Cost Eff Resour Alloc* 2021;19:21.

[138] Sharma S, Verma PB, Viramgami AP, et al. Analysis of Out-of-Pocket Expenditure in Utilization of Maternity Care Services in Urban Slums of Rajkot City, Gujarat. *Indian J Community Med* 2018;43:215-9.

[139] Wekesah FM, Kyobutungi C, Grobbee DE, et al. Understanding of and perceptions towards cardiovascular diseases and their risk factors: a qualitative study among residents of urban informal settings in Nairobi. *BMJ Open* 2019;9:e026852.

[140] Odhiambo GO, Musuva RM, Odiere MR, et al. Experiences and perspectives of community health workers from implementing treatment for schistosomiasis using the community directed intervention strategy in an informal settlement in Kisumu City, western Kenya. *BMC Public Health* 2016;16:986.

[141] Patil SK, Ahmed MM. Assessment of integrated child development services in urban slums of Belagavi city, Karnataka. *Indian J Public Health Res Dev* 2016;7:208-11.

For peer review only

For peer review only - http://bmjopen.bmj.com/site/about/guidelines.xhtml

[142] Agonigi RC, Carvalho SM, Freire MAM, et al. The production of care in the routine of Family Health Teams. *Rev Bras Enferm* 2018;71:2659-65.

[143] Das Gupta M, Dasgupta R, Kuganathan P, et al. Flies without Borders: Lessons from Chennai on Improving India's Municipal Public Health Services. *J Dev Stud* 2020;56:907-28.

[144] Prado Junior JC, Virgilio TC, Medronho Rde A. Cure rates for tuberculosis in the municipality of Rio de Janeiro, Brazil, in 2012 compared with coverage by, and time of establishment of, Family Health units, and socio-economic and demographic factors. *Ciencia & Saude Coletiva* 2016;21:1491-8.

[145] Banerjee S, Selvaraj K, Bandyopadhyay K, et al. Urban health and nutrition day or only immunisation day? barriers and bottlenecks in implementing Urban health and nutrition day in an Urban primary health centre of Nagpur, Central India. *J Mother Child* 2021;25:51-60.

[146] Ongarora D, Karumbi J, Minnaard W, et al. Medicine Prices, Availability, and Affordability in Private Health Facilities in Low-Income Settlements in Nairobi County, Kenya. *Pharmacy (Basel)* 2019;7:40.

[147] Liu Y, Kong Q, Yuan S, et al. Factors influencing choice of health system access level in China: A systematic review. *PLoS One* 2018;13:e0201887.

[148] Banke-Thomas OE, Banke-Thomas AO, Ameh CA. Factors influencing utilisation of maternal health services by adolescent mothers in Low-and middle-income countries: a systematic review. *BMC Pregnancy Childbirth* 2017;17:65.

[149] Mendoza-Sassi R, Béria J. Health services utilization: a systematic review of related factors. *Cad Saude Publica* 2001;17:819-32.

[150] Lilford RJ, Oyebode O, Satterthwaite D, et al. Improving the health and welfare of people

For peer review only - http://bmjopen.bmj.com/site/about/guidelines.xhtml

who live in slums. *Lancet* 2017;389:559-70.

[151] World Health Organization. Strengthening Health Systems to Improve Health Outcomes, 2007. Available from: https://apps.who.int/iris/handle/10665/43918 [Accessed 25 Dec 2021]

[152] McIntyre D, Thiede M, Birch S. Access as a policy-relevant concept in low- and middle-income countries. *Health Econ Policy Law* 2009;4:179-93.

For peer review only

For peer review only - http://bmjopen.bmj.com/site/about/guidelines.xhtml

Figure legends.

Figure 1. Preliminary framework for factors influencing slum residents' healthcare seeking behaviour and utilization of health services and the provision of services in slum settings

Figure 2. Updated framework of factors influencing healthcare-seeking behaviour/healthcare utilisation/provision of healthcare services in slums.

Figure 3. Flowchart.

For peer review only

For peer review only - http://bmjopen.bmj.com/site/about/guidelines.xhtml

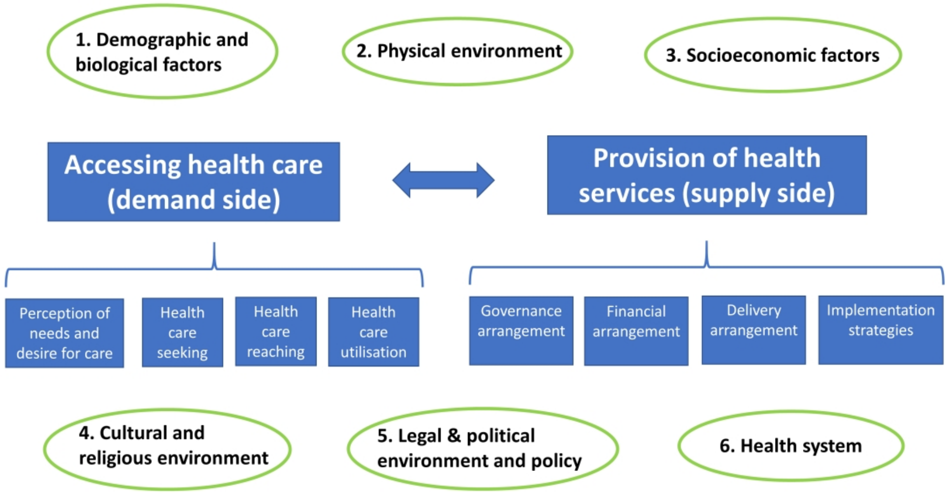

Preliminary framework for factors influencing slum residents' healthcare seeking behaviour and utilization of health services and the provision of services in slum settings

338x190mm (300 x 300 DPI)

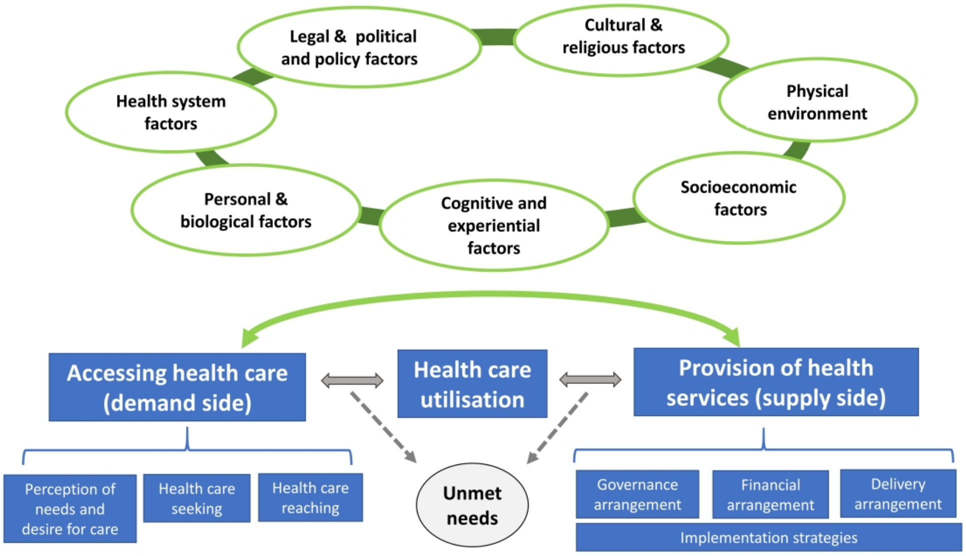

Updated framework of factors influencing healthcare-seeking behaviour/healthcare utilisation/provision of healthcare services in slums.

338x190mm (300 x 300 DPI)

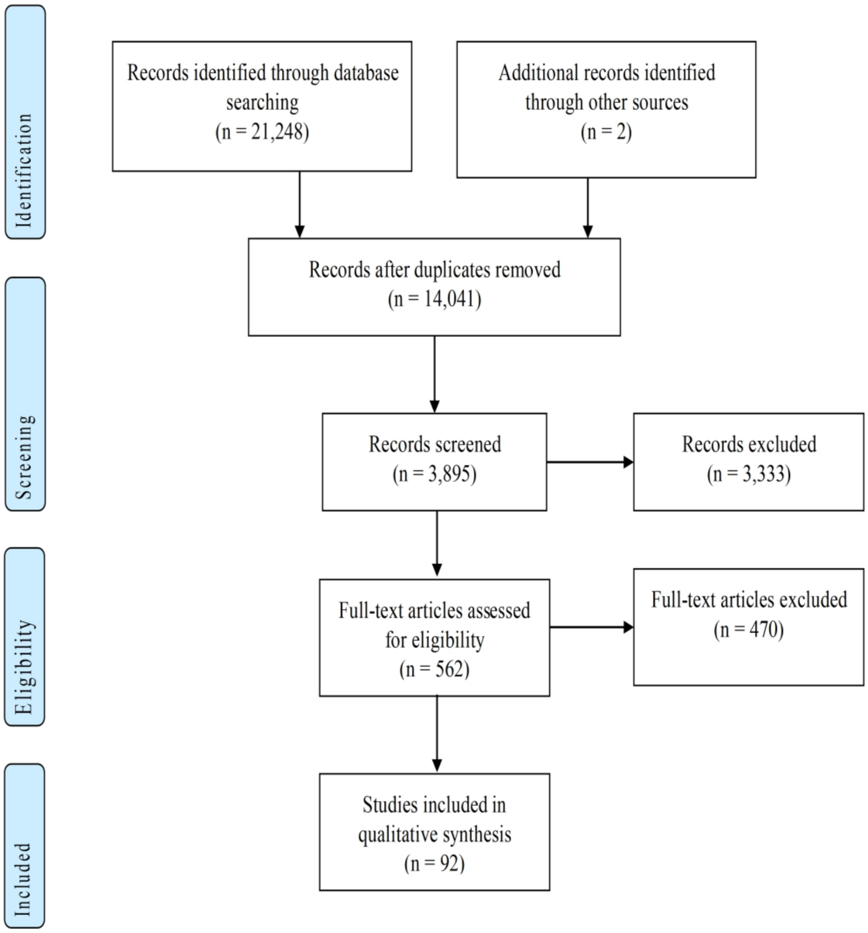

Flowchart

338x451mm (300 x 300 DPI)

Supplement 1. Healthcare-seeking behaviours of slum residents reported by included studies and associated factors.

Subcategory	Author (year)	Participants	Country	Study design	Methodology	Outcome	Factors of interest
General healthcare seeking behaviour	Pakhare (2021)[55]	Slum residents diagnosed hypertension or diabetes	India	Prospective study	Quantitative	Linking to healthcare facilities	Age; wealth; distance to facilities ; early engagement by healthcare workers
	Gaiha (2020)[121]	Hetero-couples in slums	India	Cross-sectional study	Mixed method	Ability to attend any health promotion activity	Lack of time related to work as a reason for low male participation
	van der Heijden (2019)[120]	Female workers and key informants in slums	Bangladeshi	Cross-sectional study	Qualitative	Healthcare-seeking behaviour	Competing interest (ability to work and income)
	Aleemi (2018)[118]	Slum residents	Pakistan	Cross-sectional study	Quantitative	Healthcare-seeking behaviour	Household income; government facility; NGO facility
	Wekesah (2019)[139]	Slum residents	Kenya	Cross-sectional study	Qualitative	Care-seeking and adherence to treatment for CVD	Cost of healthcare; lack of healthcare facilities
	Kar (2017)[58]	Slum residents	India	Cross-sectional study	Quantitative	Undiagnosed hypertension	Sex; poverty; unskilled laborer; literacy
	Mistry (2016)[71]	TB patients in slums	India	Retrospective study	Quantitative	Delays in care seeking	Perception of symptoms; home remedies; not want to miss work; provider shopping; delay in advising TB-relevant tests; referral.
	Kulkarni (2016)[70]	Women in slums	India	Cross-sectional study	Quantitative	Participation in breast cancer screening	Age; education; religion; Mother tongue; occupation; marital status; tobacco habits; family history of cancer; history of cancer screening
	Misra (2017)[101]	Slum households	India	Cross-sectional study	Quantitative	Health-seeking practice for cataract	Lack of time, fear of surgery, financial difficulties

For peer review only

	Ramagiri (2020)[77]	Slum residents with diabetes	India	Case control study	Mixed-method	Uptake of diabetic retinopathy screening	Realization of consequences of disease; travel assistance and proximity of the screening facility; absence of an accompanying person; cost
Healthcare for children	Mohanty (2021)[54]	Caregivers of under-five children in urban slums,	India	Cross-sectional study	Quantitative	Healthcare seeking for children	Sex of child; size of the household; social group of caregiver, mother with mass media knowledge; age of mother; education and occupation of mother; suffering from chronic disease; decision making person for seeking health care; time lapse in approaching the health care facility; income loss due to children illnesses
	Lungu (2020)[53]	Caregivers of children under 5 years of age in slums	Malawi	Cross-sectional study	Quantitative	Healthcare-seeking behaviour	Age; illness was perceived to be severe; fever; home management of childhood illness
						Timely healthcare seeking behaviour	Home management of childhood; knowledge of caregivers about child danger signs
	McNairy (2019)[19]	Slum households with children ≤ 5 years old	Haiti	Cross-sectional study	Quantitative	Healthcare access	Inability to afford care
	Hutain (2019)[100]	Caregiver at the time of the child's death in slums	Sierra Leone	Cross-sectional study	Mixed-method	Health care-seeking	Use of traditional medicine; difficultly reaching the health facility; doubts about need for medical care; mistreatment by staff
	Kerai (2019)[56]	Caregiver of children aged 2 months to 5 years in slums	Pakistan	Cross-sectional study	Quantitative	Healthcare-seeking behaviour	Age of child; gender of child; income; education of caretaker; vaccine awareness; breastfeeding awareness;

For peer review only

							presence of symptoms such as fever, tachypnea, chest indrawing, persistent vomiting, recurrent illness.
	Lungu (2018)[110]	Caregivers of children under 5 years of age in slums	Malawi	Prospective study	Quantitative	Healthcare-seeking behaviour	Cost; waiting time; availability of medicines and supplies; attitude of health workers; thorough examination of the child
						Willingness to pay for the health facility	Waiting time; availability of medicine and equipment; superficial or thorough examination; attitude of health workers
	Kamati (2019)[73]	Slum residents	Namibia	Cross-sectional study	Mixed-method	Self-medication	Perceived diagnosis as "minor or mild"; waiting times and queues to receive care
	Mishra (2017)[65]	Mothers living in slums with a child and migrated recently	India	Cross-sectional study	Quantitative	Healthcare seeking behaviour	Symptoms and severity
	Lungu (2016)[85]	Caregivers and health providers in slums	Malawi	Longitudinal study	Qualitative	Healthcare-seeking behaviour	Home management; lack of medicines and supplies; waiting times; facility opening times; attitude of health workers; suboptimal examination of the sick child; distance to health facility; cost of healthcare
Healthcare for women	Muralidharan (2019)[123]	Girls and mothers in slums	India	Cross-sectional study	Qualitative	Healthcare-seeking behaviour	Proximity of healthcare facilities
	Nasrin (2019)[111]	Married women with a child in slums	Bangladesh	Cross-sectional study	Mixed-method	Healthcare-seeking behaviours	Inability to spend the treatment cost

For peer review only

	Jayaweera (2018)[79]	Girls and women in slums	Kenya	Cross-sectional study	Qualitative	Access to contraception and abortion in health facilities	Stigma; lack of education about safe methods of abortion; perceived illegality of abortion; limited access to services because of financial barrier; fear of mistreatment and mistrust of health providers/facilities; geographical proximity
	Williams (2018)[130]	Mothers and medical personnel in slums	Bangladesh	Cross-sectional study	Qualitative	Mental healthcare seeking	Culture and stigma
	Ilankoo (2018)[78]	Women in slums	Sri Lanka	Cross-sectional study	Qualitative	Health-seeking behaviours related to vaginal discharge	Confusion in differentiating normal from abnormal vaginal discharge; effects on day-to-day life; confusion toward the causative factors; difficulties in disclosing; neglecting behaviours; and socio-cultural influences toward health-seeking behaviours.
	Athie (2017)[131]	Anxious and depressed women in slums	Brazil	Cross-sectional study	Qualitative	Healthcare seeking behaviour	High medical turnover and overload of healthcare providers
	Sudhinaraset (2016)[90]	Mothers and their families in slums	India	Cross-sectional study	Qualitative	Maternal health services and delivery experiences	Financial barriers; disrespectful care
	Pune Municipal corporation[38]	Recently delivered slum residents	India	Cross-sectional study	Mixed-method	Seeking front-line worker during labor	No time to call; family did not allow; being out of town; lack of trust; delivery at night
						Going to the Referred Place for Pregnancy Complications	Not necessary; family did not allow; lack of trust/poor quality services; don't like going to a difference facility; too far; cost; no transportation; private hospital
Preference for	Das (2018)[102]	Slum	India	Cross-	Qualitative	Healthcare-seeking	Female prefer informal healers

For peer review only - http://bmjopen.bmj.com/site/about/guidelines.xhtml

healthcare providers		residents		sectional study		practice (preference for formal/informal healers)	(cultural competency of care, easy communication, gender-induced affordability, avoidance of social stigma and labelling, living with the burden of cultural expectations and geographical and cognitive distance of formal health care)
							Male prefer formal care (ease of access, quality of treatment, expected outcome of therapies)
	Angeli (2018)[104]	Slum residents	India	Cross-sectional study	Mixed-method	Choice between public or private hospital	Bottom-of-the pyramid patients visit a public hospital more than top-of-the-pyramid patients
Health insurance	Kalyango[*] (2021)[137]	Households in slum and non-slums	Uganda	Cross-sectional study	Qualitative	Willingness to pay for health insurance	Public and private providers; extended family enrolment
HIV testing	Thomson (2018)[72]	Stakeholder including residents and healthcare service provider	Kenya	Cross-sectional study	Qualitative	HIV testing	Denial; complacency; fear of death; anticipation of unbearable stress; felt ill; had a partner die; learned that their partner was HIV-positive.
Expenditure	Mishra (2017)[59]	Slum households with a child aged 0–14 years and who had migrated within the last 12 years	India	Cross-sectional study	Quantitative	Treatment-seeking behaviour	Child's gender

[*]Factors reported in the study were associated with participants covering both slum and non-slum residents. CVD: cardiovascular disease; HIV: human immunodeficiency virus; NGO: non-governmental organization; TB: tuberculosis.

For peer review only

Supplement 2. Healthcare utilisation of slum residents reported by included studies and associated factors

Subcategory	Author (year)	Participants	Country	Study design	Methodology	Outcome	Factors of interest
General utilisation	Wambiya (2021)[64]	Slum household members	Kenya	Cross-sectional study	Quantitative	Private and public healthcare utilisation	Public- satisfaction with cost; satisfaction with healthcare quality; having acute infection or other diseases<br><br>Private- insurance coverage; having acute infection
	Chauhan (2020)[96]	Elderly slum residents	India	Cross-sectional study	Quantitative	Utilization of healthcare services	Unawareness of healthcare facilities; behaviour of service providers; distance from home; transport facility; amenities at healthcare facilities; convenience for attendants
	Otieno (2020)[134]	Slum household members	Kenya	Cross-sectional study	Quantitative	Access to primary healthcare services	Sex of household head; average out-of-pocket healthcare expenditure; source of primary care
	Vora (2020)[46]	Slum household members	India	Cross-sectional study	Quantitative	Unmet need for surgical services	Financial reasons; lack of trust; age; sex; type of problem
	Agrawal (2019)[115]	Older adults in slums	India	Cross-sectional study	Quantitative	Utilisation of welfare schemes	Religion; Caste; education;
	Ahmed (2019)[128]	N/A	Bangladesh	Cross-sectional study	Quantitative	Access to, and availability of healthcare services	Variability in traffic congestion
	Madan (2019)[87]	Female slum residents	India	Cross-sectional study	Qualitative	Access to primary care	Long waiting times and opening times of the primary health care; quality of services; satisfaction with treatments; home remedies; cost; rude attitude of healthcare providers
	Owiti (2018)[86]	Pregnant women in slums	Kenya	Cross-sectional study	Quantitative	Utilisation of maternal health services in public	Perception about public health facility delivery; living within close proximity; waiting time at

For peer review only

					health facilities	the facility; learning about the program; quality of service; ANC attendance at a private and a non-profit health facility
Castiglione (2018)[112]	Slum residents	Brazil	Cross-sectional study	Qualitative	Barrier to healthcare services	*Public healthcare services:* structural aspects of the healthcare system in their community as a whole, such as scarcity of personnel and equipment, or long waiting periods; experiences of conflict when dealing with doctors and other professionals of the public healthcare system<br><br>*Private healthcare services*: Insufficient funds to seek assistance; services or products in the private sector;
Tabrizi* (2018)[114]	Households in slum and non-slums	Iran	Cross-sectional study	Quantitative	Utilisation of health services	High cost of services
					Home care services	High cost of services
					Not taking drugs prescribed	Slums:<br>financial problems<br><br>Non-slums:<br>getting better/feeling well
Wairiuko (2017)[88]	Elderly in slums	Kenya	Cross-sectional study	Mixed-method	Health service utilisation	Family support; satisfaction with healthcare services; gender healthcare worker preference; services by community health worker
Owusu-Ansah (2016)[83]	Slum residents	Ghana	Cross-sectional study	Qualitative	Utilization of healthcare	Education; occupation; NHIS membership; knowledge of symptom; overall knowledge score; transportation

For peer review only

Adane (2017)[81]	Mothers/caregivers of under-five children in slums	Ethiopia	Cross-sectional study	Quantitative	Utilization of healthcare facilities in children with diarrhoea	Mothers/caregivers education; occupation; time of walking to the nearest health facility; household monthly income; recognized danger signs
MacPherson (2019)[124]	Slum residents	Malawi	Prospective study	Quantitative	Access to TB diagnosis	Distance to the nearest TB registration clinic
Wingfield (2017)[122]	Slum households with patients treated for TB	Peru	Randomized controlled study	Quantitative	Initiation of TB preventive therapy	Socioeconomic support and social support
Iberico (2016)[99]	Healthcare workers and community members in slums	Peru	Cross-sectional study	Qualitative	Utilization of TB preventive therapy	Misunderstanding and fear of treatment
Snyder[*] (2016) [52]	TB patients living in slum and non-slum	Brazil	Retrospective study	Quantitative	Abandonment of TB treatment	Residency in a slum; sex; age; extrapulmonary clinical disease; HIV/AIDS; interaction (directly observed treatment × residency in a slum)
Oluoch (2017)[97]	Slum residents	Nairobi	Cross-sectional study	Quantitative	Attendance to HIV testing and counselling services	Previous test experience
Martinez Perez (2016)[89]	Healthcare workers and community members in slums	South Africa	Cross-sectional study	Mixed method	HIV Counselling and Testing	Fear; lack of trust
Amiresmaili (2019)[18]	Slum residents	India	Cross-sectional study	Quantitative	Utilisation of outpatients services	Gender; marital status
					Utilisation of inpatients services	Age of household head; marital status; insurance
Horng (2019)[49]	Slum households with children under 5 years old who either recently	Bangladesh	Cross-sectional study	Quantitative	Healthcare utilisation in severe acute respiratory illness	Relocation; age of child; education of mother; household wealth; health service knowledge

For peer review only

		relocated <12 months or who were residentially stable living >24 months				Full vaccination coverage	Relocation; number of children in household; age of child; education of mother; occupation of household head; household wealth; health service knowledge
	Kuria (2018)[132]	Patients received hypertension treatment in slums	Kenya	Retrospective study	Quantitative	Compliance with hypertensive treatment	Health facility group than walkway or weekend clinic attenders
	Cernauskas (2018)[125]	Slum residents	India	Cross-sectional study	Quantitative	Health provider choice	Distance to health facilities; friendly attitude of healthcare workers; appropriate service; familiarity
	Kaba (2020)[74]	Stakeholders (community members, community opinion leaders, health professionals, health office representatives.)	Ethiopia	Cross-sectional study	Qualitative	Utilisation of health services	Individual level: awareness about health problems; competing priorities; capacity to pay for services when referred.
	Mataboge (2016)[133]	Health services' clients and healthcare providers in an informal settlement	South Africa	Cross-sectional study	Qualitative	Healthcare utilisation	Long waiting time
Immunisation	Muhammad (2021)[129]	Caregivers of children, community influencers, immunisation staff in peri-urban slums	Pakistan	Cross-sectional study	Mixed-method	Childhood vaccination	Permission for immunisation by decision-maker; lack of knowledge and awareness of the benefit of immunisation; misconceptions and fears regarding vaccines; social and religious barriers
	de Araujo Veras (2020)[45]	Children in slums	Brazil	Cross-sectional study	Quantitative	Childhood vaccination	Age of child: mother's education

For peer review only

Mutua (2020)[106]	Children in slums	Nairobi	Prospective study	Quantitative	Full and on-time vaccination coverage	Place of residence; wealth
Roja (2020)[44]	Mothers of children in slums	India	Cross-sectional study	Quantitative	Immunisation status of children	Number of children in family; age of child; father's education
Obanewa (2020)[60]	Rural/urban formal/slum residents	Nigeria	Retrospective cross-sectional study	Quantitative	Fully-immunized child coverage	For slums: delivery place; maternal education; birth order; antenatal attendance; religion<br><br>For slum and non-slums: year; birth order; antenatal attendance; maternal education; religion; maternal age at child's birth; media exposure; region of the country; interaction between place of residence and place of delivery
Viramgami (2019)[119]	Married slum residents in reproductive age	India	Cross-sectional study	Quantitative	Vaccination status of child	Mother's employment
Singh (2018)[68]	N/A	India	Literature review	-	Childhood vaccination	Fear of adverse events; lack of information/knowledge; disease not harmful/serious; parents busy; income; mother's education; travel/transfer/migration; unawareness of need for health services; faith in immunisation; mother ill; forgetfulness; lack of initiative; family problems; services not available/lack of facility; shortages/reluctant to open 10 dose vials for 1 or 2 infants; current/history of sickness lead to withhold the vaccine

For peer review only

Pugliese-Garcia (2018)[76]	Stakeholders including slum residents, healthcare workers, health committee members, vaccinators	Zambia	Cross-sectional study	Qualitative	Vaccine hesitancy	Traditional remedies; alcohol use; religious beliefs; distrust towards western medicine; previous adverse events; fear of injections and low perceived need for immunisation; limited understanding of how vaccines work; overlapping local terms for vaccine; pain; perceived risk of infection
Manandhar (2018)[93]	Slum household with children age of 12-60 months	Nepal	Cross-sectional study	Quantitative	Incomplete immunisation	Knowledge on immunisation schedule
Dasgupta (2018)[116]	Slum household with children aged 0-59 months, resides in the study area for the past 12 months	India	Cross-sectional study	Quantitative	Vaccine hesitancy	Family type; education of mother
Lae (2018)[50]	Caregivers in slums	Myanmar	Cross-sectional study	Qualitative	Utilisation of immunisation services	Age of child; income; migration; antenatal visit; receiving additional vaccines before; having immunisation card.
Schultz (2017)[126]	Parents with children <5 years old in slums	Kenya	Prospective study	Quantitative	Timeliness of vaccination	Close to the clinic; birth in December
Crocker-Buque (2017)[21]	People living in a low-income urban area or slum in a low-middle income countries	Multiple nations	Systematic review	-	Immunisation coverage	*Socioeconomic and demographic characteristics:* socioeconomic status; wealth; parents' literacy; mother's education; employment; residential status; place of residence; place of delivery; household visit by health workers; premature birth; malnourishment; inadequate housing; poor

						prenatal care; ethnicity; age; maternal age; birth order; sex of child; number of children<br><br>*Migration status:*<br>migration; recent migration<br><br>*Information, beliefs and behaviour:*<br>unaware of the need for vaccines; unaware of clinic location or timing; maternal knowledge of immunisation; lack of access to information; parents being too busy; return to home village; difficulty in accessing services; fear of side effects; attitude of health workers; concerns over cost; being suspicious of free services<br><br>*Health services:*<br>distance from health centre; timing of services; fear of costs; risk of lost income; lack of local knowledge; patients' satisfaction; provision of accurate information; accessing pre-natal care
Shrestha (2016)[82]	Slum households with children aged 12–23 months.	Nepal	Case-control study	Quantitative	Incompletion of immunisation	Home delivery; type of residence; knowledge about healthcare services of primary care-taker; perception towards healthcare services, conflicting priorities, side effect
Devasenapathy (2016)[57]	Slum household with children aged between 12 and 42 months	India	Cross-sectional study	Quantitative	Childhood complete immunisation	Sex; mother's literacy; place of birth; place of childbirth; religion; socioeconomic position; birth certificate

For peer review only

Maternal	Sendo (2021)[92]	Female slum residents	Ethiopia	Cross-sectional study	Qualitative	Delivery in healthcare facilities	Provision of quality, respectful and dignified midwifery care; lack of awareness about facility delivery.
	Kardalkar (2020)[135]	Female delivered within three months in slums	India	Cross-sectional study	Quantitative	Utilization of antenatal care	Literacy; Gravida; occupation
	Sendo (2020)[91]	Women of reproductive age in slums	Ethiopia	Cross-sectional study	Qualitative	Delivery in health facilities	Perceived benefits of home delivery; knowledge deficit about health facility-based delivery; poor access to healthcare facilities; inadequate resources
	Sharma (2020)[127]	Women delivered a baby within one year in slums	India	Cross-sectional study	Quantitative	Utilization of maternal healthcare services	Education; employment of mother; category and type of family; distance and time to reach health facility;
	Yadav (2020)[42]	Married women in slums	India	Cross-sectional study	Quantitative	Unmet need for family planning services	Age; educational status; duration of marriage; number of pregnancies; knowledge of contraceptive methods; opposition to contraceptive use; contact with a midwife
	Razzaque (2020)[66]	Slum residents	Bangladeshi	Cross-sectional study	Quantitative	Healthcare utilisation	Recent migration; wealth; education; employment
	Getachew (2020)[113]	Slum households	Ethiopia	Cross-sectional study	Quantitative	Delivery in healthcare facilities	Perceived as not customary to deliver at health facility; not necessary; unavailability of female birth attendants; perceived quality of services; cost
	Shrestha (2019)[61]	Mothers with infant residing in slums	Nepal	Cross-sectional study	Quantitative	Utilisation of antenatal and delivery services	Educational status of respondents and their husbands; number of pregnancy
						Institutional delivery	Educational status; occupation of husband; number of pregnancy

For peer review only

					Postnatal visit	Occupation of husband
					Utilisation of family planning services	Occupation of husband
					Tetanus Toxoid immunisation	Educational status of respondents; economic status; knowledge about healthcare services; educational status of husband; number of pregnancies
Atusiimire (2019)[98]	Mothers delivered in the past one year in slums	Uganda	Cross-sectional study	Quantitative	Facility based–deliveries	Exposure to media concerning facility delivery; frequency of ANC; timing of 1st ANC
Upadhyai (2019)[39]	Recently delivered mothers residing in slums	India	Cross-sectional study	Quantitative	Healthcare utilisation	Age; education of mother and father; socioeconomic class; antenatal check-ups; institutional delivery services; family type; caesarean delivery; complication or perceived health problem
Angeles[*] (2019)[47]	Slum and non-slum residents	Bangladesh	Prospective study	Quantitative	Use of modern contraceptive methods	Parity, mother's age; mother's education, socioeconomic status, interaction (slum × time period)
					Delivery by skilled birth attendant	Residing in slums, parity, mother's age, mother's education, length of stay in current city of residence, socioeconomic status, number of available community health worker, distance from health facility, interaction (slum x time period)
Kusuma (2018)[80]	Recent migrant and settled mothers with a child up to the age of 1 year in slums	India	Cross-sectional study	Quantitative	Birth in health facility	Listening to radio; number of ANC visits; plan for hospital birth; plan for transport; some danger sign; knowledge of danger sign

For peer review only

Sharma (2018)[138]	Women living in urban slums and delivered a baby within 1 year	India	Cross-sectional study	Quantitative	Utilisation of maternal care services	Mode of delivery; hospital stay after delivery
Islam* (2018) [107]	Ever-married women aged 15-49 years living in slum and non-slum	Bangladesh	Cross-sectional study	Quantitative	ANC visits	Education; wealth index of the household
Geddam (2017)[67]	Rural to urban internal migrant mothers with a child of less than 2 years of age	India	Cross-sectional study	Quantitative	Utilisation of maternal health services	Education of the mother; family size; occupation of mother
					Delivery in institution	Educational status of mother; number of ANC visit; adequacy of ANC; migration status
Kaba (2017)[94]	Stakeholders including city administrators, community members, healthcare providers	Ethiopia	Cross-sectional study	Qualitative	Maternal health service utilisation	Lack of awareness and lack of perceived needs about available services; fear of stigma; competing priorities, social connectedness; perceived lack of respectful service providers; socio-cultural factors including socially sanctioned expectations
Verma (2017)[75]	Pregnant women and infants in slums	India	Case-control study	Mixed-method	Antenatal care registration/immunisation	Knowledge of healthcare services; perceived need for healthcare services; family support; fear; negative experience with previous vaccination
Sharma (2016)[51]	Married women in slums	Nepal	Cross-sectional study	Quantitative	Antenatal healthcare utilisation	Age; husband education; spouse occupation; family income; type of family; planned pregnancy; death of children
Jolly (2016)[108]	Married women with a pregnancy outcome in the previous year in slums	Bangladesh	Cross-sectional study	Quantitative	Antenatal care; birth assisted by medically trained provider; postnatal care; treatment seeking for	Education; wealth

						delivery complications	
						Use of modern family planning	Wealth
	Tebekaw (2016)[117]	Women in slums	Ethiopia	Cross-sectional study	Quantitative	Antenatal care services	Education; private/public hospital
	Sadhna (2016)[109]	Married women in slums	India	Cross-sectional study	Quantitative	Utilisation of maternal health services	Education; Caste; wealth; distance to preferred health facility
	Neyaz (2016)[62]	Married women in slums	India	Cross-sectional study	Quantitative	Delivery in hospitals	Received ANC; number of ANC visits; education; birth order; living index
	Rahman (2016)[105]	Married women in rural and slum area	India	Cross-sectional study	Quantitative	Intrauterine contraceptive device utilisation	Income; occupation
	Sheehy (2016)[103]	Informant and women in slums	Myanmar	Cross-sectional study	Qualitative	Giving birth in hospital	Financial constraints; lack of transportation; sociocultural and financial considerations
Contraceptive	Renzaho (2017)[48]	Slum residents aged 13-24	Uganda	Cross-sectional study	Quantitative	Access to contraceptive services and family planning	Age; disability
	Abd El Fatah (2019)[136]	Married women aged 15–49 years in slums	Egypt	Cross-sectional study	Quantitative	Contraceptive use	Number of male children
Health insurance	Iyalomhe (2021)[41]	Slum residents	Nigeria	Cross-sectional study	Quantitative	Healthcare insurance coverage	Age; sex; marriage; income; religion; education
	Mendhe (2021)[40]	Female slum residens	India	Cross-sectional study	Quantitative	Healthcare insurance coverage	Socioeconomic status;
						Out of pocket expenditure	Age; government/ private hospital
	Otieno (2019)[84]	Slum residents	Kenya	Cross-sectional study	Quantitative	Enrolment in a health insurance programme	Employment; source of primary care; satisfaction with cost of care; satisfaction with procedure of care; perceived health status
	Kusuma (2018)[69]	Slum residents	India	Cross-	Quantitative	Health insurance	Residential background (old

For peer review only

				sectional study		possession	slums than new); migration period; possession of ration card; household size; occupation of household head
	Gupta (2017)[95]	Slum households having health insurance cards	India	Cross-sectional study	Mixed-method	Utilisation of healthcare insurance	Awareness of the empanelled hospitals; experiences of friends and relatives at national health insurance empanelled hospitals; hospitals refused to accept health insurance cards
Expenditure	Sahu (2017)[63]	Women delivered within a period of 6 weeks in slums	India	Cross-sectional study	Quantitative	Out-of-pocket expenditure for maternal and neonatal health services	Gravidity; type of delivery; place of delivery; morbidity
	Mishra (2017)[59]	Slum households with a child aged 0–14 years and who had migrated within the last 12 years	India	Cross-sectional study	Quantitative	Out-of-pocket expenditure	Child's gender; mother's education; type of illness

*Factors reported in the study were associated with participants covering both slum and non-slum residents. ANC: antenatal care; CVD: cardiovascular disease; HIV: human immunodeficiency virus; N/A: not applicable; NGO: non-governmental organization; TB: tuberculosis.

For peer review only

Supplement 3. Provision of healthcare services in slums examined by included studies and associated factors

Subcategory	Author (year)	Participants	Country	Study design	Methodology	Outcome	Factors of interest
General provision	Banerjee (2021)[145]	Community-level service providers in the selected city of Nagpur, Maharashtra.	India	Cross-sectional study	Mixed-methods	Implementing urban health and nutrition day	Unserved areas and left-out urban slum pockets; the distribution paradox of Urban Health and Nutrition Day location with an ill-defined geographic boundary; restriction of range of services to antenatal registration and immunisation with gross neglect of other components; suboptimal training of staff; insufficient availability of space, logistics, and health manpower; non-involvement of community members and Urban Local Bodies; and poor monitoring and supervision.
	Muhammad (2021)[129]	Caregivers of children, community influencers, immunisation staff in peri-urban slums	Pakistan	Cross-sectional study	Mixed-method	Childhood vaccination	Underperformance of staff; unreliable immunisation and household data; inefficient utilization of funds; interference of polio campaigns with immunisation
	Kaba (2020)[74]	Stakeholders (community members, community opinion leaders, Urban Health Extension Professionals, and	Ethiopia	Cross-sectional study	Qualitative	Provision of health services	Institutional-level: medical supplies; a lack of passion; attitudes on the part of health service providers<br><br>Community level: shared understanding of the

For peer review only

	city health office representatives.)					problems; services and the community's established values in relation to the problems and services.
Das Gupta (2020)[143]	N/A	India	Case study	Mixed-method	Improving public health services	Devolution of service delivery transferring funds and responsibilities to elected local bodies; management by professional managerial and technical cadres; Tight organisation of public health services; Professional support from the state directorate of public health
Ongarora (2019)[146]	Private healthcare facilities	Kenya	Cross-sectional study	Quantitative	Provision of medicine	Medicine price, affordability and availability of medicine
Agonigi (2018)[142]	Health professionals	Brazil	Cross-sectional study	Qualitative	Production of care in the daily work of health professionals	Issues related to assignment of tasks; inadequate space and equipment; requirement to follow standardised protocol; demands from the management; workload; environment (sanitation, territory); violence; registration
Odhiambo (2016)[140]	Community health workers	Kenya	Longitudinal study	Quantitative	Drug administration activities for schistosomiasis	Community health worker familiarity with households led to warm reception; good knowledge of intervention area by community health workers; high demand for drugs in the final year of treatment; effective community mobilization; opportunity to integrate mass drug administration with other health interventions; presence of community health workers and their supervisory structure, and

For peer review only

						points of referral for serious side effects; fear of side effects, size of tablet and misconceptions regarding treatment; unrelated death and the associated negative publicity by the media; religious beliefs and mistrust of interventions; insufficient time; absence of community members during the drug administration exercise; difficulty in directly observing treatment; unsanitary environmental conditions; inaccessibility (filthy and bush environment); demand for incentives by community members to take drugs.
Patil (2016)[141]	Healthcare service centres	India	Cross-sectional study	Quantitative	Services provided under Integrated Child Development Services	Lack of basic infrastructural facilities; absence of essential drugs, equipment and logistics; poor pay scale, untimely drug supply, poor community support, more of documentation work, increased work burden, lack of supportive staff and no incentives for the increased work
Mataboge (2016)[133]	Health services' clients and healthcare providers in an informal settlement	South Africa	Cross-sectional study	Qualitative	Provision of reproductive healthcare services	Healthcare policies; work overload; community-based care
Prado Junior (2016)[144]	New TB cases living in slum and non-slum	Brazil	Cross-sectional study	Quantitative	Coverage under Family Health system for TB patients	Policy prioritizing low social development areas

TB: tuberculosis

For peer review only

Appendix 1. Search strategy and the result of each database.

Database	Search strategy	Number of studies
Ovid Medline	1. (informal* and settlement*).ti,ab,kw.<br>2. (shanty and town*).ti,ab,kw.<br>3. (favela* or ghetto* or shantytown* or shanty-town* or slum or slums).ti,ab,kw.<br>4. or/1-3<br>5. limit 4 to english language	4,688
Embase	1 (informal* and settlement*).ti,ab,kw.<br>2 (shanty and town*).ti,ab,kw.<br>3 (favela* or ghetto* or shantytown* or shanty-town* or slum or slums).ti,ab,kw.<br>4 or/1-3<br>5 limit 4 to english language	5,090
Web of Science	1 (TS=(favela* OR ghetto* OR shantytown* OR shanty-town* OR slum OR slums)) AND language: (English)<br>2 ((TS=(informal* NEAR settlement*))) AND language: (English)<br>3 (TS=(shanty NEAR town*)) AND language: (English)<br>4 (#1 OR #2 OR #3) AND language: (English)	3,553
Cochrane	1 (informal* and settlement*).ti,ab,kw.<br>2 (shanty and town*).ti,ab,kw.<br>3 (favela* or ghetto* or shantytown* or shanty-town* or slum or slums).ti,ab,kw.<br>4 #1 or #2 or #3	381
CINAHL	1 TI (informal* and settlement*) OR AB (informal* and settlement*)<br>2 TI (shanty and town*) OR AB (shanty and town*)<br>3 TI (favela* or ghetto* or shantytown* or shanty-town* or slum or slums) OR AB (favela* or ghetto* or shantytown* or shanty-town* or slum or slums)<br>S1 OR S2 OR S3	1,757

Preferred Reporting Items for Systematic reviews and Meta-Analyses extension for Scoping Reviews (PRISMA-ScR) Checklist

SECTION	ITEM	PRISMA-ScR CHECKLIST ITEM	REPORTED ON PAGE #
**TITLE**			
Title	1	Identify the report as a scoping review.	1
**ABSTRACT**			
Structured summary	2	Provide a structured summary that includes (as applicable): background, objectives, eligibility criteria, sources of evidence, charting methods, results, and conclusions that relate to the review questions and objectives.	2
**INTRODUCTION**			
Rationale	3	Describe the rationale for the review in the context of what is already known. Explain why the review questions/objectives lend themselves to a scoping review approach.	4
Objectives	4	Provide an explicit statement of the questions and objectives being addressed with reference to their key elements (e.g., population or participants, concepts, and context) or other relevant key elements used to conceptualize the review questions and/or objectives.	5
**METHODS**			
Protocol and registration	5	Indicate whether a review protocol exists; state if and where it can be accessed (e.g., a Web address); and if available, provide registration information, including the registration number.	5
Eligibility criteria	6	Specify characteristics of the sources of evidence used as eligibility criteria (e.g., years considered, language, and publication status), and provide a rationale.	6-7
Information sources*	7	Describe all information sources in the search (e.g., databases with dates of coverage and contact with authors to identify additional sources), as well as the date the most recent search was executed.	5-6
Search	8	Present the full electronic search strategy for at least 1 database, including any limits used, such that it could be repeated.	5-6, appendix1
Selection of sources of evidence†	9	State the process for selecting sources of evidence (i.e., screening and eligibility) included in the scoping review.	5-7
Data charting process‡	10	Describe the methods of charting data from the included sources of evidence (e.g., calibrated forms or forms that have been tested by the team before their use, and whether data charting was done independently or in duplicate) and any processes for obtaining and confirming data from investigators.	7-9
Data items	11	List and define all variables for which data were sought and any assumptions and simplifications made.	7-9
Critical appraisal of individual sources of evidence§	12	If done, provide a rationale for conducting a critical appraisal of included sources of evidence; describe the methods used and how this information was used in any data synthesis (if appropriate).	N/A

For peer review only

SECTION	ITEM	PRISMA-ScR CHECKLIST ITEM	REPORTED ON PAGE #
Synthesis of results	13	Describe the methods of handling and summarizing the data that were charted.	7-9
**RESULTS**			
Selection of sources of evidence	14	Give numbers of sources of evidence screened, assessed for eligibility, and included in the review, with reasons for exclusions at each stage, ideally using a flow diagram.	10-11, Figure 1
Characteristics of sources of evidence	15	For each source of evidence, present characteristics for which data were charted and provide the citations.	Supplement 1-3
Critical appraisal within sources of evidence	16	If done, present data on critical appraisal of included sources of evidence (see item 12).	N/A
Results of individual sources of evidence	17	For each included source of evidence, present the relevant data that were charted that relate to the review questions and objectives.	Table 2-3
Synthesis of results	18	Summarize and/or present the charting results as they relate to the review questions and objectives.	13-25, Figure 2
**DISCUSSION**			
Summary of evidence	19	Summarize the main results (including an overview of concepts, themes, and types of evidence available), link to the review questions and objectives, and consider the relevance to key groups.	29-30
Limitations	20	Discuss the limitations of the scoping review process.	31
Conclusions	21	Provide a general interpretation of the results with respect to the review questions and objectives, as well as potential implications and/or next steps.	34
**FUNDING**			
Funding	22	Describe sources of funding for the included sources of evidence, as well as sources of funding for the scoping review. Describe the role of the funders of the scoping review.	35-36

JBI = Joanna Briggs Institute; PRISMA-ScR = Preferred Reporting Items for Systematic reviews and Meta-Analyses extension for Scoping Reviews.

* Where *sources of evidence* (see second footnote) are compiled from, such as bibliographic databases, social media platforms, and Web sites.

† A more inclusive/heterogeneous term used to account for the different types of evidence or data sources (e.g., quantitative and/or qualitative research, expert opinion, and policy documents) that may be eligible in a scoping review as opposed to only studies. This is not to be confused with *information sources* (see first footnote).

‡ The frameworks by Arksey and O'Malley (6) and Levac and colleagues (7) and the JBI guidance (4, 5) refer to the process of data extraction in a scoping review as data charting.

§ The process of systematically examining research evidence to assess its validity, results, and relevance before using it to inform a decision. This term is used for items 12 and 19 instead of "risk of bias" (which is more applicable to systematic reviews of interventions) to include and acknowledge the various sources of evidence that may be used in a scoping review (e.g., quantitative and/or qualitative research, expert opinion, and policy document).

From: Tricco AC, Lillie E, Zarin W, O'Brien KK, Colquhoun H, Levac D, et al. PRISMA Extension for Scoping Reviews (PRISMAScR): Checklist and Explanation. Ann Intern Med. 2018;169:467–473. doi: 10.7326/M18-0850.

For peer review only - http://bmjopen.bmj.com/site/about/guidelines.xhtml
